# Supplementary material for: Flavin-enabled reductive and oxidative epoxide ring opening reactions
Source: Nat Commun. 2022 Aug 20;13:4896. doi: 10.1038/s41467-022-32641-1 (PMC9391479; doi:10.1038/s41467-022-32641-1)
Supplement: Supplementary file 1 — Supplementary Information [file 41467_2022_32641_MOESM1_ESM.pdf]

Supplementary Information

Flavin-Enabled Reductive and Oxidative Epoxide Ring Opening Reactions

Bidhan Chandra De et al

Content

Supplementary Materials and Methods 6

1.1. General information........ 6

1.2. Fermentation and isolation 7

1.3. General analytical HPLC methods 8

1.4. X-ray Crystallographic data........ 8

1.5. Structural elucidation of reaction products........ 10

Supplementary Tables 20

Supplementary Table 1. ^{1}H and ^{13}C NMR spectroscopic data of **3** and **12** in DMSO-d_6. 20

Supplementary Table 2. ^{1}H (700 MHz) and ^{13}C (175 MHz) NMR spectroscopic data of **7**, **8** and ^{1}H (700 MHz) NMR spectroscopic data of **7**-^{2}H and **8**-^{2}H in DMSO-d_6........ 21

Supplementary Table 3. Crystal data and structure refinement for **7** and **23**........ 23

Supplementary Table 4. ^{1}H (700 MHz) and ^{13}C (175 MHz) NMR spectroscopic data of **9**, **9**-^{18}O and **3**-^{18}O........ 24

Supplementary Table 5. ^{1}H (700 MHz) and ^{13}C (175 MHz) NMR spectroscopic data of **10**, **11** and **14** in DMSO-d_6........ 25

Supplementary Table 6. Crystal data and structure refinement for **10**. 26

Supplementary Table 7. ^{1}H (700 MHz) and ^{13}C (175 MHz) NMR spectroscopic data of **22** and **24** in DMSO-d_6........ 27

Supplementary Table 8. ^{1}H (700 MHz) and ^{13}C (175 MHz) NMR spectroscopic data of **23** and **25** in DMSO-d_6........ 28

Supplementary Table 9. ^{1}H (700 MHz) and ^{13}C (175 MHz) NMR spectroscopic data of **26** and **27** in DMSO-d_6........ 29

Supplementary Table 10. ^{1}H (700 MHz) and ^{13}C (175 MHz) NMR spectroscopic data of **38** in methanol-d_4. 30

Supplementary Table 11. ^{1}H (700 MHz) and ^{13}C (175 MHz) NMR spectroscopic data for **39**, **40** and **41** in DMSO-d_6........ 31

Supplementary Table 12. Crystal data and structure refinement for **40** 32

Supplementary Table 13. ^{1}H (700 MHz) and ^{13}C (175 MHz) NMR spectroscopic data of **47** in DMSO-d_6........ 33

Supplementary Table 14. Strains and plasmids used in this study. 34

Supplementary Figures........ 35

Supplementary Fig. 1. Naturally-occurring atypical angucyclines and FSTs proposed to be derived from epoxide precursors........ 35

Supplementary Fig. 2. SDS-PAGE analysis of recombinant enzymes and HPLC analysis of their reactions with FST C (**1**)........ 36

Supplementary Fig. 3. Spectroscopic data for FST C2 (**3**). 37

Supplementary Fig. 4. Spectroscopic data for FST B1 (**7**). 44

Supplementary Fig. 5. Comparison of experimental ECD and calculated ECD spectra of (1*R*,2*R*,3*S*)-**7** in MeCN. 51

Supplementary Fig. 6. Spectroscopic data for FST B2 (**8**).. 52
Supplementary Fig. 7. Comparison of experimental and calculated ECD spectra of **8** in MeCN. 59
Supplementary Fig. 8. Spectroscopic data for **9**..60
Supplementary Fig. 9. Comparison of the experimental ECD spectra of **3** with **9** and comparison of experimental and calculated ECD spectra of **9** in MeCN.. 67
Supplementary Fig. 10. Spectroscopic data for **10**..68
Supplementary Fig. 11. Spectroscopic data for **11**..75
Supplementary Fig. 12. LC-MS analysis of reactions of **1** with FAD/NADH using PBS buffers prepared with H_2O (control) or ^2H_2O. 82
Supplementary Fig. 13. Spectroscopic data for **7**-^{2}H. 83
Supplementary Fig. 14. Spectroscopic data for **8**-^{2}H. 87
Supplementary Fig. 15. LC-HRMS analysis of reactions of FST C (**1**) with FAD/NADH under air (control) or under $^{18}O_2$..91
Supplementary Fig. 16. HRESIMS spectroscopic data for **3**-^{18}O and **9**-^{18}O.........................92
Supplementary Fig. 17. Spectroscopic data of **9**-^{18}O 94
Supplementary Fig. 18. Comparison the ^{13}C NMR spectroscopic data of 9-^{18}O in DMSO-d_6 and 9 in DMSO-d_6:trifluoroacetic acid (TFA)-d_1 (100:1) 99
Supplementary Fig. 19. HPLC analysis of reactions of **1** and FAD/NADH in buffers of varying pH values...100
Supplementary Fig. 20. The spontaneous conversion of **7** to **8** (or **8** to **7**) in buffers with varying pH values...101
Supplementary Fig. 21. The stability of FST C (**1**) under varying conditions....................102
Supplementary Fig. 22. HPLC analysis for a time course assay of **1** and FAD/NADH and reactions of **1** with varying concentrations of FAD and NADH. 103
Supplementary Fig. 23. The comparison of UV spectra of **13** and FSTs and LC-HRMS analysis of **13**...104
Supplementary Fig. 24. HPLC analysis of reactions with **13**..105
Supplementary Fig. 25. Spectroscopic data for **14**. 106
Supplementary Fig. 26. HPLC analysis of reactions with **14**..109
Supplementary Fig. 27. HPLC analysis of reactions **1** with different Flavin cofactors.......110
Supplementary Fig. 28. HPLC and LC-MS analysis of FST F (**15**) reaction with FAD/NADH.. 111
Supplementary Fig. 29. Spectroscopic data for **22**..112
Supplementary Fig. 30. Spectroscopic data for **23.**...119
Supplementary Fig. 31. Spectroscopic data for **24**..126
Supplementary Fig.32. Spectroscopic data for **25**..133
Supplementary Fig. 33. HPLC and LC-MS analysis of 7-methyl-FST C (**16**) reaction with FAD/NADH. 140
Supplementary Fig. 34. HPLC and LC-MS analysis for 6-methyl-FST C (**17**) reaction with FAD/NADH.. 141
Supplementary Fig. 35. HPLC and LC-MS analysis of FST D (**18**) reaction with FAD/NADH. 142
Supplementary Fig. 36. HPLC and LC-MS analysis of FST S (**19**) reaction with FAD/NADH.. 143

Supplementary Fig. 37. HPLC and LC-MS analysis of DiFST H (**20**) reaction with FAD/NADH. 144

Supplementary Fig. 38. Spectroscopic data for **26**..145

Supplementary Fig. 39. Spectroscopic data for **27**..152

Supplementary Fig. 40. HPLC profile for FST Q (**21**) reaction with FAD/NADH 159

Supplementary Fig. 41. HPLC analysis of FAD/NADH-mediated reactions with expoxyikarugamycin (**28**) and capsimycin (**29**). 160

Supplementary Fig. 42. HPLC analysis of FAD/NADH-mediated reaction with epoxykinamycin FL-120B′ (**30**). 161

Supplementary Fig. 43. HPLC analysis of FAD/NADH-mediated reaction with 1,2-epoxy-cyclohexane (**31**) and 2,3-epoxy-cyclohexanone (**32**).. 162

Supplementary Fig. 44. HPLC analysis of FAD/NADH-mediated reaction with 16,17-epoxyprogesterone (**33**)..163

Supplementary Fig. 45. HPLC analysis of FAD/NADH-mediated reaction with 16,17-epoxypregnenolone (**34**)..164

Supplementary Fig. 46. HPLC analysis of FAD/NADH-mediated reaction with triptonide (**35**). 165

Supplementary Fig. 47. HPLC and LC-MS analysis of the reaction of auxarthrol H (**36**) with FAD/NADH. 166

Supplementary Fig. 48. Spectroscopic data for **38**..167

Supplementary Fig. 49. Comparison of experimental and calculated ECD spectra of **38** in MeOH...173

Supplementary Fig. 50. HPLC analysis of the reaction of menadione 2,3-epoxide (**37**) with FAD/NADH. 174

Supplementary Fig. 51. Spectroscopic data for **39.**..175

Supplementary Fig. 52. Spectroscopic data for **40**..181

Supplementary Fig. 53. Spectroscopic data for **41**..187

Supplementary Fig. 54. TLC profile for the reaction, UV spectra, and Optical rotation of vitamin K1 2,3-epoxide (**42**) with FAD/NADH..193

Supplementary Fig. 55. Structure features of substrated for FAD/NADH-mediated epoxide ring opening reactions. 194

Supplementary Fig. 56. HPLC analysis of the reactions of *trans*-1,3-diphenyl-2,3-epoxypropan-1-one (**45**) and chalcone α,β-epoxide (**46**) reaction with RslO5...195

Supplementary Fig. 57. Spectroscopic data for **47**..196

Supplementary Fig. 58. HPLC and LC-HRMS analysis of the reactions of **46** with RslO5 in coupling with GDHs..203

Supplementary Fig.59. HPLC and LC-HRMS analysis of the reactions of **1** with FAD/NAD(P)H in the coupled reactions with GDHs.. 204

Supplementary Fig. 60. The time course assay of reduced concentration of **1** and **7** with FAD and NADH...205

Supplementary Fig. 61. Determination of the equilibrium of the spontaneous conversion of **7** to **8** (or **8** to **7**)...206

Supplementary Fig. S62. The proposed mechanism for the formation of **12** from **13** and **7**.207

Supplementary Fig. 63. HPLC analysis of reactions of **1** with H_2O_2..................................208

Supplementary Fig. 64. The proposed mechanism for the formation of **3** and **9** from **13**. ..209

Supplementary Fig. 65. The proposed mechanism for the formation of **3** and **9** from **14**. ..210
Supplementary References 211

Supplementary Materials and Methods

1.1. General information

Commercially available compounds were purchased from Sigma-Aldrich, Aladdin, and Tokyo Chemical Industry Co., Ltd. (TCI) (Shanghai). Materials for column chromatography (CC) were silica gel (100−200 mesh; 300−400 mesh; Jiangyou Silica gel development, Inc.), Sephadex LH-20 (40−70 μm; Amersham Pharmacia Biotech AB), and YMC*GEL ODS-A (12 nm S-50 μm; YMC Company Ltd.). Thin-Layer-Chromatography (TLC, 0.1−0.2 mm or 0.3−0.4 mm) was conducted with pre-coated glass plates (silica gel GF254, 10−40 nm, Jiangyou Silica gel development, Inc.). Medium pressure liquid chromatography (MPLC) was performed on automatic flash chromatography (Cheetahtmmp 200, Bonna-Agela Technologies Co., Ltd.) with the wavelength at 304 nm. Semi-preparative HPLC was performed on a Hitachi HPLC station (Hitachi-L2130) with Diode Array Detector (Hitachi L-2455) using a Agilent Eclipse XDB-C18 (250 mm × 9.4 mm, 5 μm; Agilent technology Ltd., USA) or Phenomenex ODS column (250 mm × 10.0 mm, 5 μm; Phenomenex, USA), with a flow rate of 2.5 mL min^{-1}. HR-ESI-MS data were measured using a MaXis 4G UHR-TOFMS spectrometer (Bruker Daltonics Inc.). The optical rotations were recorded on a 341 Polarmeter (PerkinElme, Inc.). Deuterated NMR solvents were purchased from Cambridge Isotopes (Andover, MA). ^{1}H, ^{13}C, and 2D NMR spectra were recorded on Bruker AV-500 MHz NMR spectrometer or Bruker AVANCE III HD 700 MHz NMR spectrometer (Bruker Biospin GmbH), with tetramethylsilane (TMS) as an internal standard.

PCR amplifications were performed on an Eppendorf Mastercycler nexus GX2 using Trans start Fast Pfu-DNA polymerase from TransGen biotech Co., LTD (Beijing, China). Restriction enzymes and DNA ligase were purchased from Thermo Fisher Scientific Inc (MA, USA). Unless otherwise stated, other biochemicals and chemicals were purchased from standard commercial sources. All DNA manipulations in *E. coli* and *Streptomyces* were performed according to standard procedures. DNA sequencing was performed in Guangzhou IGE Biotech Co., Ltd. All primers used in this work were synthesized by Tianyihuiyuan Biotech Co., Ltd (Guangzhou). Cofactors Flavin adenine dinucleotide (FAD), alloxazine, Riboflavin, Flavin mononucleotide (FMN), Nicotinamide adenine dinucleotide (NADH) are purchased from Sigma-Aldrich, USA.

1.2. Fermentation and isolation

The wild type strain *Micromonospora. rosaria* SCSIO N160 were used for the isolation of FST C (**1**), FST F (**15**), and FST D (**18**).[1, 2] The strain *M. rosaria* SCSIO N160 were regularly propagated on Gauze's Medium No. 1 (soluble starch 2%, KNO_3 0.1%, K_2HPO_4 0.05%, $MgSO_4\cdot7H_2O$ 0.05%, $FeSO_4\cdot7H_2O$ 0.01%, pH 7.4) containing 3% sea salts. For preparing seed culture, a single colony of *M. rosaria* SCSIO N160 was inoculated into liquid production media consisting of starch 1%, glucose 2%, yeast extract 1%, corn powder 0.3%, beef extract 0.3%, $MgSO_4\cdot7H_2O$ 0.05%, K_2HPO_4 0.05%, $CaCO_3$ 0.2%, and sea salt 3% (adjusted to pH 7.2 − 7.4 before sterilization) in a 250 mL Erlenmeyer flask containing 50 mL media in a rotary shaker (200 rpm) at 28 °C for 3 days. Freshly prepared seed cultures were used for inoculating a 24 liter-scale fermentation using the production media supplemented with polystyrene resin (Amberlite XAD-16; 5% vol, 200 mL) at 28 °C for 7 days.[1] The fermentation cultures were centrifuged at 3900 rpm. The mycelia and XAD-16 resins were filtered through a metal sieve (40 mesh) to be separated from the liquid portion. The products in the supernatants were absorbed by XAD-16 resins. The resins were washed twice with H_2O and transferred to a glass column to be extracted with 6 L acetone. The acetone fractions were concentrated by rotary vacuum evaporation to afford an aqueous residue, which was extracted four times with 2 L butanone. The mycelia cake was sonicated and extracted with 6 L butanone. After drying under vacuum, the crude extracts (20 g) were obtained. The crude extracts were dissolved in 20 mL of CH_3Cl/MeOH (8:2, v/v) and subjected to normal phase silica column (100-200 mesh) chromatography. The elution was performed by four gradient flow of CH_3Cl/MeOH (0:100; 95:5; 90:10 and 0:100) to yield four fractions (Fr.1-4). Fr. 1 was collected and further purified by semi-preparative C18 reverse phase Medium Pressure Liquid Chromatography (MPLC) (YMC*GEL ODS-A, 12 nm S-50 μm, 30 × 2.5 cm I.D.), eluting with a linear gradient (MeCN/H_2O, 20%-100%, 20 mL min^{-1}, 230 min). Fr.1-2 was further purified by semi-preparative HPLC using Phenomenex C18 column (5 μm, 250 mm × 10 mm, 2.5 mL min^{-1}). An isocratic elution gradient of 70% A (H_2O with 0.8% formic acid) /30% B (MeCN) with a flow rate of 2.5 mL min^{-1} was applied to afford FST C (**1**) (250 mg). Fr. 2 was purified similar manner to afford FST F (**15**) (18 mg) and FST D (**18**) (3 mg). The identity and purity of isolated compounds were checked by comparing the HPLC retention time, UV spectrum, and LC-MS data with those of standard samples.

1.3. General analytical HPLC methods

HPLC analysis of non-enzyme reactions was carried out on the Agilent 1260 Infinity series instrument (Agilent Technologies Inc., USA) using a reversed phase C18 column (Kinetex® 5μm C18 100 Å, LC Column 150 × 4.6 mm, Phenomenex, USA) or a polar column (Comixsep®, P/N FMG-BPF5-EONU, Polar BiPFP 5u, 250 × 4.6 mm, China) with UV detection at 256 nm or 304 nm under the following program: solvent system (solvent A, 10% MeCN in water supplementing with 0.1% formic acid; solvent B, 90% MeCN in water); 5% B to 80% B (0 to 20 min), 80% B to 100% B (20 to 21 min), 100% B (21 to 24 min), 100% B to 5% B (24 to 25 min), 5% B (25 to 30 min); flow rate at 1 mL min^{-1}.

1.4. X-ray crystallographic data

Crystal data for FST B1 (**7**) mp 210 °C, $C_{18}H_{15.54}O_{6.77}$ (M = 340.12 g/mol); orthorhombic, space group $P2_12_12_1$, a = 6.5747(2) Å, b = 12.7647(3), c = 17.4860(4) Å, α = 90°, β = 90°, γ = 90°, V = 1467.50(7) $Å^3$, Z = 4, T = 100.00(10) K, μ(Cu Kα) = 1.005 mm^{-1}, D_{calc} = 1.539 g/cm^3, 6446 reflections measured (8.576 ≤ 2Θ ≤ 148.056), 2857 unique (R_{int} = 0.0199, R_{sigma} = 0.0176) which were used in all calculations. The final R_1 was 0.0404 (I > 2σ(I)) and $w$$R_2$ was 0.01105 (all data).

Crystal Data for FST C4 (**10**) mp 214 °C, $C_{18}H_{16}O_9$ (M = 376.31 g/mol): triclinic, space group $P1$ (no. 1), a = 8.0646(2) Å, b = 9.4581(4) Å, c = 12.3793(4) Å, α = 105.647(3), β = 91.092(2), γ = 110.786(3), V = 843.13(5) $Å^3$, Z = 2, T = 100.00(10) K, μ(Cu Kα) = 1.035 mm^{-1}, D_{calc} = 1.482 g/cm^3, 17982 reflections measured (7.478 ≤ 2Θ ≤ 148.556), 6389 unique (R_{int} = 0.0248, R_{sigma} = 0.0264) which were used in all calculations. The final R_1 was 0.0333 (I > 2σ(I)) and wR_2 was 0.0891 (all data).

Crystal Data for 1-*O*-methyl-FST B1 (**23**) mp 228 °C, $C_{20}H_{20}O_7$ (M = 372.36 g/mol): orthotriclinic, space group $P2_12_12_1$, a = 6.6367(4) Å, b = 14.0428(7) Å, c = 17.9812(12) Å, α = 90, β = 90, γ = 90, V= 1675.81(17) $Å^3$, Z = 4, T = 100.00(10) K, μ(Cu Kα) = 0.940 mm^{-1}, D_{calc} = 1.476 g/cm^3, 9395 reflections measured (7.988 ≤ 2Θ ≤ 148.046), 3288 unique (R_{int} = 0.0623, R_{sigma} = 0.0592) which were used in all calculations. The final R_1 was 0.0663 (I > 2σ(I)) and wR_2 was 0.2017 (all data).

Crystal Data for Compound **40** mp 178 °C, $C_{11}H_8O_3$ (M = 188.17 g/mol): monoclinic, space group $P2_1$, a = 7.6523(5) Å, b = 4.7776(3) Å, c = 11.6829(9) Å, α =

90°, β = 90.544(8)°, γ = 90°, V = 427.10(5) $Å^3$, Z = 2, T = 100.01(12) K, μ(Cu Kα) = 427.10(5) mm^{-1}, D_{calc} = 1.463 g/cm^3, 1758 reflections measured (7.568° ≤ 2Θ ≤ 148.544°), 1084 unique (R_{int} = 0.0445, R_{sigma} = 0.0497) which were used in all calculations. The final R_1 was 0.0743 (I > 2σ(I)) and wR_2 was 0.1832 (all data).

1.5. Structural elucidation of reaction products

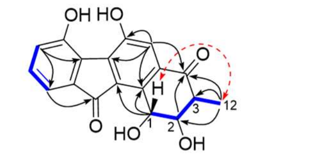

7

COSY — HMBC ⌒ NOESY

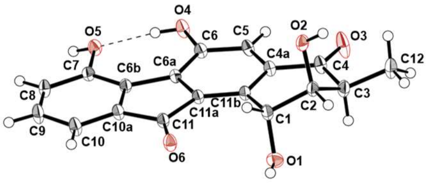

X-ray crystal structure of **7**

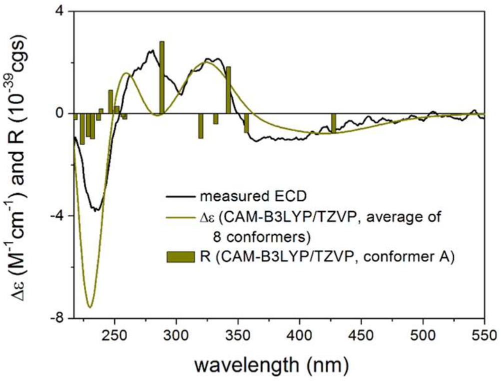

Comparison of experimental ECD and calculated ECD spectrum of (1*R*,2*R*,3*S*)-**7** in MeCN

FST B1 (**7**) was isolated as yellow powder, $[\alpha]_D^{25}$ -9.4 (*c* 0.05, MeOH). The molecular formula of **7** was established as $C_{18}H_{14}O_6$ according to HRESIMS *m/z* 325.0715 $[M - H]^-$ (calcd 325.0718, Supplementary Fig. 4). Extensive NMR analysis suggested that compound **7** had the same planar structure as FST B (Supplementary Table 2, Supplementary Fig. 4), however, the absolute configuration of FST B had not been previously established.[3] NOESY correlations between H-1 & H_3-12 as well as H-1 & OH-2 (Supplementary Fig. 4) were consistent with the *cis*-configuration of H-2/H-3 in **7**. The electronic circular dichroism (ECD) spectrum of **7** was acquired and compared with that computed for **7** using the solution TDDFT-ECD methodology to support its assignment as the (1*R*,2*R*,3*S*) configuration (Supplementary Fig. 5). This assignment was confirmed by a single crystal X-ray analysis (CCDC 2036399) using Cu Kα radiation with a Flack parameter value of 0.04(13) (Supplementary Table 3).

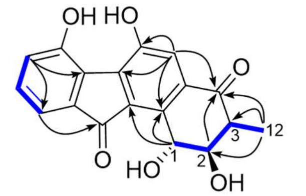

8

COSY — HMBC ⌒ NOESY

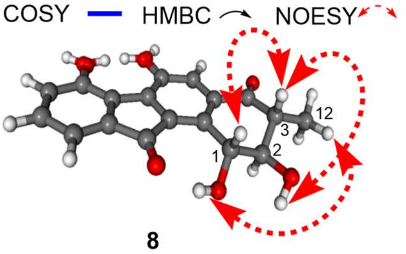

8

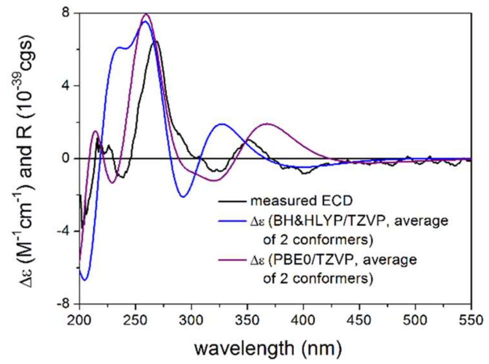

Comparison of experimental and calculated ECD spectrum of (1*R*,2*R*,3*R*)-**8** in MeCN

FST B2 (**8**) was isolated as yellow powder, $[\alpha]_D^{25}$ -8.8 (*c* 0.05, MeOH). The molecular formula was deduced to have $C_{18}H_{14}O_6$ based on HRESIMS *m/z* $[M - H]^-$ 325.0728 (calcd 325.0718) (Supplementary Fig. 6). The NMR spectra of **8** was essentially identical to that of **7** (Supplementary Table 2, Supplementary Fig. 6), the NOESY correlations of the 2-OH and H-3 indicated a *trans*-configuration of the H-2 and H-3 bond in **8**, which is different from that of **7** (Supplementary Fig. 4). The *trans* configuration of the 1-OH and 2-OH of **8** was further supported by NOESY correlations of H-1 and H-3 as well as 1-OH and 3-Me (Supplementary Fig. 6). Based on comparison of the experimental and calculated ECD spectra carried out on the (1*R*,2*R*,3*R*) stereoisomer of FST B1 (**7**), compound **8** was deduced to possess the (1*R*,2*R*,3*R*) configuration (Supplementary Fig. 7). In order to distinguish the two stereoisomers of FST B, **7** and **8** were named FST B1 and FST B2, respectively.

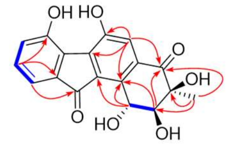

9

COSY — HMBC NOESY

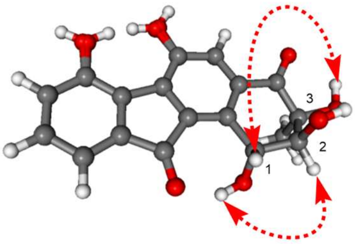

9

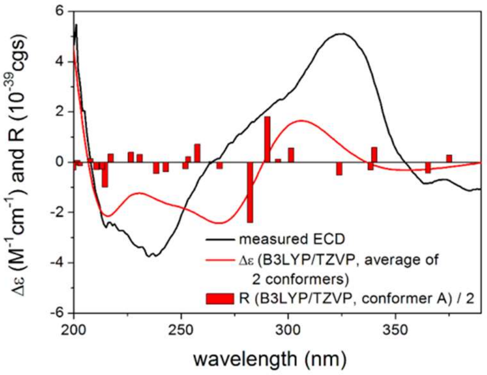

Comparison of experimental and calculated ECD spectra of (1R,2S,3S)-**9** in MeCN

FST C3 (**9**) was obtained as yellow powder, $[\alpha]_D^{25}$ 7.4 (*c* 0.05, MeOH). The molecular fomula of **9** was established as $C_{18}H_{14}O_7$ by HRESIMS *m/z* 341.0668 $[M - H]^-$ (calcd 341.0667), same as that of **3**. The ^1H, ^{13}C, and 2D NMR data of **9** were highly similar to those of **3** (Supplementary Table 4, Supplementary Fig. 8). However, NOESY correlations of H-1 & 2-OH as well as 1-OH & 3-Me (Supplementary Fig. 8, Fig. 17) indicated a *cis* configuration of the 2-OH and 3-OH, differing from the *trans* configuration in **3**. Consequently, the absolute configuration of **9** was assigned as (1*R*,2*S*,3*S*) by means of the good agreement observed between the experimental ECD of **9** and the TDDFT-ECD spectrum of (1*R*,2*S*,3*S*)-**9** (Supplementary Fig. 9). Compound **9** was designated FST C3.

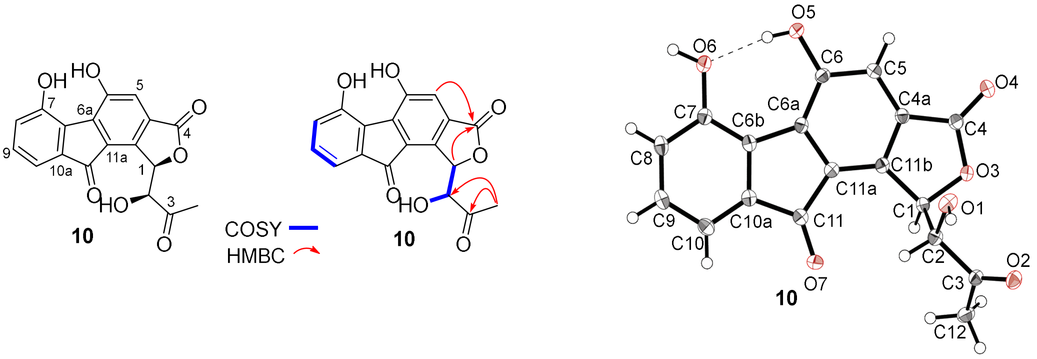

FST C4 (**10**) was obtained as yellow powder, $[\alpha]_D^{25}$ -12.6 (*c* 0.05, MeOH). The molecular formula of **10** was established as $C_{18}H_{11}O_7$ by HRESIMS *m/z* 339.0519 [M – H]$^-$ (calcd 335.0510), requiring 13 degrees of unsaturation. Careful analysis of 1D and 2D NMR data of **10** (Supplementary Table 5, Supplementary Fig. 10) indicated that the rings B, C, and D of **10** were the same as those of **1**. These assignments were supported by the presence of characteristic aromatic ABC spin system (δ_H 6.59, 1H, dd, J = 0.7, 8.2 Hz; δ_H 6.93, 1H, dd, J = 7.2, 8.2 Hz; δ_H 6.67, 1H, dd, J = 0.7, 8.1 Hz) and HMBC correlations from H-5 to C-6/C-6a/C-11b, from H-8 to C-6b/C-10, from H-9 to C-7/C-10a, and from H-10 to C-8/C-6b. Besides the signals for these fragments, the remaining ^{13}C NMR data of **10** showed 4 carbons resonances for two sp^3 methine carbons (δ_C 81.2; δ_C 75.5), and two carbonyl carbons (δ_C 210.1; δ_C 170.4). Detailed analysis of 1D and 2D NMR data, these signals were assigned as a (1-hydroxy-2-oxopropyl)furan-2-one unit fused to the ring B at C-4a and C-11b, which were supported by the COSY correlations of H-1 and H-2, HMBC correlations from H_3-12 to C-2 (δ_C 210.1)/C-3, and from H-1/H-5 to C-4 (δ_C 170.4), as well as three less degrees of unsaturation. Finally, high-quality crystals of **10** were obtained to allow the successful performance of a single-crystal X-ray diffraction experiment using Cu Kα radiation (Supplementary Table 6 CCDC 2129081), to confirm the planar structure of **10**, and to establish the absolute configuration of **10** as (1*R*,2*S*) with the Flack -0.01(5) (Supplementary Table 6).

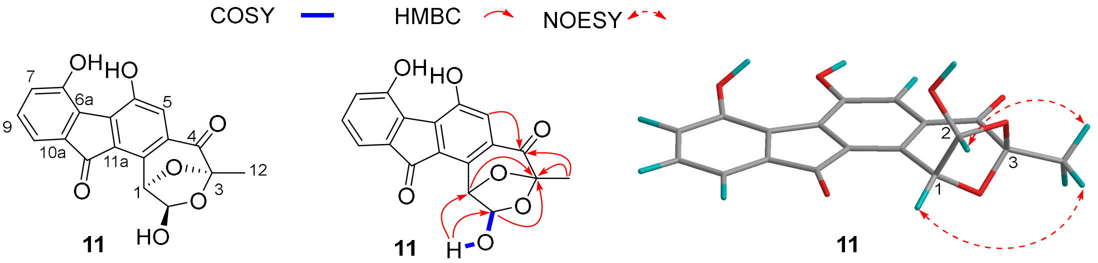

FST C5 (**11**) was obtained as yellow powder, $[\alpha]_D^{25}$ -3.2 (*c* 0.05, MeOH). the molecular formula of **11** was established as $C_{18}H_{12}O_7$ by HRESIMS *m/z* 339.0512 $[M - H]^-$ (calcd 339.0510). Careful analysis of ^{1}H NMR data of **11** (Supplementary Table 5, Supplementary Fig. 11) showed that the rings B, C, and D were the same as those of **1**, which was confirmed by detailed assignment of 2D NMR data of **11**. In additions to signals for fragments of rings B–D, the remaining ^{13}C NMR data of **11** showed 4 carbons resonances for two sp^3 methine carbons (δ_C 78.5; δ_C 98.0), one non-protonated sp^3 hybridized oxygenated carbon (δ_C 106.7), and one carbonyl carbons (δ_C 189.0), which were assigned as a 6,8-dioxabicyclo[3.2.1]oct-2-en-4-one unit fused to the ring B at C-4a and C-11b. This assignment was further supported by the COSY correlations of 2-OH and H-2, HMBC correlations from H_3-12 to C-3/C-4, from 2-OH to C-1/C-2, from H-1/H-2 to C-3, from H-5 to C-4, as well as three less degrees of unsaturation. Thus, the planar structure of **11** was assigned. The relative configuration of **11** was assigned by the NOESY correlations of H-1/H-2/H_3-12 (Supplementary Fig. 11), which indicated that H-1/H-2/H_3-12 located on the same side of the dioxolane ring. Given the configuration of C-1 remained unchanged, the absolute configuration of **11** was tentatively assigned as (1*R*,2*R*,3*R*).

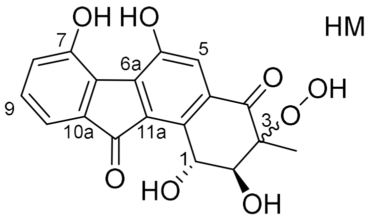

14

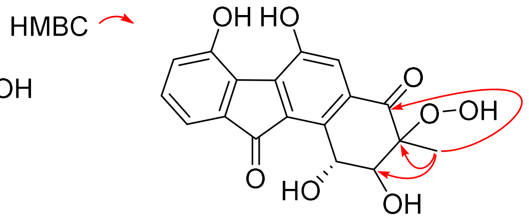

14

Hydroperoxyfluostatin (**14**) was obtained as yellow powder and deduced the molecular formula $C_{18}H_{14}O_8$ by HRESIMS *m/z* 357.0613 $[M - H]^-$ (calcd 357.0616) (Supplementary Fig. 25), which is 34 mass units greater than that of **1**. Careful analysis of ^{1}H NMR data (Supplementary Table 5, Supplementary Fig. 25) of **14** indicated the presence of characteristic aromatic ABC spin system (δ_H 6.56, 1H, dd, J = 0.5, 8.0 Hz; δ_H 6.91, 1H, dd, J = 6.9, 8.0 Hz; δ_H 6.68, 1H, d, J = 0.5, 6.9 Hz) and one singlet aromatic proton (δ_H 6.92, 1H, s), which suggested that the rings B, C, and D of **14** were the same as those of **1**. The deshielded C-2 ($\delta_{C,}$ 72.0, Δ 8.0 ppm) and C-3 ($\delta_{C,}$ 87.6, Δ 28.1 ppm) (assigned by HMBC correlations) in **14** suggested that the 2,3-epoxy ring in **1** is opened in **14**. The presence of a hydroperoxy group at C-3 in **14** was supported by the molecular formula of **14** and the chemical shift value of C-3 ($\delta_{C,}$ 87.6). Thus, the planar structure of

14 was assigned. Given **14** was the ring opened product of **1** and the observed conversion of **14** to both **3** and **9**, compound **14** is likely a mixture of the (1*S*,2*S*,3*R*) and (1*S*,2*S*,3*S*) stereoisomers.

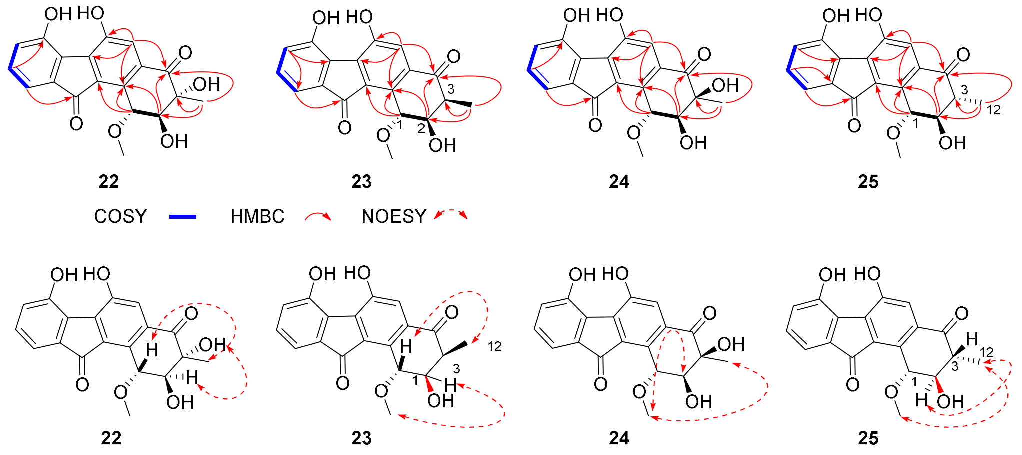

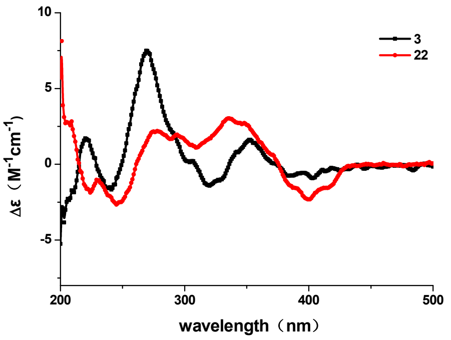

Comparison of experimental ECD spectrum of **3** and **22**

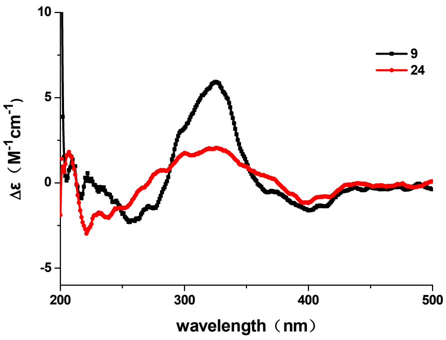

Comparison of experimental ECD spectrum of **9** and **24**

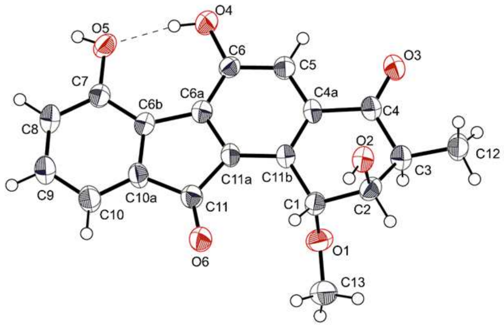

X-ray crystallographic structure of **23**

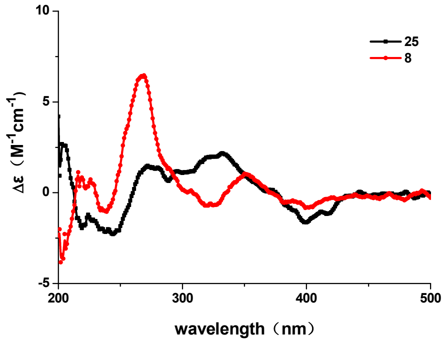

Comparison of experimental ECD spectrum of **8** and **25**

1-*O*-methyl-FST C2 (**22**) was obtained as yellow powder, $[\alpha]_D^{25}$ 6.4 (*c* 0.05, MeOH). The molecular formula of **22** was assigned as $C_{19}H_{16}O_7$ based on HRESIMS *m/z* 335.0830 $[M - H]^-$ (calcd 335.0823) ^1H and ^{13}C NMR data (Supplementary Table 7, Supplementary Fig. 29) of **22** were highly similar to those of FST F (**15**). The deshielded C-2 (δ_C, 71.6, Δ 12.2 ppm) and C-3 (δ_C, 75.0, Δ 18.2 ppm) in **22** suggested the presence of

a vicinal diol subunit at C-2/C-3, instead of 2,3-epoxy ring in **15**. This assignment was supported by the HMBC correlations from H_3-12 to C-2/C-3/C-4. Further detailed analysis of 2D NMR data of **22** confirmed the its assigned planar structure. The relative configuration of **22** was deduced from the NOESY correlations of H-1 & H_3-12 as well as H-2 & OH-3 which suggested the *trans*-configuration of H-1/H-2, and the *cis*-configuration of H-2/2-OH in **22** (Supplementary Fig. 29). Based on comparison of the experimental ECD spectra of **22** and **3**, the absolute configuration of **22** was assigned as (1*R*,2*S*,3*R*).

1-*O*-methyl-FST B1 (**23**) was obtained as yellow crystal, $[\alpha]_D^{25}$ -14.8 (*c* 0.05, MeOH). The molecular formula of **23** was assigned as $C_{19}H_{16}O_6$ based on HRESIMS *m/z* 339.0881 $[M-H]^-$ (calcd 339.0874), corresponding to 12 degrees of unsaturation. ^1H and ^{13}C NMR data of **23** (Supplementary Table 8, Supplementary Fig. 30) were similar to those of FST F (**15**).[1] The difference was that **23** had one more methine and one less non-protonated carbon than that of **15**, which suggested 2,3-epoxy ring in **15** has opened. These suggestions were supported the COSY correlations of H-3 and H_3-12, and HMBC correlations from H_3-12 to C-2/C-3/C-4. Further detailed assignment of 2D NMR data confirmed the structure of **23** and designated 1-*O*-methyl-FST B1 (**23**). The relative configuration of **23** was deduced from NOESY correlations of H-1 & H_3-12 as well as H_3-13 & H-2 (Supplementary Fig. 30), which suggested the *cis* configuration of H-1/H-2 in **23**. However, the relative configuration of H-2 and H-3 could not be assigned due to the lack of observed NOESY correlations. Finally, high-quality crystals of **23** were obtained to allow the successful performance of a single-crystal X-ray diffraction experiment using Cu Kα radiation to establish the absolute configuration of **23** as (1*R*,2*R*,3*S*) based on the Flack parameter value of 0.0 (3) (Supplementary Table 3, CCDC 2036400).

1-*O*-methyl-FST C3 (**24**) was obtained as yellow-red powder, $[\alpha]_D^{25}$ 7.4 (*c* 0.05, MeOH). The molecular formula of **24** was assigned as $C_{19}H_{16}O_7$ based on HRESIMS *m/z* 355.0822 $[M-H]^-$ (calcd 355.0823), the same as that of **22**. The high similarity of the 1D and 2D NMR spectra of compounds **24** (Supplementary Table 7, Supplementary Fig. 31) and **22** indicated that they shared the same planar structure. The NOESY correlations of H_3-13 & H-2 and H_3-13 & H_3-12 suggests the *trans*-configuration of H-1/H_3-12 (Supplementary Fig. 31), different from that of **22**. To determine the absolute

configurations of **24**, the ECD spectra was compared with that of **9**. The almost identical Cotton effects of **24** and **9** suggested that **24** should have the (1*R*,2*S*,3*S*) configuration.

1-*O*-methyl-FST B2 (**25**) was obtained as yellow powder, $[\alpha]_D^{25}$ 3.8 (*c* 0.05, MeOH). The molecular formula of **25** was assigned as $C_{19}H_{16}O_6$ based on HRESIMS *m/z* 339.0881 $[M-H]^-$ (calcd 339.0874), the same as that of **23**. Extensive NMR analysis suggested that **25** (Supplementary Table 8, Supplementary Fig. 32) and **23** had the same planar structure. The NOESY correlations of the H_3-13 & H_3-12 indicated a *cis*-configuration of H-1 and H-3 in **25** (Supplementary Fig. 32), which is different from that in **23**. The *trans*-configuration of H-2 and H-3 in **25** was determined by the NOESY correlations of H-2 & H_3-12. Based on comparison of the experimental ECD spectra of **25** and **8**, the absolute configuration of **25** was assigned as (1*R*,2*R*,3*R*).

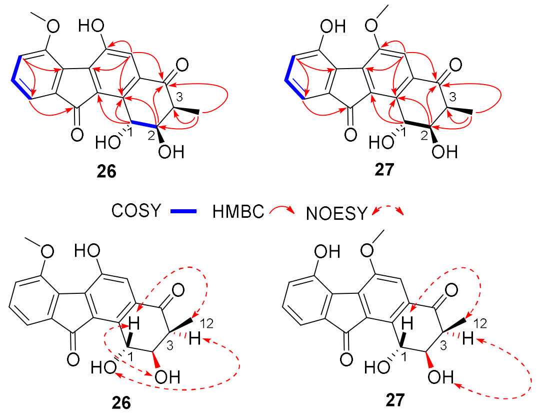

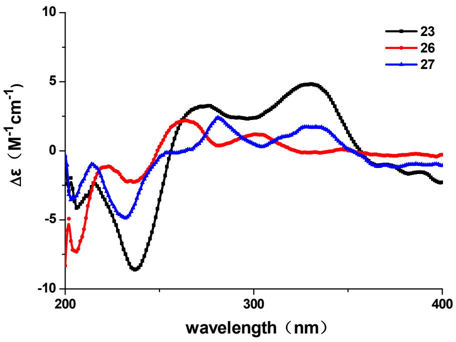

Comparison of experimental ECD spectrum of **23**, **26** and **27**.

FST M (**26**) was obtained as yellow powder, $[\alpha]_D^{25}$ 4.4 (*c* 0.05, MeOH). The molecular formula of **26** was established as $C_{19}H_{16}O_6$ by HRESIMS *m/z* 339.0881 $[M-H]^-$ (calcd 339.0874). The ^{1}H and ^{13}C NMR data (Supplementary Table 9, Supplementary Fig. 38) of **26** were similar to that of **16**. The difference was that **26** has a doublet methyl signal, instead of the singlet methyl signal in **16**, suggesting that the 2,3-epoxy in **16** was opened and transformed to the secondary alcohol in **26**. Further detailed analysis of 2D NMR data assigned the structure of **26**. The *trans* configuration of H-1/H-2 and *cis* configuration of H-2/H-3 in **26** was deduced by ROESY correlations of H-1 & H_3-12, 2-OH & H_3-12, and H-1 & 2-OH (Supplementary Fig. 38).

6-*O*-methyl-FST B1 (**27**) was obtained as yellow powder, $[\alpha]_D^{25}$ 5.6 (*c* 0.05, MeOH). The molecular formula of **27** was established as $C_{19}H_{16}O_6$ by HRESIMS *m/z* 339.0885 $[M-H]^-$ (calcd 339.0874). The ^{1}H, ^{13}C NMR data (Supplementary Table 9,

Supplementary Fig. 39) of **27** were similar to that of **17**. The difference was that **27** has a doublet methyl signal, instead of the singlet methyl signal in **17**, which suggested that the 2,3-epoxy in **17** was opened and transformed to the secondary alcohol in **27**. Further detailed analysis of 2D NMR data assigned the structure of **27**. The relative configuration of **27** was deduced by NOESY correlations of H-1 & H_3-12and H_3-12 & 2-OH, which supported the *trans*-configuration of H-1/H-2, and the *cis*-configuration of H-2/H-3 in **27** (Supplementary Fig. 39).

To determine the absolute configurations of **26** and **27**, their ECD spectra were compared with that of **23**. The almost identical cotton effects of **23**, **26** and **27** suggested that compounds **26** and **27** should have the (1*R*,2*R*,3*S*) configuration, the same as that of **23**.

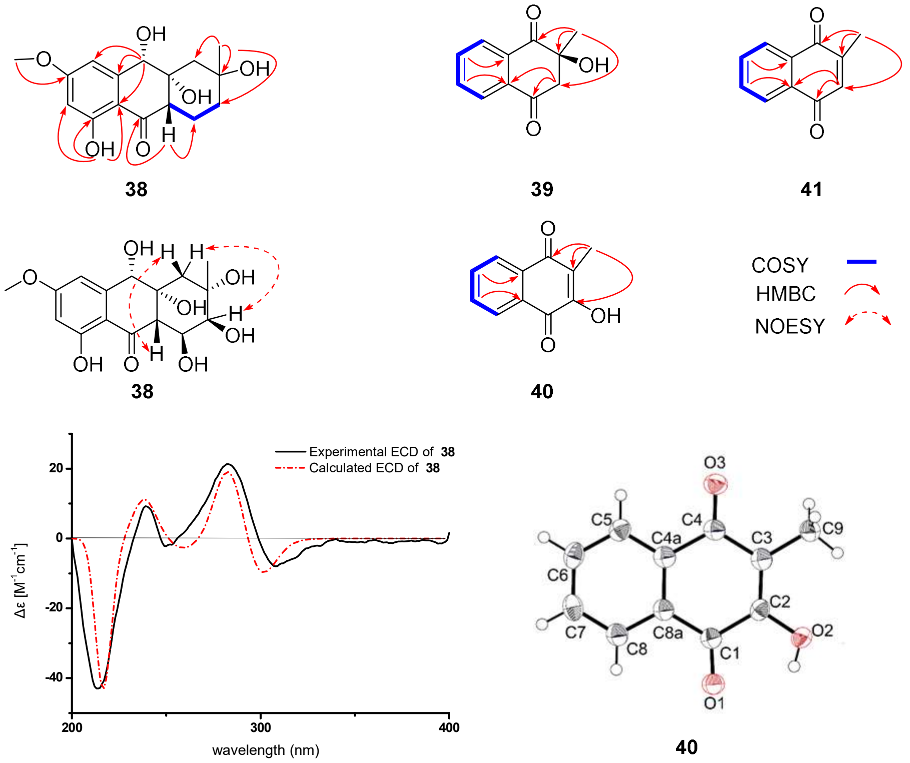

Compound **38** was obtained as pale-white powder, $[\alpha]_D^{25}$ 2.8 (*c* 0.05, MeOH). The molecular formula of **38** was assigned as $C_{16}H_{20}O_8$ based on HRESIMS *m/z* 339.1089 [M – H]$^-$ (calcd 339.1085), indicating 7 degrees of unsaturation. The ^{1}H and ^{13}C NMR data of **38** (Supplementary Table 10, Supplementary Fig. 48) displayed high similarity to those of **36**. The difference was that **38** had one more methine, instead of a non-protonated carbon in **36**, which suggested that the ring of 4a,9a-epoxy in **36** was opened in **38** and transformed to the secondary alcohol. The careful analysis 2D NMR data assigned the structure of **38**. According to the NOESY correlations of H-4a/H-1a, and H-1b/H-3 was

deduced to locate on the same side as 3-OH (Supplementary Fig. 48). Consequently, the absolute configurations of H-4a and H-9a were assigning to (4a*S*,9a*R*), which were deduced by comparison of the experimental and calculated ECD spectra of (4a*S*,9a*R*)-**38**.

3-Hydroxy-3-methyl-2,3-dihydronaphthalene-1,4-dione (**39**) was obtained as white powder, $[\alpha]_D^{25}$ 4.6 (*c* 0.05, MeOH). The molecular formula of **39** was assigned as $C_{11}H_{10}O_3$ based on HRESIMS *m/z* 189.0550 $[M - H]^-$ (calcd 189.0557), indicating 7 degrees of unsaturation. The ^1H, ^{13}C, and HSQC spectra of **39** (Supplementary Table 11, Supplementary Fig. 51) indicated the presence of methylene group, which suggested the absence of the 2,3-epoxy ring of **37** in **39**. This suggestion was supported by HMBC correlations from H_3-11 to C-2/C-3/C-4 and from H-2 to C-2/C-4/C-8a. Detailed 2D NMR analysis assigned the planar structure of **39**. Given that **39** was epoxide ring-opening product of **37**, the (3*S*) absolute configuration of C-3 was tentatively assigned.

2-Hydroxy-3-methyl-1,4-naphthoquinone (phthiocol, **40**) was obtained as white powder, $[\alpha]_D^{25}$ 5.2 (*c* 0.05, MeOH). The molecular formula of **40** was assigned as $C_{11}H_9O_3$ based on HRESIMS *m/z* 189.0550 $[M - H]^-$ (calcd 189.0546), indicating 8 degrees of unsaturation. A comparison of ^1H and ^{13}C NMR spectral data of **40** (Supplementary Table 11, Supplementary Fig. 52) and **39** revealed their high structural similarity. Compound **40** differed from **39** in the presence of resonances at δ_C 158.9 and δ_C 119.1, which were assigned to the $\Delta^{2,3}$ double bond in **40**. Detailed 2D NMR analysis confirmed the structure of **40**. The structure of **40** was further confirmed by a single-crystal X-ray structure analysis (Supplementary Table 12, CCDC 2036401) as 2-hydroxy-3-methyl-1,4-naphthoquinone.

Compound **41** was obtained as white powder. The molecular formula of **41** was assigned as $C_{11}H_8O_2$ based on HRESIMS *m/z* 173.0601 $[M - H]^-$ (calcd 173.0597), indicating 8 degrees of unsaturation. A comparison of ^1H and ^{13}C NMR spectral data of **41** (Supplementary Table 11, Supplementary Fig. 53) and **40** revealed their high structural similarity. The difference was that the hydroxyl group at C-2 in **40** was replaced by a hydrogen in **41**, which was supported by 2D NMR data. Thus, the structure of **41** was assigned to be 3-methylnaphthalene-1,4-dione.

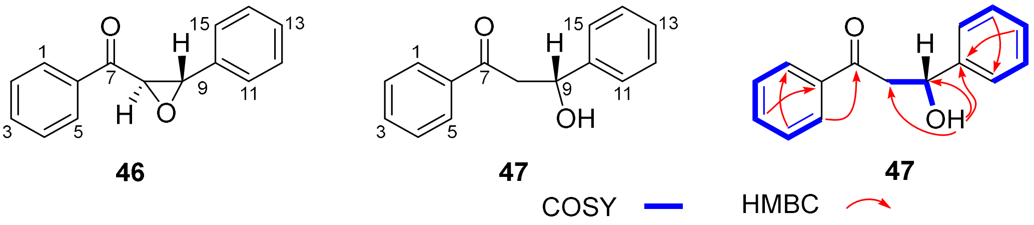

Compound **47** was obtained as yellow powder, $[\alpha]^{25}_{D}$ -8.6 (*c* 0.05, MeOH). The molecular formula of **47** was established as $C_{15}H_{14}O_2$ by HRESIMS *m/z* 249.0894 $[M + Na]^+$ (calcd 249.0886). The ^1H, ^{13}C and HSQC spectra of **47** (Supplementary Table 13, Supplementary Fig. 57) indicated the presence of one methine (δ_H 5.14, m, H-9; δ_C 69.8 C-9) and one methylene proton signals (δ_H 3.19, dd, J = 4.4, 15.8, H-8a, δ_H 3.43, dd, J = 8.5, 15.8, H-8b; δ_C 69.8, C-8), which suggested that 8,9-epoxy ring in **46** was opened in **47**. This suggestion was supported by COSY correlations of H_2-8 & H-9 and HMBC correlations from 9-OH to C-8/C-9/C-10. Further detailed analysis of 2D NMR data assigned the structure of **47**. Given that **47** was epoxide ring-opening product of **46**, the absolute configuration of C-9 was tentatively assigned as (9*R*).

Supplementary Tables

Supplementary Table 1. ^{1}H and ^{13}C NMR spectroscopic data of 3 and 12 in DMSO-*d*$_6$.

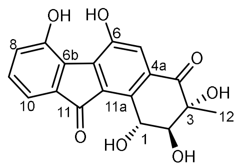

3

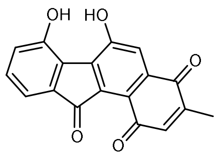

12

Position	**3**[a]		**12**[b]	
	δ_C, type	δ_H, mult. (J in Hz)	δ_C, type	δ_H, mult. (J in Hz)
1	67.3, CH	5.12, dd (4.4, 4.4)	181.8, C	
2	75.8, CH	3.74, dd (4.5, 4.5)	137.8, CH	6.66, s
3	75.2, C		144.4, C	
4	197.6, C		186.3, C	
4a	131.0, C		133.3, C	
5	122.1, CH	6.93, s	122.0, CH	7.01, overlapping
6	159.5, C		167.3, C	
6a	140.6, C		140.5, C	
6b	129.7, C		128.2, C	
7	160.1, C		156.2, C	
8	126.2, CH	6.55, d (8.3)	127.7, CH	6.69, d (7.9)
9	130.5, CH	6.90, dd (8.3, 7.1)	130.4, CH	7.01, overlapping
10	111.4, CH	6.65, d (7.1)	113.3, CH	6.80, d (7.0)
10a	134.9, C		135.7, C	
11	196.6, C		192.3, C	
11a	131.0, C		134.6, C	
11b	129.2, C		118.1, C	
12	20.8, CH_3	1.19, s	15.8, CH_3	1.99, s
1-OH		5.76, d (4.4)		
2-OH		5.38, d (4.6)		
3-OH		5.68, s		

[a]Data were recorded on a Bruker Avance 700 MHz NMR spectrometer in DMSO-*d*$_6$ with TMS as an internal standard.

[b]Data were recorded on a Bruker Avance 600 MHz NMR spectrometer in DMSO-*d*$_6$ with TMS as an internal standard.

Supplementary Table 2. ^{1}H (700 MHz) and ^{13}C (175 MHz) NMR spectroscopic data of 7, 8 , 7-^{2}H and 8-^{2}H in DMSO-d_6.

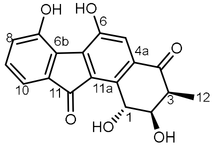

7

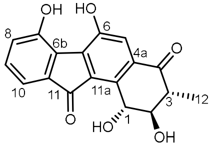

8

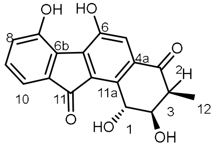

7-^{2}H

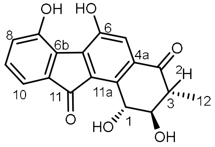

8-^{2}H

Position	**7**		**8**		**7**-^{2}H		**8**-^{2}H	
	δ_C, type	δ_H, mult. (J in Hz)	δ_C, type	δ_H, mult. (J in Hz)	δ_C, type	δ_H, mult. (J in Hz)	δ_C, type	δ_H, mult. (J in Hz)
1	63.6, CH	5.45, d (3.4)	69.2, CH	4.94, dd (6.1, 3.0)	64.1, CH	5.44, d (3.5)	68.3, CH	5.20, d (4.9)
2	74.8, CH	4.01, dd (3.4, 2.4)	74.2, CH	3.60, m	75.3, CH	4.00, d (3.5)	74.3, CH	3.80, d (4.9)
3	41.6, CH	3.17, overlapped	47.2, CH	2.52, overlapped	41.7, CH		47.4, CH	
4	197.7, C		198.4, C		198.3, C		199.1, C	
4a	132.4, C		131.5, C		132.8, C		132.4, C	
5	119.6, CH	7.48, s	121.4, CH	6.92, s	120.1, CH	7.44, s	121.5, CH	7.29, s
6	149.2, C		158.3, C		150.4, C		155.5, C	
6a	133.7, C		140.2, C		134.4, C		138.5, C	
6b	125.5, C		129.2, C		120.3, C		128.3, C	
7	150.7, C		159.7, C		152.0, C		156.8, C	
8	124.0, CH	7.09, d (8.1)	126.2, CH	6.55, d (8.1)	124.4, CH	7.05, d (8.1)	125.9, CH	6.90, d (7.3)
9	131.7, CH	7.31, dd (8.1, 7.0)	130.1, CH	6.89, dd (8.1, 7.0)	132.0, CH	7.29, dd (8.1, 7.1)	131.3, CH	7.17, dd (7.3, 6.8)

10	116.3, CH	7.21, d (7.0)	111.2, CH	6.66, d (7.0)	116.2, CH	7.16, d (7.1)	113.7, CH	7.01, d (6.8)
10a	135.4, C		134.2, C		135.8, C		135.1, C	
11	192.4, C		197.1, C		193.1, C		198.5, C	
11a	134.1, C		130.4, C		134.5, C		131.3, C	
11b	134.3, C		131.0, C		134.6, C		131.7, C	
12	11.4, CH_3	1.16, d (6.8)	13.1, CH_3	1.18, d (7.1)	11.8, CH_3	1.15, s	14.3, CH_3	1.22, s
1-OH				5.58, d (3.0)				5.46, s
2-OH				5.24, d (3.1)				5.28, s

Assignments were based on DEPT, HSQC, COSY, HMBC, and NOESY experiments.

Supplementary Table 3. Crystal data and structure refinement for 7 and 23

Identification code	**7**	**23**
Empirical formula	$C_{18}H_{15.54}O_{6.77}$	$C_{20}H_{20}O_7$
Formula weight	340.12	372.36
Temperature/K	99.9(7)	99.8(9)
Crystal system	orthorhombic	orthorhombic
Space group	$P2_12_12_1$	$P2_12_12_1$
a/Å	6.5747(2)	6.6367(4)
b/Å	12.7647(3)	14.0428(7)
c/Å	17.4860(4)	17.9812(12)
α/°	90	90
β/°	90	90
γ/°	90	90
Volume/Å^3	1467.50(7)	1675.81(17)
Z	4	4
ρ_{calc}g/cm^3	1.539	1.476
μ/mm^{-1}	1.005	0.940
F(000)	711.0	784.0
Crystal size/mm^3	0.5 × 0.5 × 0.5	0.2 × 0.05 × 0.05
Radiation	CuKα (λ = 1.54184)	CuKα (λ = 1.54184)
2Θ range for data collection/°	8.576 to 148.056	7.988 to 148.046
Index ranges	$-7 \le h \le 7$, $-15 \le k \le 15$, $-19 \le l \le 21$	$-8 \le h \le 5$, $-17 \le k \le 17$, $-21 \le l \le 22$
Reflections collected	6446	9395
Independent reflections	2857 [R_{int} = 0.0199, R_{sigma} = 0.0176]	3288 [R_{int} = 0.0623, R_{sigma} = 0.0592]
Data/restraints/parameters	2857/0/235	3288/0/251
Goodness-of-fit on F^2	1.044	1.092
Final R indexes [I>=2σ (I)]	R_1 = 0.0404, wR_2 = 0.1102	R_1 = 0.0663, wR_2 = 0.1863
Final R indexes [all data]	R_1 = 0.0407, wR_2 = 0.1105	R_1 = 0.0815, wR_2 = 0.2017
Largest diff. peak/hole / e Å^{-3}	0.26/-0.22	0.30/-0.41
Flack parameter	0.04(13)	0.0(3)

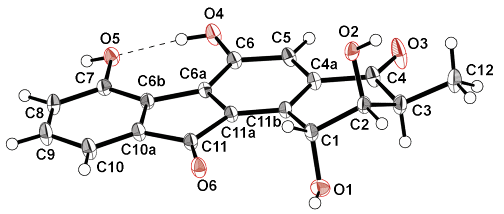

X-ray crystal structure
of **7**

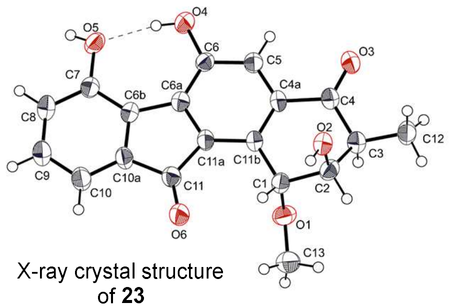

X-ray crystal structure
of **23**

The ellipsoids of non-hydrogen atoms are shown at 50% probability levels for **7** and **23**.

Supplementary Table 4. ^{1}H (700 MHz) and ^{13}C (175 MHz) NMR spectroscopic data of 9, 9-^{18}O and 3-^{18}O.

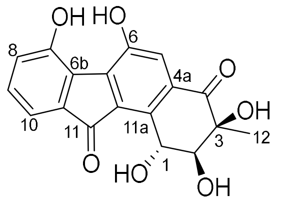

9

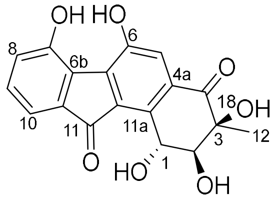

9-^{18}O

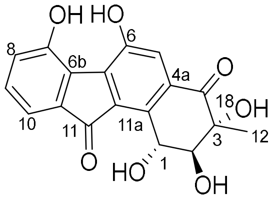

3-^{18}O

Position	**9**[a]		**9**-^{18}O[b]		**3**-^{18}O[c]	
	δ_C, type	δ_H, mult. (J in Hz)	δ_C, type	δ_H, mult. (J in Hz)	δ_C, type	δ_H, mult. (J in Hz)
1	66.3, CH	5.37, d (4.0)	67.1, CH	5.14, d (4.0, 4.0)	67.3, CH	5.11, dd (4.4, 4.4)
2	77.9, CH	3.84, d (4.0)	78.0, CH	3.82, dd (4.0, 4.0)	75.8, CH	3.74, dd (4.5, 4.5)
3	76.7, C		76.4, C		75.2, C	
4	200.2, C		200.3, C		197.6, C	
4a	133.2, C		131.3, C		131.0, C	
5	120.6, CH	7.48, s	121.6, CH	6.87, s	122.1, CH	6.92, s
6	149.9, C		159.3, C		159.5, C	
6a	135.1, C		140.3, C		140.6, C	
6b	125.9, C		129.8, C		129.7, C	
7	151.3, C		160.2, C		160.1, C	
8	124.6, CH	7.11, dd (8.2, 0.9)	126.1, CH	6.53, dd (8.1, 1.1)	126.2, CH	6.54, d (8.3)
9	132.4, CH	7.32, dd (8.2, 7.2)	130.5, CH	6.88, dd (8.1, 7.2)	130.5, CH	6.89, dd (8.3, 7.1)
10	116.9, CH	7.22, dd (7.2, 0.9)	111.3, CH	6.63, dd (7.2, 1.1)	111.4, CH	6.64, dd (7.1)
10a	135.7, C		134.8, C		134.9, C	
11	192.8, C		196.5, C		196.6, C	
11a	133.1, C		131.1, C		131.0, C	
11b	134.4, C		129.1, C		129.2, C	
12	24.0, CH_3	1.33, s	23.8, CH_3	1.29, s	20.8, CH_3	1.20, s
1-OH				5.06, d (4.0)		5.74, d (4.4)
2-OH				5.03, d (4.0)		5.41, d (4.6)
3-OH				4.90, s		5.67, s

[a]Measured in DMSO-d_6-trifluoroacetic acid (TFA)-d_1 (100:1), [b]In DMSO-d_6. Assignments were based on DEPT, HSQC, COSY, HMBC, and NOESY experiments.

Supplementary Table 5. ^{1}H (700 MHz) and ^{13}C (175 MHz) NMR spectroscopic data of 10, 11 and 14 in DMSO-d_6.

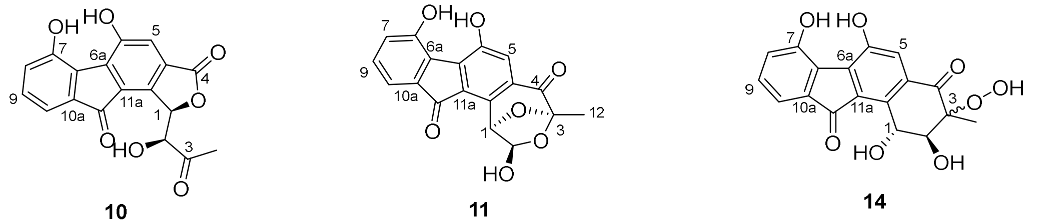

Position	10[a]		11[a]		14[b]	
	δ_C, type	δ_H, mult. (J in Hz)	δ_C, type	δ_H, mult. (J in Hz)	δ_C, type	δ_H, mult. (J in Hz)
1	81.2, CH	5.87, d (2.0)	78.5, CH	5.79, br s	N[c]	5.72, dd (4.4, 4.4)
2	75.5, CH	4.78, dd (6.7, 2.0)	98.0, CH	5.10, d (5.6)	72.0, CH	4.83, dd (7.1, 4.4)
3	210.1, C		106.7, C		87.6, C	
4	170.4, C		189.0, C		195.0, C	
4a	127.9, C		128.6, C		131.0, C	
5	118.6, CH	6.66, s	122.1, CH	6.89, s	N[c]	6.92, s
6	162.0, C		160.4, C		159.3, C	
6a	140.6, C		138.5, C		140.8, C	
6b	130.4, C		130.2, C		129.4, C	
7	159.7, C		159.7, C		160.6, C	
8	126.2, CH	6.59, dd (8.7, 0.7)	125.9, CH	6.58, dd (8.1, 0.5)	126.9, CH	6.56, dd (8.3, 0.7)
9	130.6, CH	6.93, dd (8.2, 7.2)	130.6, CH	6.93, dd (7.9, 7.0)	N[c]	6.91, dd (8.0, 6.9)
10	112.0, CH	6.67, dd (6.9, 0.7)	111.7, CH	6.68, dd (7.2, 0.7)	117.1, CH	6.68, dd (7.2, 0.7)
10a	134.3, C		134.7, C		134.9, C	
11	195.0, C		195.8, C		N[c]	
11a	N[c]		127.5, C		N[c]	
11b	130.6, C		126.3, C		129.4, C	
12	27.1, CH_3	2.24, s	18.2, CH_3	1.57, s	N[c]	1.12, s
2-OH		5.59, d (6.7)		6.97, d (5.4)		

[a] Assignments were based on DEPT, HSQC, COSY, HMBC, and NOESY experiments. [b]Assignments were based on ^{1}H and HMBC experiments. [c]N, no signal was observed.

Supplementary Table 6. Crystal data and structure refinement for 10.

Identification code	**10**
Empirical formula	$C_{18}H_{16}O_9$
Formula weight	376.31
Temperature/K	100.00(10)
Crystal system	triclinic
Space group	P1
a/Å	8.0646(2)
b/Å	9.4581(4)
c/Å	12.3793(4)
α/°	105.647(3)
β/°	91.092(2)
γ/°	110.786(3)
Volume/Å^3	843.13(5)
Z	2
ρ_{calc}g/cm^3	1.482
μ/mm^{-1}	1.035
F(000)	392.0
Crystal size/mm^3	0.02 × 0.02 × 0.01
Radiation	Cu Kα (λ = 1.54184)
2Θ range for data collection/°	7.478 to 148.556
Index ranges	$-10 \leq h \leq 10$, $-11 \leq k \leq 11$, $-15 \leq l \leq 15$
Reflections collected	17982
Independent reflections	6389 [R_{int} = 0.0248, R_{sigma} = 0.0264]
Data/restraints/parameters	6389/3/516
Goodness-of-fit on F^2	1.050
Final R indexes [I>=2σ (I)]	R_1 = 0.0333, wR_2 = 0.0882
Final R indexes [all data]	R_1 = 0.0343, wR_2 = 0.0891
Largest diff. peak/hole / e Å^{-3}	0.40/-0.34
Flack parameter	-0.01(5)

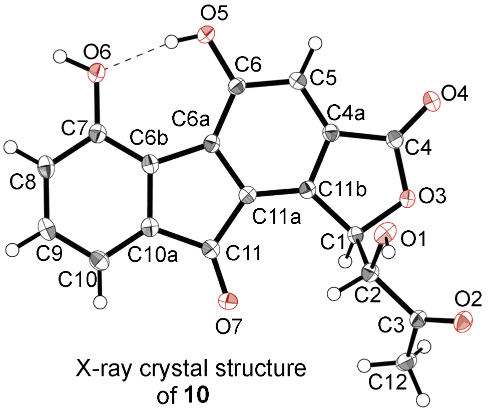

X-ray crystal structure of **10**

The ellipsoids of non-hydrogen atoms are shown at 50% probability levels for **10**.

Supplementary Table 7. ^{1}H (700 MHz) and ^{13}C (175 MHz) NMR spectroscopic data of 22 and 24 in DMSO-*d*$_6$.

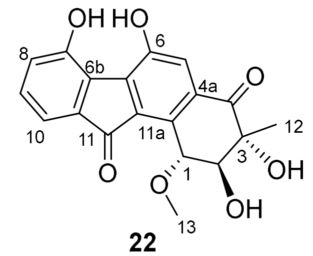

22

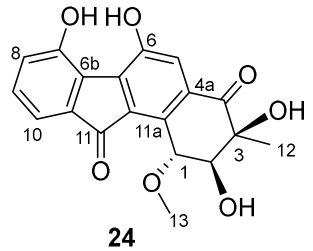

24

Position	22		24	
	δ_C, type	δ_H, mult. (*J* in Hz)	δ_C, type	δ_H, mult. (*J* in Hz)
1	75.5, CH	4.99, d (3.5)	75.8, CH	4.84, d (2.5)
2	71.6, CH	4.00, dd (3.5, 3.4)	72.8, CH	4.02, m
3	75.0, C		76.3, CH	
4	196.7, C		200.8, C	
4a	131.6, C		134.9, C	
5	121.8, CH	6.91, s	121.1, CH	6.85, s
6	160.3, C		159.7, C	
6a	140.1, C		140.9, C	
6b	129.6, C		129.6, C	
7	159.7, C		158.7, C	
8	125.8, C	6.54, d (8.2)	125.7, CH	6.53, d (8.1)
9	130.4, CH	6.89, dd (8.2, 7.0)	130.4, CH	6.89, dd (8.1, 6.7)
10	111.3, CH	6.65, d (7.0)	111.1, CH	6.63, d (6.7)
10a	135.0, C		135.2, C	
11	195.7, C		195.5, C	
11a	131.4, C		131.6, C	
11b	124.8, C		125.3, C	
12	21.3, CH_3	1.23, s	24.4, CH_3	1.26, s
13	58.3, CH_3	3.56, s	57.8, CH_3	3.54, s
2-OH		5.49, br d (4.4)		5.12, br s
3-OH		5.04, s		4.90, s

Assignments were based on DEPT, HSQC, COSY, HMBC, and NOESY experiments.

Supplementary Table 8. ^{1}H (700 MHz) and ^{13}C (175 MHz) NMR spectroscopic data of 23 and 25 in DMSO-d_6.

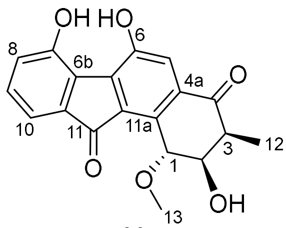

23

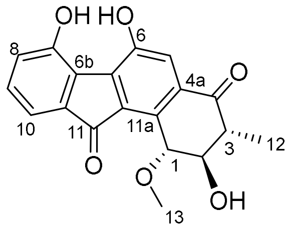

25

Position	23		25	
	δ_C, type	δ_H, mult. (J in Hz)	δ_C, type	δ_H, mult. (J in Hz)
1	72.3, CH	5.08, d (3.4)	74.2, CH	4.98, br d (3.1)
2	71.2, CH	4.22, br s	69.9, CH	4.15, dd (3.1, 2.6)
3	42.4, CH	3.00, m	48.2, CH	2.50, overlapping
4	198.2, C		199.8, C	
4a	133.9, C		131.6, C	
5	120.8, CH	7.19, s	121.3, CH	6.88, s
6	155.1, C		159.6, C	
6a	135.4, C		139.8, C	
6b	128.0, C		129.7, C	
7	155.9, C		160.1, C	
8	125.3, CH	6.81, d (8.1)	125.8, CH	6.52, dd (6.5, 0.6)
9	131.2, CH	7.09, dd (8.1, 6.9)	130.3, CH	6.88, dd (7.0, 6.5)
10	113.8, CH	6.90, d (6.9)	110.9, CH	6.62, dd (7.0, 0.6)
10a	136.8, C		135.1, C	
11	194.8, C		195.9, C	
11a	132.5, C		131.8, C	
11b	128.7, C		126.4, C	
12	12.0, CH_3	1.16, d (6.9)	16.5, CH_3	1.21, d (7.9)
13	57.9, CH_3	3.48, s	57.4, CH_3	3.42, s

Assignments were based on DEPT, HSQC, COSY, HMBC, and NOESY experiments.

Supplementary Table 9. ^{1}H (700 MHz) and ^{13}C (175 MHz) NMR spectroscopic data of 26 and 27 in DMSO-d_6.

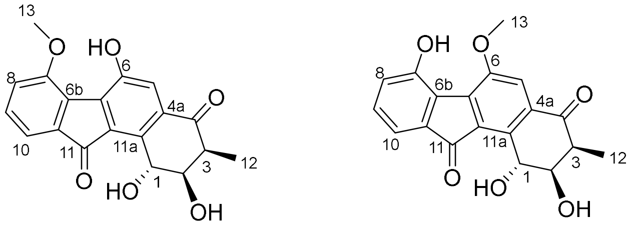

26 **27**

Position	26		27	
	δ_C, type	δ_H, mult. (J in Hz)	δ_C, type	δ_H, mult. (J in Hz)
1	64.1, CH	5.47, d (3.3)	64.1, CH	5.50, d (2.8)
2	75.3, CH	4.01, br s	75.2, CH	4.04, dd (2.8, 2.3)
3	42.1, CH	3.19, m	42.1, CH	3.20, dq (6.7, 1.9)
4	198.2, C		198.0, C	
4a	134.5, C		134.5, C	
5	121.0, CH	7.40, s	115.8, CH	7.69, s
6	150.9, C		151.3, C	
6a	132.9, C		136.3, C	
6b	128.4, C		124.5, C	
7	152.3, C		152.1, C	
8	120.3, CH	7.46, overlapping	125.3, CH	7.09, d (8.1)
9	132.3, CH	7.48, overlapping	132.8, CH	7.35, dd (8.1, 6.8)
10	118.2, CH	7.36, d (6.3)	116.8, CH	7.21, d (6.8)
10a	135.8, C		135.9, C	
11	192.6, C		192.5, C	
11a	132.1, C		132.9, C	
11b	135.2, C		136.2, C	
12	11.8, CH_3	1.17, d (7.1)	11.9, CH_3	1.18, d (7.3)
6-OMe			58.0, CH_3	4.14, s
7-OMe	58.0, CH_3	4.12, s		
1-OH		5.42, s		
2-OH		5.35, s		

Assignments were based on DEPT, HSQC, COSY, HMBC, and NOESY experiments.

Supplementary Table 10. ^{1}H (700 MHz) and ^{13}C (175 MHz) NMR spectroscopic data of 38 in methanol-d_4.

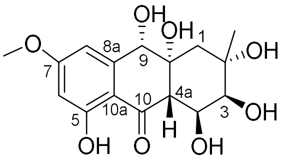

38

Position	38	
	δ_C, type	δ_H, mult.(J in Hz)
1	38.1, CH_2	2.02, d (14.5)
		1.92, d (14.5)
2	72.8, C	
3	75.2, CH	3.65, br s
4	66.6, CH	4.58, d (3.0)
4a	52.1, CH	2.93, d (10.0)
5	164.4, C	
6	99.0, CH	6.34, d (2.7)
7	166.9, C	
8	105.3, CH	6.79, m
8a	147.4, C	
9	73.7, CH	
9a	77.7, C	
10	204.9, C	
10a	110.1, C	
11	54.7, CH_3	3.85, s
12	26.0, CH_3	1.31, s

Assignments were based on DEPT, HSQC, COSY, HMBC, and NOESY experiments.

Supplementary Table 11. ^{1}H (700 MHz) and ^{13}C (175 MHz) NMR spectroscopic data for 39, 40 and 41 in DMSO-*d*$_6$.

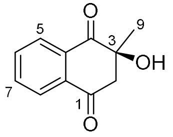

39

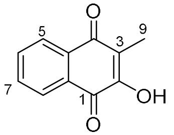

40

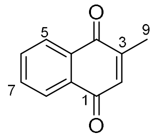

41

Position	39		40		41	
	δ_C, type	δ_H, mult. (*J* in Hz)	δ_C, type	δ_H, mult. (*J* in Hz)	δ_C, type	δ_H, mult. (*J* in Hz)
1	195.5, C		184.1, C		183.0, C	
2	52.3, CH_2	3.05, d (16.5)	158.9, C		133.6, C	6.98, m
		3.26, d (16.5)				
3	74.8, C		119.1, C		146.4, C	
4	197.4, C		182.3, C		183.4, C	
4a	134.1, C		130.7, C		130.2, C	
5	127.5, CH	7.99, dd (7.1, 1.6)	132.9, CH	7.95, dd (7.7, 1.1)	124.4, C	8.02, m
6	134.5, CH	7.87, ddd (7.6, 7.1, 1.6)	125.9, CH	7.73, dd (7.7, 7.3)	132.3, C	7.86, m, overlapping
7	134.9, CH	7.85, ddd (7.6, 7.1, 1.6)	125.9, CH	7.79, ddd (7.3, 7.2, 1.1)	132.4, C	7.86, m, overlapping
8	126.1, CH	7.90, dd (7.1, 1.6)	134.6, CH	7.94, d (7.2)	123.9, C	7.97, m
8a	135.3, C		133.3, C		130.1, C	
9	24.5, CH_3	1.38, s	9.2, CH_3	1.91, s	14.3, CH_3	2.12, s

Assignments were based on DEPT, HSQC, COSY, HMBC, and NOESY experiments.

Supplementary Table 12. Crystal data and structure refinement for 40.

Identification code	**40**
Empirical formula	$C_{11}H_8O_3$
Formula weight	188.17
Temperature/K	100.01(12)
Crystal system	monoclinic
Space group	$P2_1$
a/Å	7.6523(5)
b/Å	4.7776(3)
c/Å	11.6829(9)
α/°	90
β/°	90.544(8)
γ/°	90
Volume/Å^3	427.10(5)
Z	2
ρ_{calc}g/cm^3	1.463
μ/mm^{-1}	0.893
F(000)	196.0
Crystal size/mm^3	0.05 × 0.01 × 0.01
Radiation	CuKα (λ = 1.54184)
2Θ range for data collection/°	7.568 to 148.544
Index ranges	$-9 \le h \le 9, -3 \le k \le 5, -14 \le l \le 8$
Reflections collected	1758
Independent reflections	1084 [R_{int} = 0.0445, R_{sigma} = 0.0497]
Data/restraints/parameters	1084/1/129
Goodness-of-fit on F^2	1.025
Final R indexes [I>=2σ (I)]	R_1 = 0.0743, wR_2 = 0.1797
Final R indexes [all data]	R_1 = 0.0779, wR_2 = 0.1832
Largest diff. peak/hole / e Å^{-3}	0.56/-0.34
Flack parameter	0.6(5)

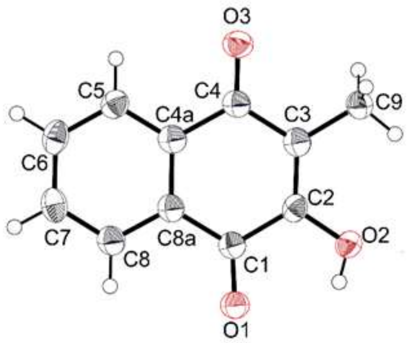

X-ray crystal structure of **40**

The ellipsoids of non-hydrogen atoms are shown at 50% probability levels for **40**.

Supplementary Table 13. ^{1}H (700 MHz) and ^{13}C (175 MHz) NMR spectroscopic data of 47 in DMSO-d_6.

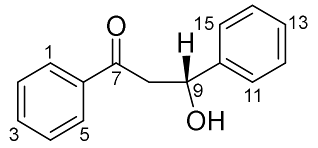

47

Position	47	
	δ_C, type	δ_H, mult.(J in Hz)
1/5	128.6, CH	7.98, dd (7.3, 1.3)
2/4	133.5, C	7.64, tt (7.3, 1.3)
3	129.1, C	7.53, t (7.3)
6	137.6, C	
7	198.8, CH	
8	48.4, CH_2	3.19, dd (15.8, 4.4)
		3.43, dd (15.8, 8.5)
9	69.8, CH	5.14, m
10	145.9, C	
11/15	126.4, C	7.42, d (6.8)
12/14	128.5, C	7.34, t (6.8)
13	127.4, C	7.25, tt (6.8, 1.2)
9-OH		5.38, br d (4.3)

Assignments were based on DEPT, HSQC, COSY, HMBC, and NOESY experiments.

Supplementary Table 14. Strains and plasmids used in this study.

**Strains/plasmids**	**Description**	**Reference/sources**
E. coli		
DH5α	Host strain for cloning	Invitrogen
BL21 (DE3)	Host strain for protein expression	Novagen
Rosetta (DE3)	Host strain for protein expression	Novagen
**Actinomycete**		
Micromonospora rosaria SCSIO N160	Native producer of FSTs	[1]
**Plasmids**		
pET28a	Kanamycin (Kmr), expression vector	Novagen
pCSG118	0.7 kb *fre* *Nde*I/*Bam*HI PCR fragment from genomic DNA of *E. coli* BL21(DE3) into pET28a	[4]
pCSG5102	1467 bp *flsO2* *Nde*I/*Bam*HI PCR fragment from genomic DNA of *M. rosaria* SCSIO N160 into pET28a	[5]
pCSG5213	A 780 bp *Nde*I/*Bam*HI fragment of *flsH* by PCR from genomic DNA of *M. rosaria* SCSIO N160 inserted into pET28a	[5]
pCSG5225	A 957 bp *alp1U* fragment was synthesized and digested with *Nde*I/*Eco*RI and then was inserted into pET28a	[5]
pCSG2607	1.8 kb *xiaK* PCR fragment (*Nde*I/*Bgl*II) from SCSIO 02999 inserted into pET28a (*Nde*I/*Bam*HI)	[6]
pCSG99	1.56 kb *Nde*I/*Eco*RI *tiaM* PCR fragment from genomic DNA of *Dactylosporangium aurantiacum* subsp. *hamdenensis* NRRL 18085 into pET28a	[7]
pCSG8112	A 1083 bp *rslO5* fragment was synthesized and digested with *Nde*I/*Bam*HI and then was inserted into pET28a	This study
pRSF-BmGDH	Expression vector for D-glucose dehydrogenase from *Bacillus megaterium* (BmGDH)	[8]
pCSG3622	1.06 kb *Nde*I/*Bam*HI PCR fragment encoding TaGDH from genomic DNA of *Thermoplasma acidophilum* in pET28a	[8]

Supplementary Figures

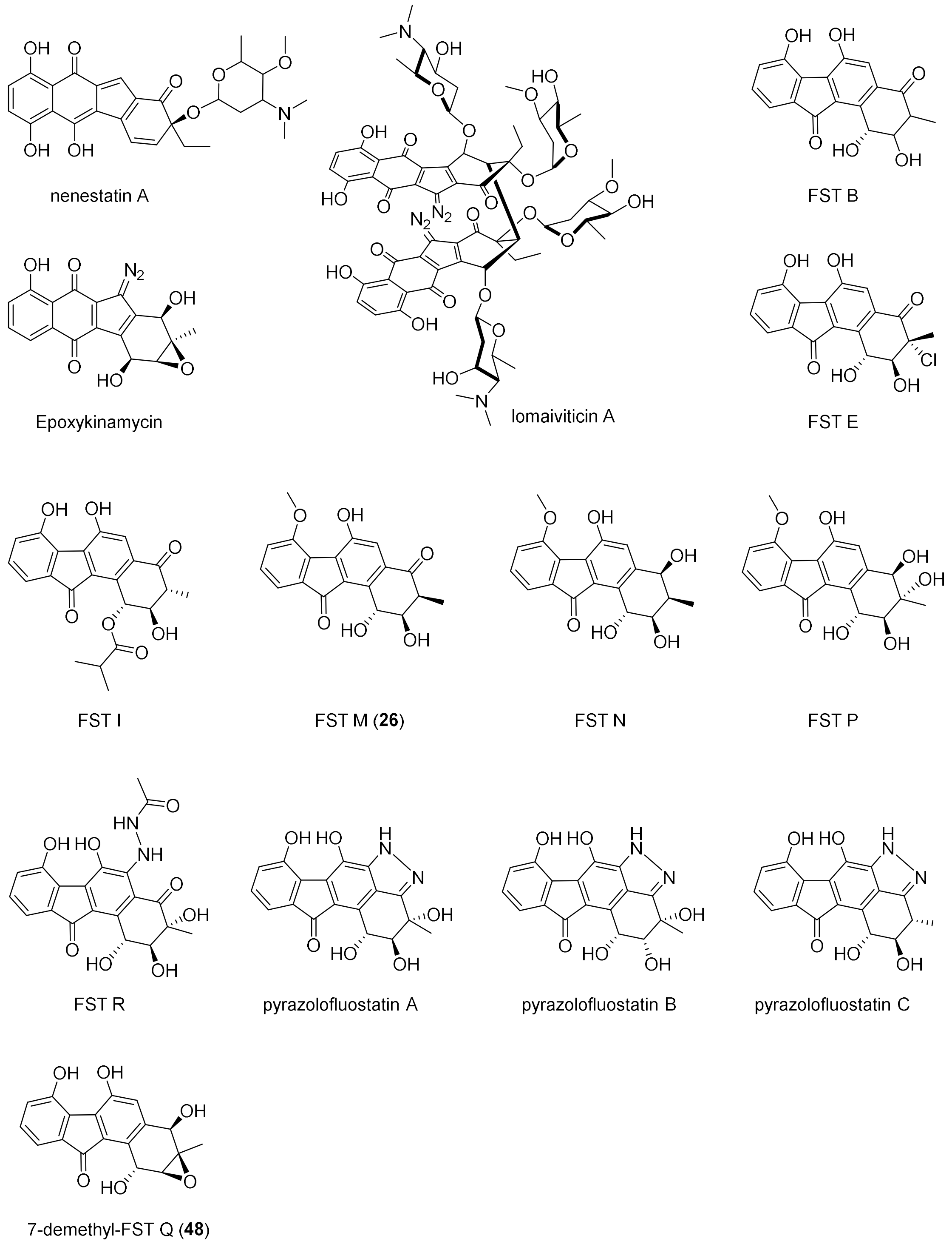

Supplementary Fig. 1. Naturally-occurring atypical angucyclines and FSTs proposed to be derived from epoxide precursors.

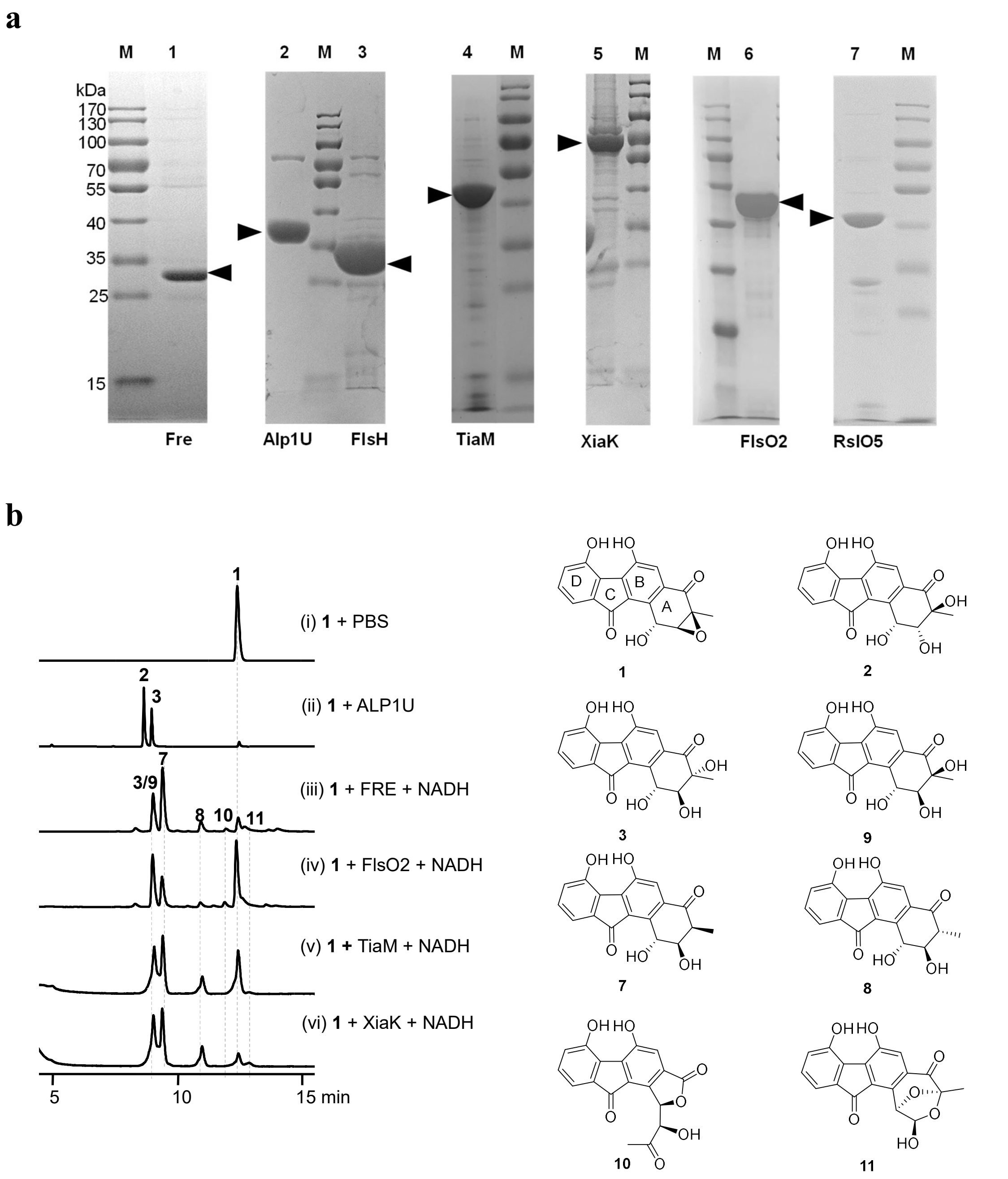

Supplementary Fig. 2. SDS-PAGE analysis of recombinant enzymes and HPLC analysis of their reactions with FST C (1).

(**a**) SDS-PAGE analysis of purified recombinant enzymes. The expected sizes of the recombinant proteins are 30 kDa for Fre (lane 1), 36.3 kDa for Alp1U (lane 2), 30 kDa for FlsH (lane 3), 55.6 kDa for TiaM (lane 4), 65.4 kDa for XiaK (lane 5), 51 kDa for FlsO2 (lane 6), and 38.4 kDa for RslO5 (lane7); protein molecular weight standard (lane M). (**b**) HPLC analysis of reactions of 10 μM Alp1U (or Fre, FlsO2, TiaM, XiaK), 100 μM **1**, and 10 mM NADH in 50 mM PBS buffer (pH 7) for 2 h at 30 ºC.

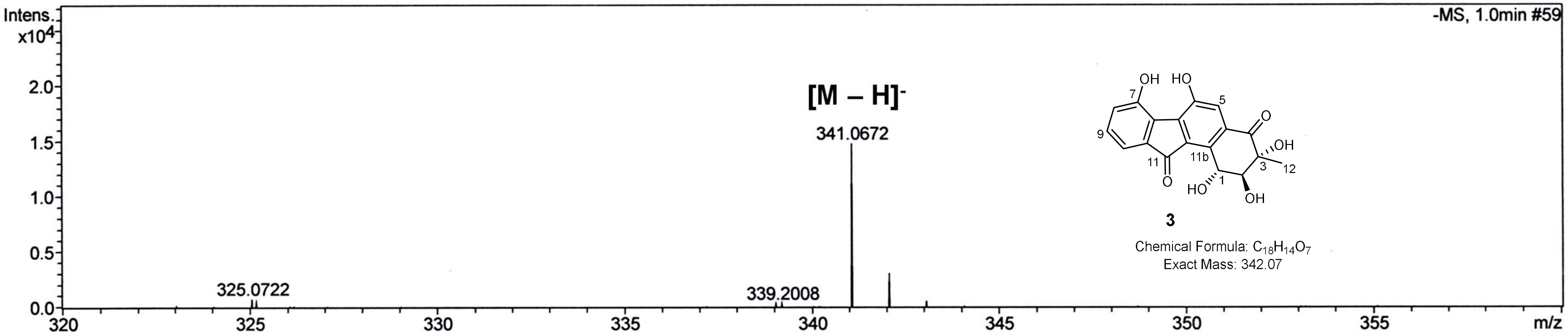

Meas. m/z	#	Ion Formula	Score	m/z	err [ppm]	err [mDa]	mSigma	rdb	e⁻ Conf	N-Rule
341.0672	1	C18H13O7	100.00	341.0667	1.6	0.5	6.3	12.5	even	ok
683.1410	1	C36H27O14	100.00	683.1406	0.5	0.4	18.1	23.5	even	ok

Supplementary Fig. 3. Spectroscopic data for FST C2 (3). (**a**) HRESIMS spectrum.

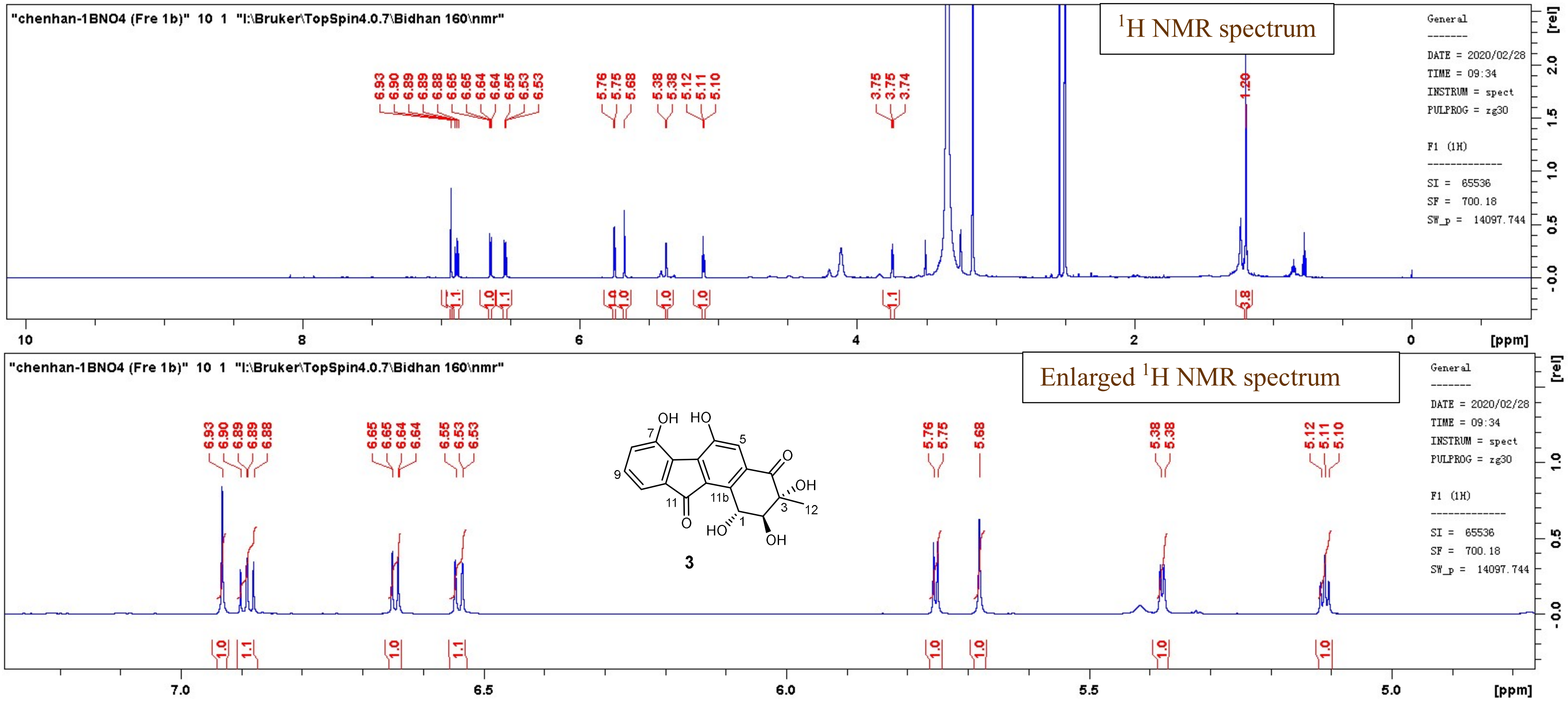

Supplementary Fig. 3. Spectroscopic data for FST C2 (3). (**b**) The ^{1}H and enlarged ^{1}H NMR spectrum of **3** in DMSO-d_6.

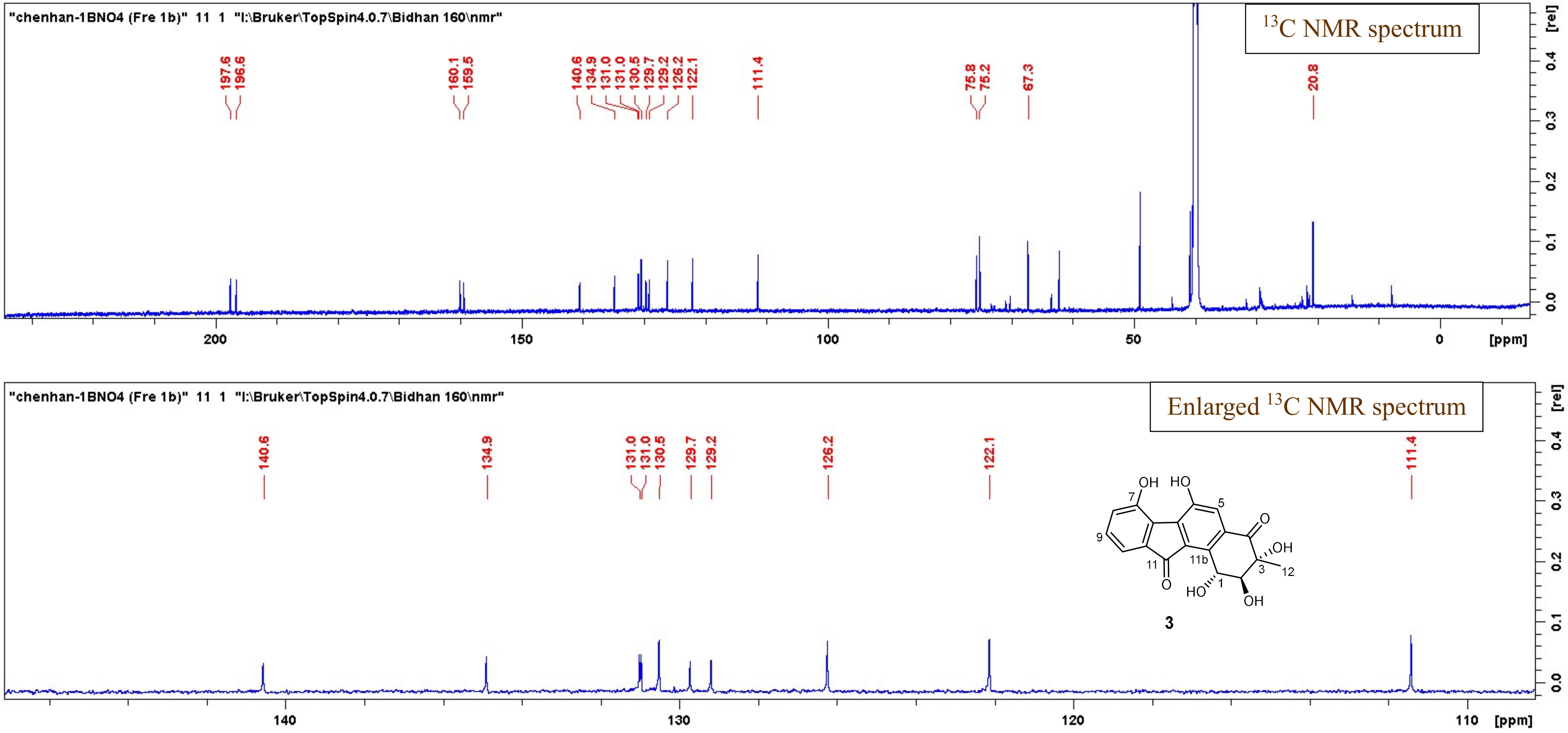

Supplementary Fig. 3. **Spectroscopic data for FST C2 (3).** (**c**) The ^{13}C and enlarged ^{13}C NMR spectrum of **3** in DMSO-d_6.

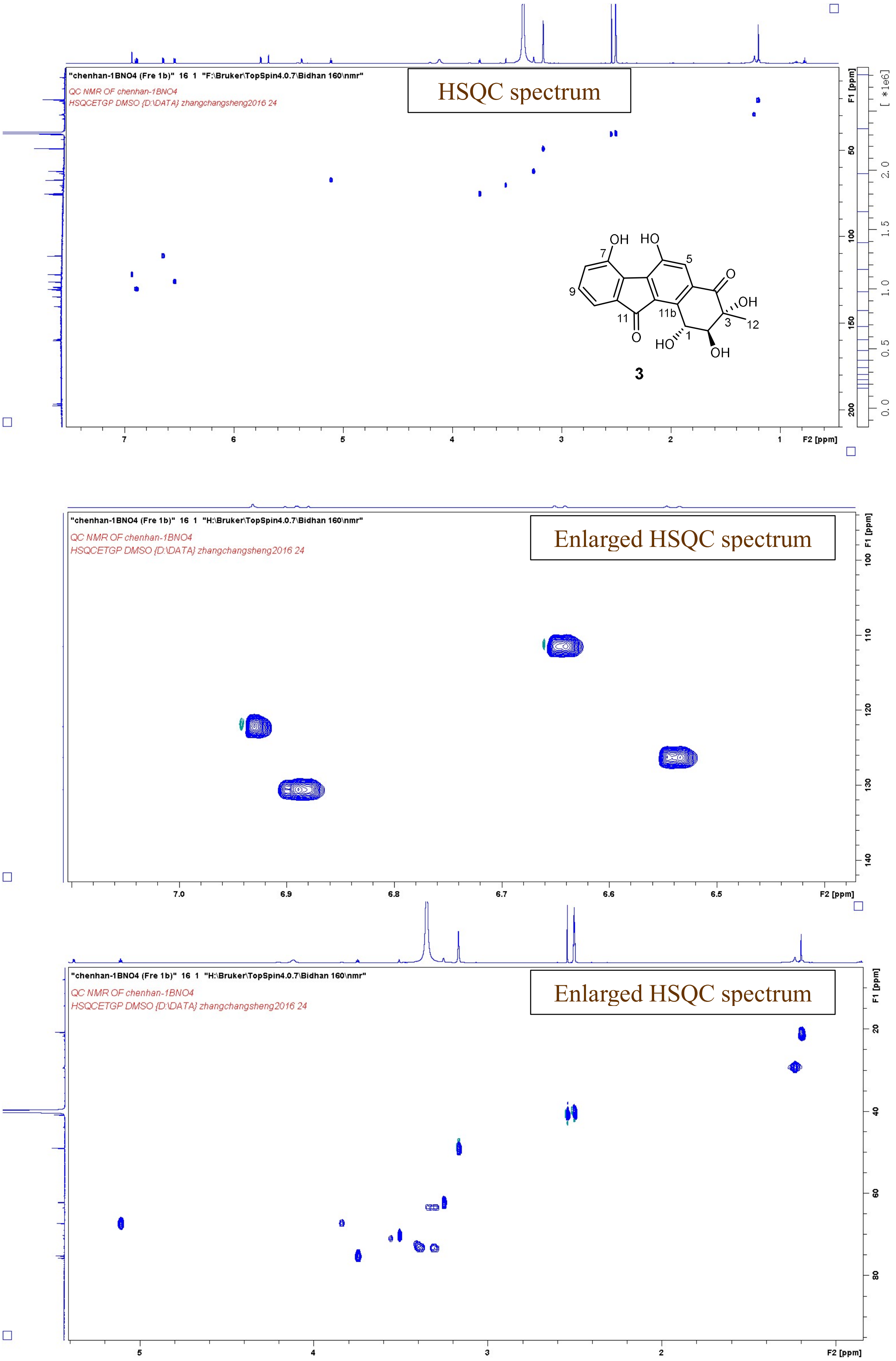

Supplementary Fig. 3. Spectroscopic data for FST C2 (3). (**d**) The HSQC and enlarged HSQC spectrum of **3** in DMSO-d_6.

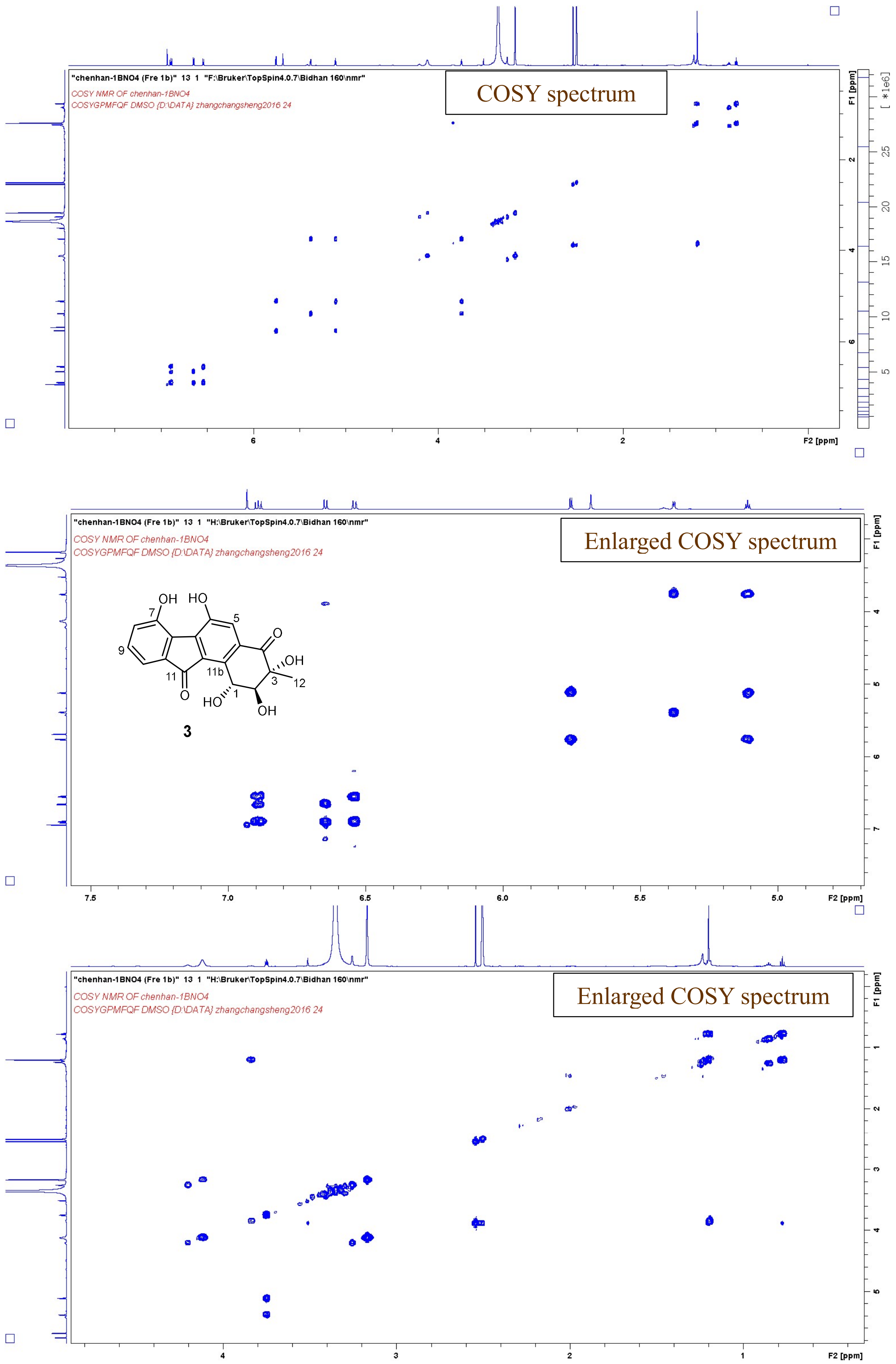

Supplementary Fig. 3. **Spectroscopic data for FST C2 (3).** (**e**) The COSY and enlarged COSY spectrum of **3** in DMSO-d_6.

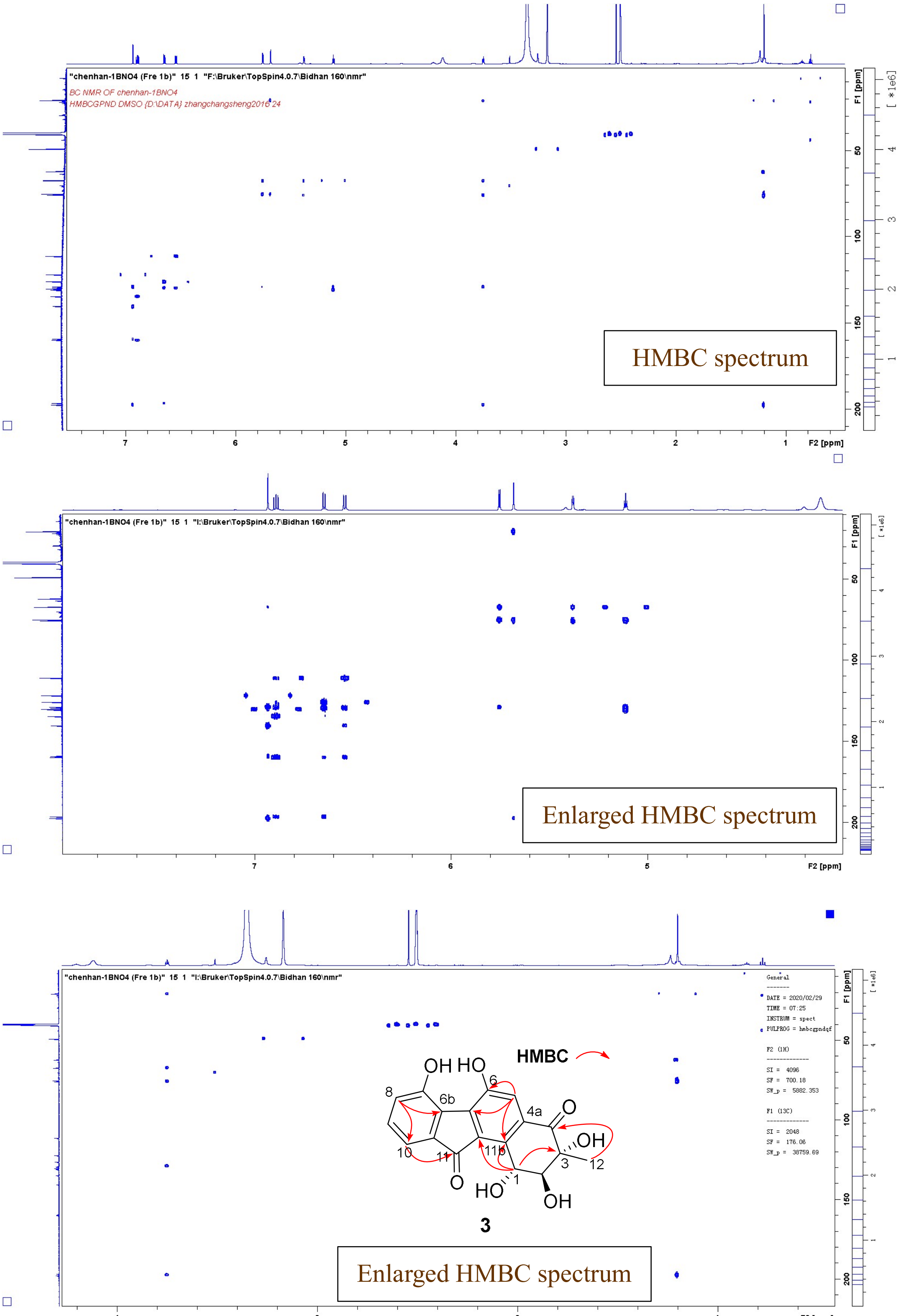

Supplementary Fig. 3. **Spectroscopic data for FST C2 (3).** (**f**) The HMBC and enlarged HMBC spectrum of **3** in DMSO-d_6.

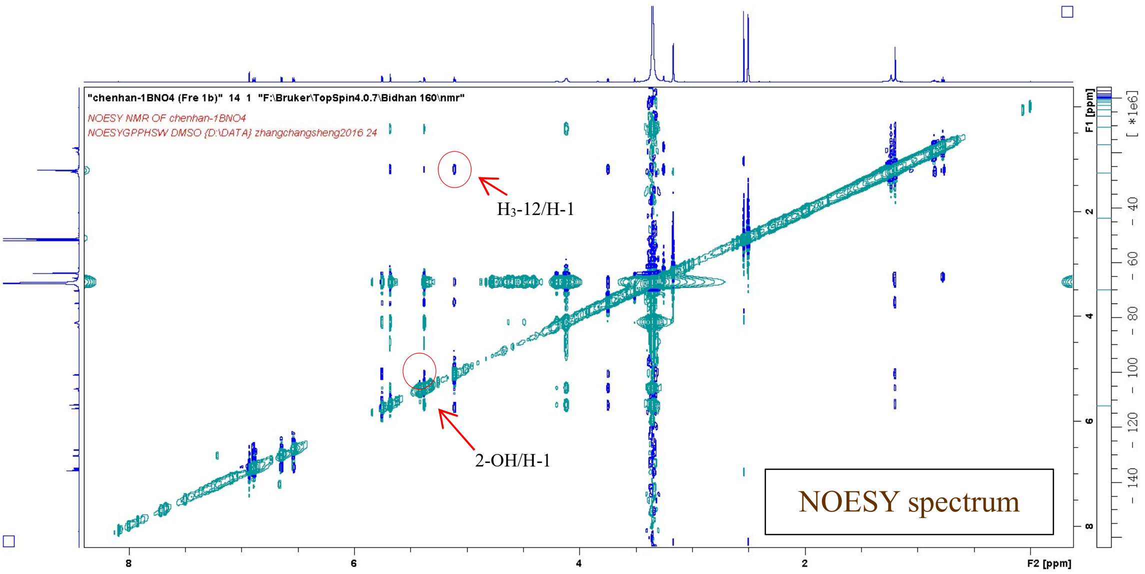

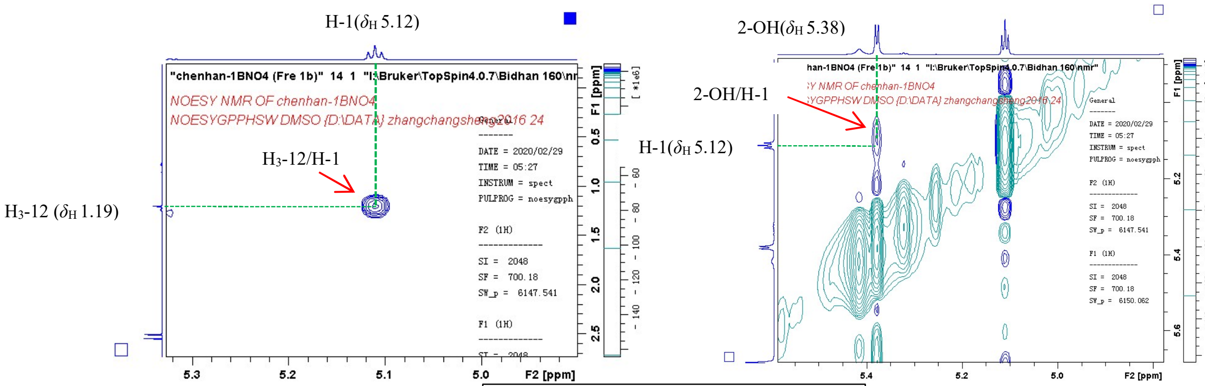

Enlarged NOESY spectrum

Supplementary Fig. 3. **Spectroscopic data for FST C2 (3).** (g) The NOESY and enlarged NOESY spectrum of **3** in DMSO-d_6.

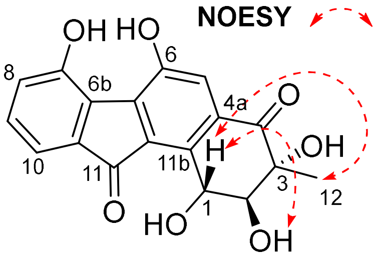

3

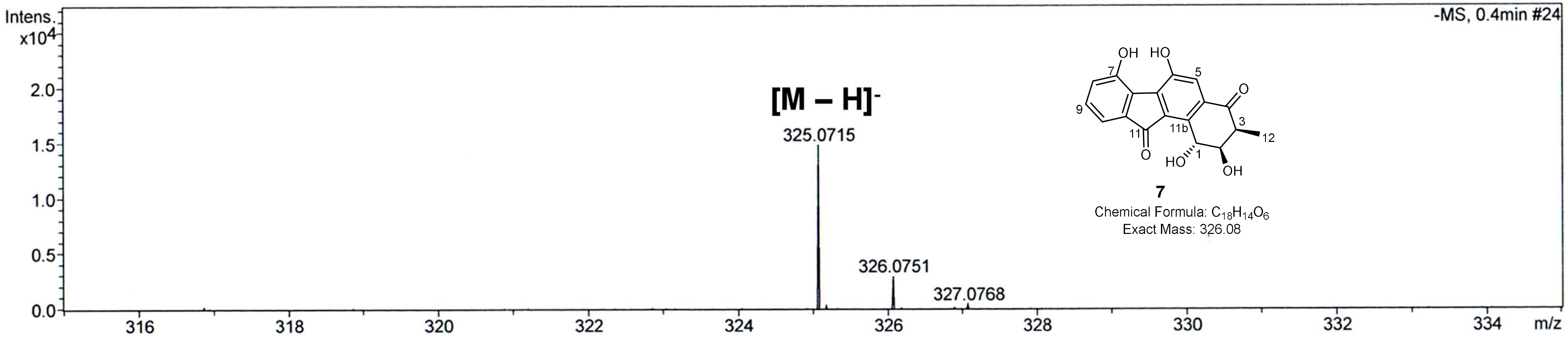

Meas. m/z	#	Ion Formula	Score	m/z	err [ppm]	err [mDa]	mSigma	rdb	e¯ Conf	N-Rule
325.0715	1	C18H13O6	100.00	325.0718	0.9	0.3	1.8	12.5	even	ok
651.1508	1	C36H27O12	100.00	651.1508	0.1	0.0	24.8	23.5	even	ok

Supplementary Fig. 4. Spectroscopic data for FST B1 (7). (**a**) HRESIMS spectrum.

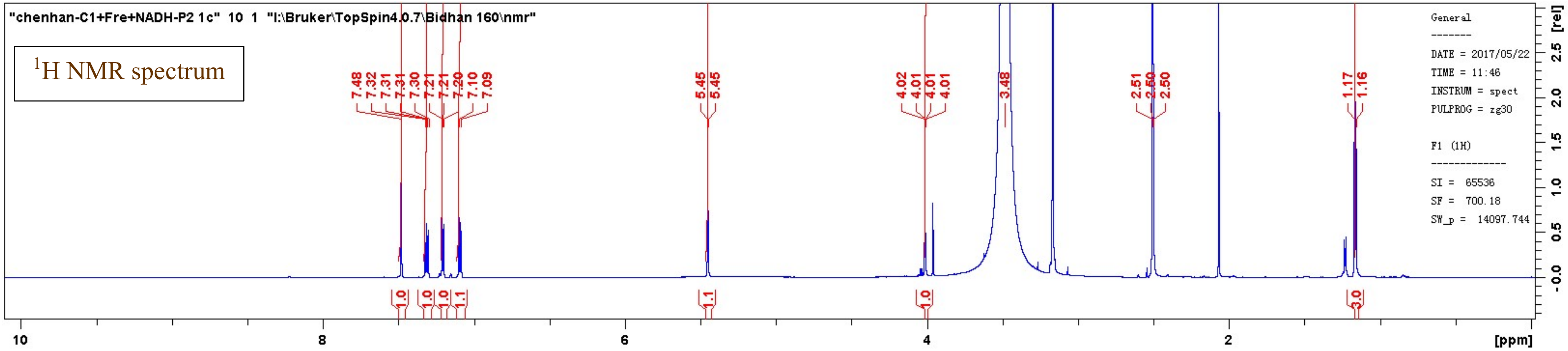

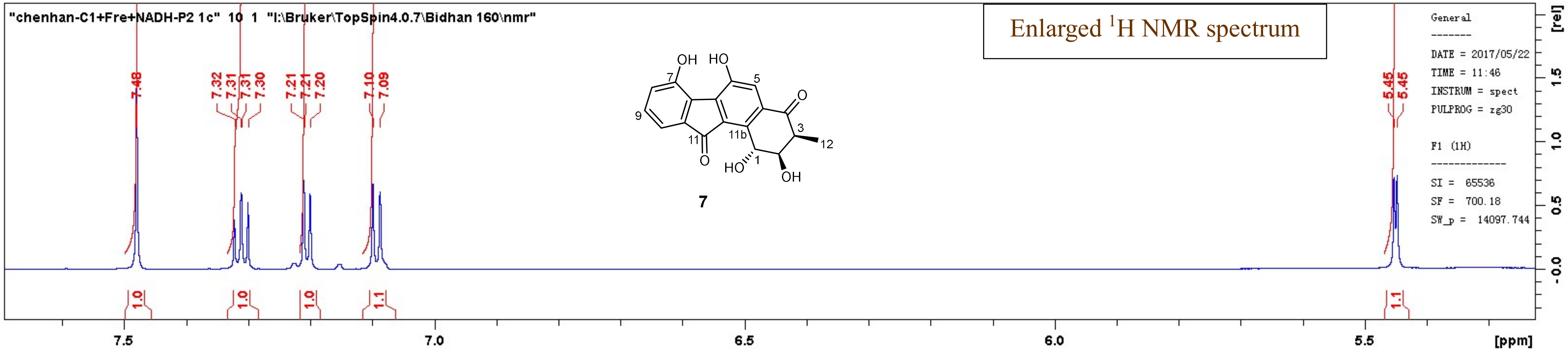

Supplementary Fig. 4. **Spectroscopic data for FST B1 (7).** (**b**) The ^{1}H and enlarged ^{1}H NMR spectrum of **7** in DMSO-d_6.

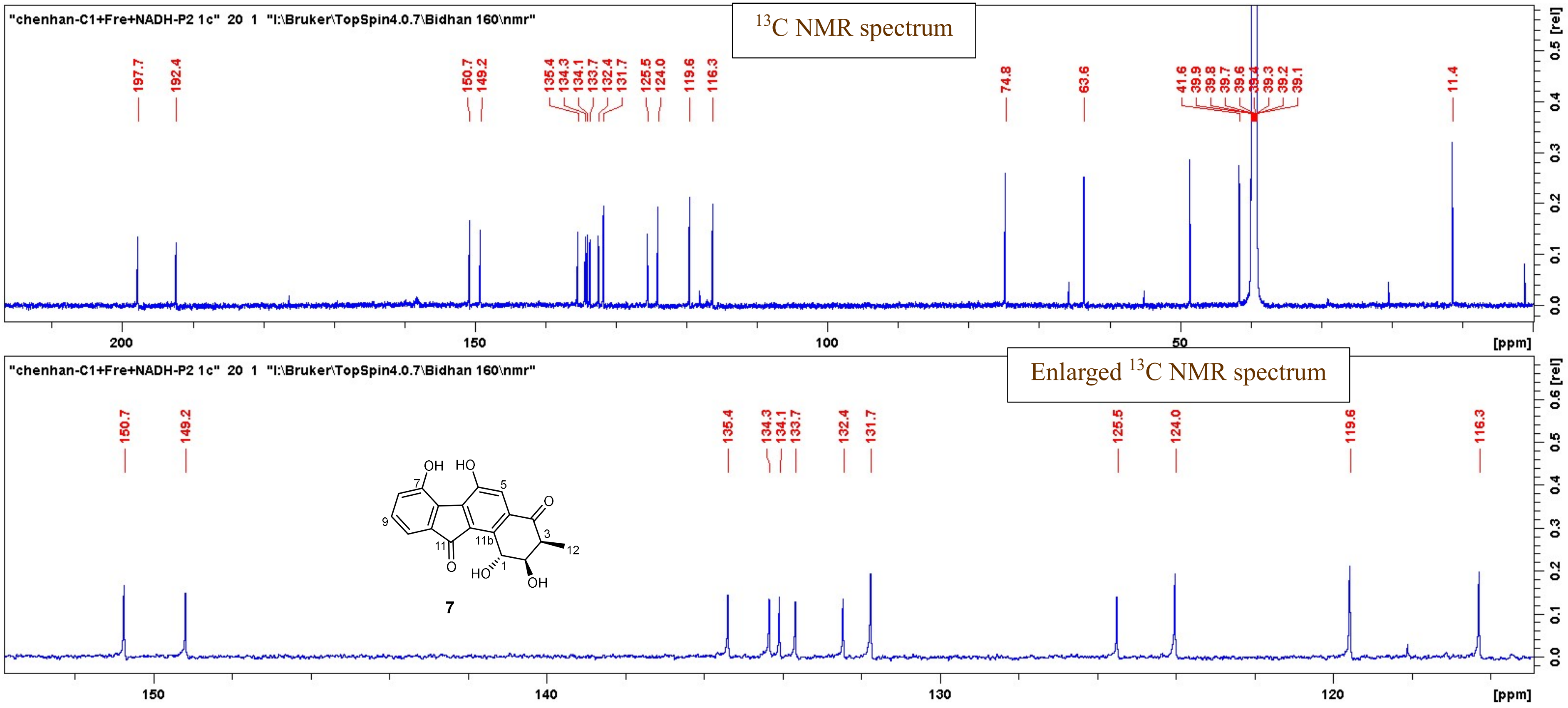

Supplementary Fig. 4. **Spectroscopic data for FST B1 (7).** (**c**) The ^{13}C and enlarged ^{13}C NMR spectrum of **7** in DMSO-d_6.

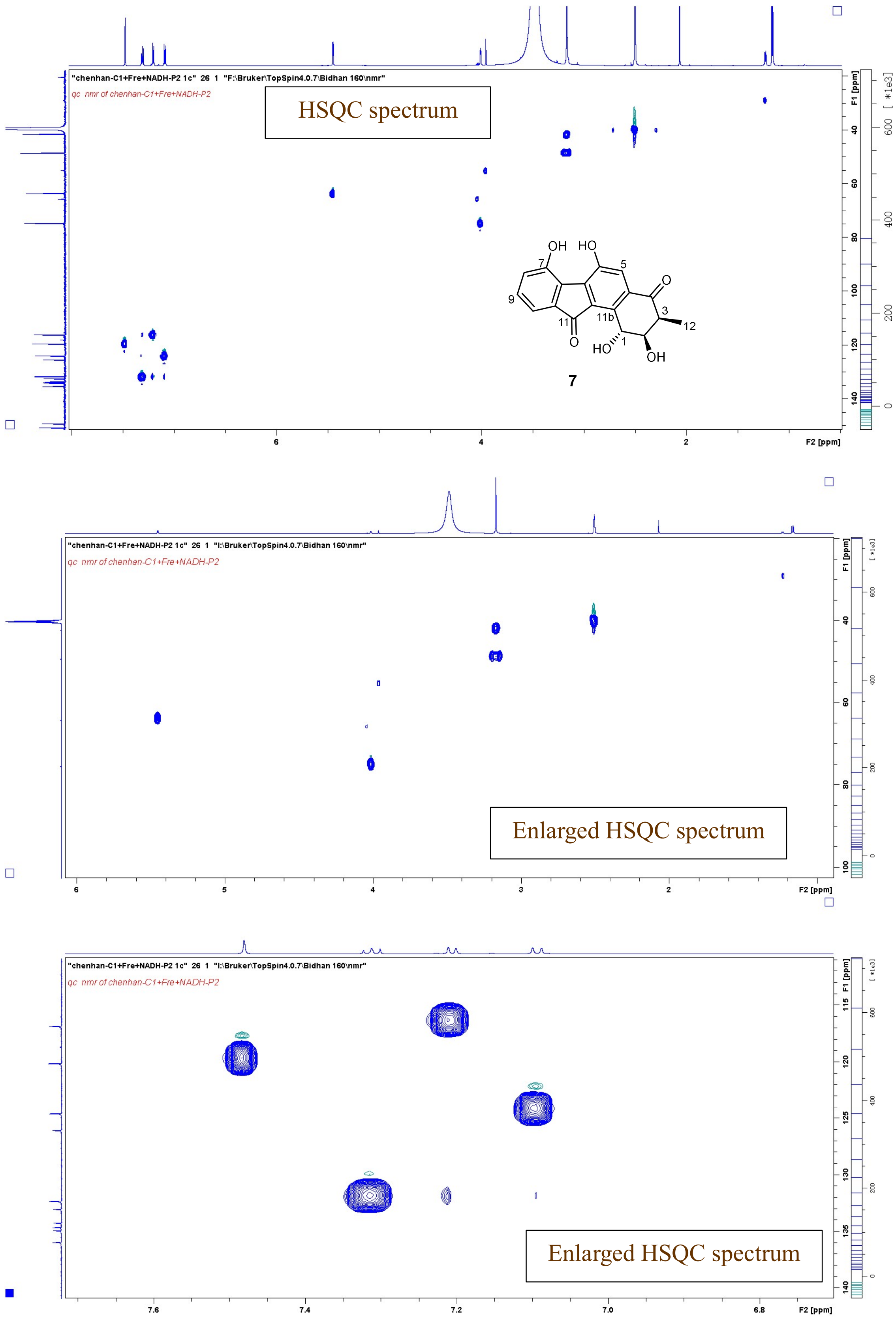

Supplementary Fig. 4. **Spectroscopic data for FST B1 (7).** (**d**) The HSQC and enlarged HSQC spectrum of **7** in DMSO-d_6.

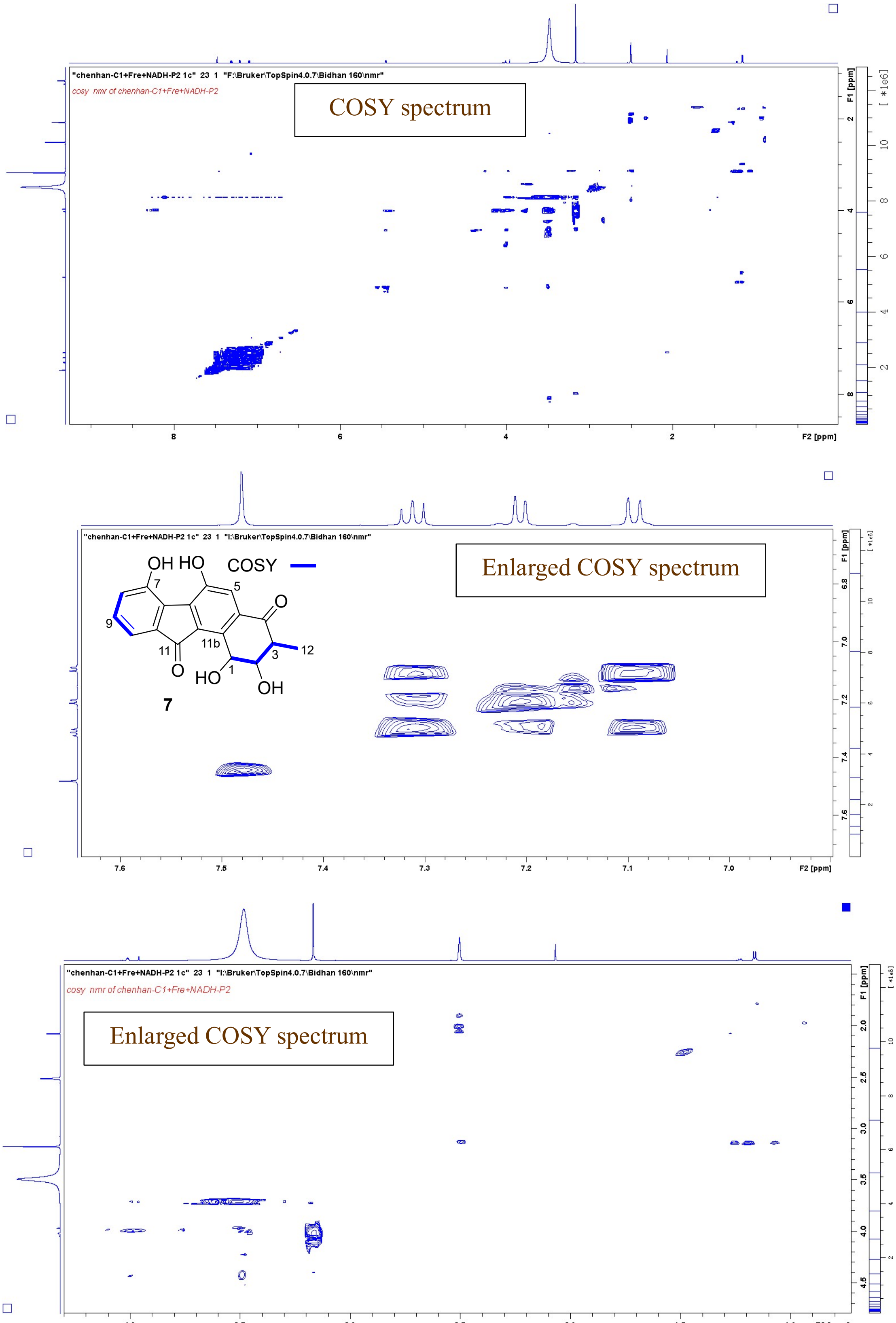

Supplementary Fig. 4. **Spectroscopic data for FST B1 (7).** (**e**) The COSY and enlarged COSY spectrum of **7** in DMSO-d_6

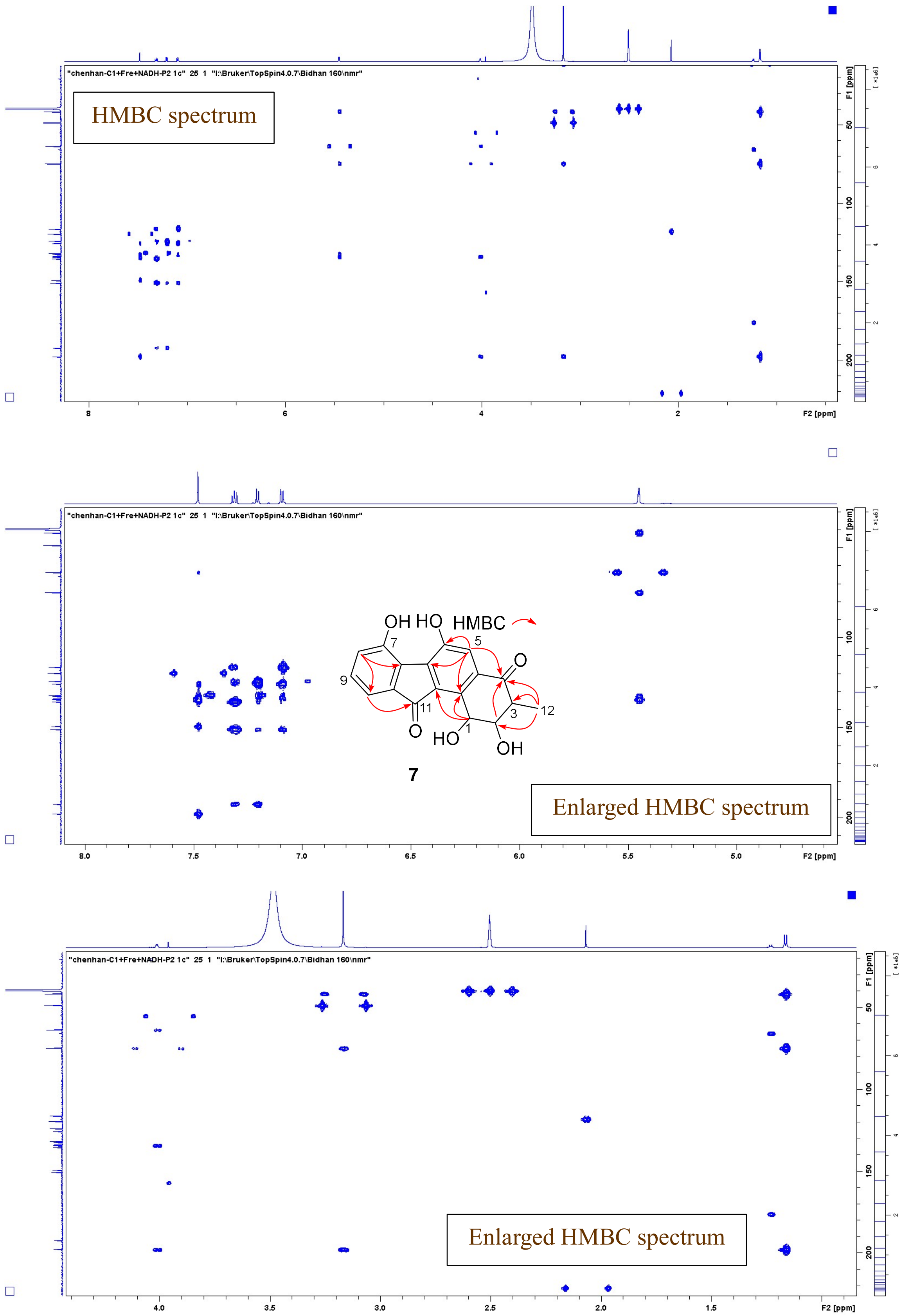

Supplementary Fig. 4. **Spectroscopic data for FST B1 (7).** (**f**) The HMBC and enlarged spectrum of **7** in DMSO-d_6.

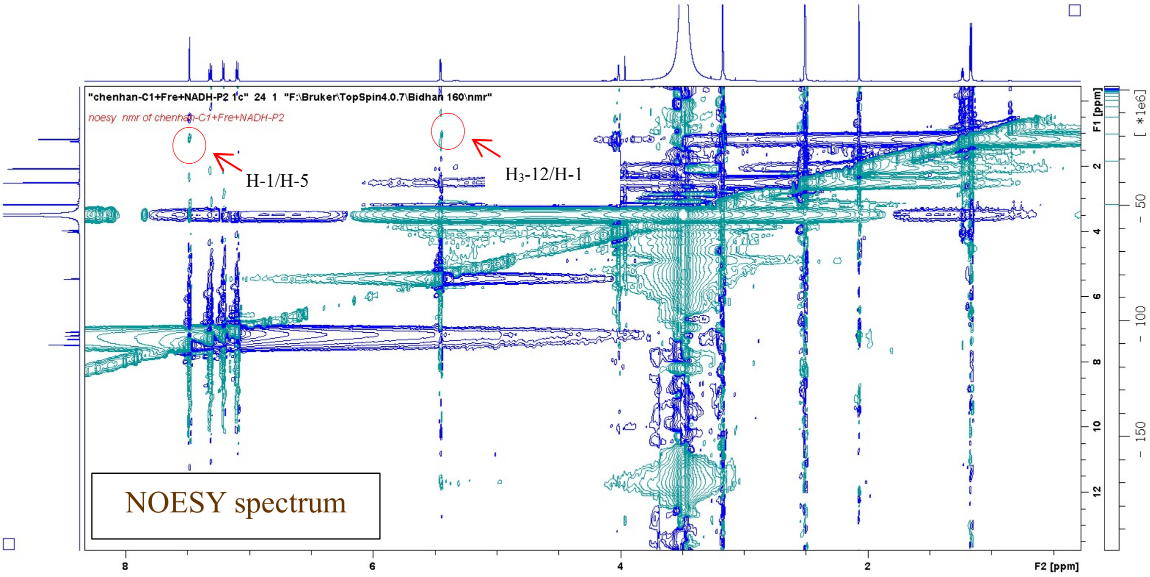

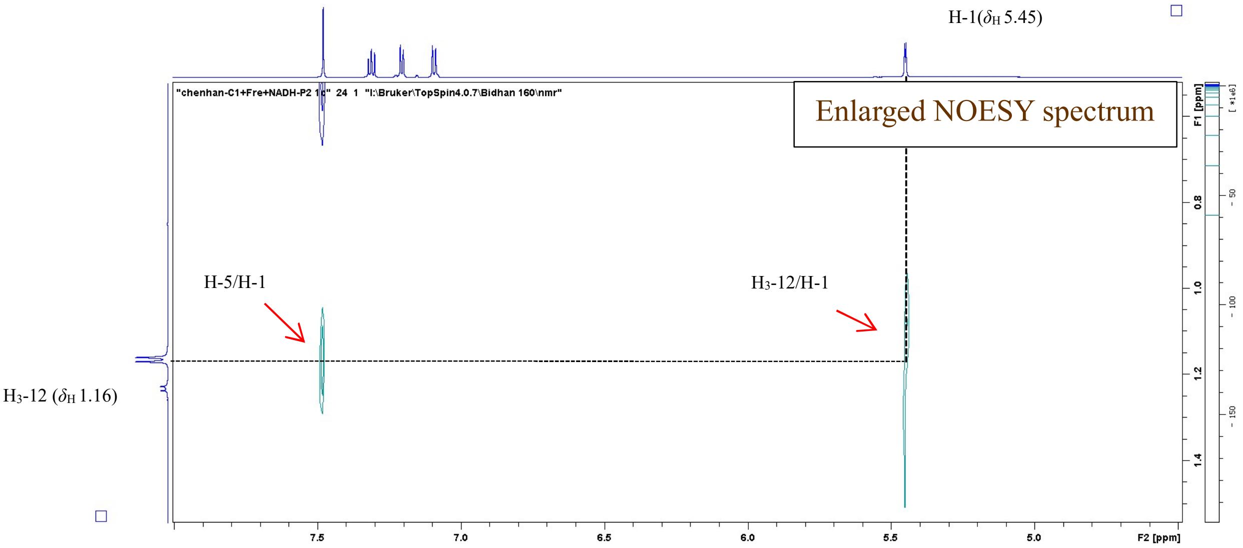

Supplementary Fig. 4. **Spectroscopic data for FST B1 (7).** (g) The NOESY and enlarged NOESY spectrum of **7** in DMSO-d_6.

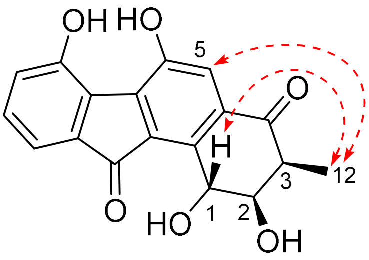

7

a

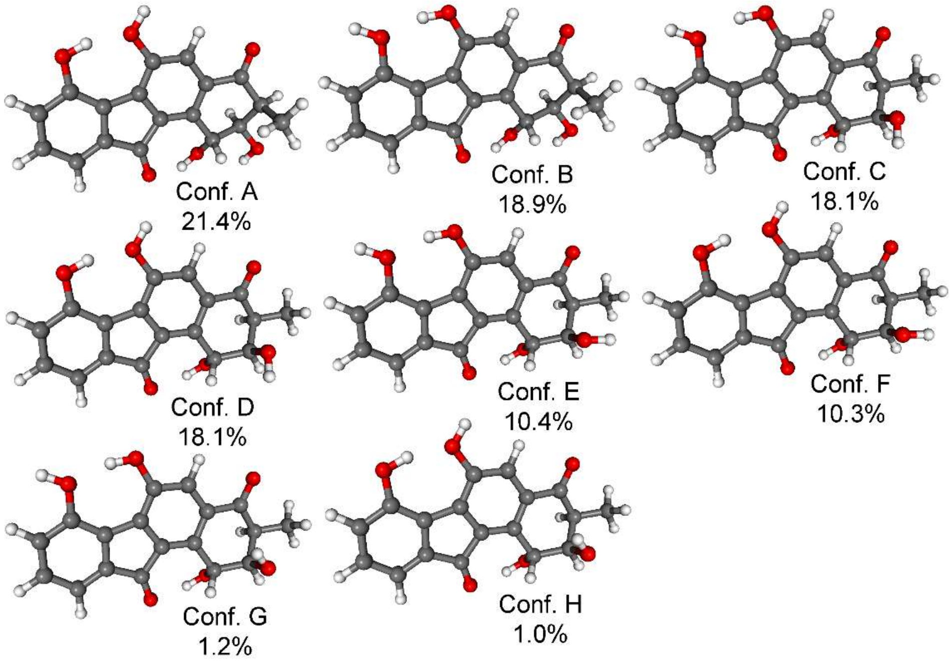

b

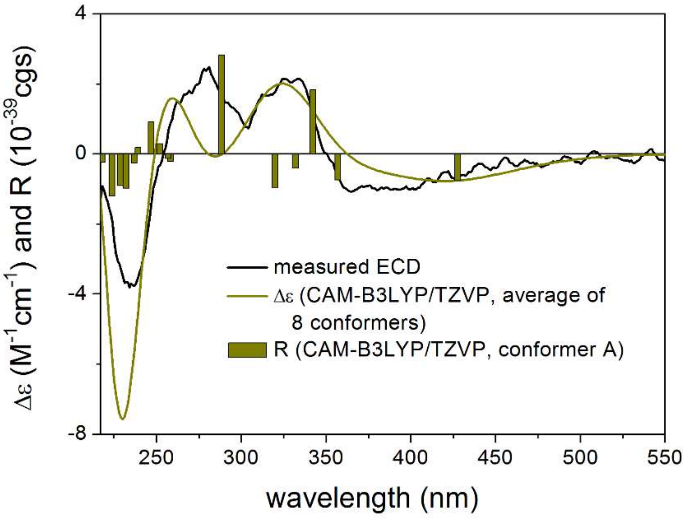

Supplementary Fig. 5. Comparison of experimental ECD and calculated ECD spectra of (1*R*,2*R*,3*S*)-7 in MeCN. (**a**) Structure and population of the low-energy ωB97X/TZVP PCM/MeCN conformers (> 1%) of (1*R*,2*R*,3*S*)- **7**. (**b**) Experimental ECD spectrum of **7** in MeCN compared with the Boltzmann-weighted CAM-B3LYP/TZVP PCM/MeCN ECD spectrum of (1*R*,2*R*,3*S*)-**7** computed for the ωB97X/TZVP PCM/MeCN conformers. Bars represent the rotational strength of the lowest-energy conformer.

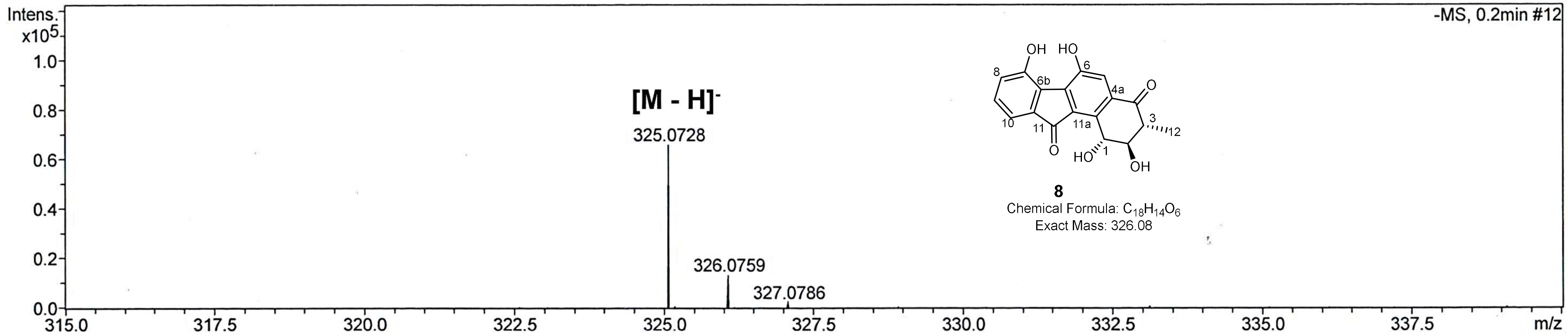

Meas. m/z	#	Ion Formula	Score	m/z	err [ppm]	err [mDa]	mSigma	rdb	e⁻ Conf	N-Rule
325.0728	1	C18H13O6	100.00	325.0718	-3.2	-1.0	1.4	12.5	even	ok
651.1518	1	C36H27O12	100.00	651.1508	-1.5	-1.0	3.1	23.5	even	ok

Supplementary Fig. 6 Spectroscopic data for FST B2 (8). (**a**) HRESIMS spectrum.

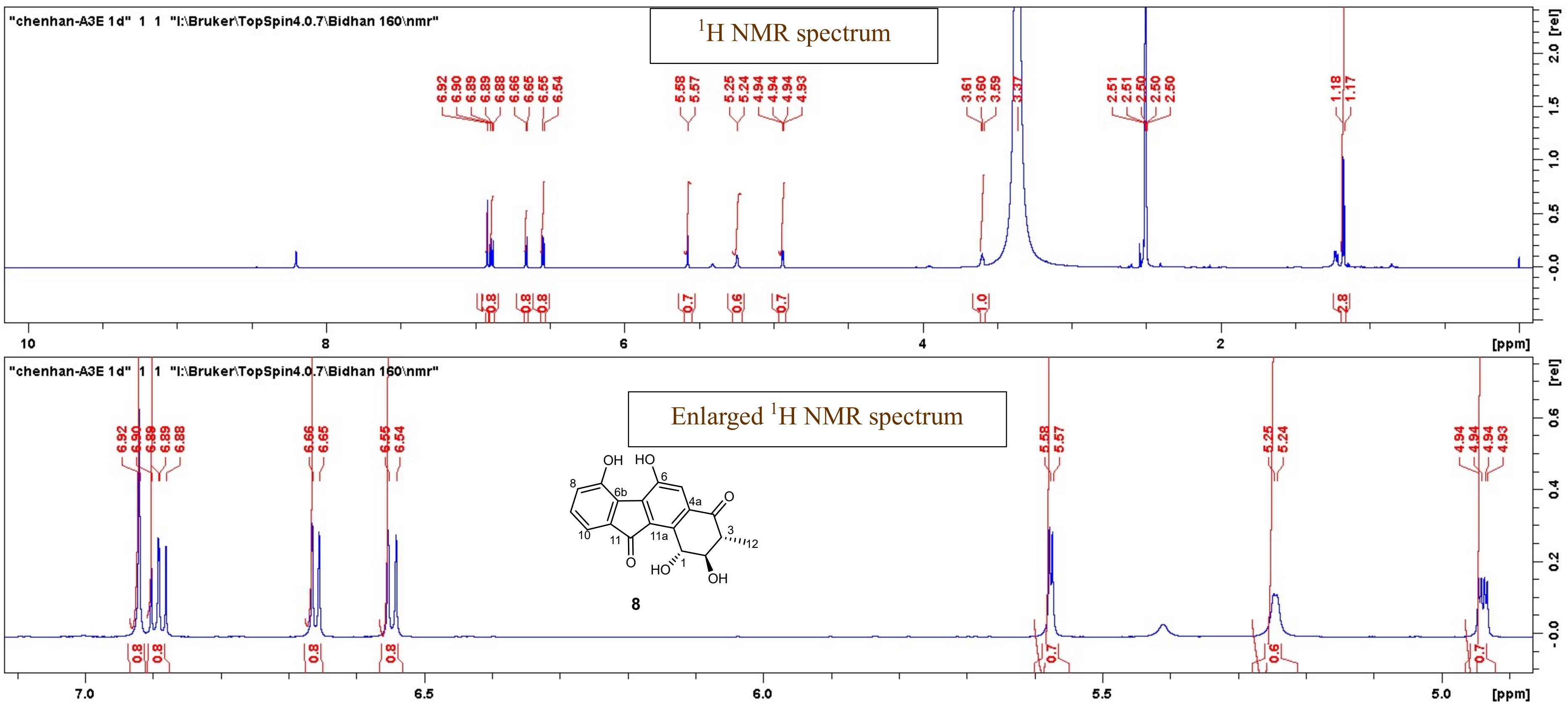

Supplementary Fig. 6 Spectroscopic data for FST B2 (8). (**b**) The ^{1}H and enlarged ^{1}H NMR spectrum of **8** in DMSO-d_6.

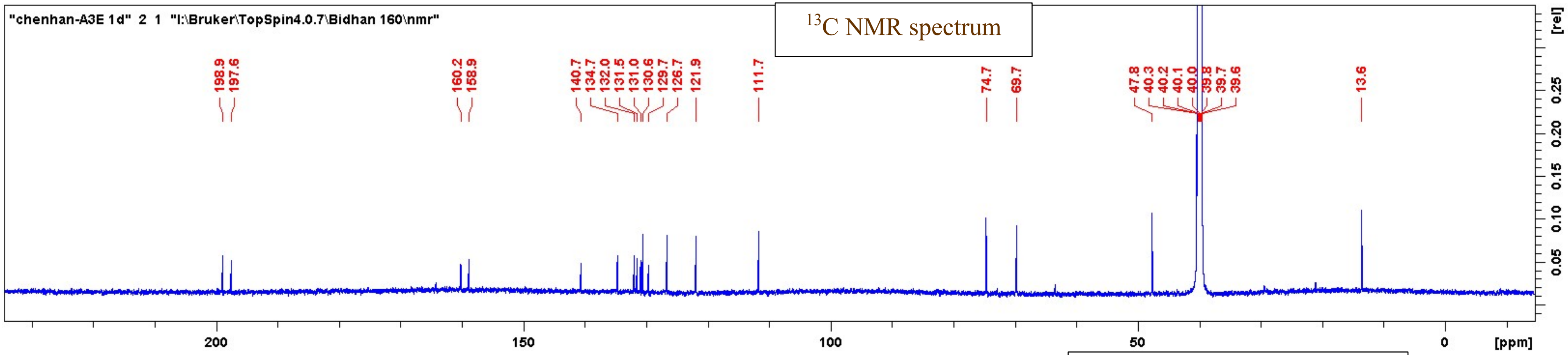

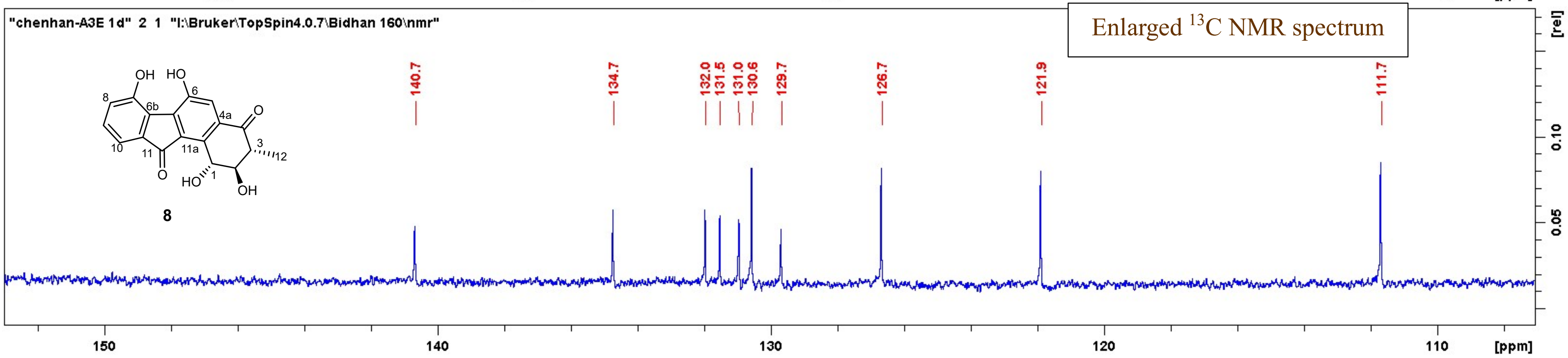

Supplementary Fig. 6 Spectroscopic data for FST B2 (8). (**c**) The ^{13}C and enlarged ^{13}C NMR spectrum of **8** in DMSO-d_6.

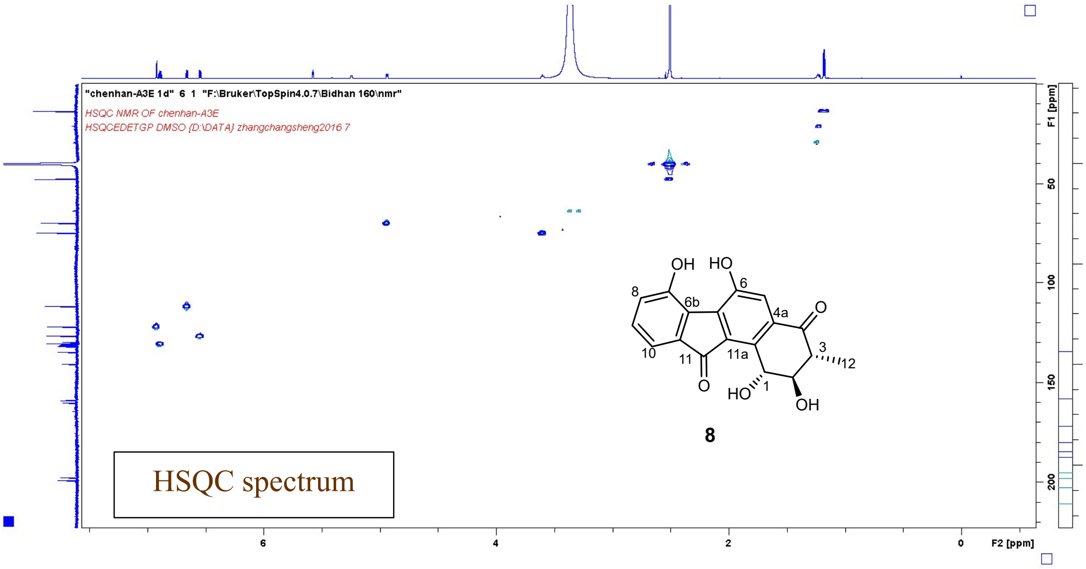

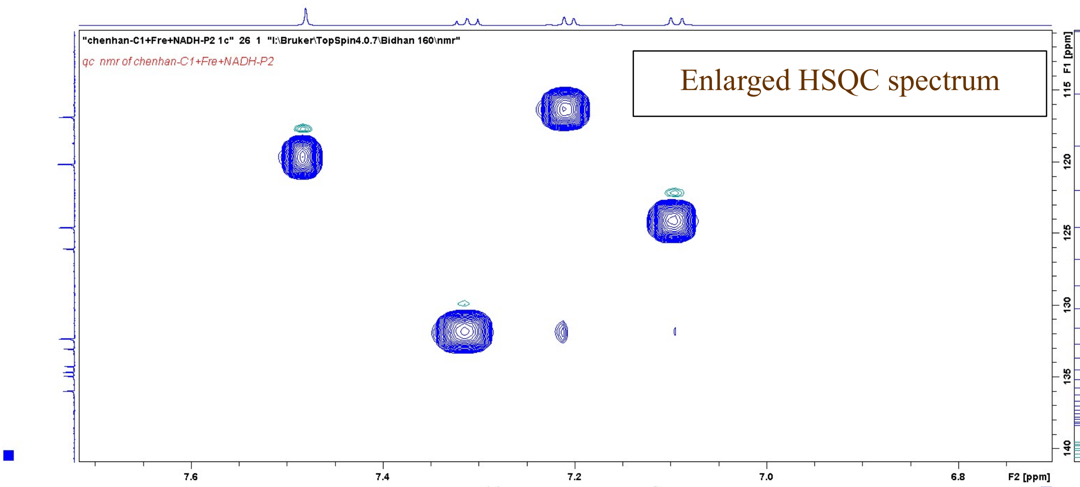

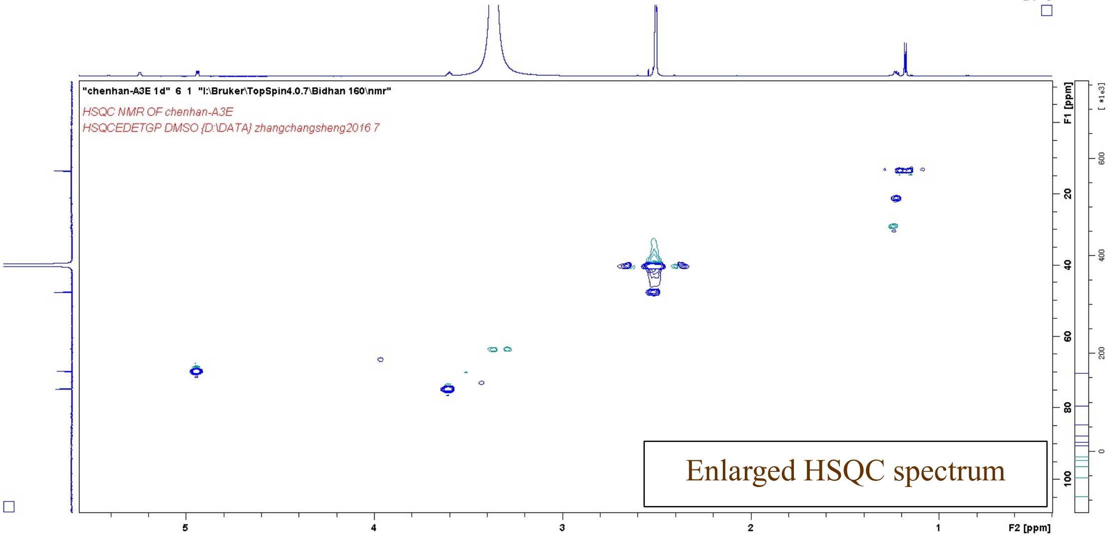

Supplementary Fig. 6 Spectroscopic data for FST B2 (8). (**d**) The HSQC and enlarged HSQC spectrum of **8** in DMSO-d_6.

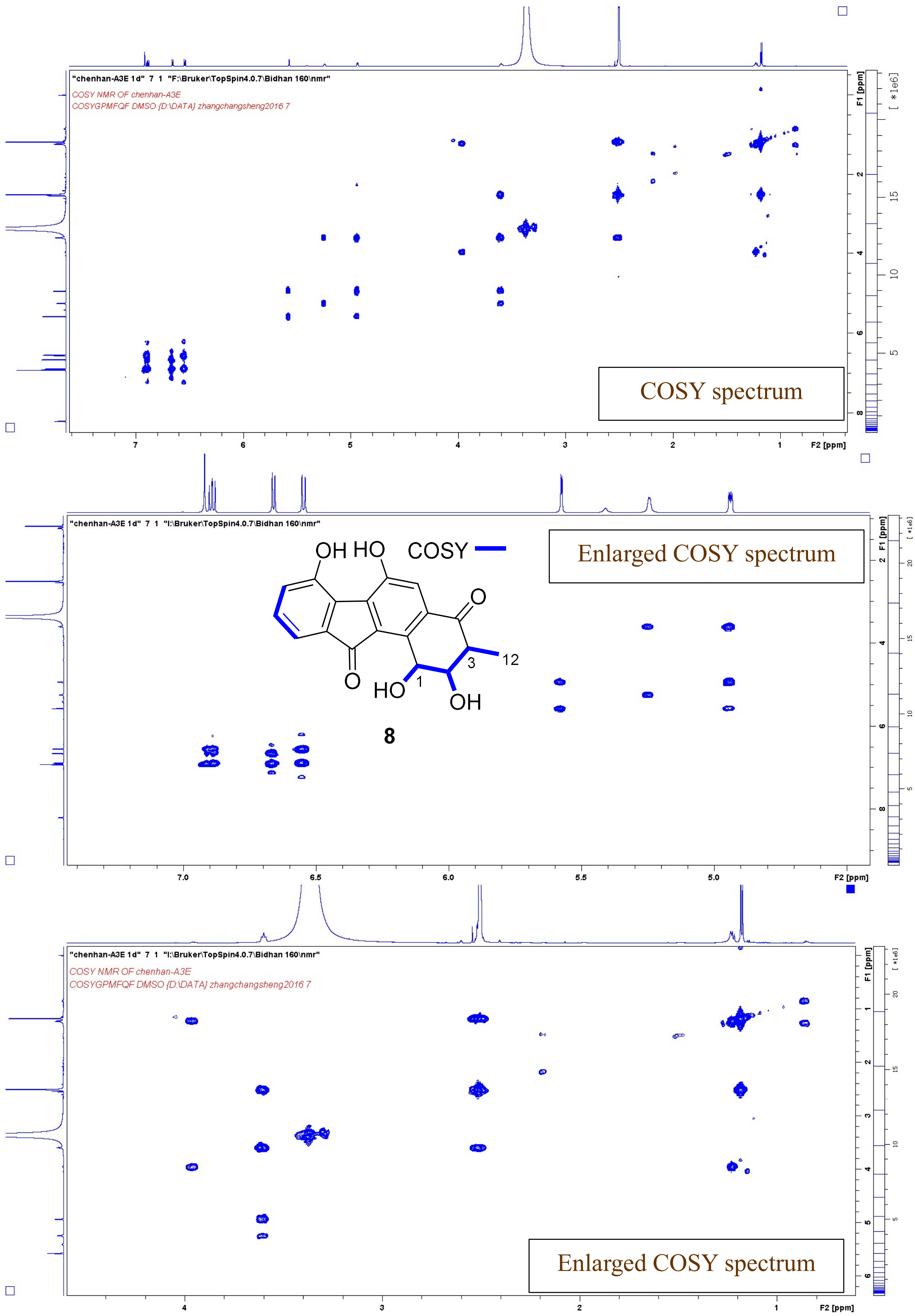

Supplementary Fig. 6 Spectroscopic data for FST B2 (8). (**e**) The COSY and enlarged COSY spectrum of **8** in DMSO-d_6.

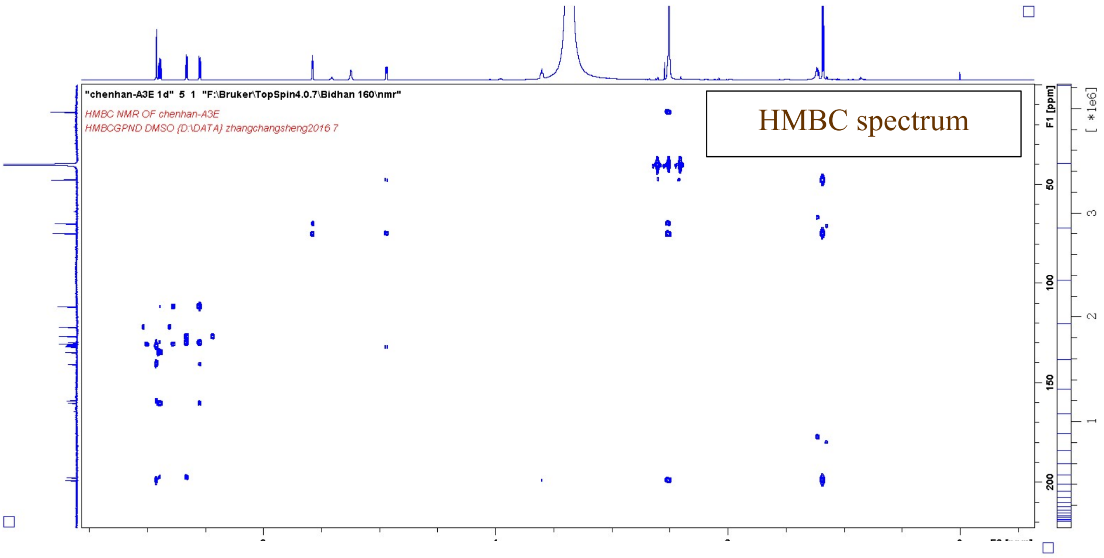

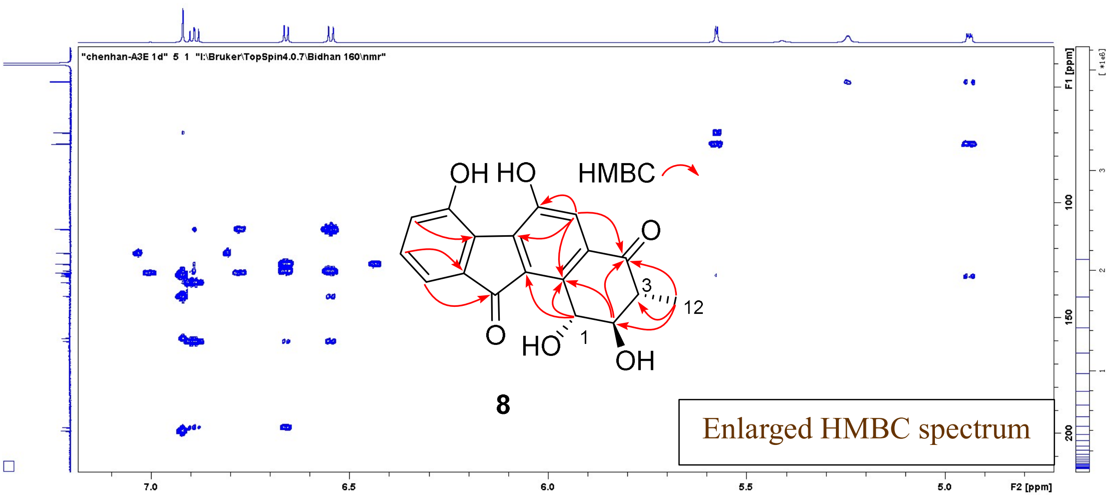

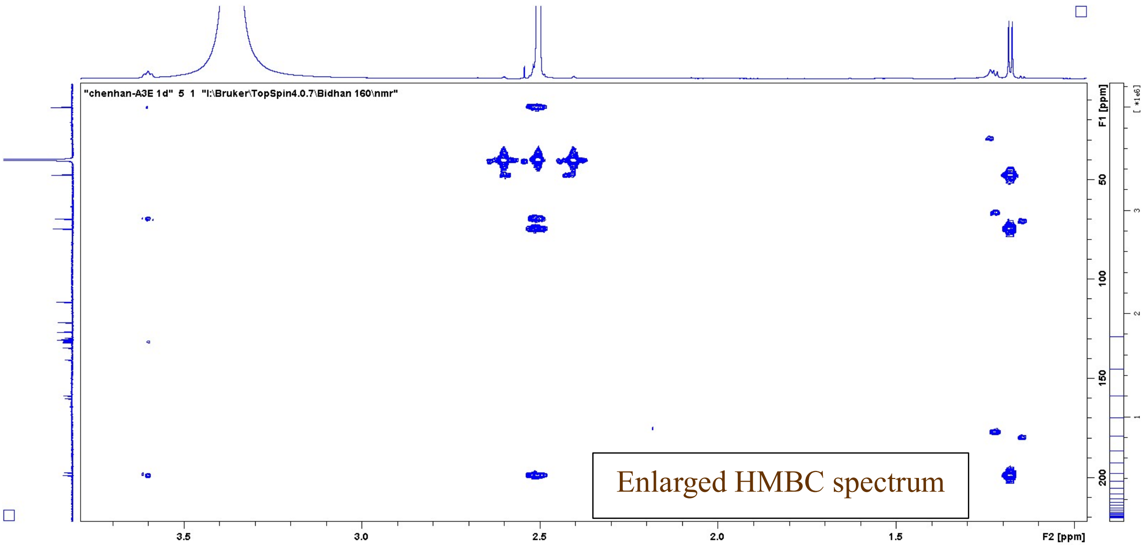

Supplementary Fig. 6 Spectroscopic data for FST B2 (8). (**f**) The HMBC and enlarged HMBC spectrum of **8** in DMSO-d_6.

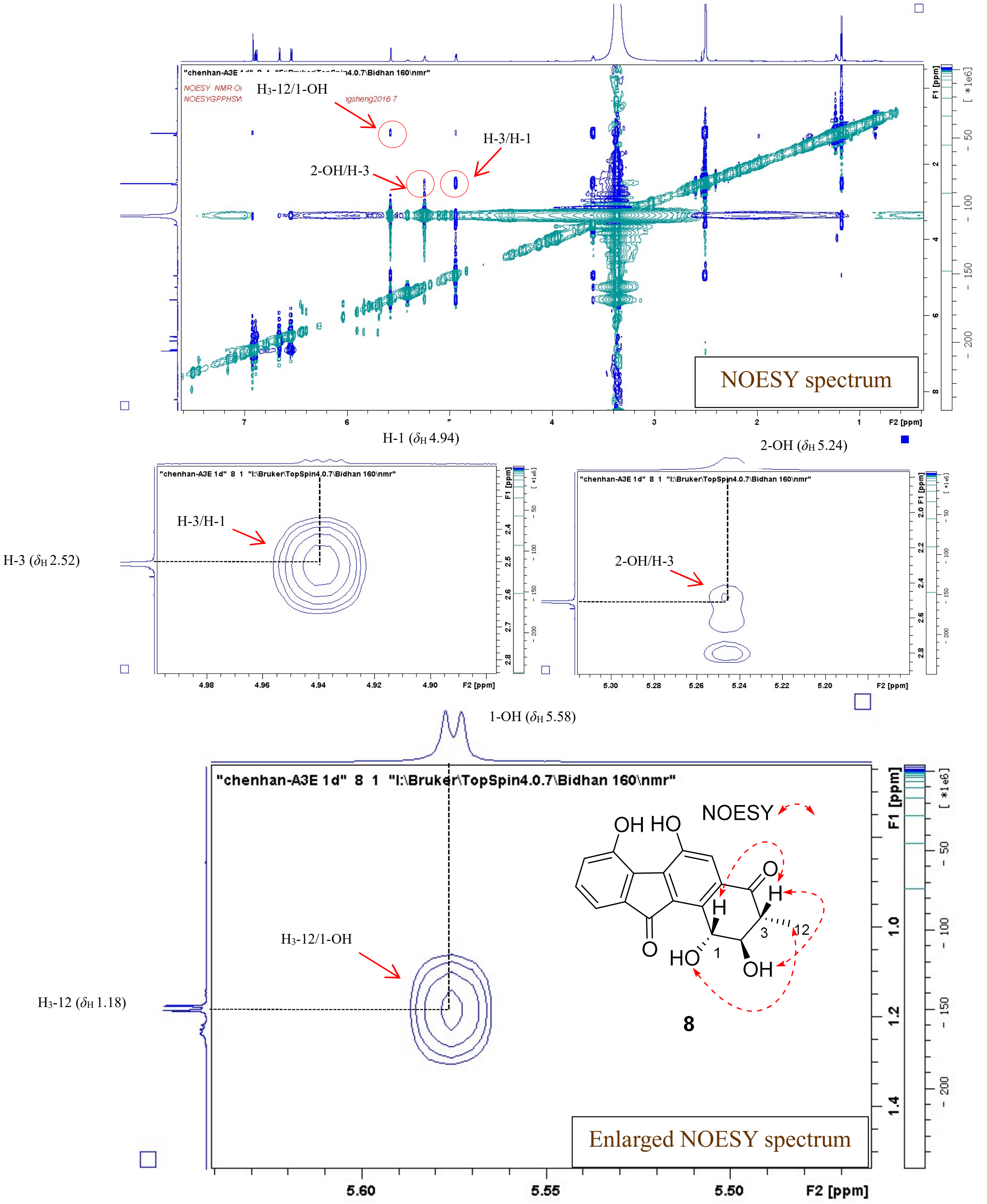

Supplementary Fig. 6 Spectroscopic data for FST B2 (8). (g) The NOESY and enlarged NOESY spectrum of **8** in DMSO-d_6.

a

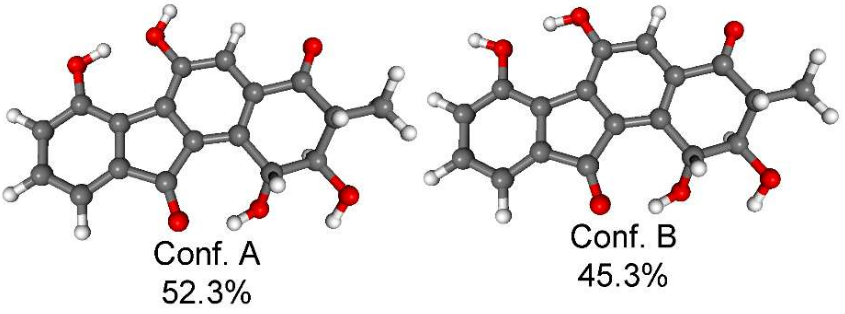

b

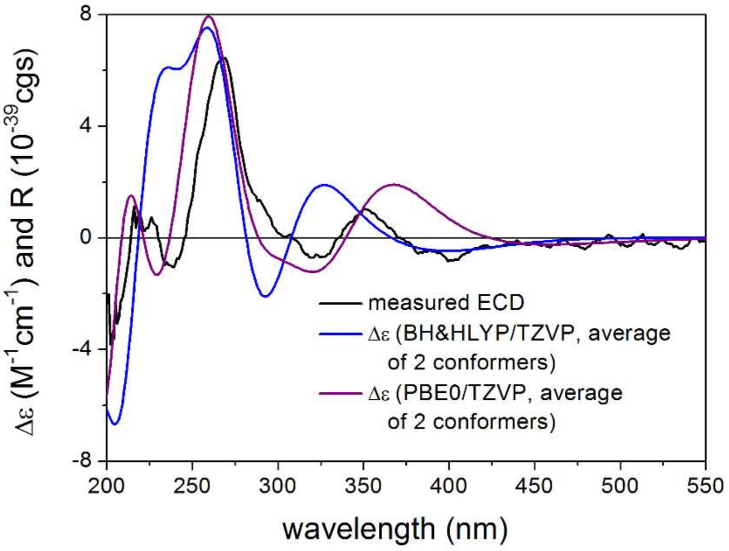

Supplementary Fig. 7. Comparison of experimental and calculated ECD spectra of 8 in MeCN. (**a**) Structure and population of the low-energy ωB97X/TZVP PCM/MeCN conformers (> 1%) of (1*R*,2*R*,3*R*)-**8**. (**b**) Experimental ECD spectrum of **8** in MeCN compared with the Boltzmann-weighted BH&HLYP/TZVP PCM/MeCN and PBE0/TZVP PCM/MeCN ECD spectra of (1*R*,2*R*,3*R*)-**8** computed for the ωB97X/TZVP PCM/MeCN conformers.

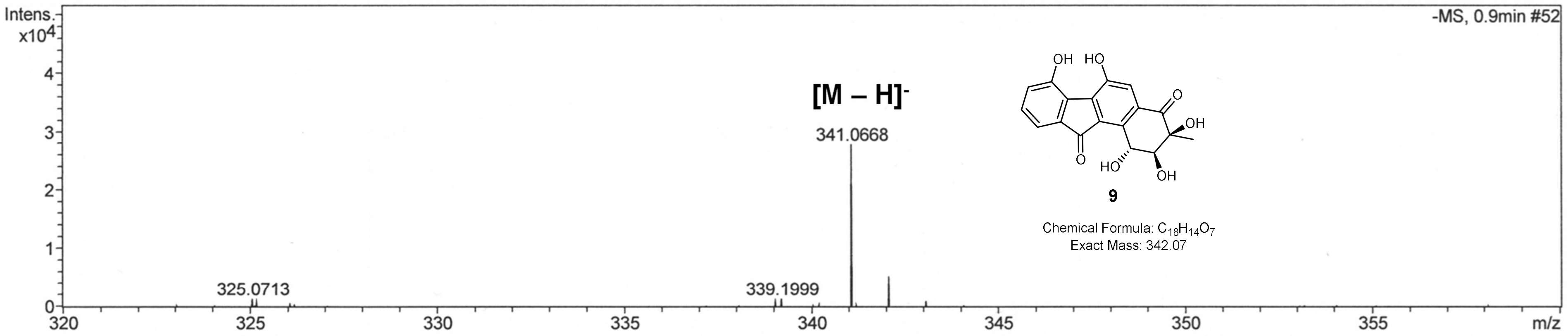

Meas. m/z	#	Ion Formula	Score	m/z	err [ppm]	err [mDa]	mSigma	rdb	e⁻ Conf	N-Rule
341.0668	1	C18H13O7	100.00	341.0667	0.5	0.2	4.5	12.5	even	ok
683.1404	1	C36H27O14	100.00	683.1406	-0.3	-0.2	11.3	23.5	even	ok

Supplementary Fig. 8. **Spectroscopic data for 9.** (**a**) HRESIMS spectrum.

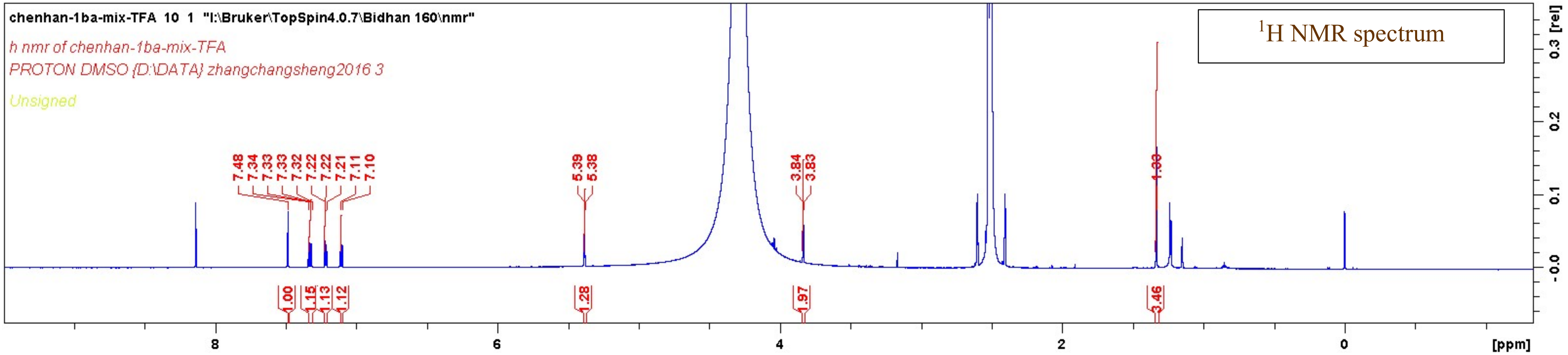

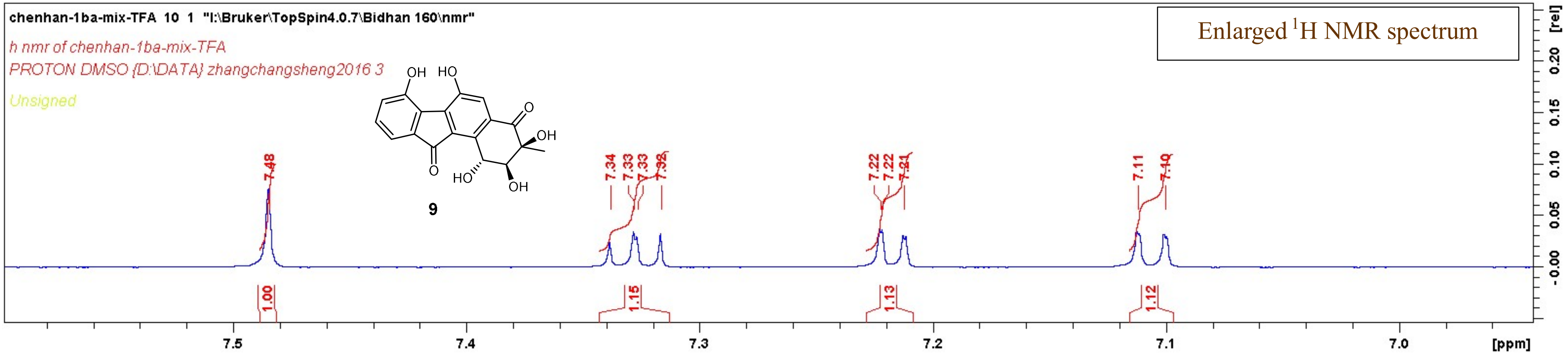

Supplementary Fig. 8. Spectroscopic data for 9. (**b**) The ^{1}H and enlarged ^{1}H NMR spectrum of **9** in DMSO-d_6:trifluoroacetic acid (TFA)-d_1 (100:1).

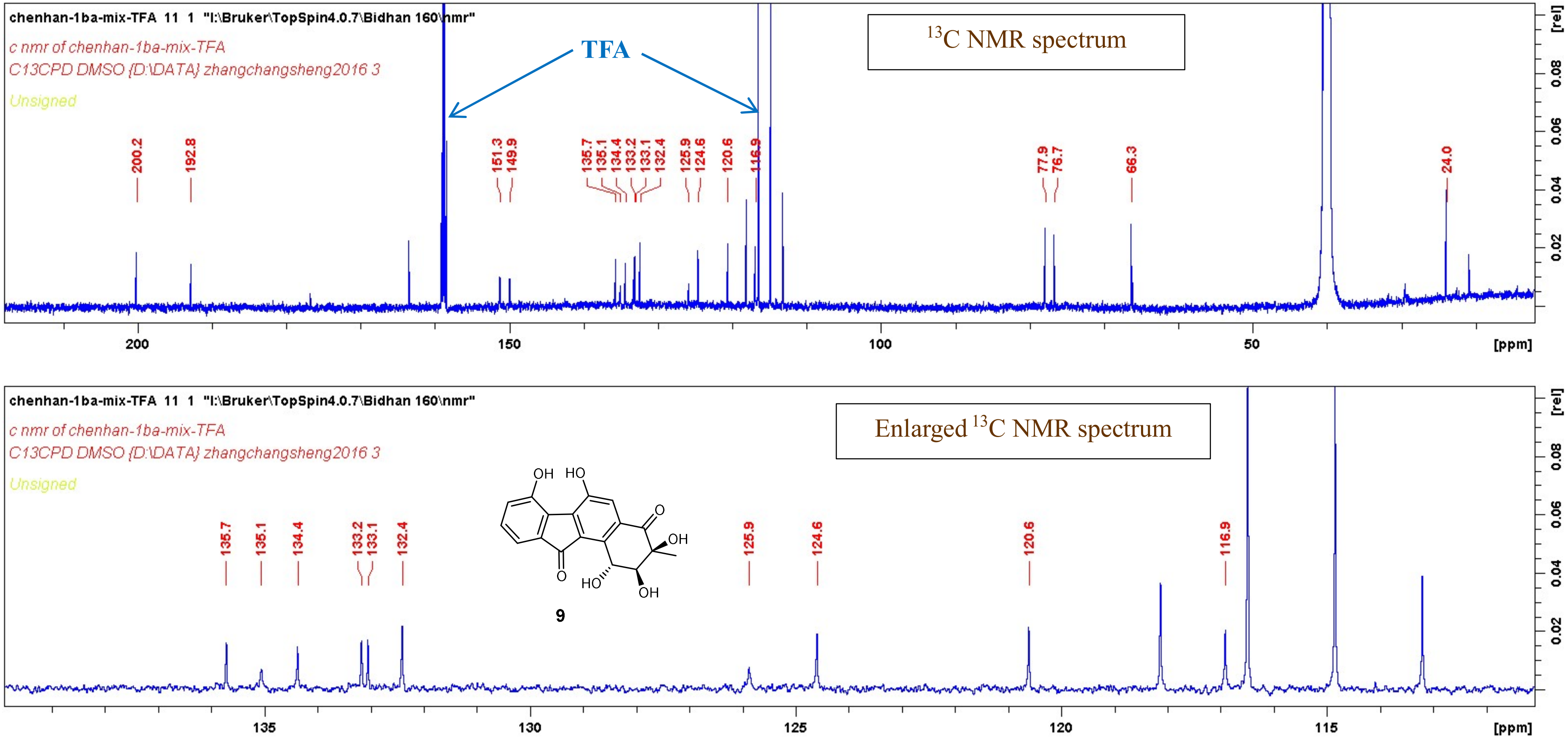

Supplementary Fig. 8. Spectroscopic data for 9. **(c)** The ^{13}C and enlarged ^{13}C NMR spectrum of **9** in DMSO-d_6:trifluoroacetic acid (TFA)-d_1 (100:1).

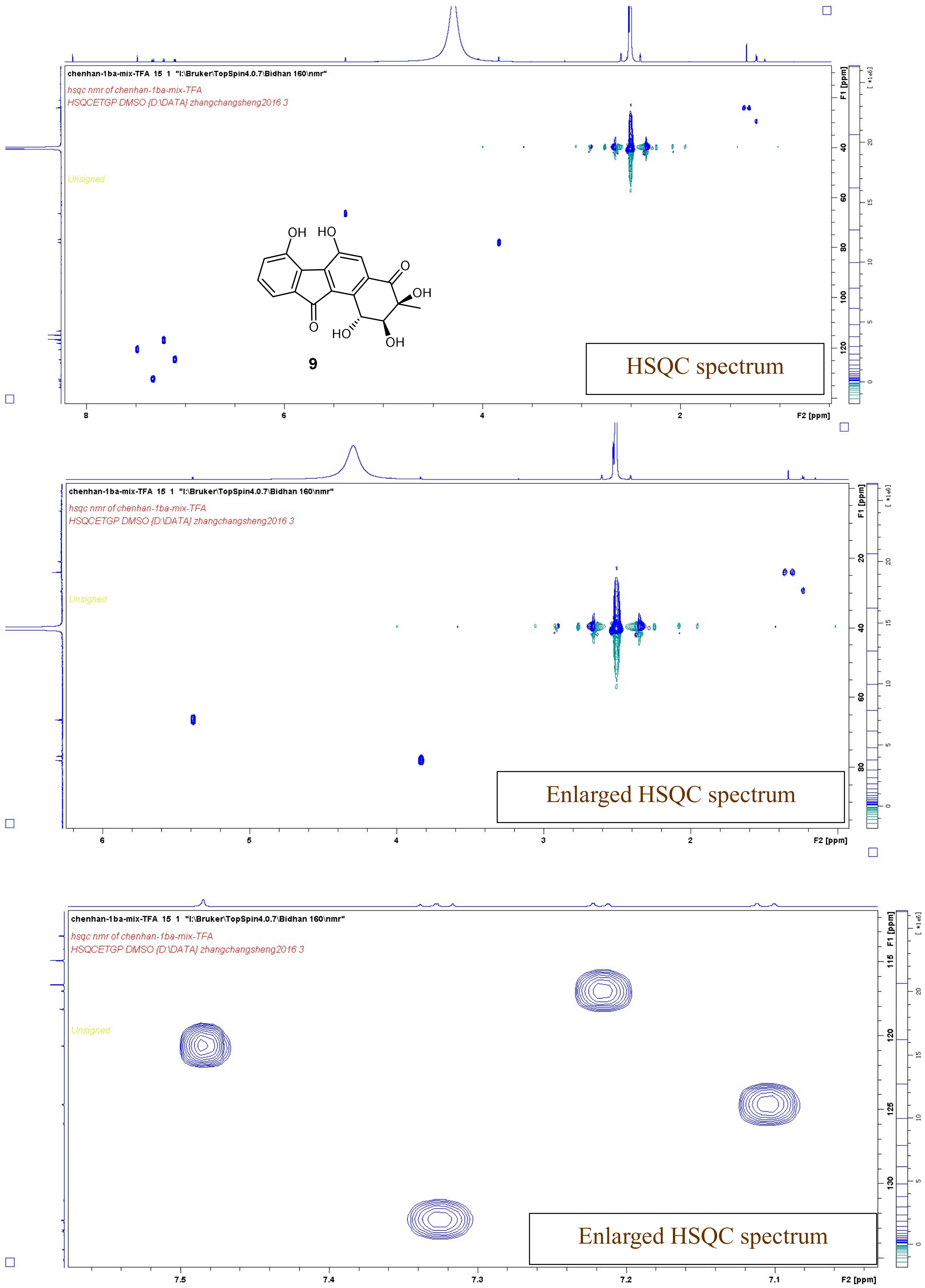

Supplementary Fig. 8. Spectroscopic data for 9. (**d**) The HSQC and enlarged HSQC spectrum of **9** in DMSO-d_6.

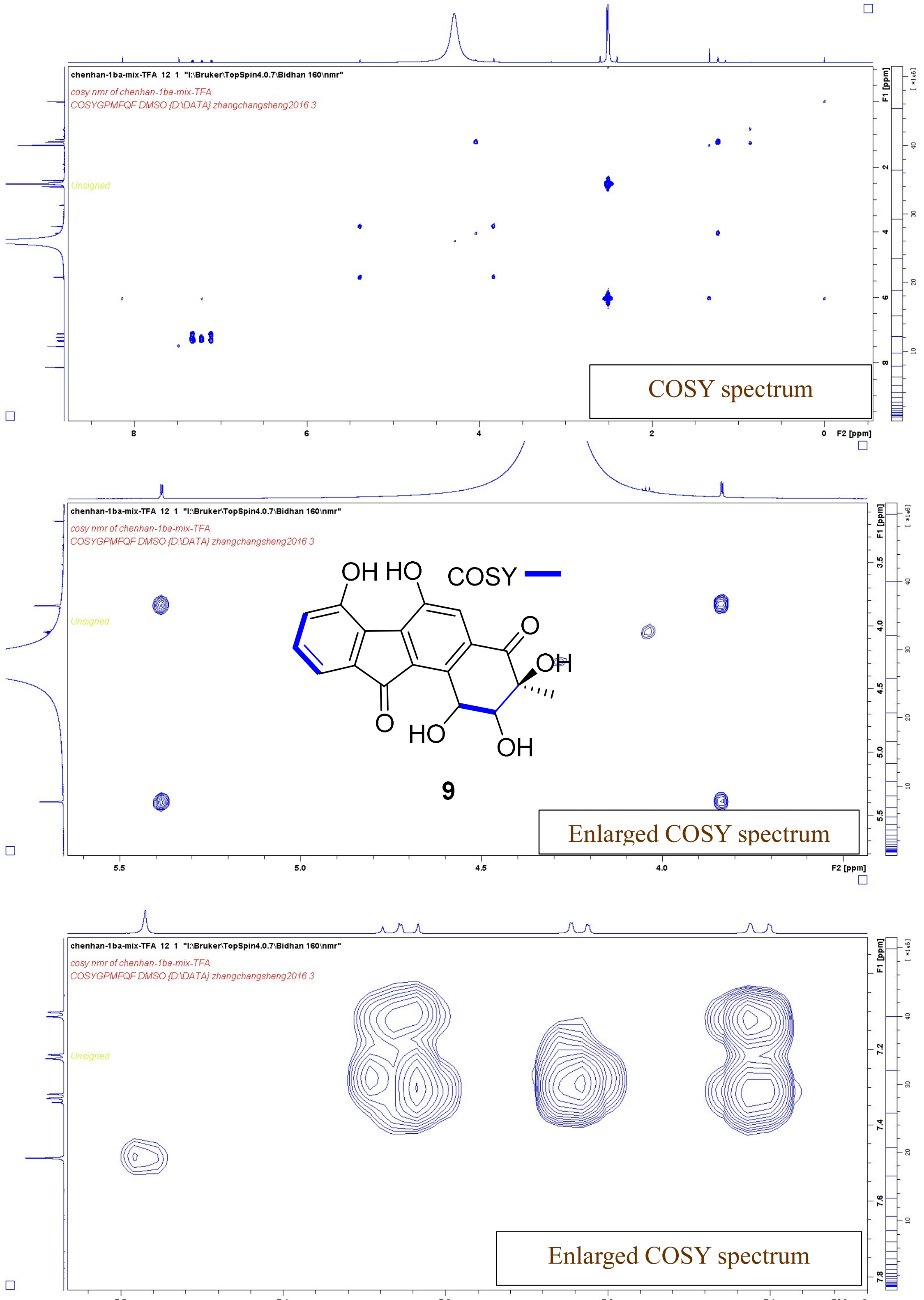

Supplementary Fig. 8. Spectroscopic data for 9. (**e**) The COSY and enlarged COSY spectrum of **9** in DMSO-d_6.

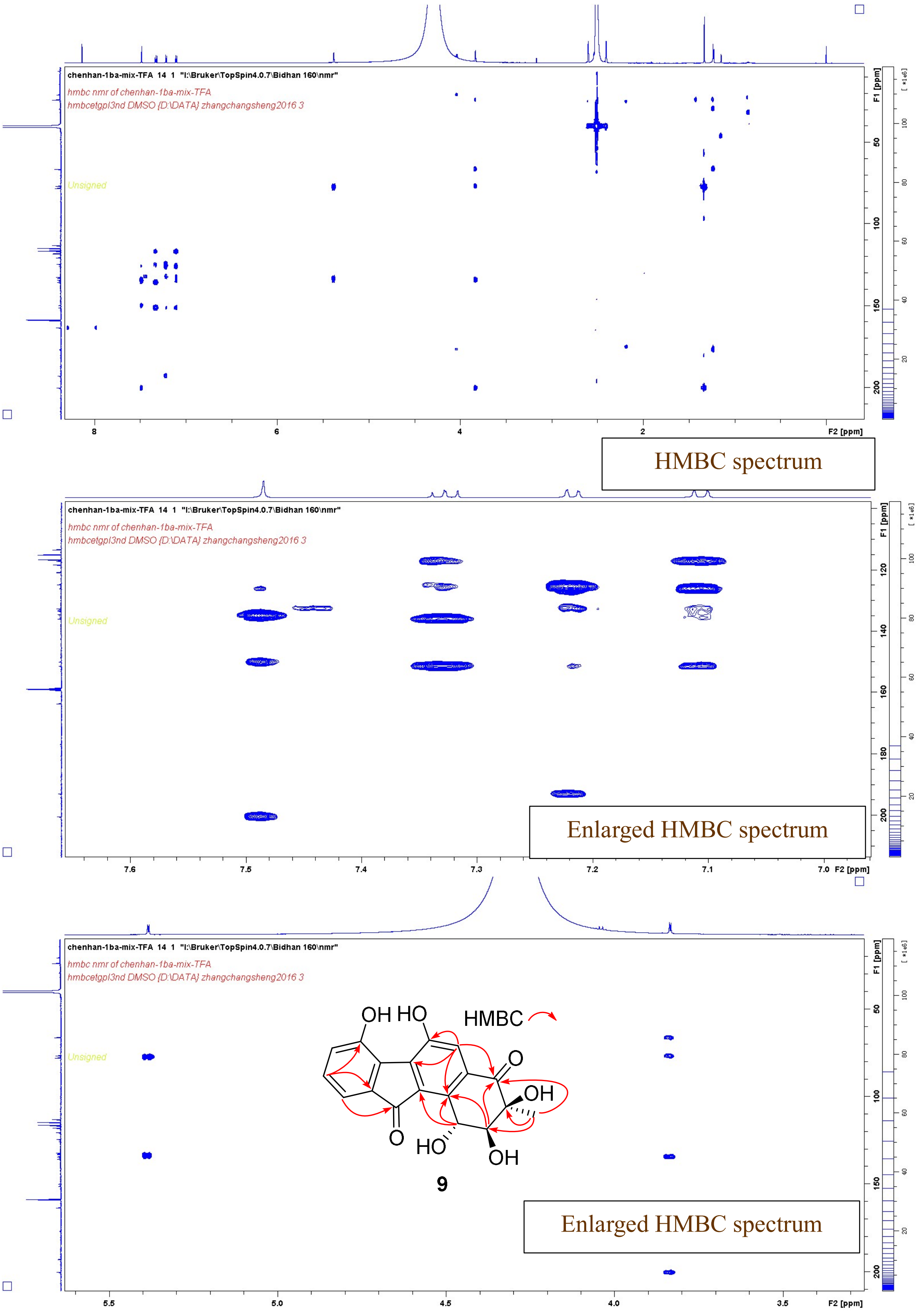

Supplementary Fig. 8. Spectroscopic data for 9. (**f**) The HMBC and enlarged HMBC spectrum of **9** in DMSO-d_6.

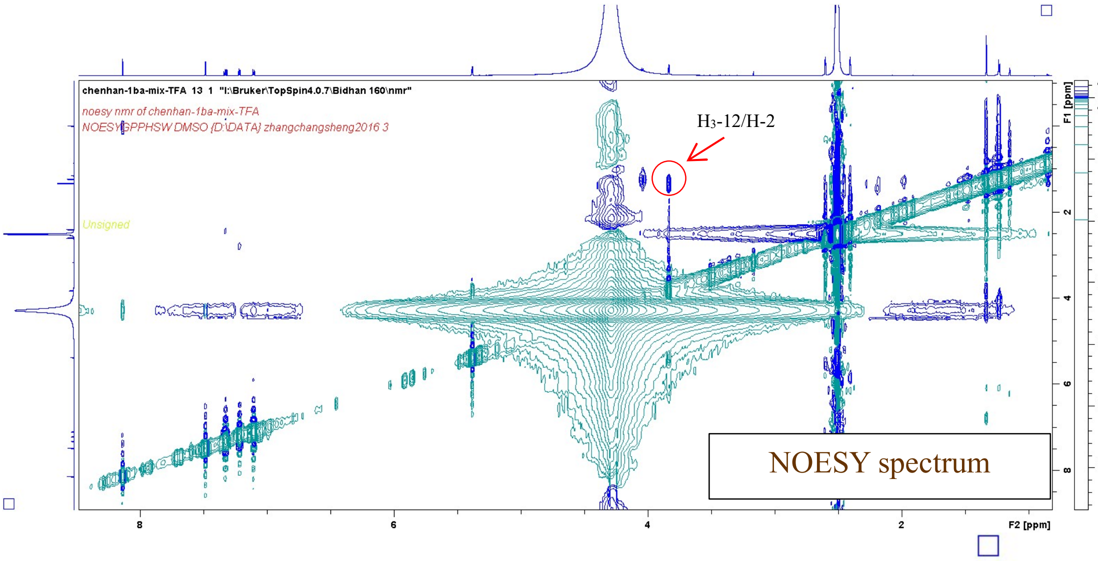

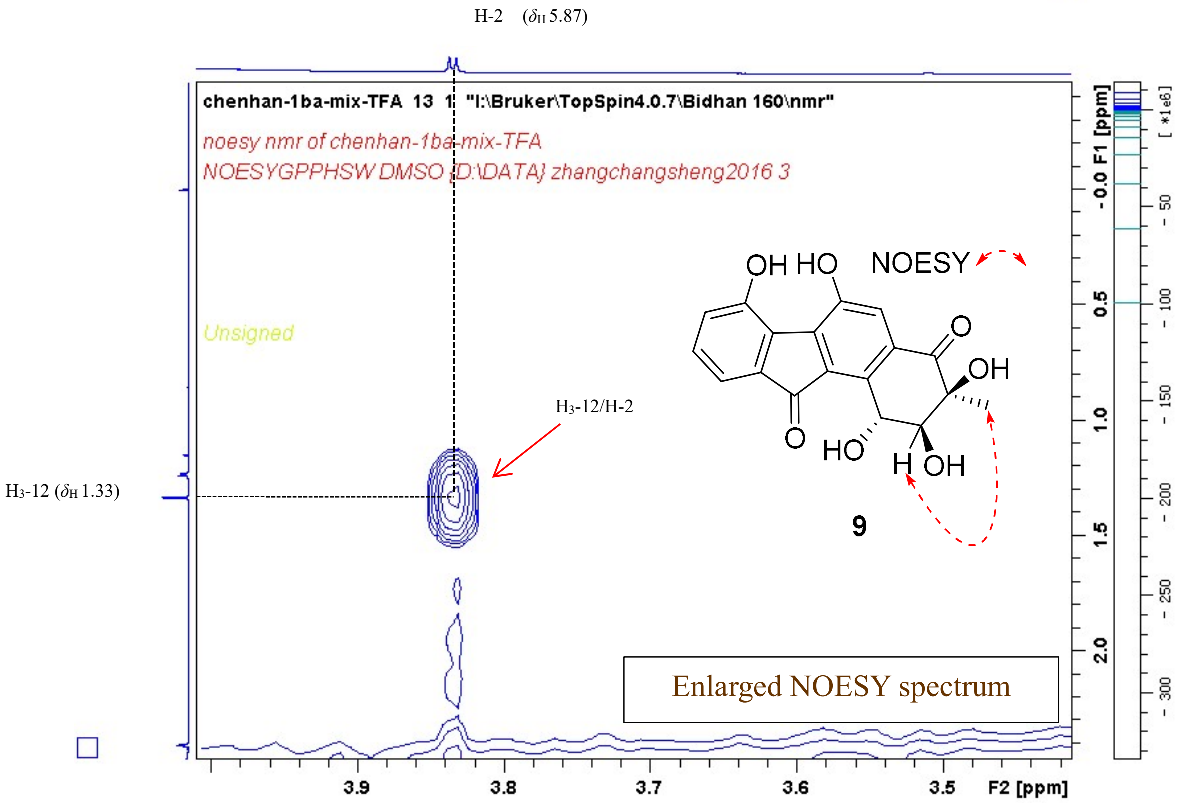

Supplementary Fig. 8. Spectroscopic data for 9. (**g**) The NOESY and enlarged NOESY spectrum of **9** in DMSO-d_6.

a

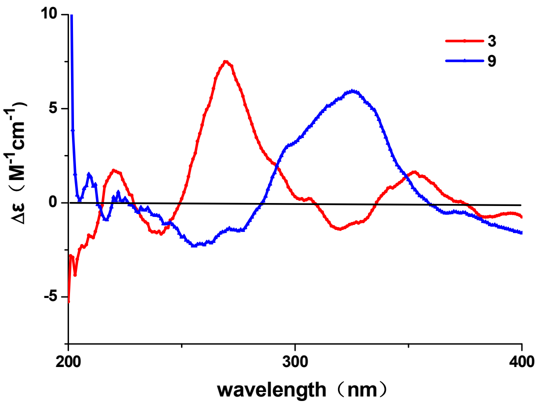

b

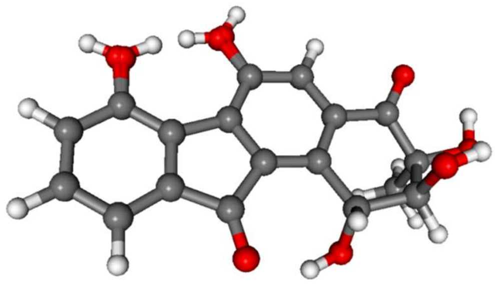

Overlapped conformers A and B (99%)

c

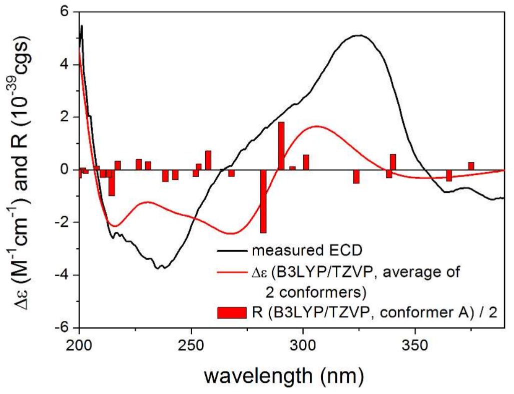

Supplementary Fig. 9. Comparison of the experimental ECD spectra of 3 with 9 and comparison of experimental and calculated ECD spectra of 9 in MeCN. (**a**) Comparison of the experimental ECD spectrum of **9** with **3**. (**b**) Structure and population of the low-energy ωB97X/TZVP PCM/MeCN conformers (> 1%) of (1*R*,2*S*,3*S*)-**9**. (**c**) Experimental ECD spectrum of **9** in MeCN compared with the Boltzmann-weighted B3LYP/TZVP PCM/MeCN ECD spectrum of (1*R*,2*S*,3*S*)-**9** computed for the ωB97X/TZVP PCM/MeCN conformers.

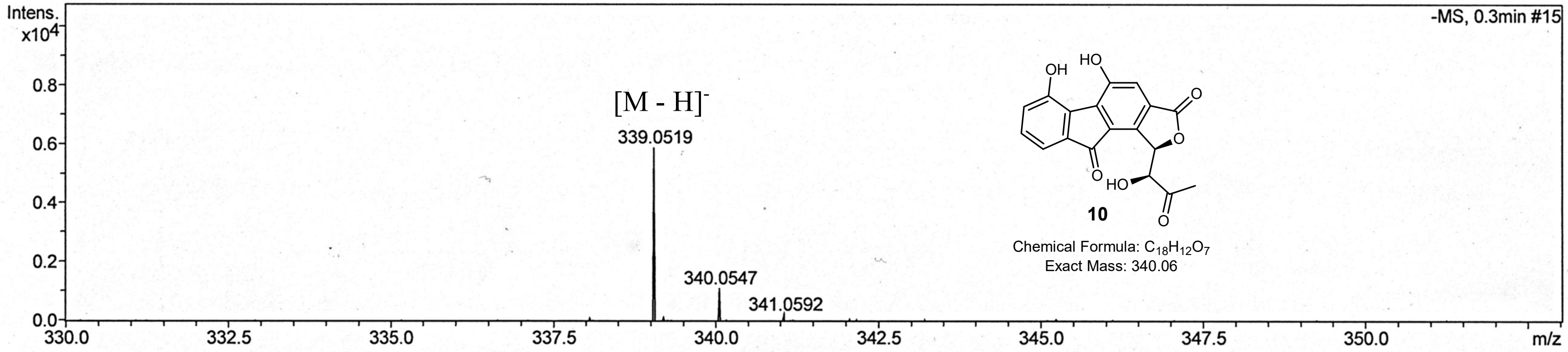

Meas. m/z	#	Ion Formula	Score	m/z	err [ppm]	err [mDa]	mSigma	rdb	e⁻ Conf	N-Rule
339.0519	1	C18H11O7	100.00	339.0510	-2.5	-0.8	6.0	13.5	even	ok
679.1112	1	C36H23O14	100.00	679.1093	-2.8	-1.9	18.8	25.5	even	ok

Supplementary Fig. 10. Spectroscopic data for 10. (**a**) HRESIMS spectrum.

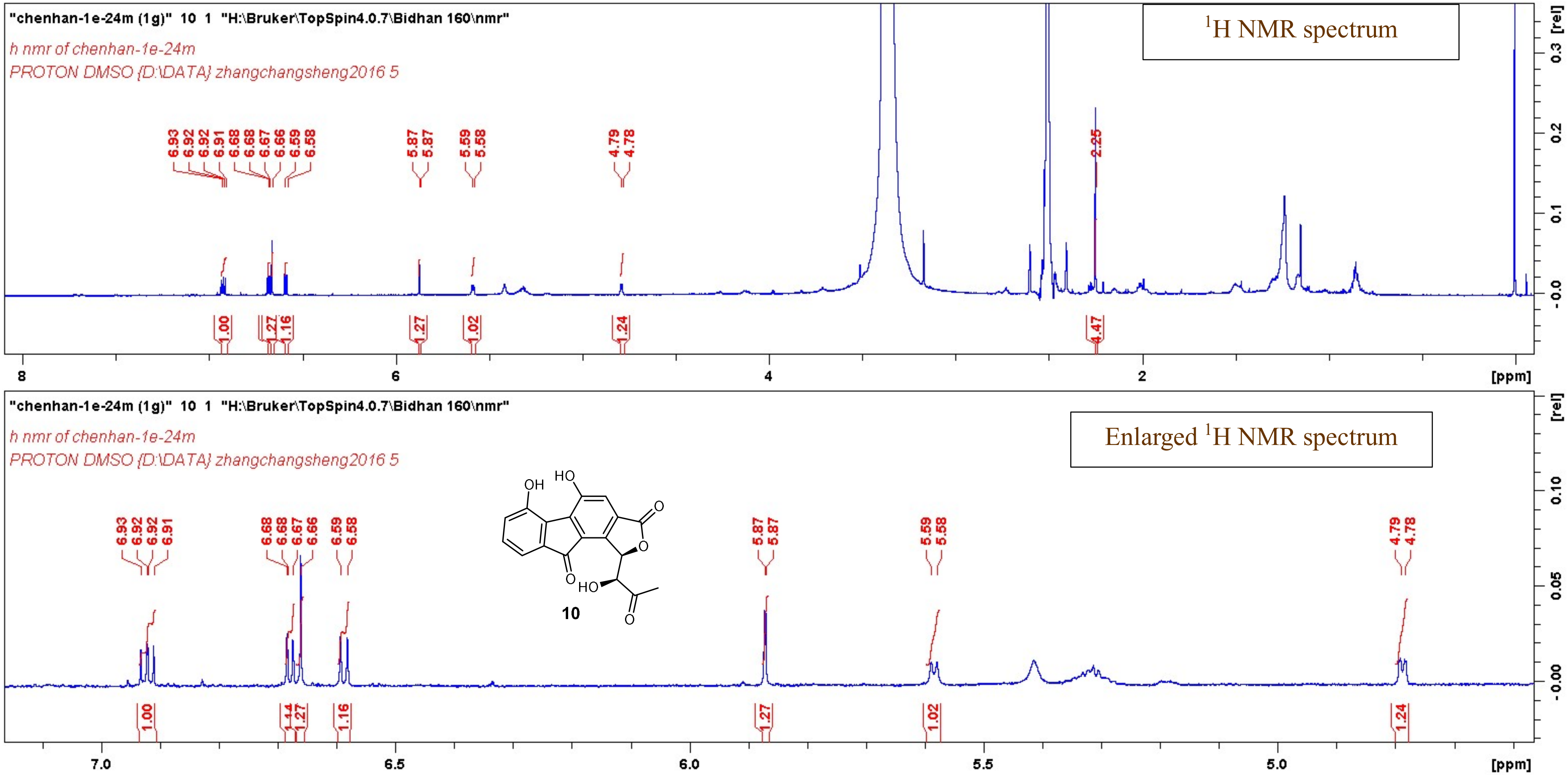

Supplementary Fig. 10. **Spectroscopic data for 10.** (**b**) The ^{1}H and enlarged ^{1}H NMR spectrum of **10** in DMSO-d_6.

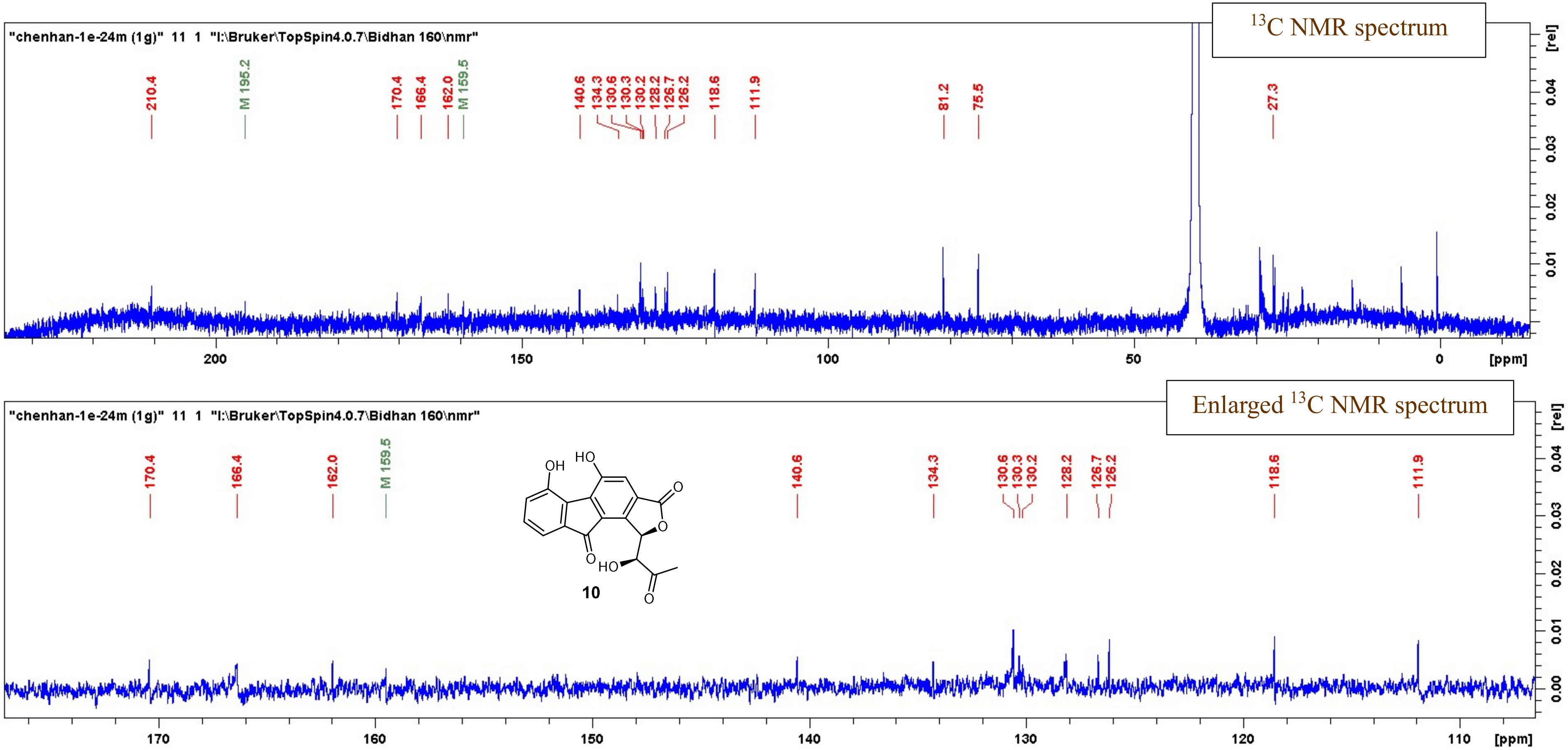

Supplementary Fig. 10. **Spectroscopic data for 10.** (**B**) (**c**) The ^{13}C and enlarged ^{13}C NMR spectrum of **10** in DMSO-d_6.

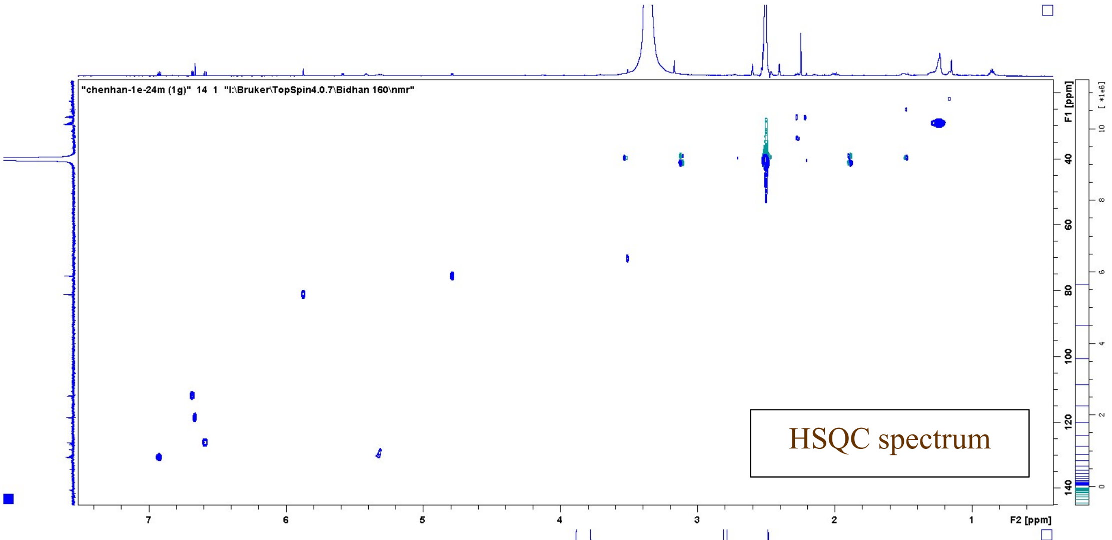

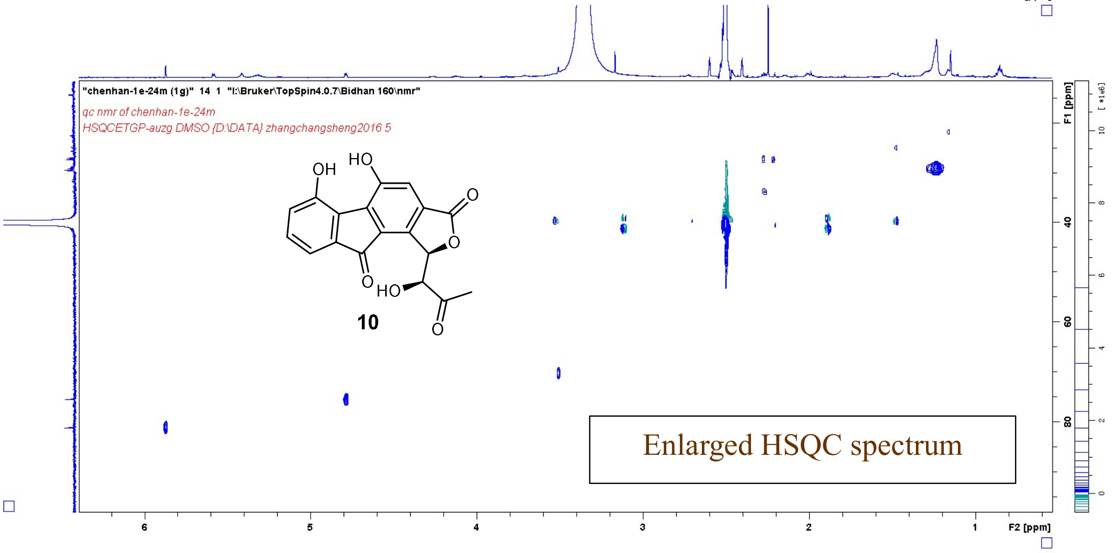

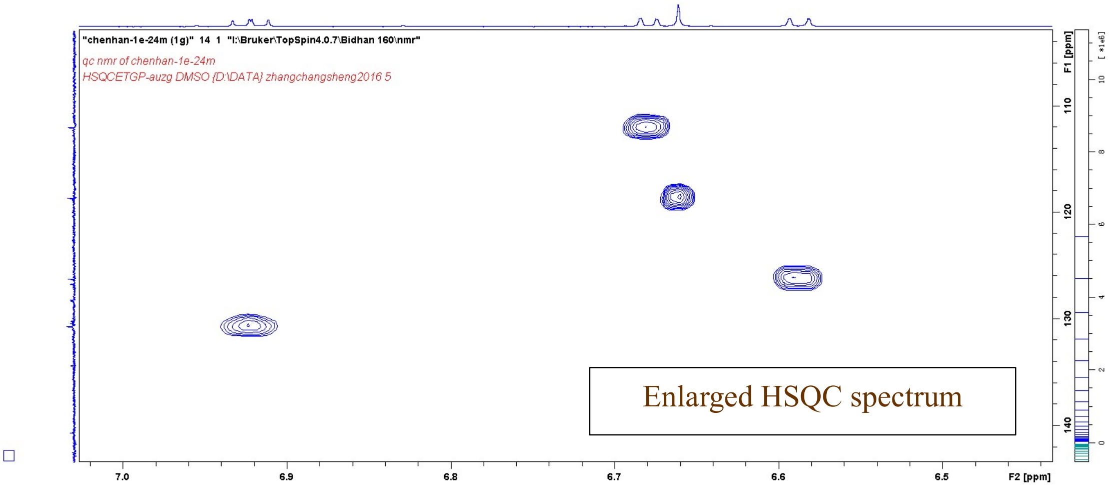

Supplementary Fig. 10. Spectroscopic data for 10. (**d**) The HSQC and enlarged HSQC spectrum of **10** in DMSO-d_6.

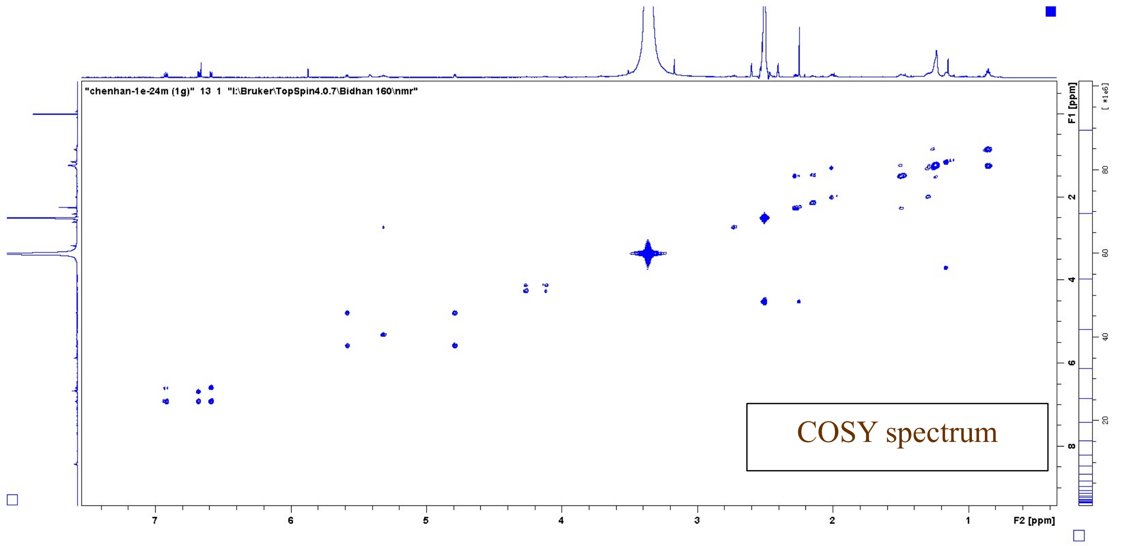

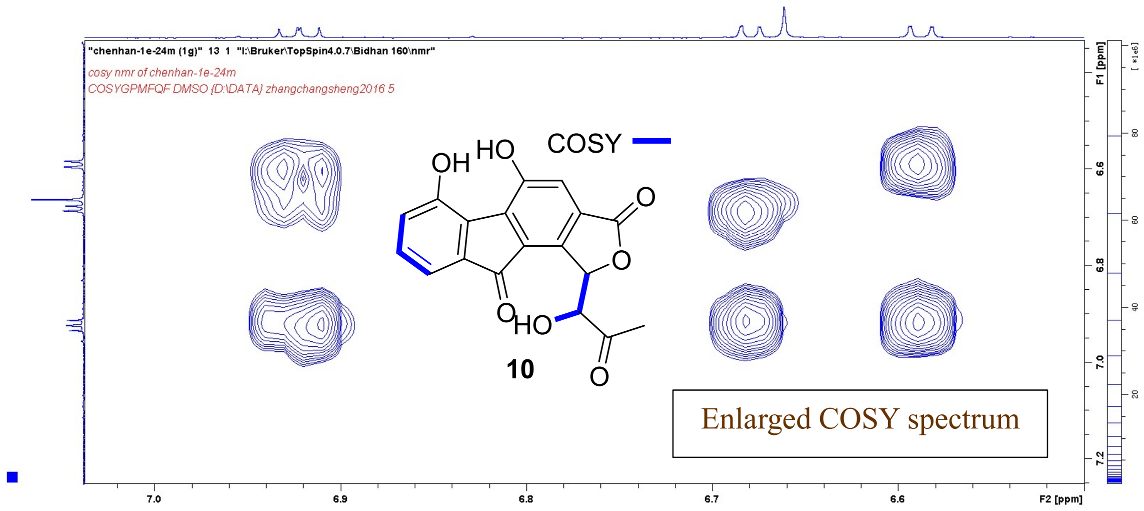

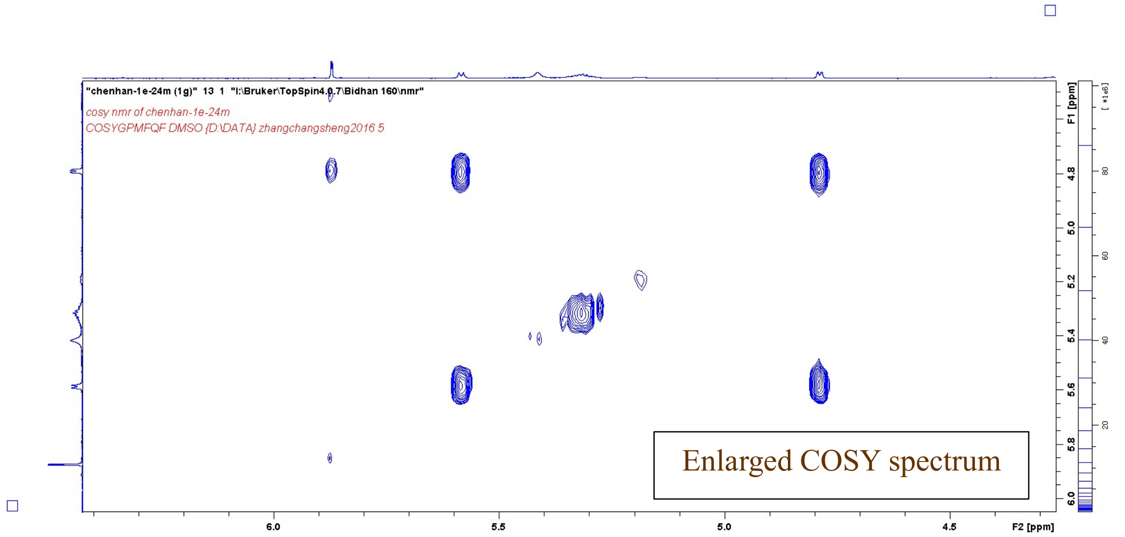

Supplementary Fig. 10. Spectroscopic data for 10. (**e**) The COSY and enlarged COSY spectrum of **10** in DMSO-d_6.

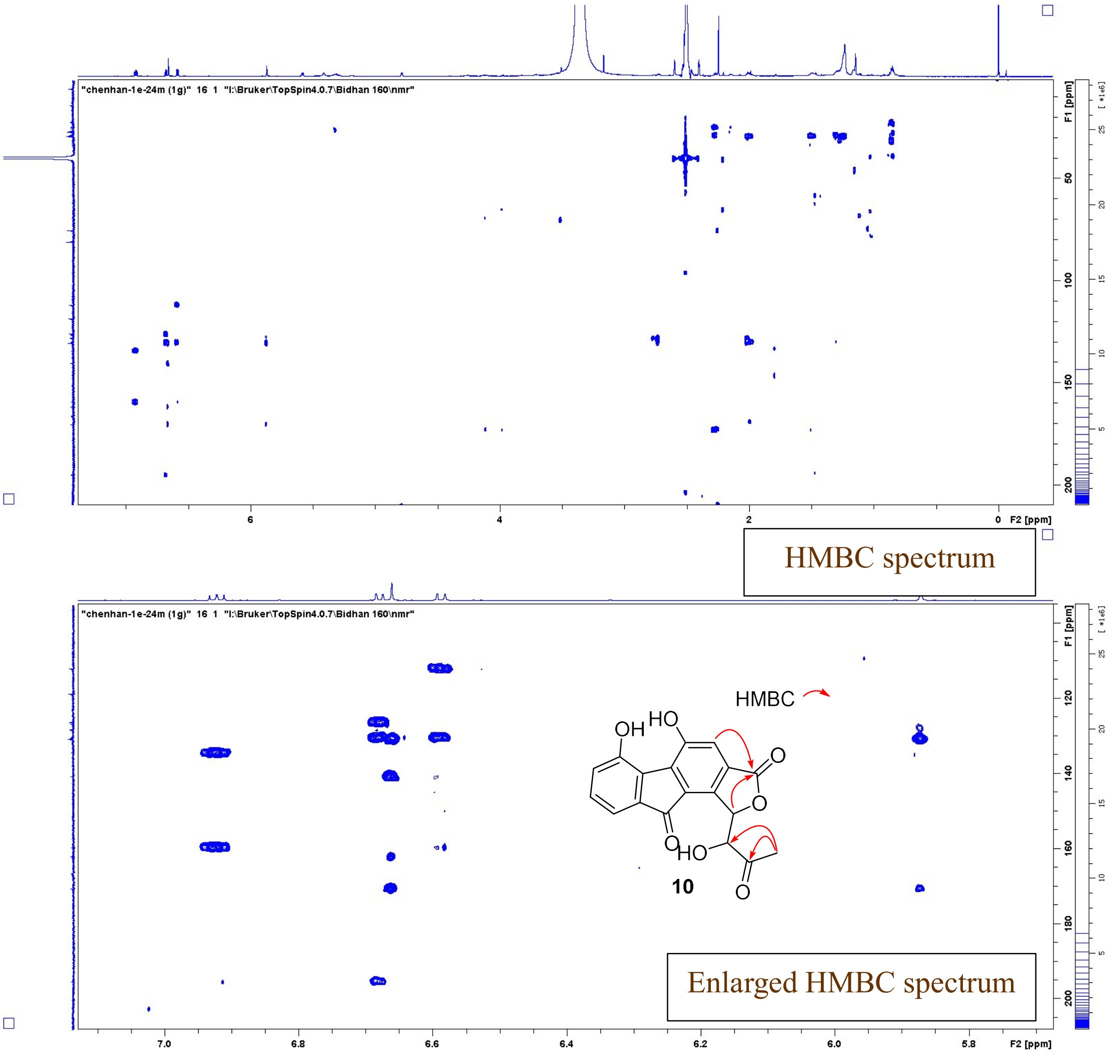

Supplementary Fig. 10. Spectroscopic data for 10. (**f**) The HMBC and enlarged HMBC spectrum of **10** in DMSO-d_6.

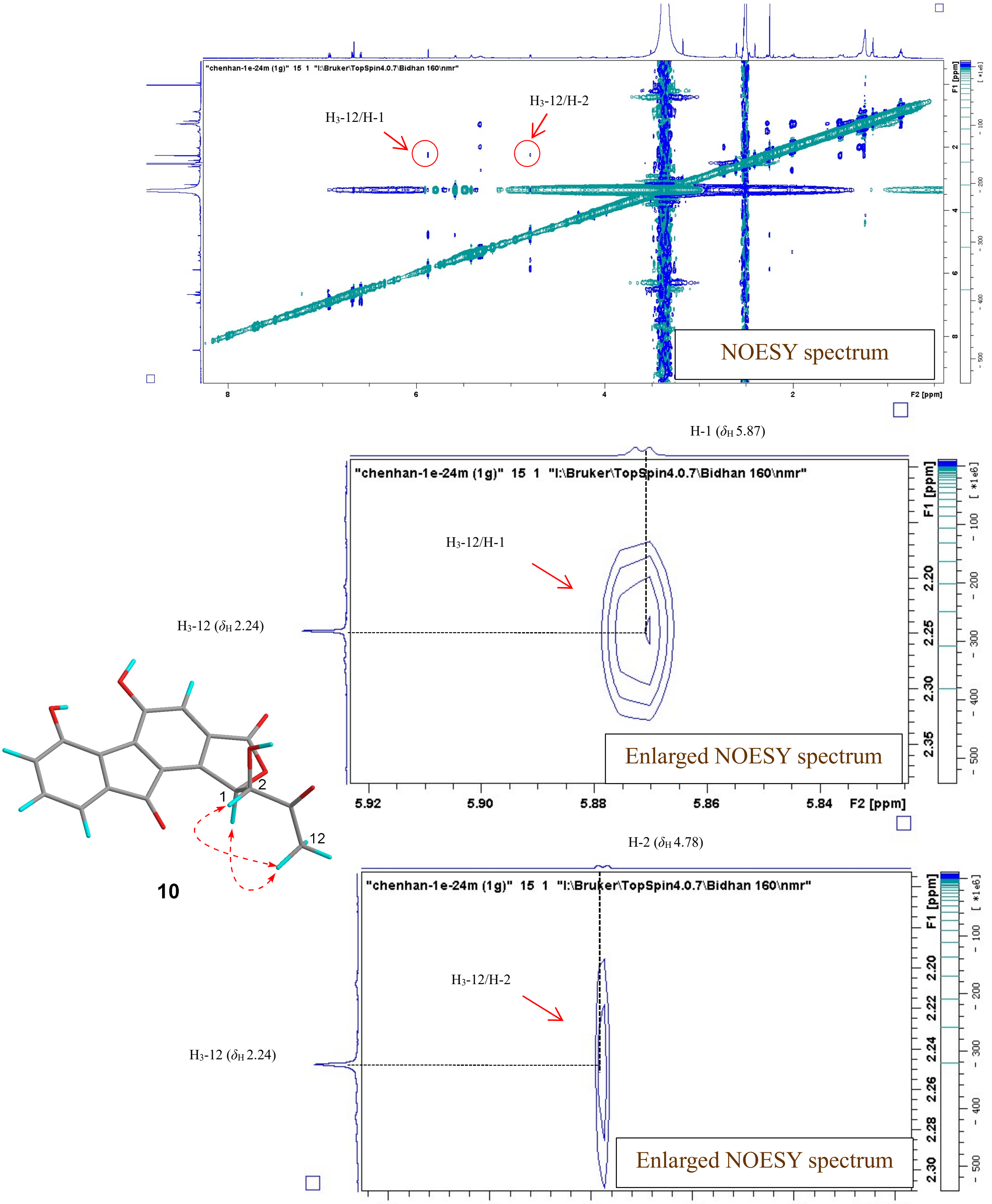

Supplementary Fig. 10. Spectroscopic data for 10. (g) The NOESY and enlarged NOESY spectrum of **10** in DMSO-d_6.

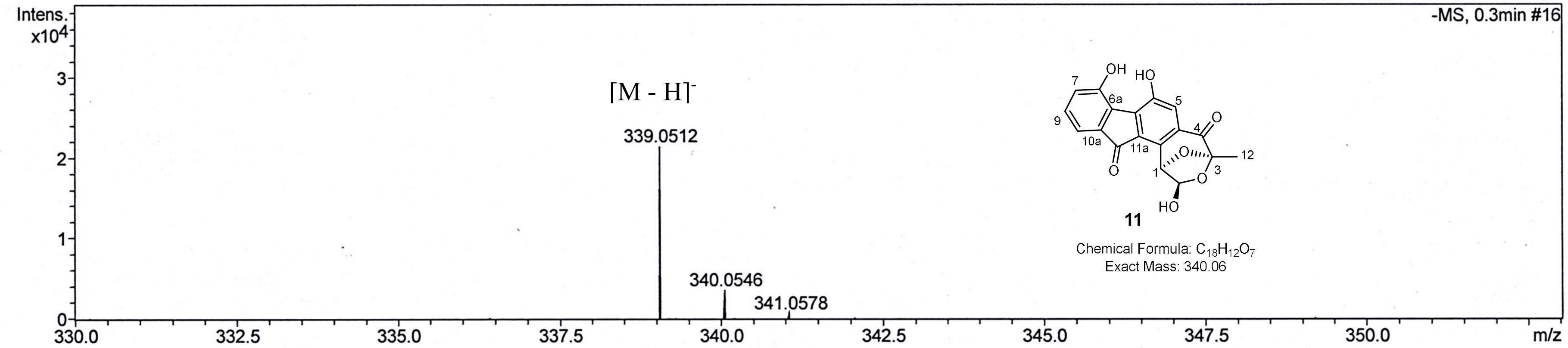

Meas. m/z	#	Ion Formula	Score	m/z	err [ppm]	err [mDa]	mSigma	rdb	e⁻ Conf	N-Rule
339.0512	1	C18H11O7	100.00	339.0510	-0.7	-0.2	13.8	13.5	even	ok
679.1101	1	C36H23O14	100.00	679.1093	-1.2	-0.8	2.2	25.5	even	ok

Supplementary Fig. 11 Spectroscopic data for 11. (**a**) HRESIMS spectrum.

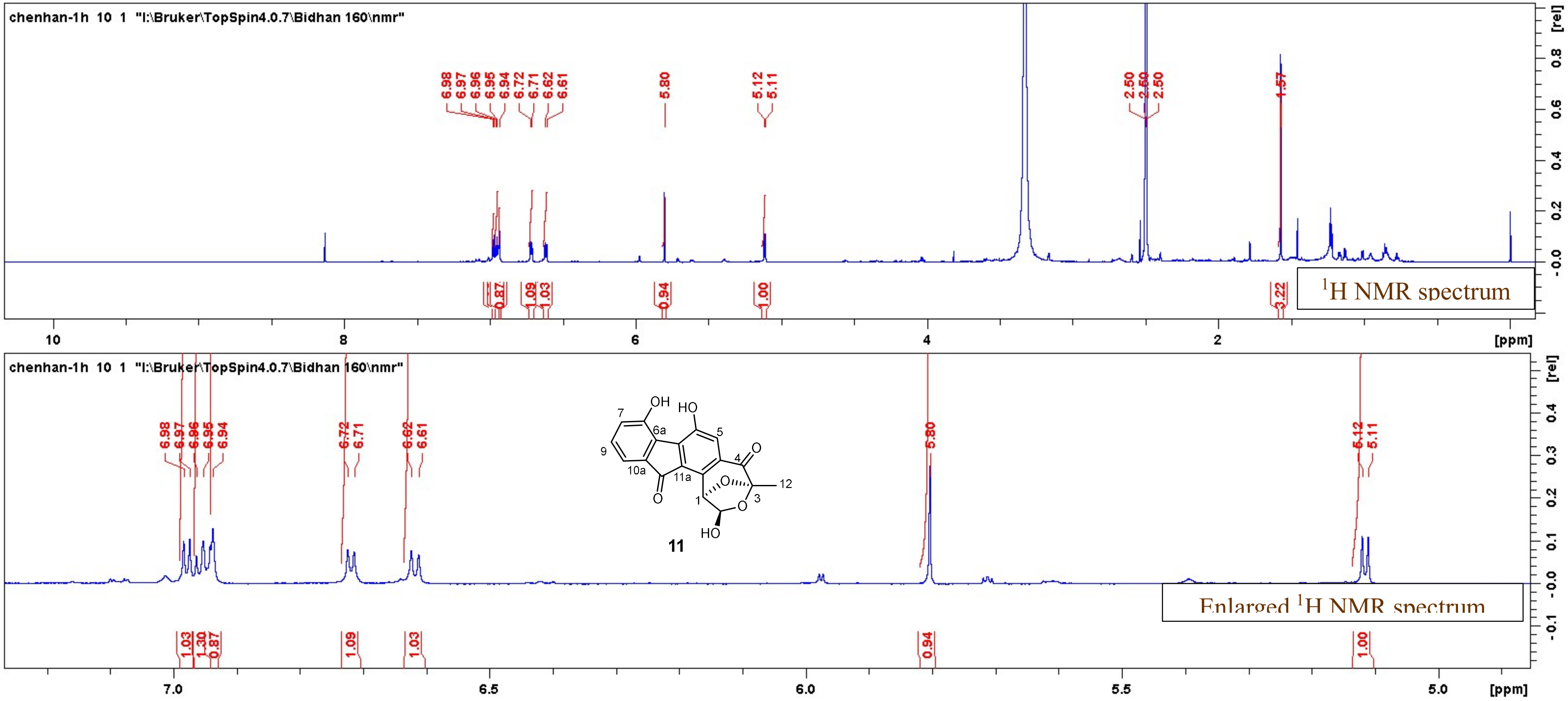

Supplementary Fig. 11 Spectroscopic data for 11. (**b**) The ^{1}H and enlarged ^{1}H NMR spectrum of **11** in DMSO-d_6.

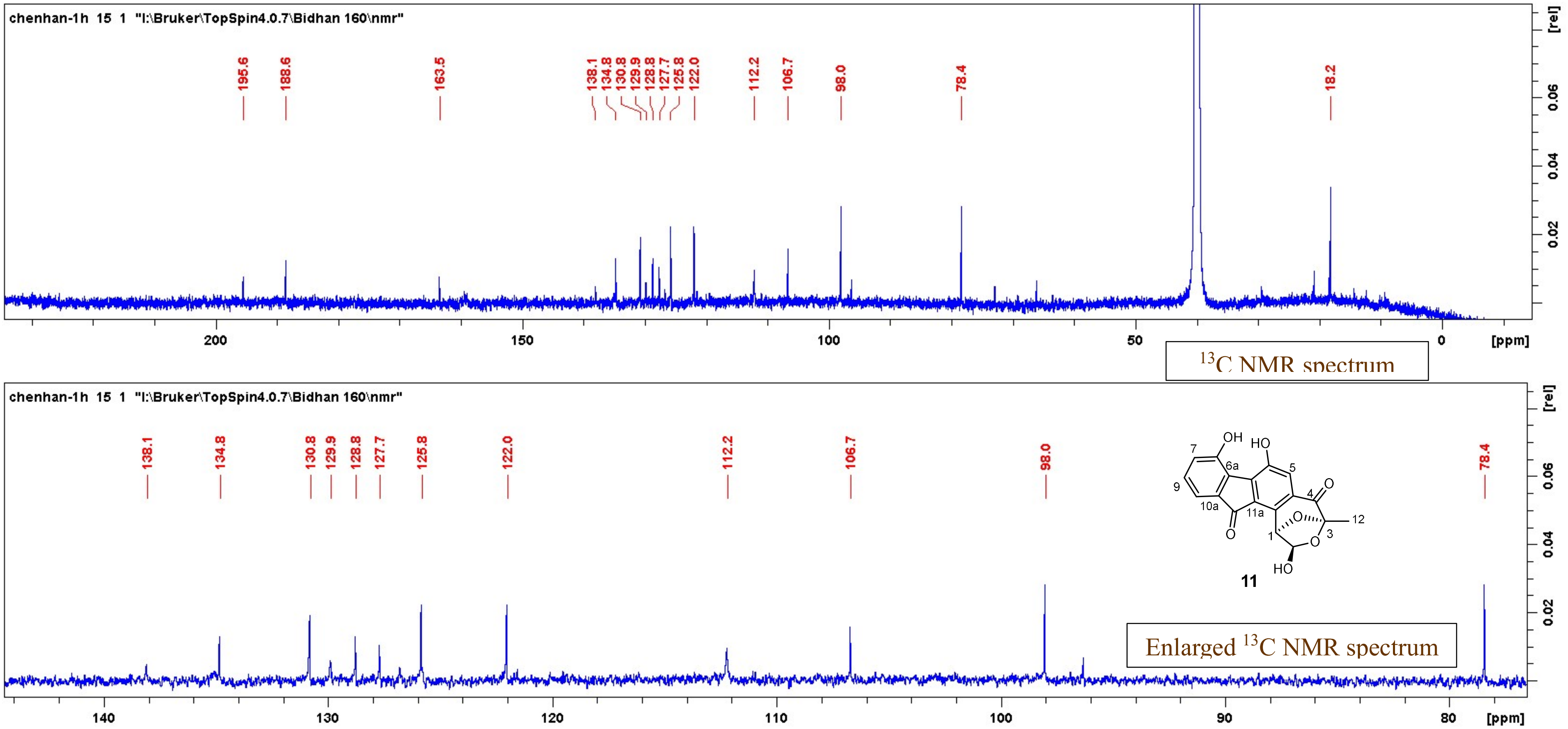

Supplementary Fig. 11 Spectroscopic data for 11. (**c**) The ^{13}C and enlarged ^{13}C NMR spectrum of **11** in DMSO-d_6.

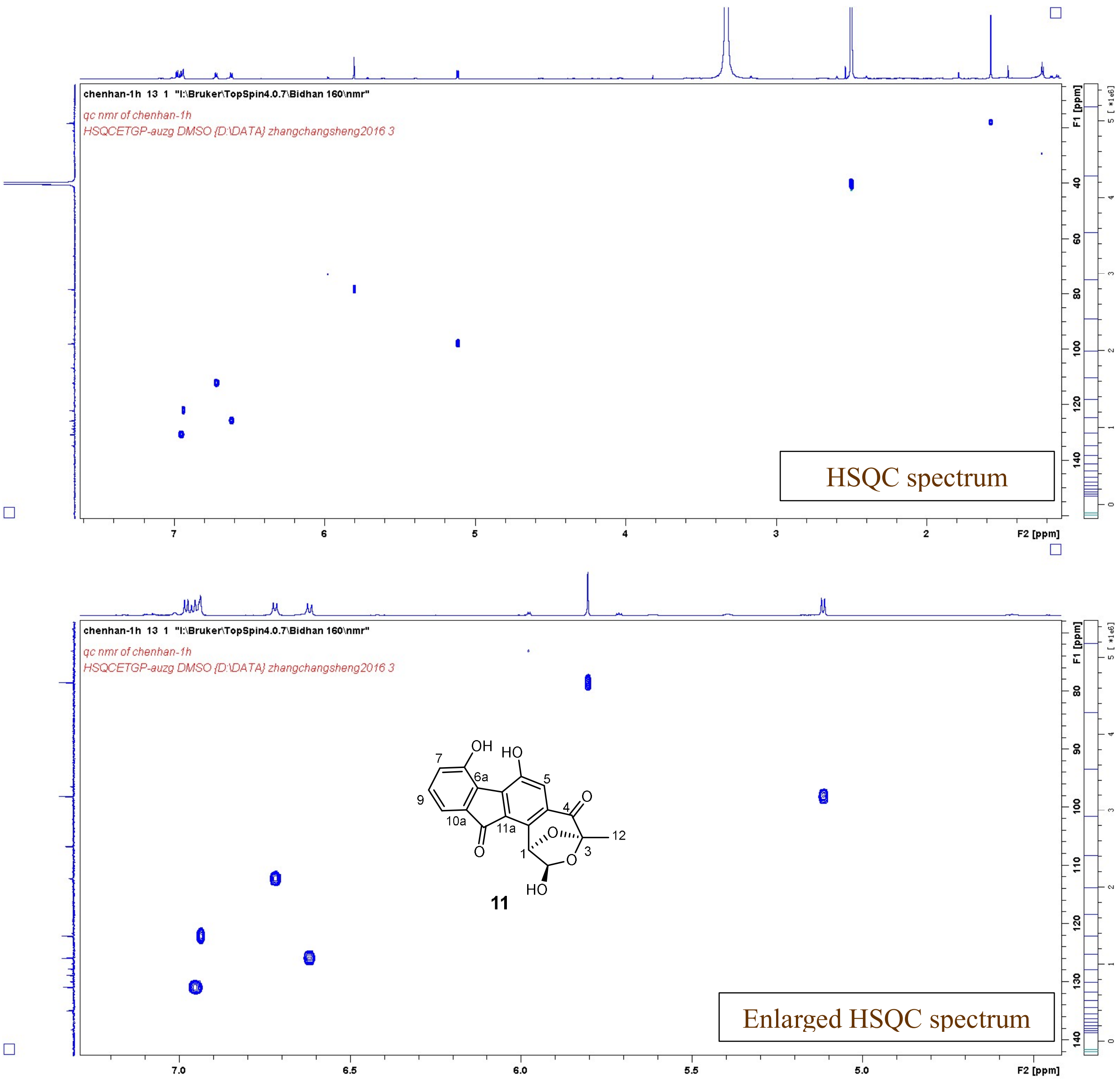

Supplementary Fig. 11 Spectroscopic data for 11. (d) The HSQC and enlarged HSQC spectrum of **11** in DMSO-d_6.

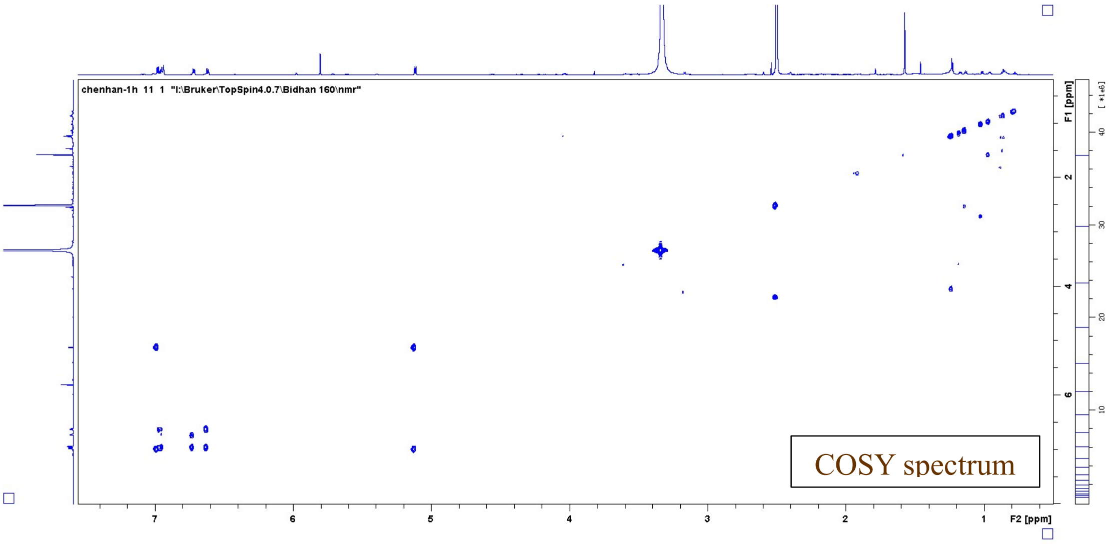

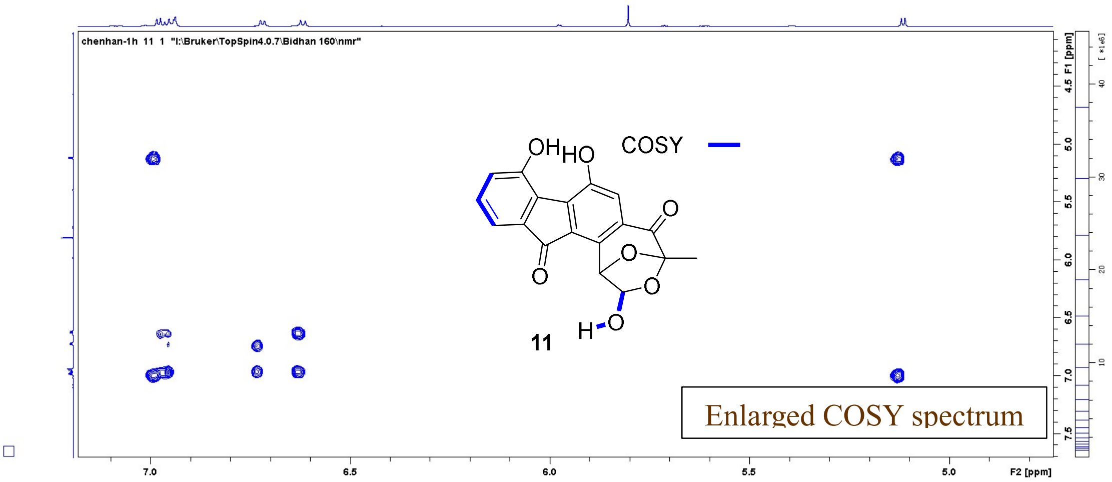

Supplementary Fig. 11 Spectroscopic data for 11. (**e**) The COSY and enlarged COSY spectrum of **11** in DMSO-d_6.

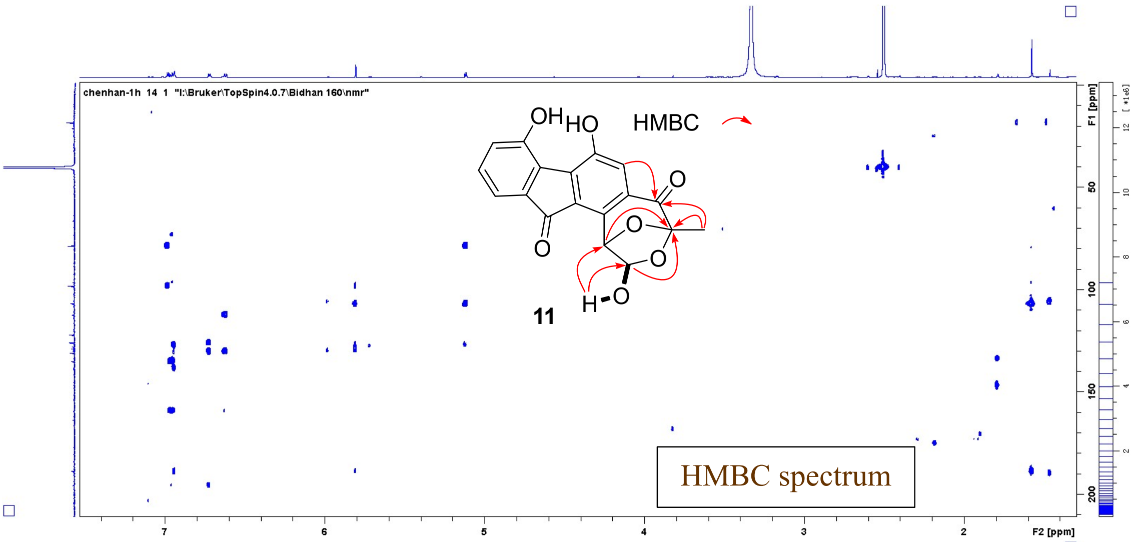

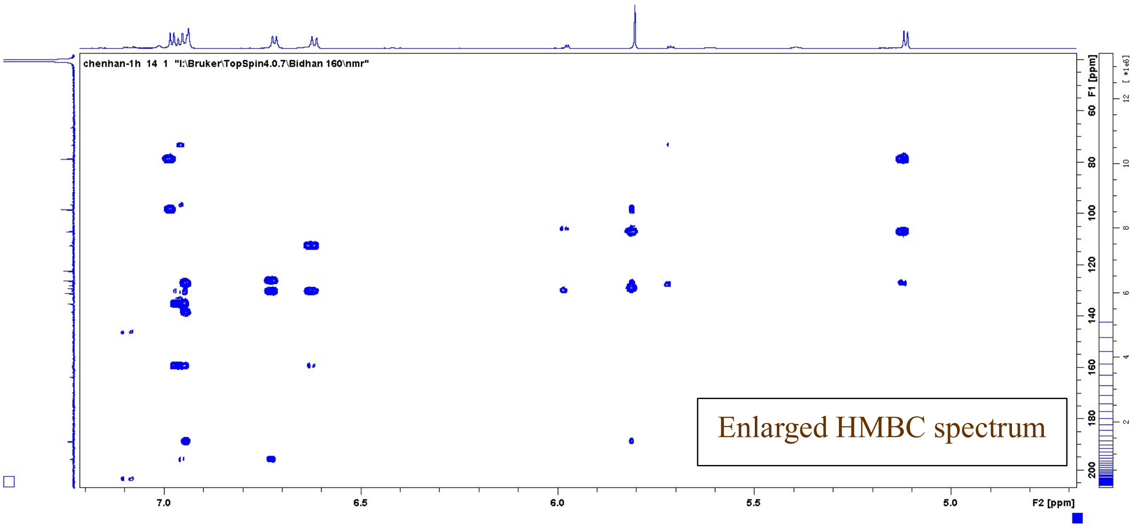

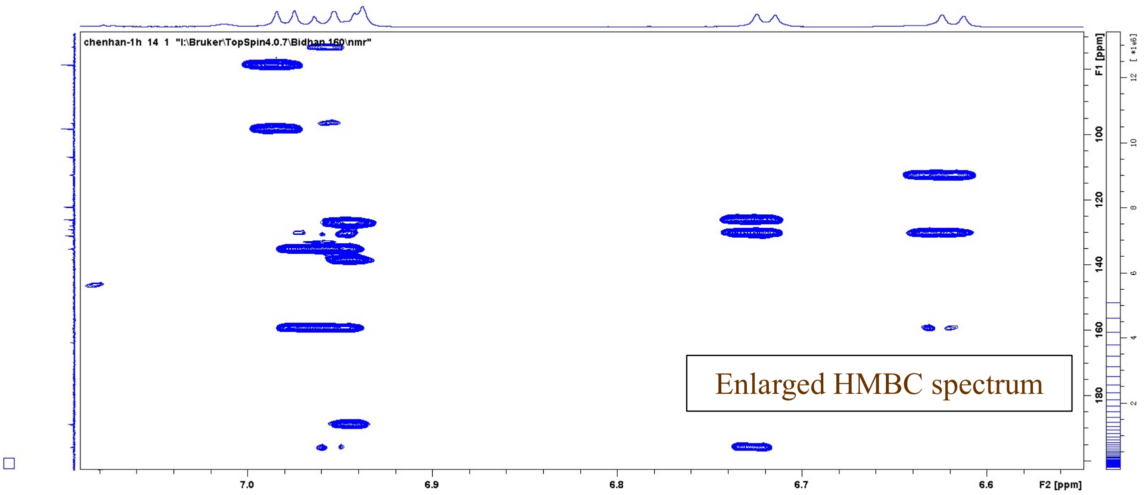

Supplementary Fig. 11 Spectroscopic data for 11. (**f**) The HMBC and enlarged HMBC spectrum of **11** in DMSO-d_6.

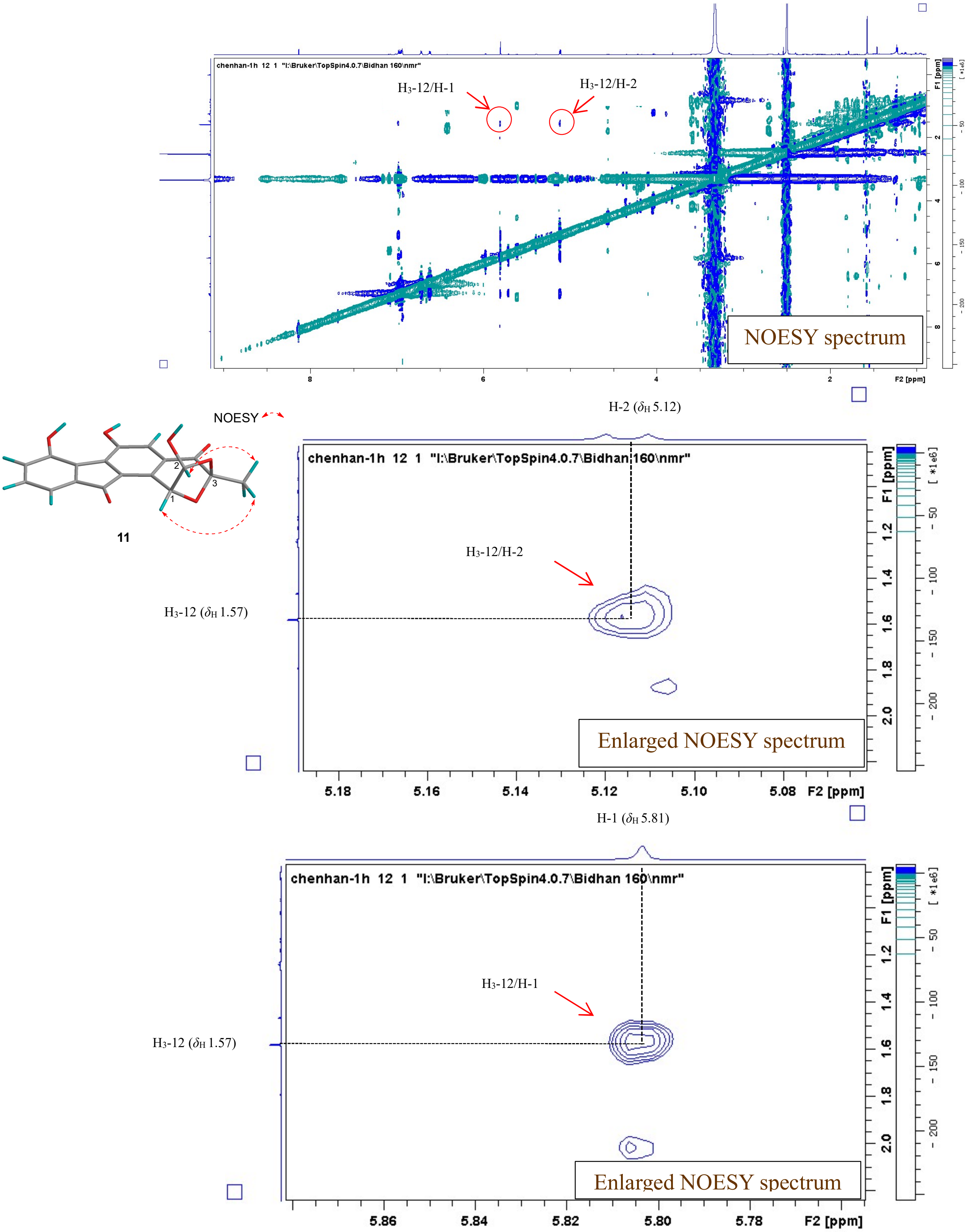

Supplementary Fig. 11 Spectroscopic data for 11. (**g**) The NOESY and enlarged NOESY spectrum of **11** in DMSO-d_6.

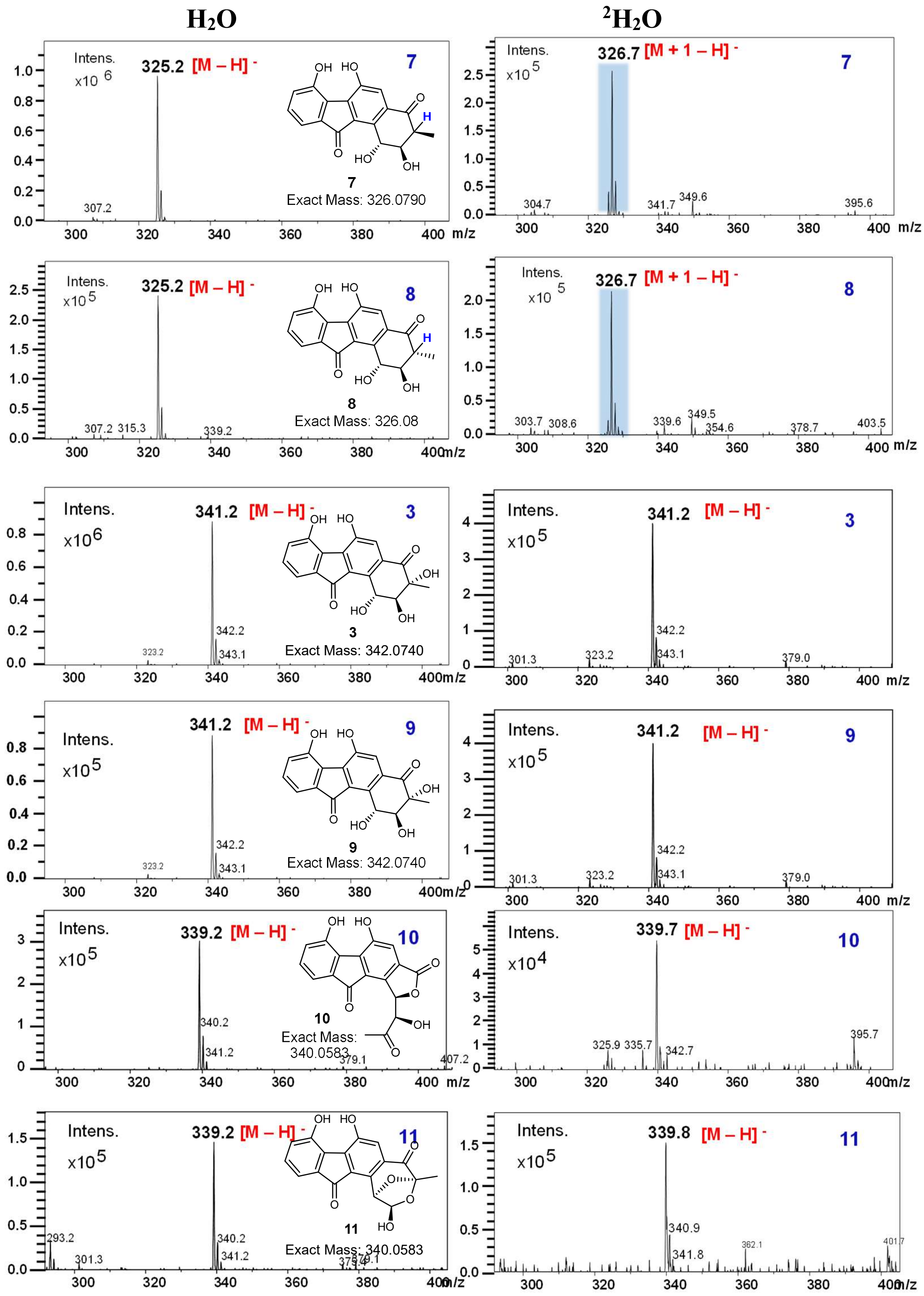

Supplementary Fig. 12. LC-MS analysis of reactions of 1 with FAD/NADH using PBS buffers prepared with H_2O (control) or ^2H_2O.

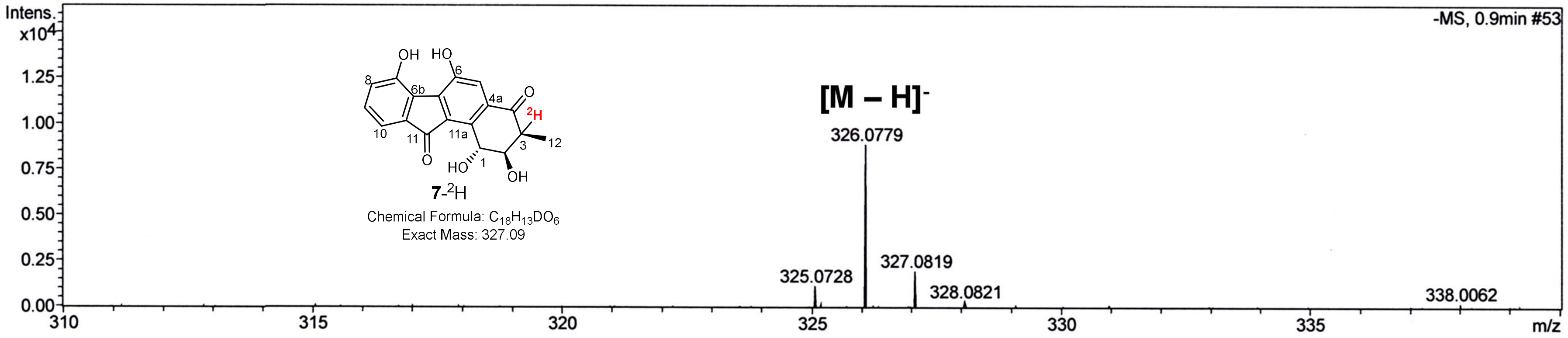

Supplementary Fig. 13. Spectroscopic data for 7-^{2}H. (**a**) HRESIMS spectrum.

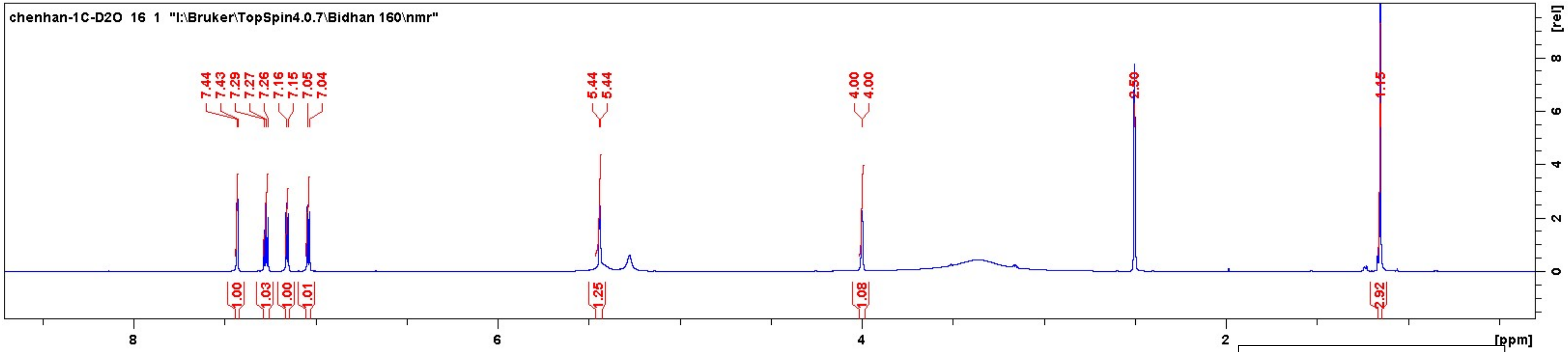

^{1}H NMR spectrum

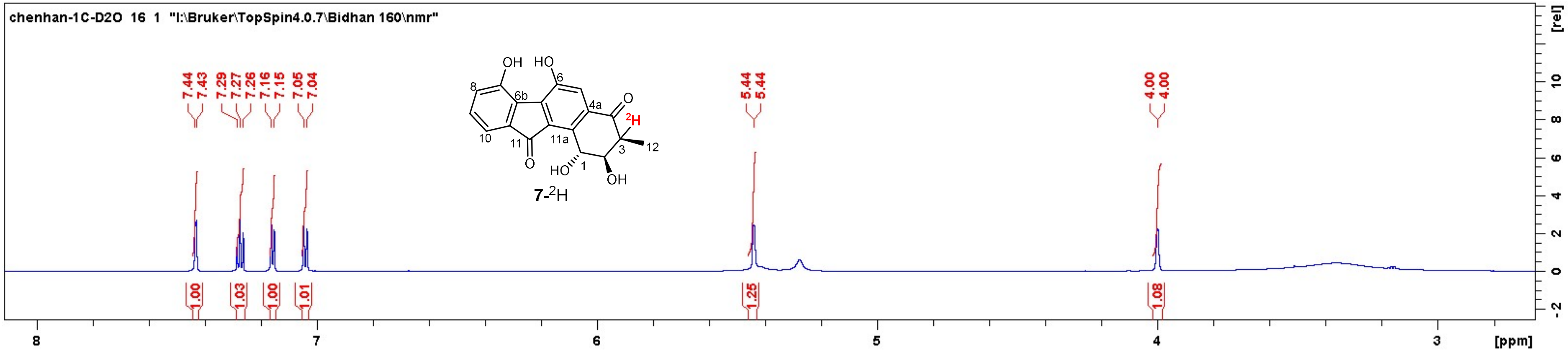

Enlarged ^{1}H NMR spectrum

Supplementary Fig. 13. Spectroscopic data for 7-^{2}H. (**b**) The ^{1}H and enlarged ^{1}H NMR spectrum of **7**-^{2}H in DMSO-d_6.

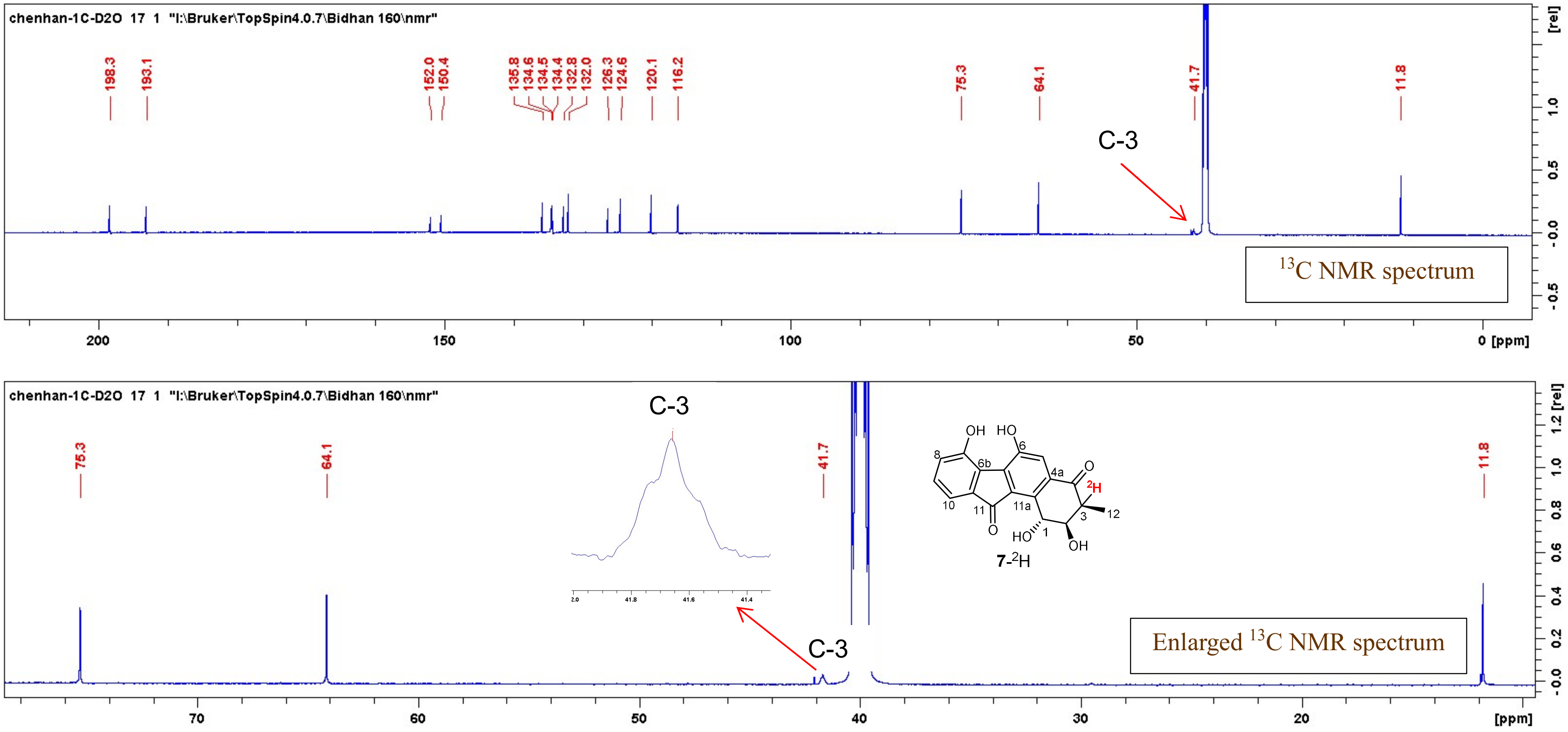

Supplementary Fig. 13. Spectroscopic data for 7-^{2}H. (**c**) The ^{13}C NMR and enlarged ^{13}C NMR spectrum of **7**-^{2}H in DMSO-*d*$_6$.

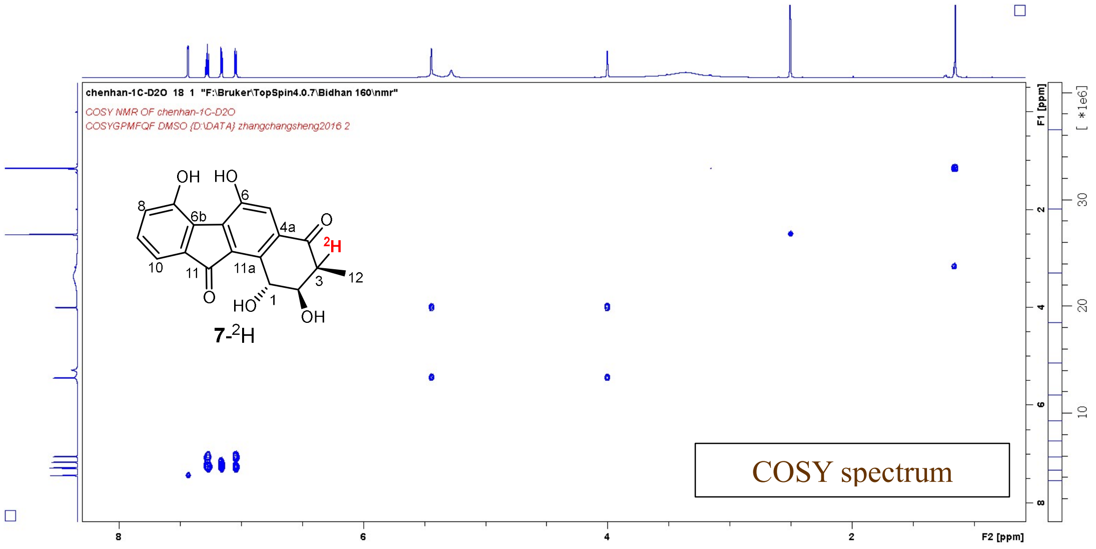

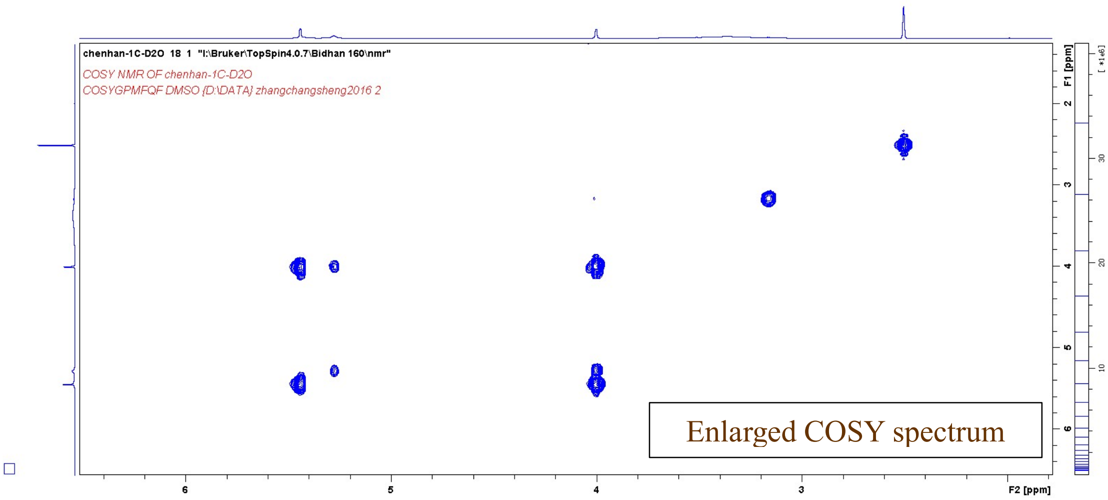

Supplementary Fig. 13. Spectroscopic data for 7-^{2}H. (**d**) The COSY and enlarged COSY spectrum of **7**-^{2}H in DMSO-d_6.

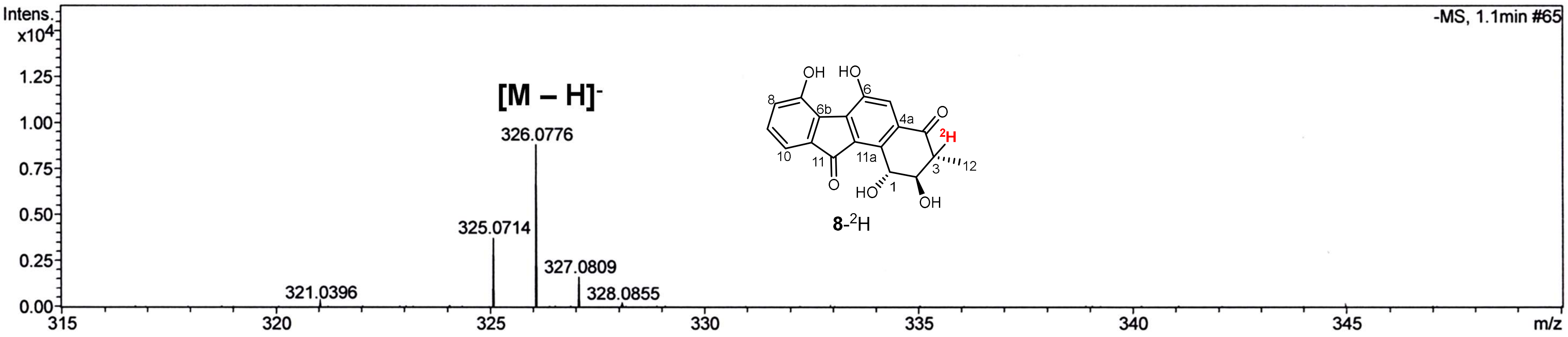

Supplementary Fig. 14. Spectroscopic data for 8-^{2}**H.** (**a**) HRESIMS spectrum.

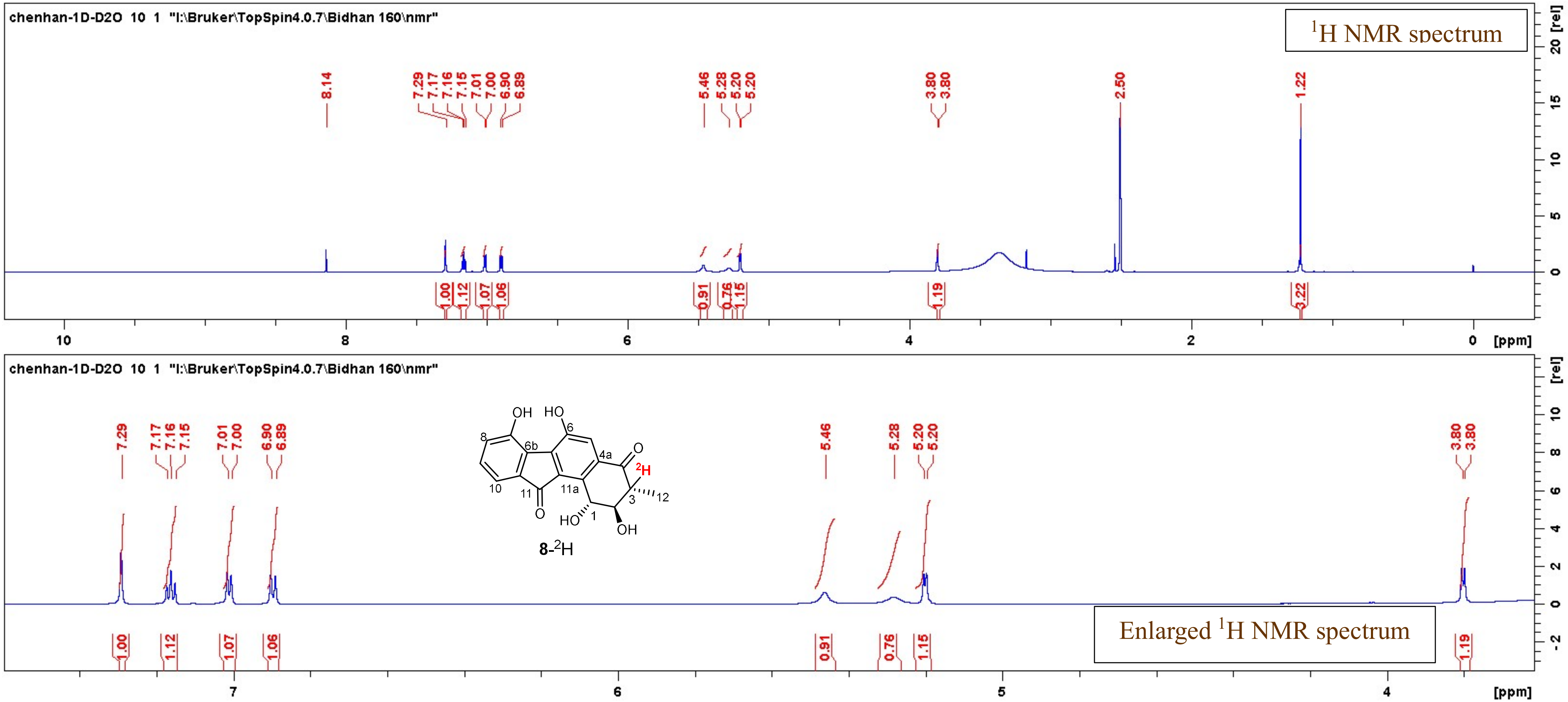

Supplementary Fig. 14. Spectroscopic data for 8-^{2}H. (**b**) The ^{1}H and enlarged ^{1}H NMR spectrum of **8**-^{2}H in DMSO-d_6.

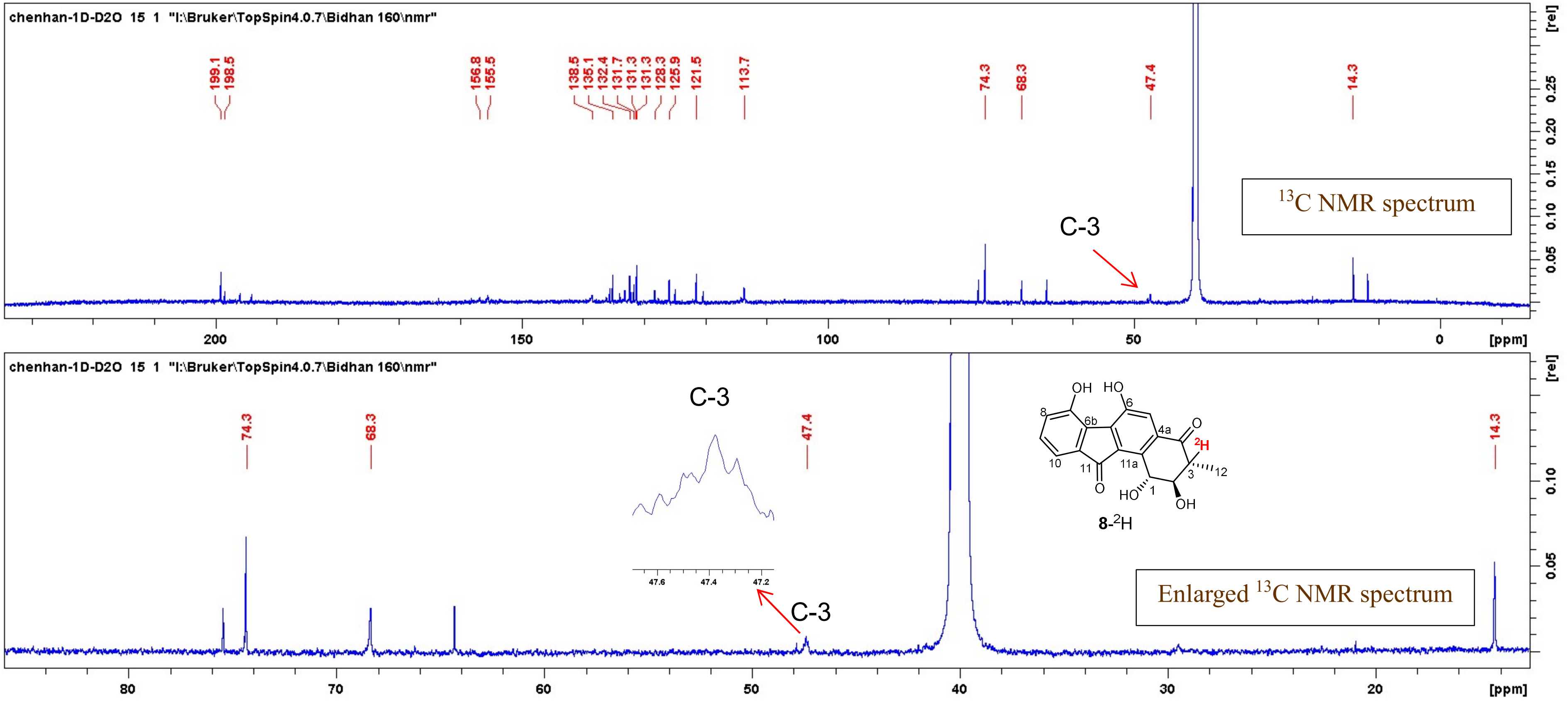

Supplementary Fig. 14. Spectroscopic data for 8-^{2}H. (**c**) The ^{13}C and enlarged ^{13}C NMR spectrum of **8**-^{2}H in DMSO-d_6.

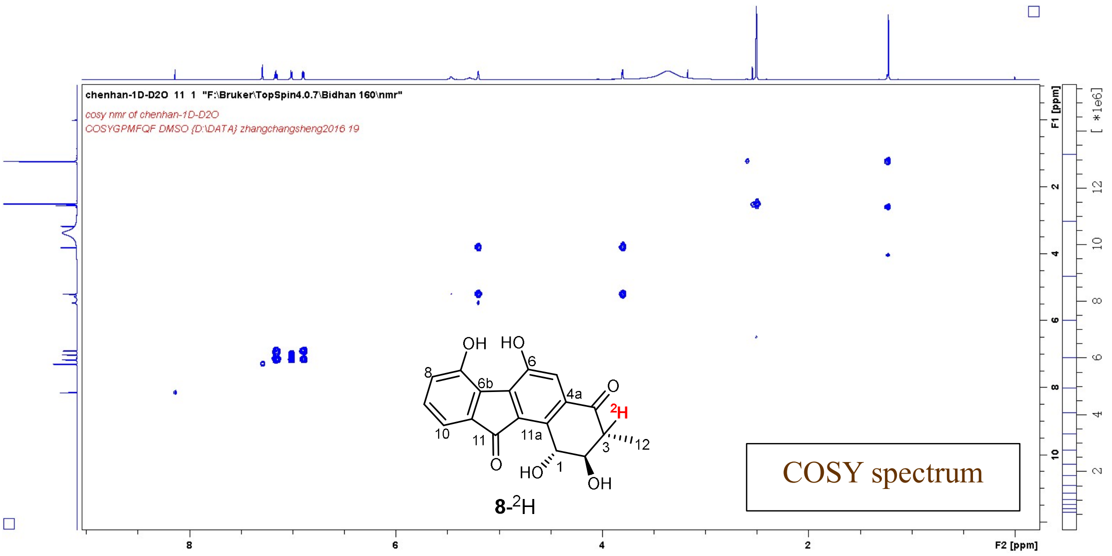

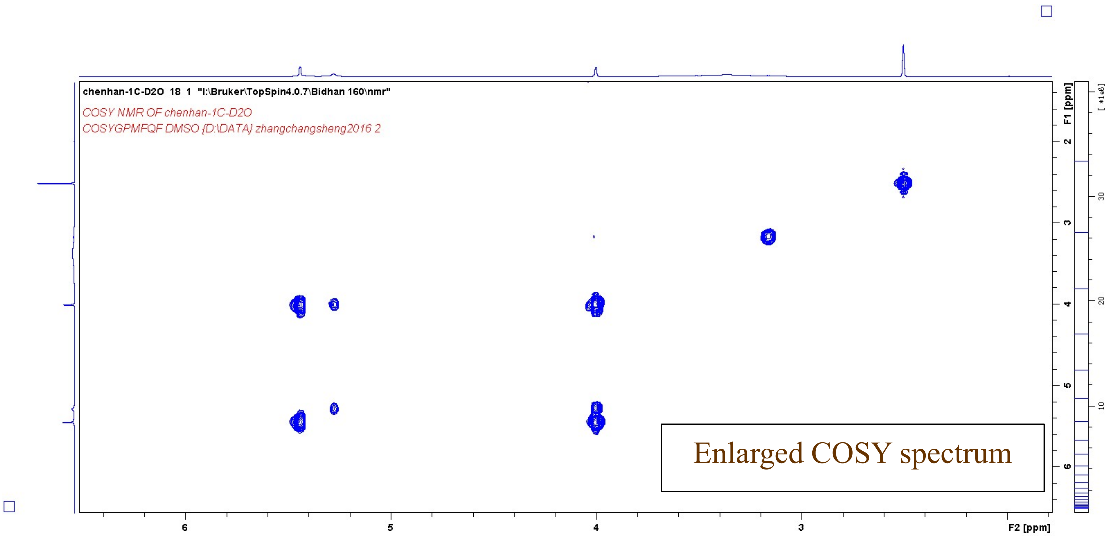

Supplementary Fig. 14. Spectroscopic data for 8-^{2}H. (**d**) The COSY and enlarged COSY spectrum of **8**-^{2}H in DMSO-d_6.

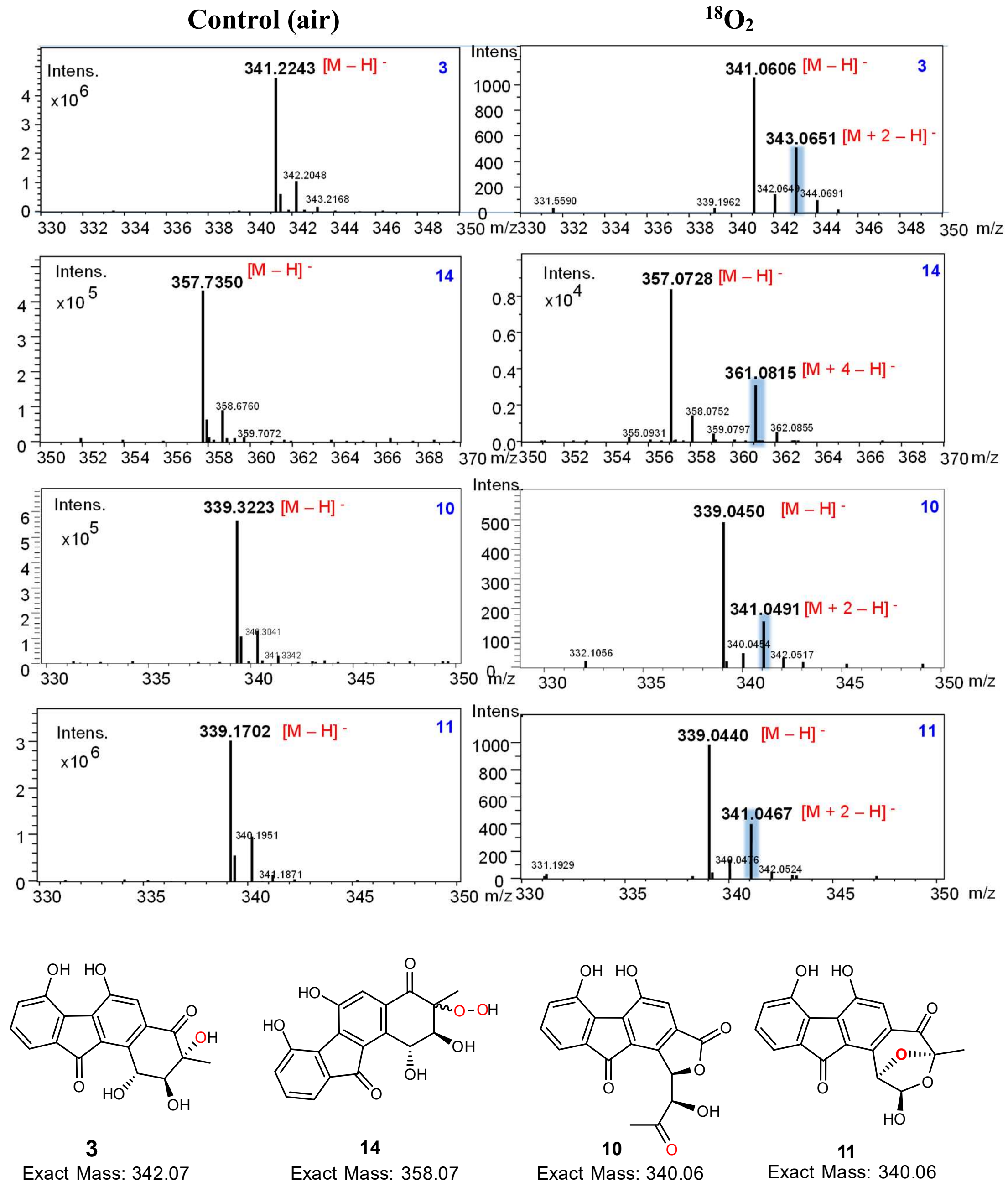

Supplementary Fig. 15. LC-HRMS analysis of reactions of FST C (1) with FAD/NADH under air (control) or under $^{18}O_2$.

(**a**) HRESIMS for **3**-^{18}O.

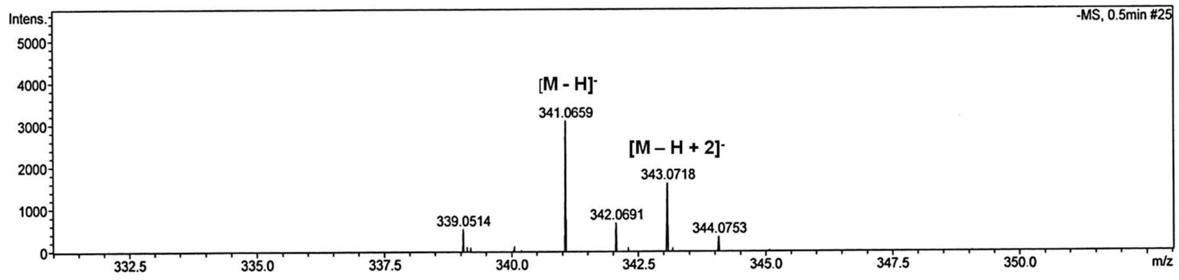

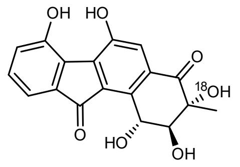

3 -^{18}O

Chemical Formula: $C_{18}H_{14}O_6{}^{18}O$

Exact Mass: 344.08

(**b**) HRESIMS for **9**-^{18}O.

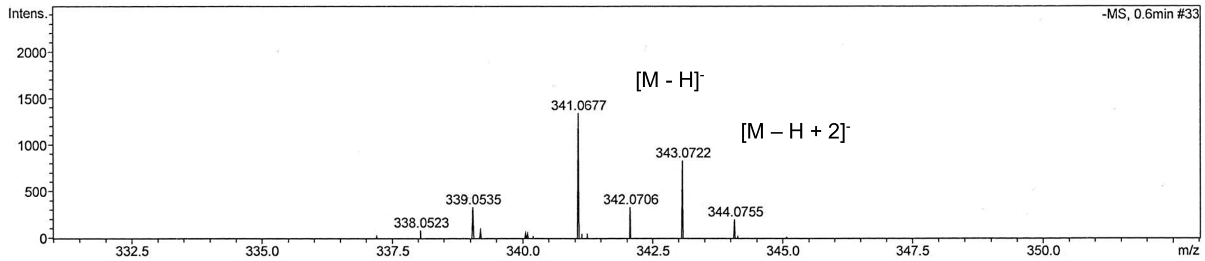

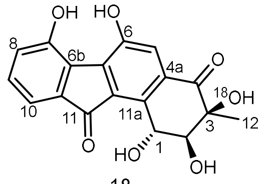

9-^{18}O

Chemical Formula: $C_{18}H_{14}O_6{}^{18}O$

Exact Mass: 344.08

Supplementary Fig. 16. HRESIMS spectroscopic data for 3-^{18}O and 9-^{18}O.

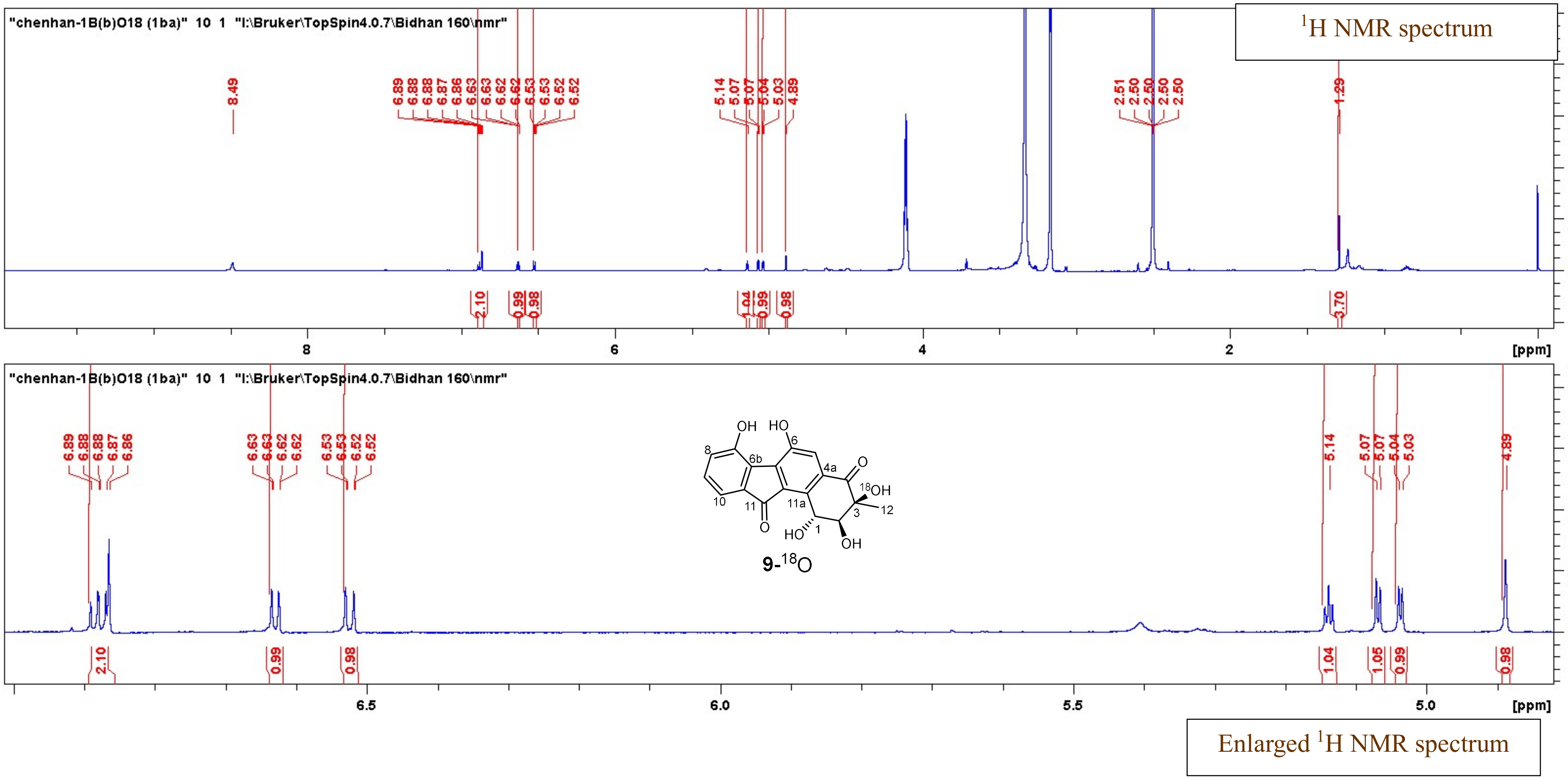

Supplementary Fig. 17. Spectroscopic data of 9-^{18}O. (**a**) The ^{1}H and enlarged ^{1}H NMR spectrum of **9-**^{18}O in DMSO-d_6.

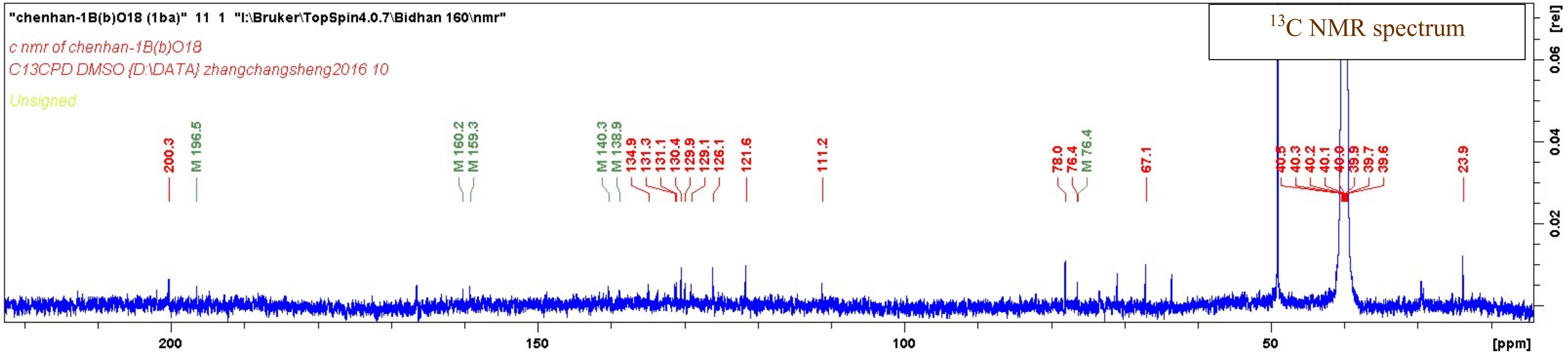

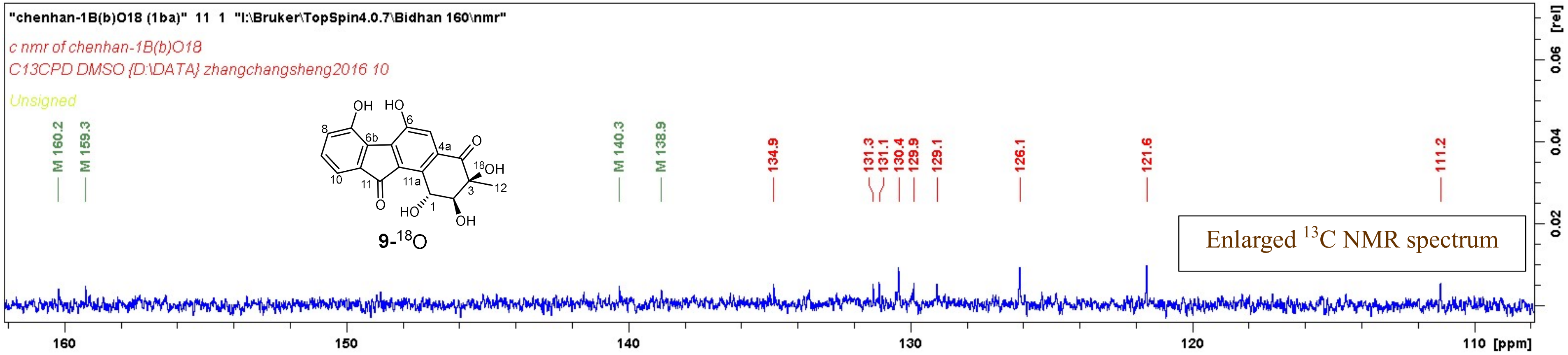

Supplementary Fig. 17. Spectroscopic data of 9-^{18}O. (**b**) The ^{13}C and enlarged ^{13}C NMR spectrum of **9**-^{18}O in DMSO-d_6.

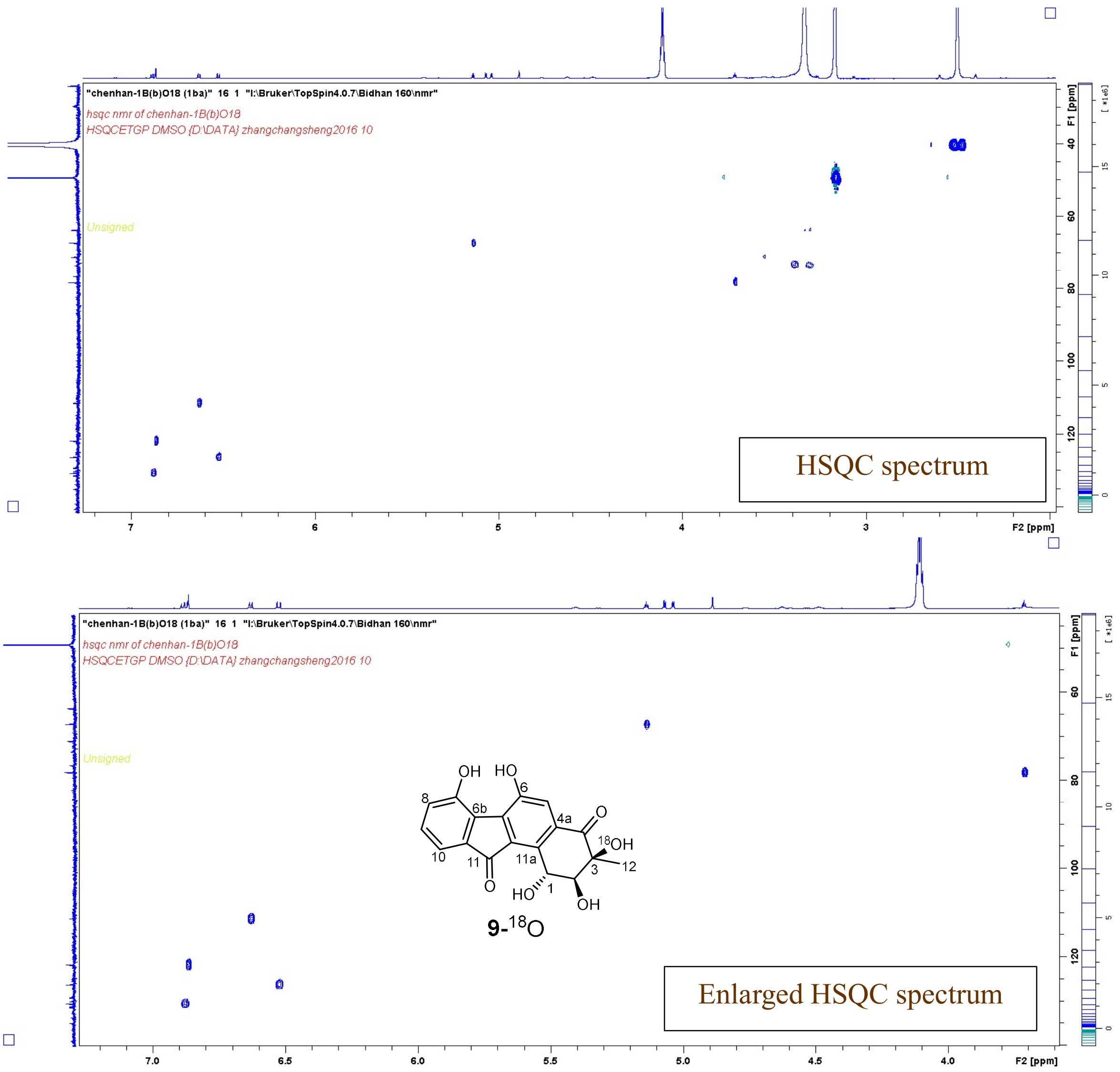

Supplementary Fig. 17. Spectroscopic data of 9-^{18}O. (**c**) The HSQC and enlarged HSQC spectrum of **9**-^{18}O in DMSO-d_6.

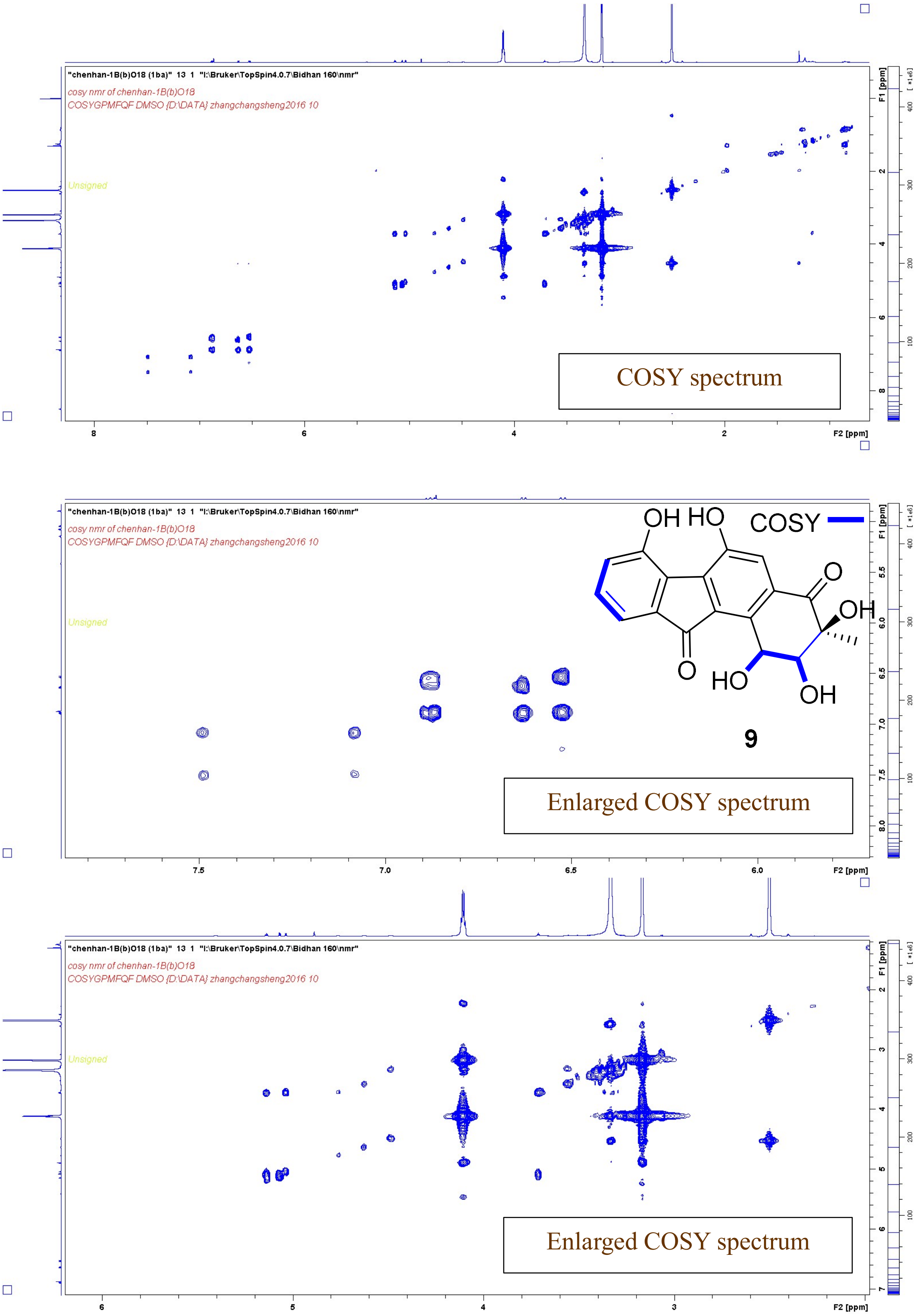

Supplementary Fig. 17. Spectroscopic data of 9-^{18}O. (**d**) The COSY and enlarged COSY spectrum of **9**-^{18}O in DMSO-d_6.

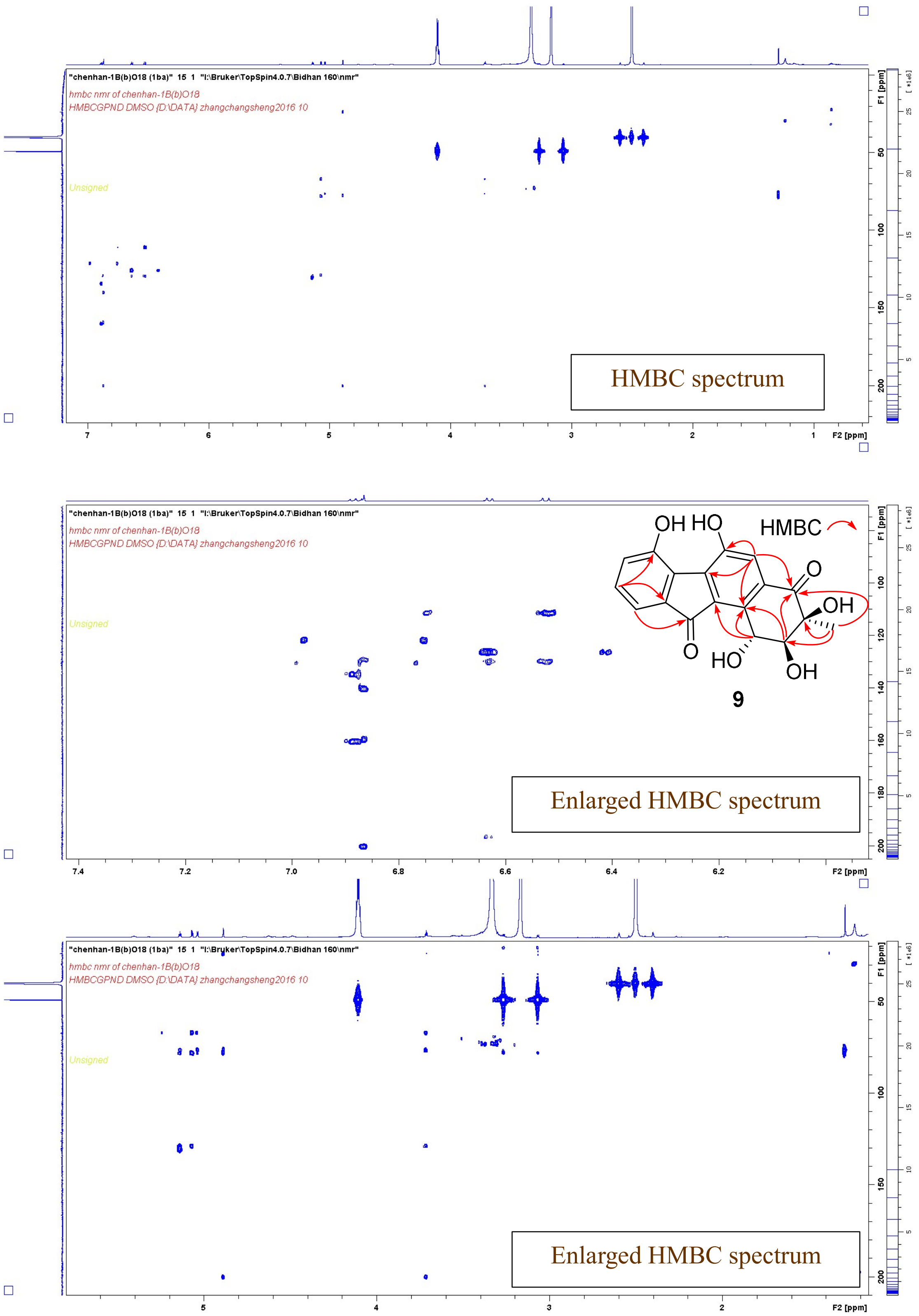

Supplementary Fig. 17. Spectroscopic data of 9-^{18}O. (e) The HMBC and enlarged HMBC spectrum of **9**-^{18}O in DMSO-d_6.

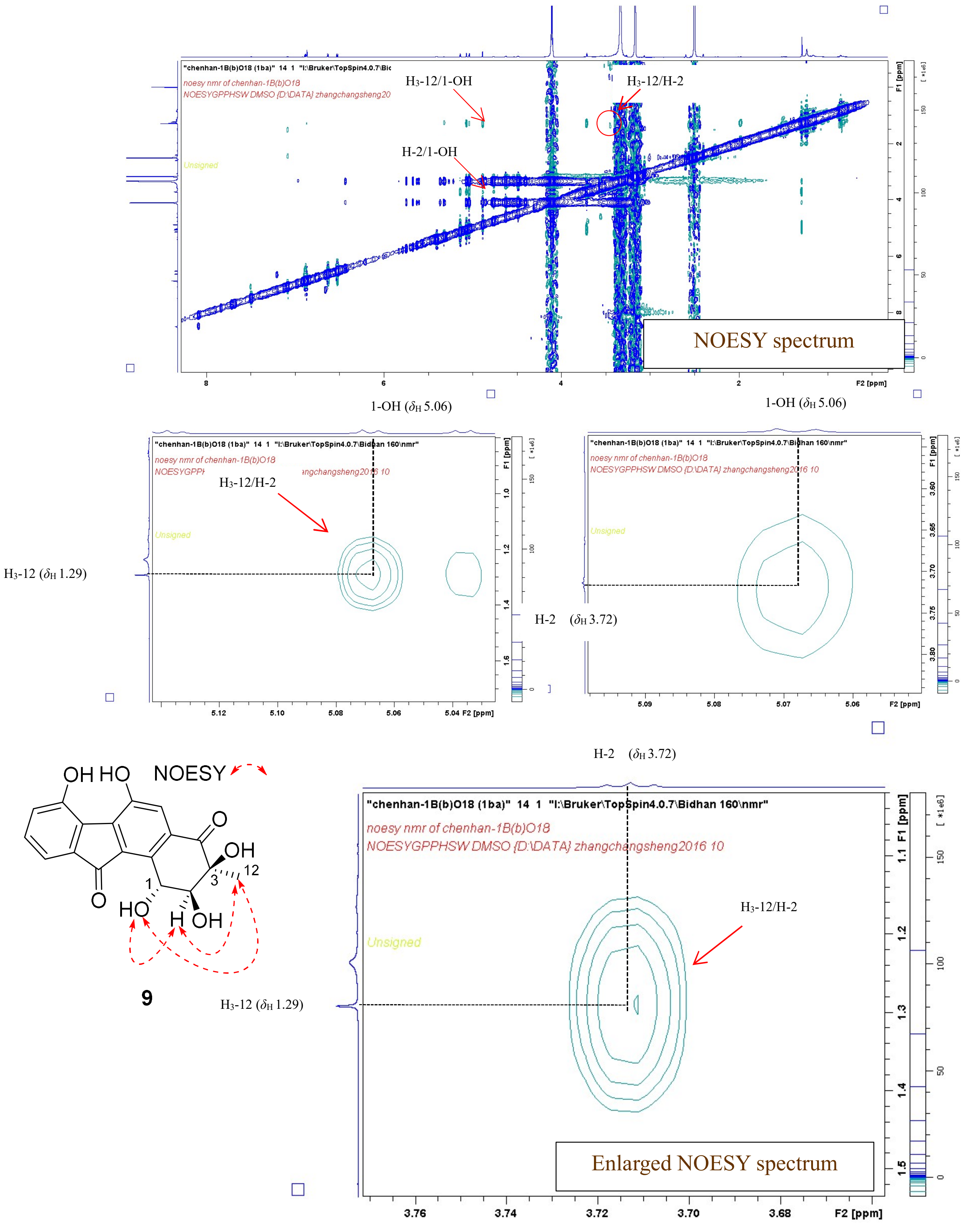

Supplementary Fig. 17. Spectroscopic data of 9-^{18}O. (**f**) The NOESY and enlarged NOESY spectrum of **9**-^{18}O in DMSO-d_6.

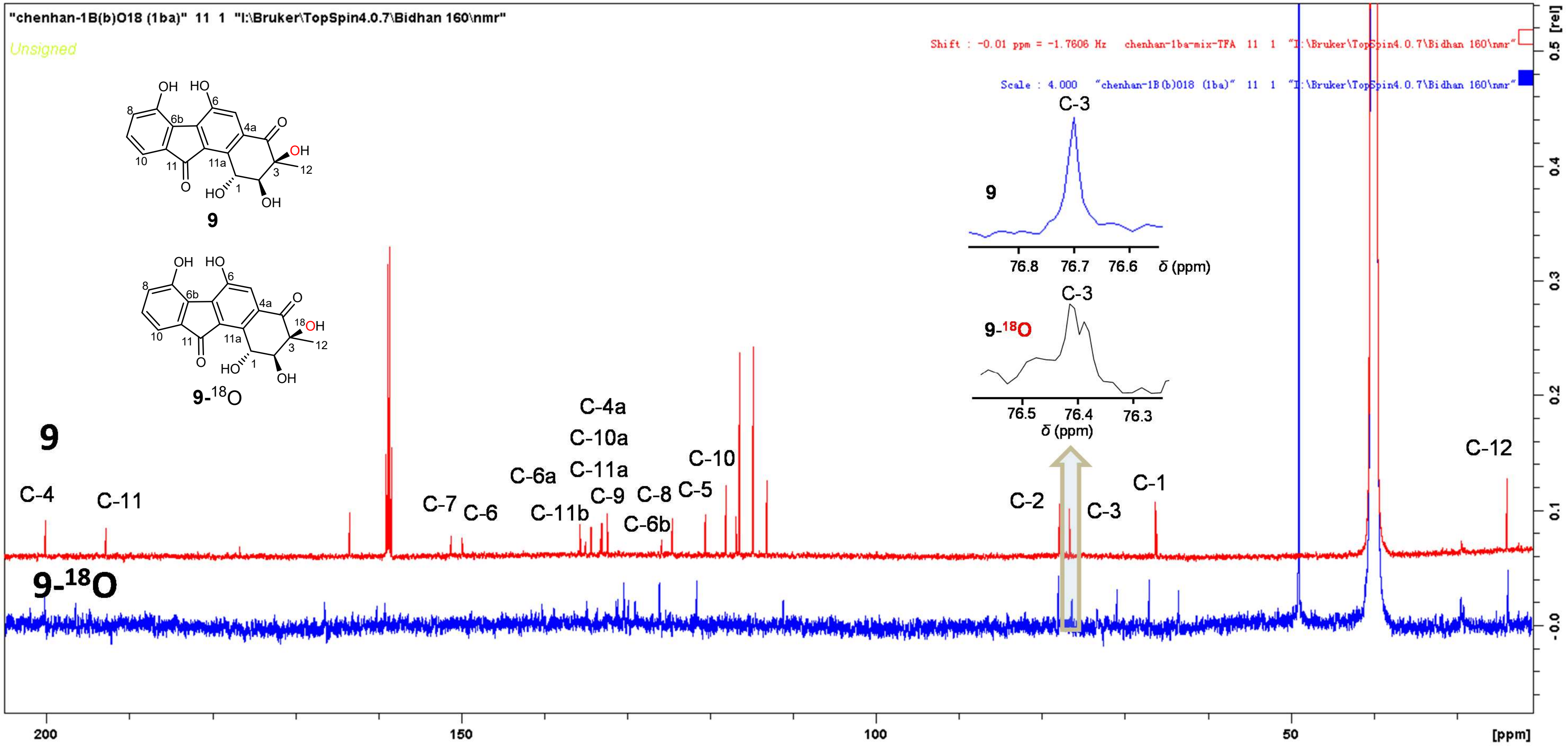

Supplementary Fig. 18. Comparison the ^{13}C NMR spectroscopic data of 9-^{18}O in DMSO-d_6 and 9 in DMSO-d_6:trifluoroacetic acid (TFA)-d_1 (100:1).

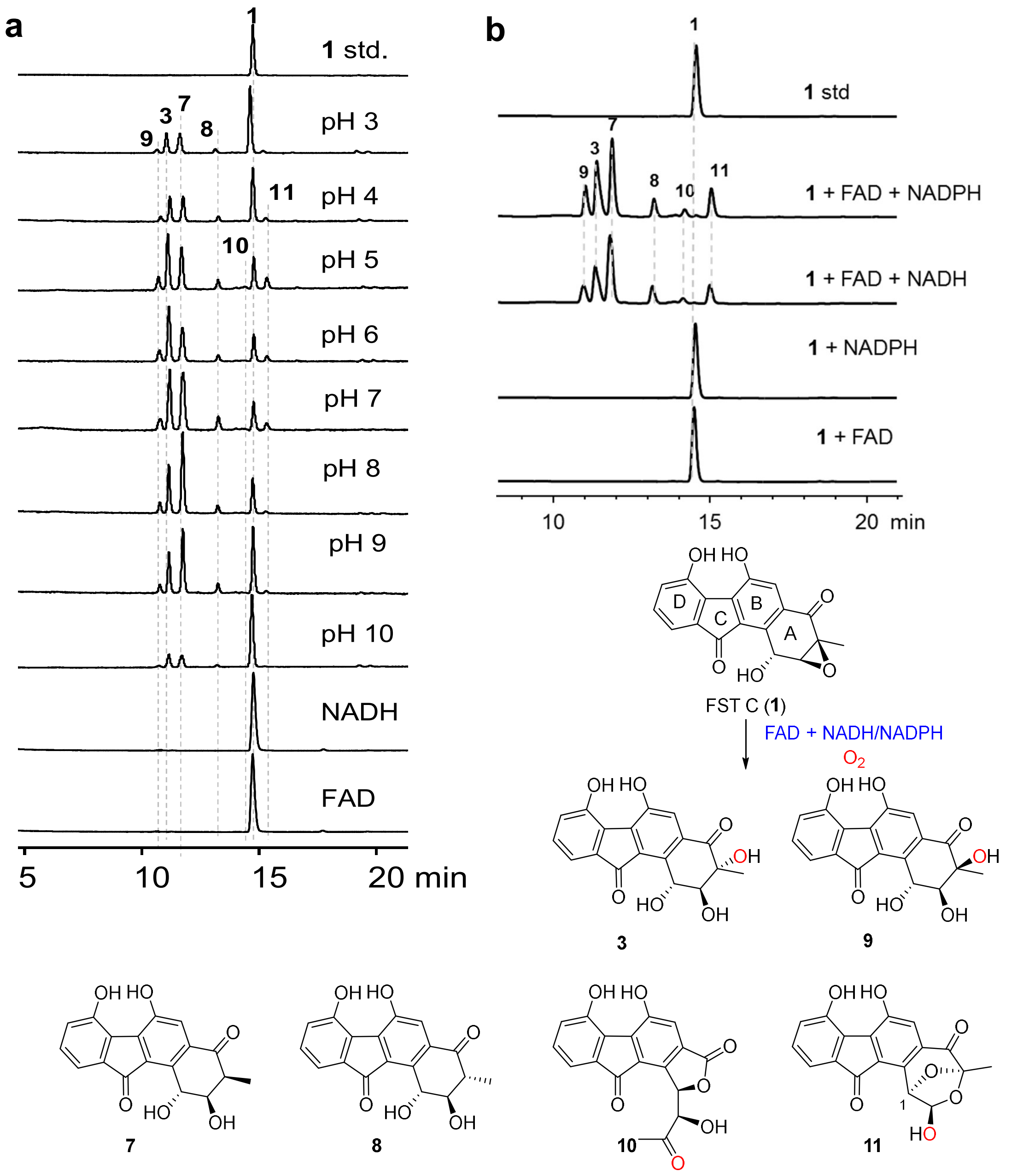

Supplementary Fig. 19. HPLC analysis of reactions of 1 and FAD/NAD(P)H in buffers of varying pH values. (**a**) A standard assay contains 100 µM **1**, 100 µM FAD and 10 mM NADH in buffers from pH 3 to pH 10. The buffers include citric acid/Na_2HPO_4 buffer (50 mM, pH 3 to 6); PBS buffer (50 mM, pH 7); boric acid/borax buffer (50 mM, pH 8 and 9); borax/NaOH buffer (50 mM, pH 10). The reaction mixtures were incubated at 30 °C for 2 h. (**b**) the assay contains 100 µM **1**, 100 µM FAD and 10 mM NADH or NADPH in PBS buffer (50 mM, pH 7) with incubation at 30 °C for 2hrs. HPLC analysis was performed on the Agilent 1260 Infinity series instrument (Agilent Technologies Inc., USA) using a polar column (Comixsep®, P/N FMG-BPF5-EONU, Polar BiPFP 5u, 250 × 4.6 mm, China) with UV detection at 304 nm.

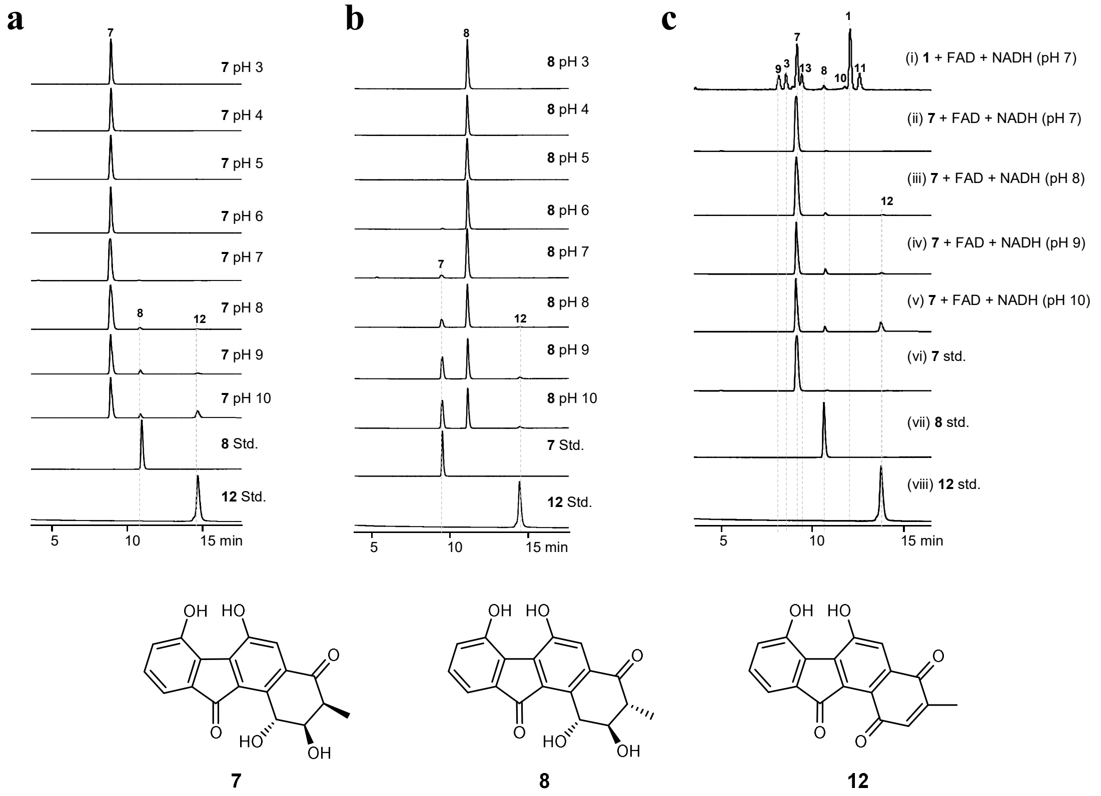

Supplementary Fig. 20. The spontaneous conversion of 7 to 8 (or 8 to 7) in buffers with varying pH values. (**a**) HPLC analysis of spontaneous conversion of **7** to **8** and **12**. Compound **7** was incubated in 50 mM buffers with varying pHs from 3 to 10 for 30 min at 30 °C. (**b**) HPLC analysis of spontaneous conversion of **8** to **7** and **12**. Compound **8** was incubated in 50 mM buffers with varying pHs from 3 to 10 for 30 min at 30 °C. (**c**) HPLC analysis of **7** with FAD/NADH. (i) The assay contains 100 μM **1**, 10 μM FAD and 2 mM NADH at 30 °C for 20 min (pH 7); (ii) **7** + 10 μM FAD and 2 mM NADH (pH 7); (iii) **7** + FAD and NADH (pH 8); (iv) **7** + FAD and NADH (pH 9); (v) **7** + FAD and NADH (pH 10); (vi) **7** std.; (vii) **8** std.; (viii) **12** std. The reaction mixtures of (i–v) were incubated at 30 °C for 30 min and the HPLC analysis were performed using polar column.

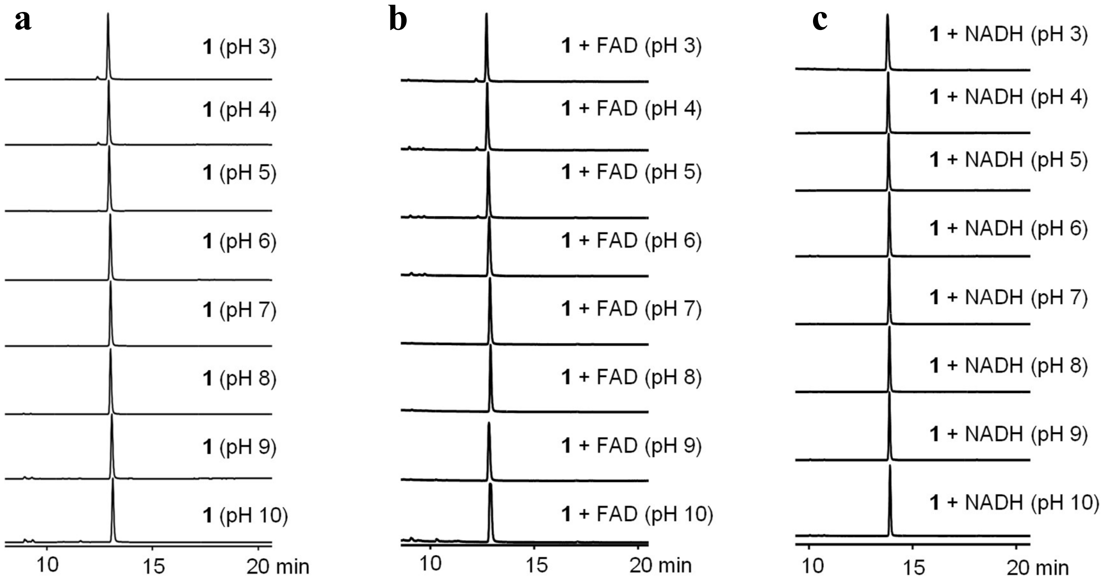

Supplementary Fig. 21. The stability of FST C (1) under varying conditions. (**a**) HPLC analysis of 100μM FST C (**1**) in 50 mM buffers with varying pHs from **3** to **10**. (**b**) HPLC analysis of 100μM **1** + 100 μM FAD in in 50 mM buffers with varying pH from 3 to 10. (**c**) HPLC analysis of 100μM **1** + 10 mM NADH in 50 mM buffers with varying pH from 3 to 10. The buffers include citric acid/Na_2HPO_4 buffer (50 mM, pH 3 to 6); PBS buffer (50 mM, pH 7); boric acid/borax buffer (50 mM, pH 8 and 9); borax/NaOH buffer (50 mM, pH 10). The mixtures were incubated at 30 °C for 2 h.

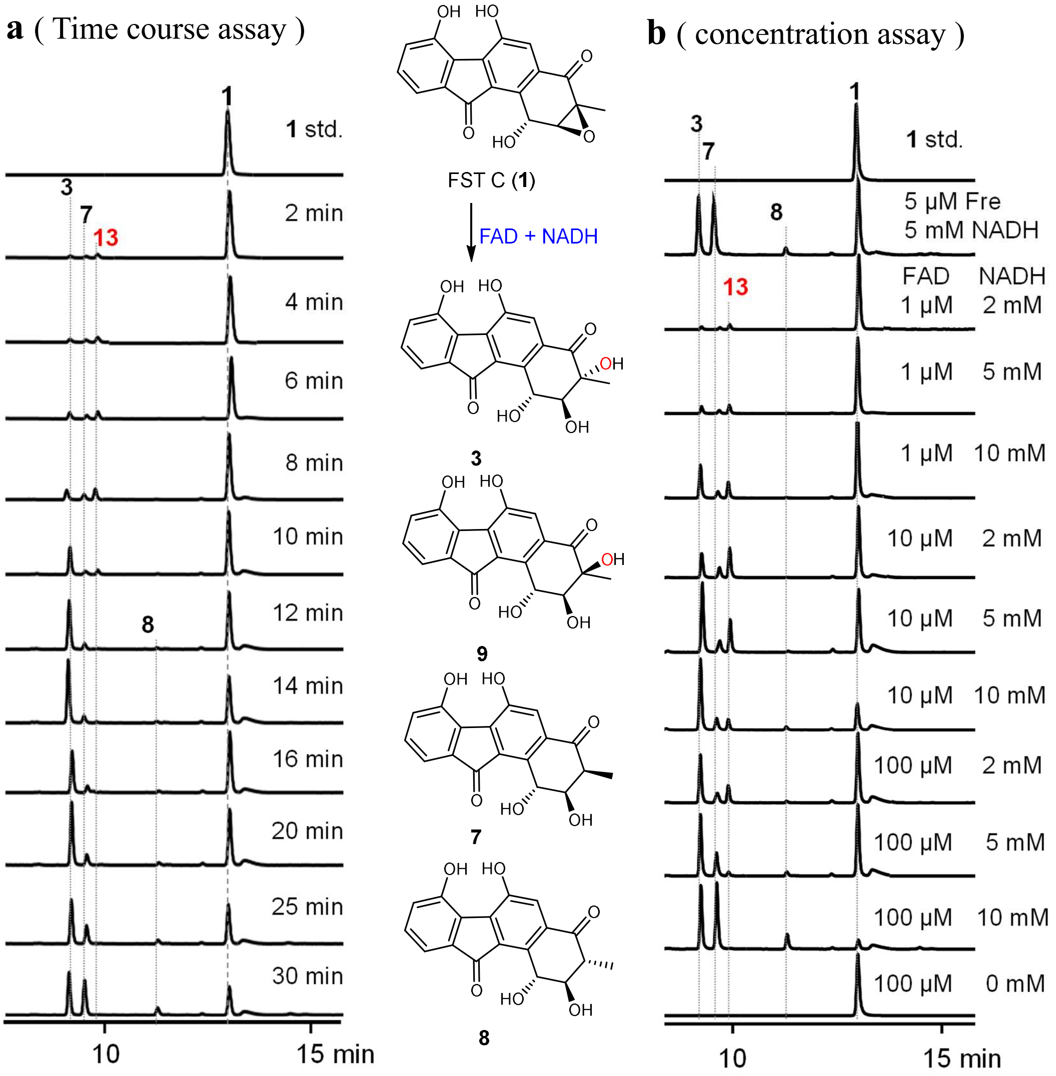

Supplementary Fig. 22. HPLC analysis for a time course assay of 1 and FAD/NADH and reactions of 1 with varying concentrations of FAD and NADH. (**a**) HPLC analysis of a time course assay of 100 µM **1** in 50 mM PBS buffer (pH 7) with 100 µM FAD and 10 mM NADH. The reactions at 30 °C were sampled at 2, 4, 6, 8, 10, 12, 14, 16, 20, 25, and 30 min. The instable intermediate **13** was observed between 2 to 10 min. (**b**) HPLC analysis of the reactions of 100 µM **1** in 50 mM PBS buffer (pH 7) with varying concentrations of FAD (1/ 10 / 100 µM) and NADH (2 / 5 / 10 mM) at 30 °C for 30 min. The best production of **13** was observed with 10 µM FAD and 2 mM NADH. HPLC analysis was performed on the Agilent 1260 Infinity series instrument (Agilent Technologies Inc., USA) using a reversed phase C18 column (Kinetex® 5µm C18 100 Å, LC Column 150 × 4.6 mm, Phenomenex, USA) with UV detection at 304 nm.

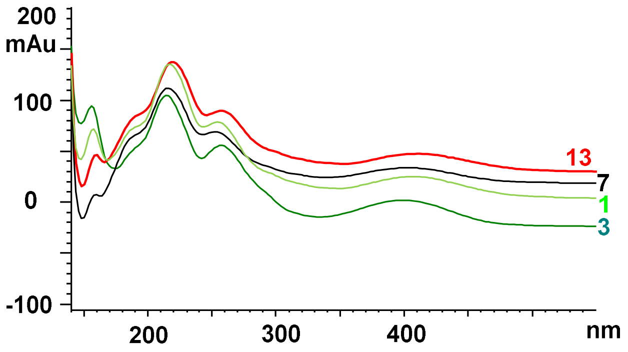

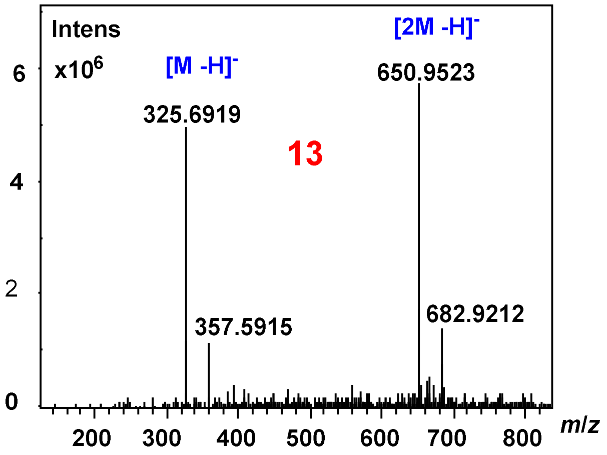

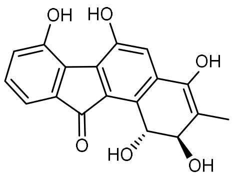

13

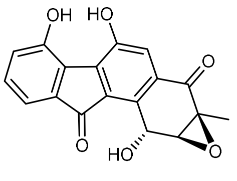

1

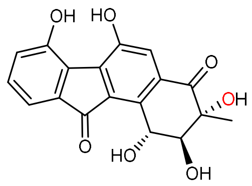

3

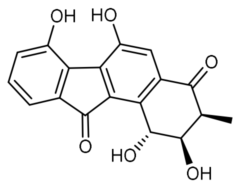

7

Supplementary Fig. 23. The comparison of UV spectra of 13 and FSTs and LC-HRMS analysis of 13.

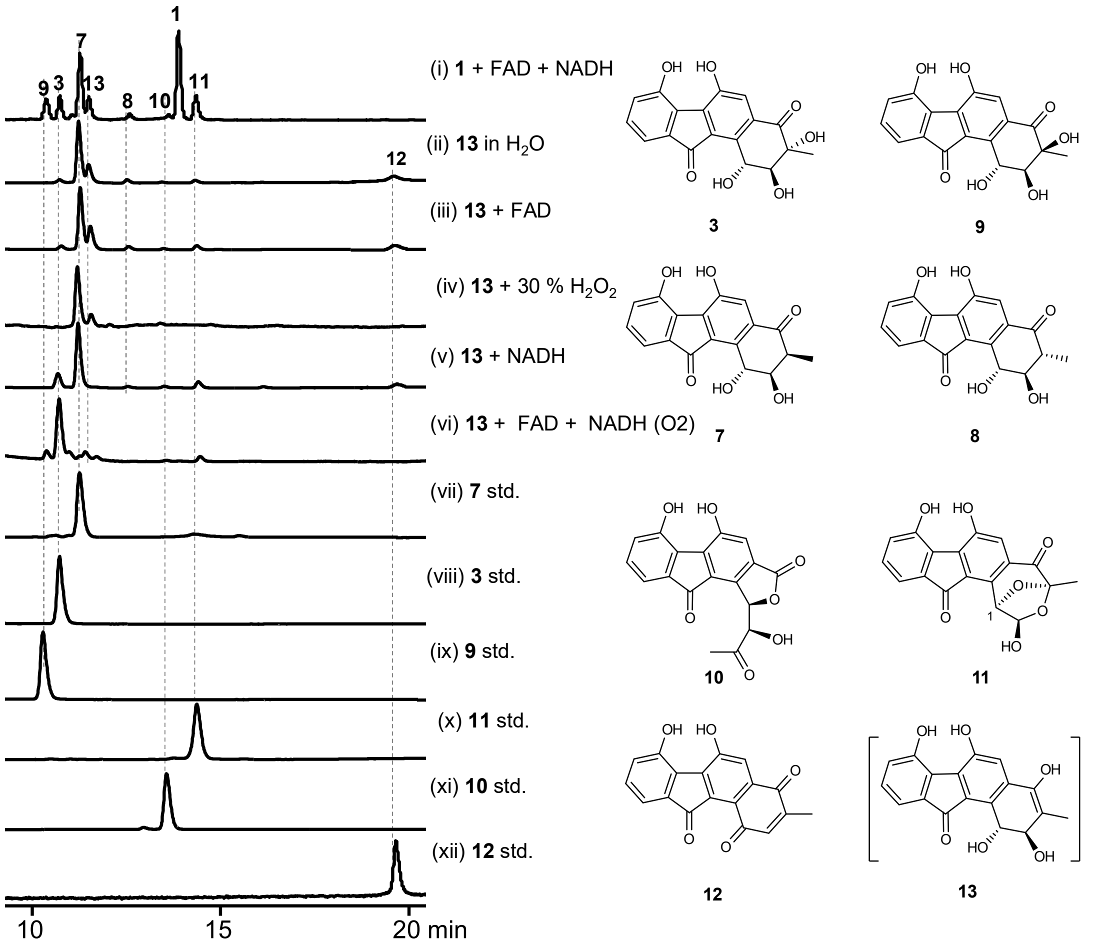

Supplementary Fig. 24. HPLC analysis of reactions with 13. (i) The assay contains 100 μM **1**, 10 μM FAD and 2 mM NADH at 30 °C for 10 min; (ii) **13** (collected from i) in H_2O; (iii) **13** + 10 μM FAD; (iv) **13** + 30 % H_2O_2; (v) **13** + 2 mM NADH; (vi) **13** + 10 μM FAD and 2 mM NADH; (vii) **7** std.; (viii) **3** std.; (ix) **9** std.; (x) **10** std.; (xi) **11** std.; (xii) **12** std. The reaction mixtures of (ii–vi) were incubated at 30 °C for 30 min and the HPLC analysis were performed using a polar column with UV detection at 304 nm.

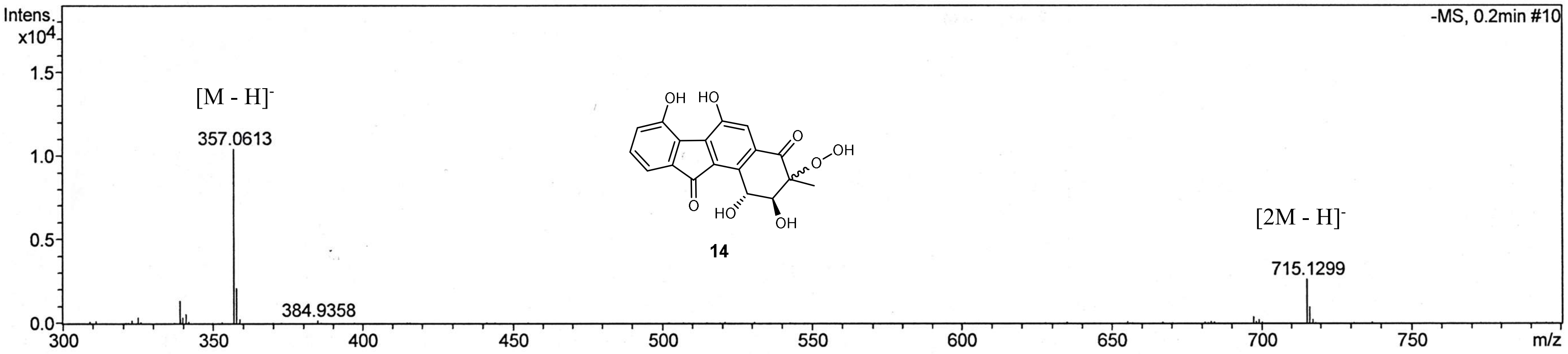

Meas. m/z	#	Ion Formula	Score	m/z	err [ppm]	err [mDa]	mSigma	rdb	e¯ Conf	N-Rule
357.0613	1	C18H13O8	100.00	357.0616	0.8	0.3	3.8	12.5	even	ok
715.1299	1	C36H27O16	100.00	715.1305	-0.8	-0.5	8.5	23.5	even	ok

Supplementary Fig. 25. Spectroscopic data for 14. (**a**) HRESIMS spectrum

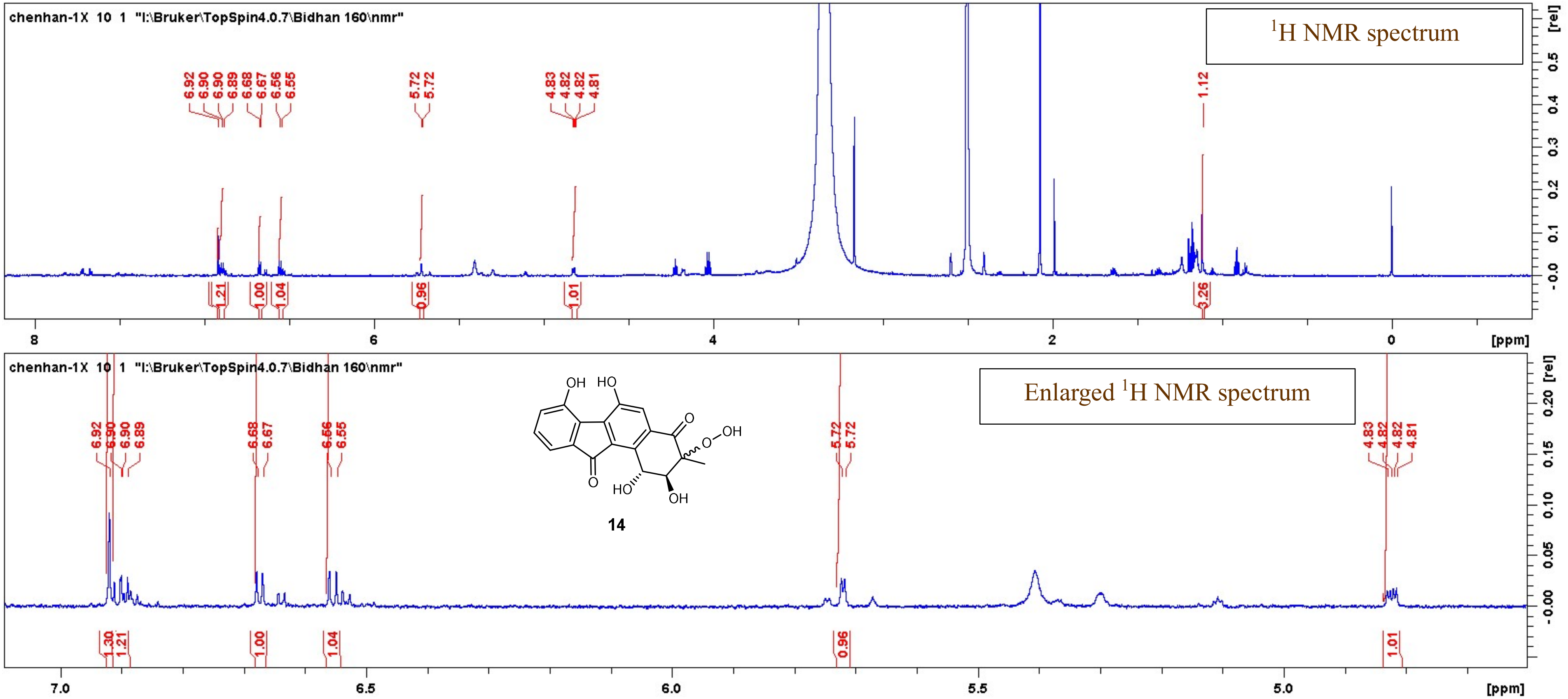

Supplementary Fig.25. Spectroscopic data for 14. (**b**) The ^{1}H and enlarged ^{1}H NMR spectrum of **14** in DMSO-d_6.

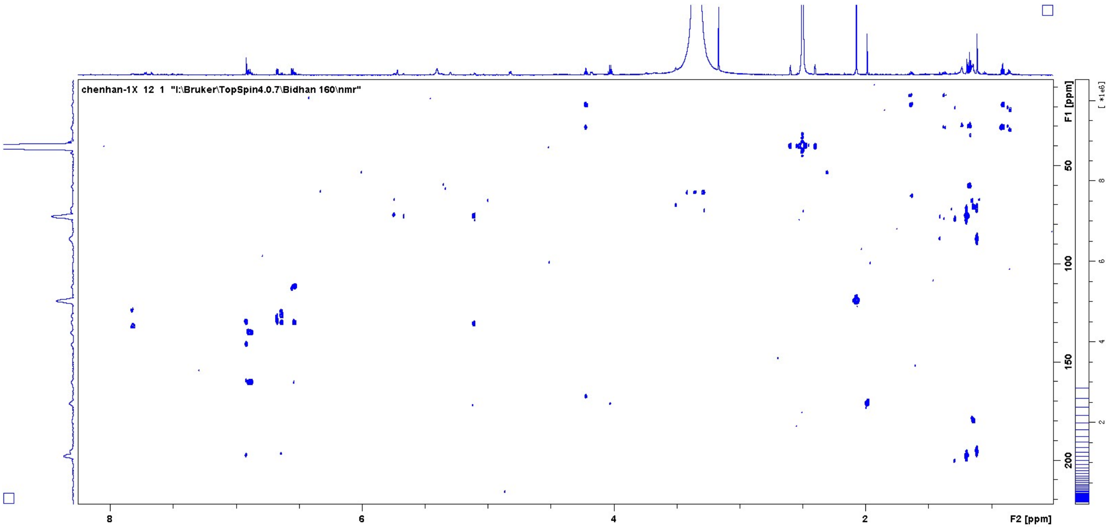

HMBC spectrum

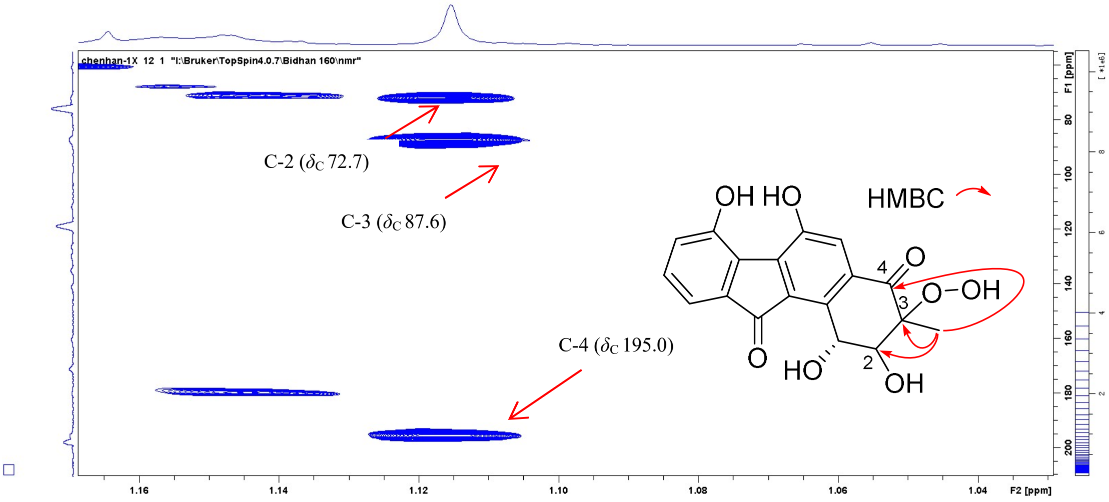

Enlarged HMBC spectrum

Supplementary Fig.25. Spectroscopic data for 14. (**c**) The HMBC and enlarged HMBC spectrum of **14** in DMSO-d_6.

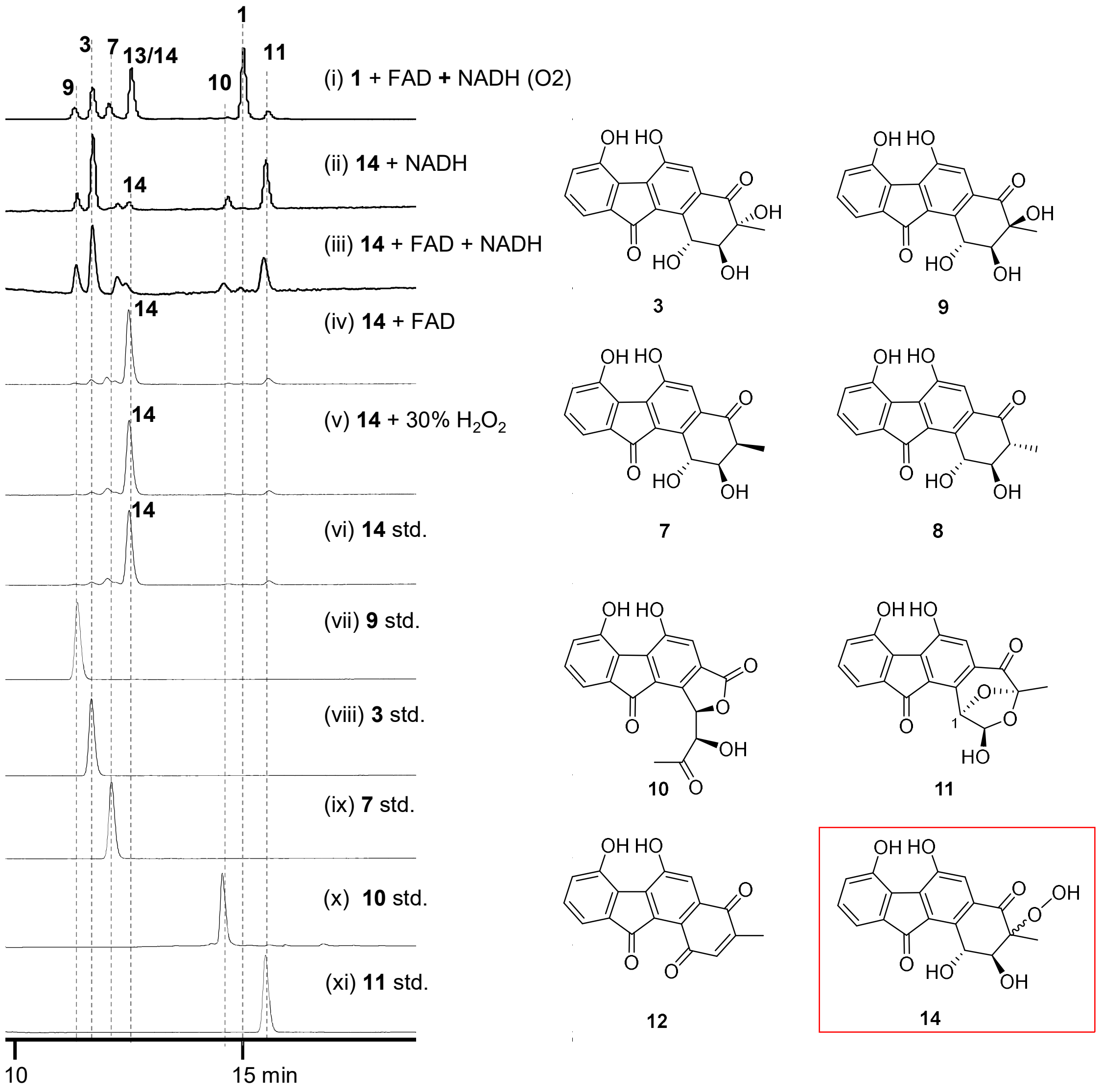

Supplementary Fig. 26. HPLC analysis of reactions with 14. HPLC analysis of reactions with **14** (i) standard assay contains 100 µM 1, 10 µM FAD and 2 mM NADH in presence of excess O_2; (ii) **14** + 2 mM NADH; (iii) 100 µM **14** + 10 µM FAD and 2 mM NADH; (iv) **14** + 10 µM FAD; (v) **14** + 30 % H_2O_2; (vi) **14** std.; (vii) **9** std.; (viii) **3** std.; (ix) **7** std.; (x) **10** std.; (xi) **11** std. The reaction mixtures were incubated at 30 ºC for 30 minutes HPLC analysis was performed using a polar column with UV detection at 304 nm.

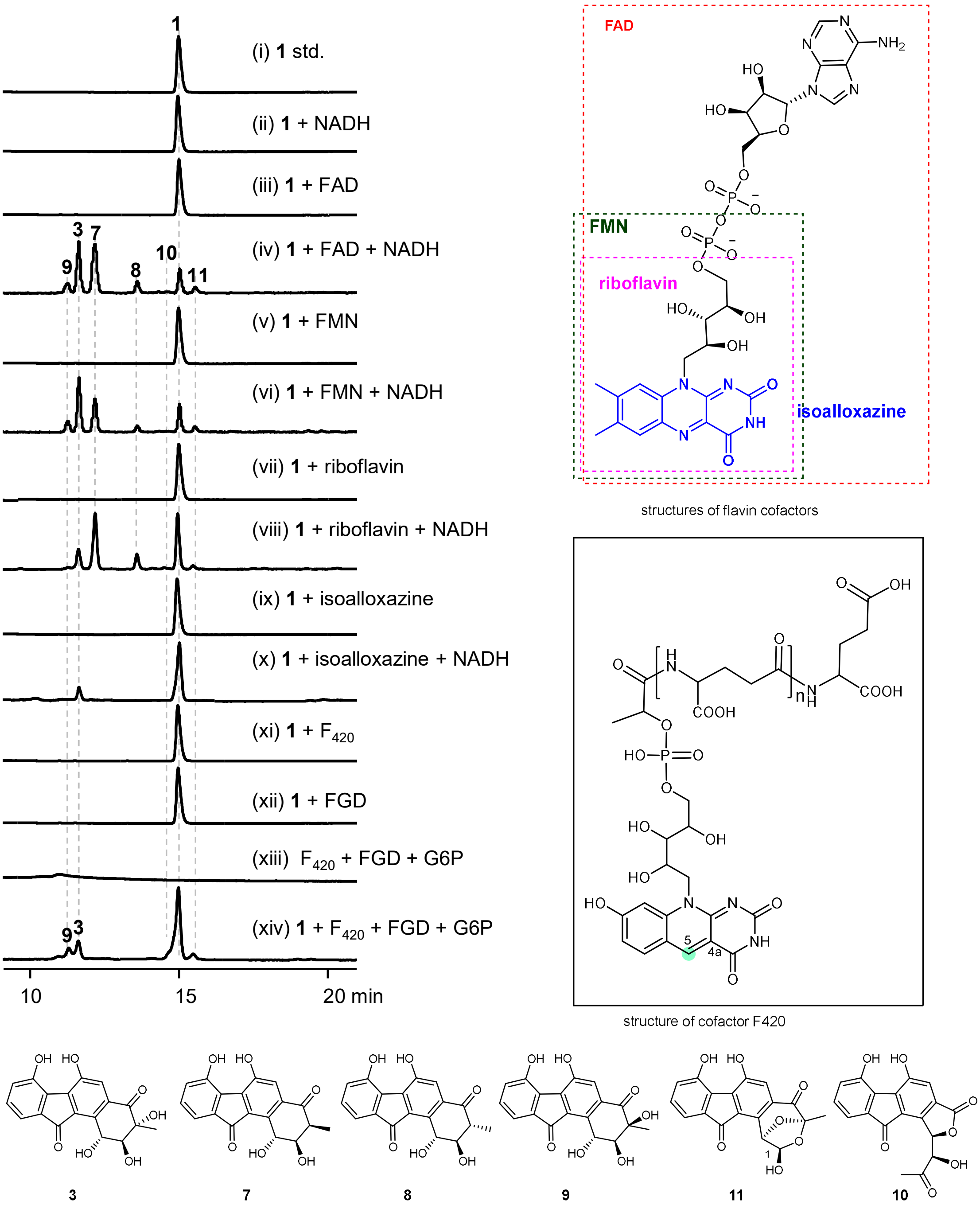

Supplementary Fig. 27. HPLC analysis of reactions 1 with different Flavin cofactors. HPLC analysis of reactions **1** with different flavin cofactors. (i) **1** standard; (ii) **1** + 10 mM NADH; (iii) **1** + 100 μM FAD; (iv) **1** + 100 μM FAD + 10 mM NADH; (v) **1** + 100 μM FMN; (vi) **1** + 100 μM FMN + 10 mM NADH; (vii) **1** + 100 μM riboflavin; (viii) **1** + 100 μM riboflavin + 10 mM NADH; (ix) **1** + 100 μM isoalloxazine; (x) **1** + 100 μM isoalloxazine + 10 mM NADH. The reaction mixtures were incubated at 30 ºC for 30 min. The next set of reactions were performed using the F_{420} system: (xi) **1** + 200 μM F_{420}; (xii) **1** + 10 μM F_{420}-dependent glucose-6-phosphate dehydrogenase (FGD); (xiii) 200 μM F_{420} + 2 mM glucose 6-phosphate (G6P) + 10 μM FGD; (xiv) **1** + 200 μM F_{420} + 2 mM G6P + 10 μM FGD. The reaction mixtures were incubated at 30 ºC for 10 h. HPLC analysis was run using polar column with UV detection at 304 nm.

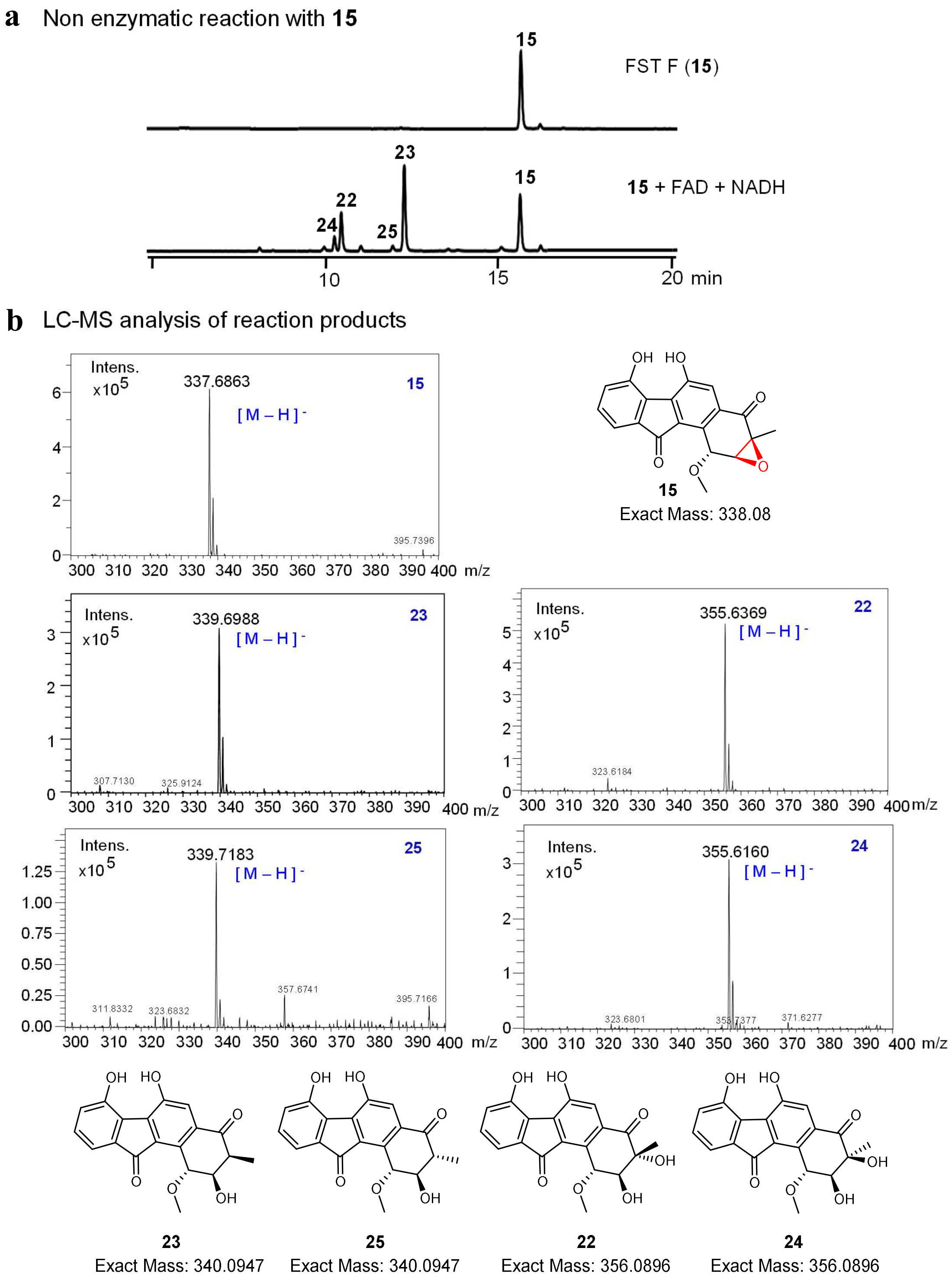

Supplementary Fig. 28. **HPLC and LC-MS analysis of FST F (15) reaction with FAD/NADH.** (**a**) HPLC analysis of the reaction mixtures containing 100 μM 15, 100 μM FAD and 10 mM NADH in 50 mM PBS buffer (pH 7). The reaction mixtures were incubated at 30 °C for 30 min. (**b**) LC-MS analysis of **7**-methyl- FST F (**15**) reaction products.

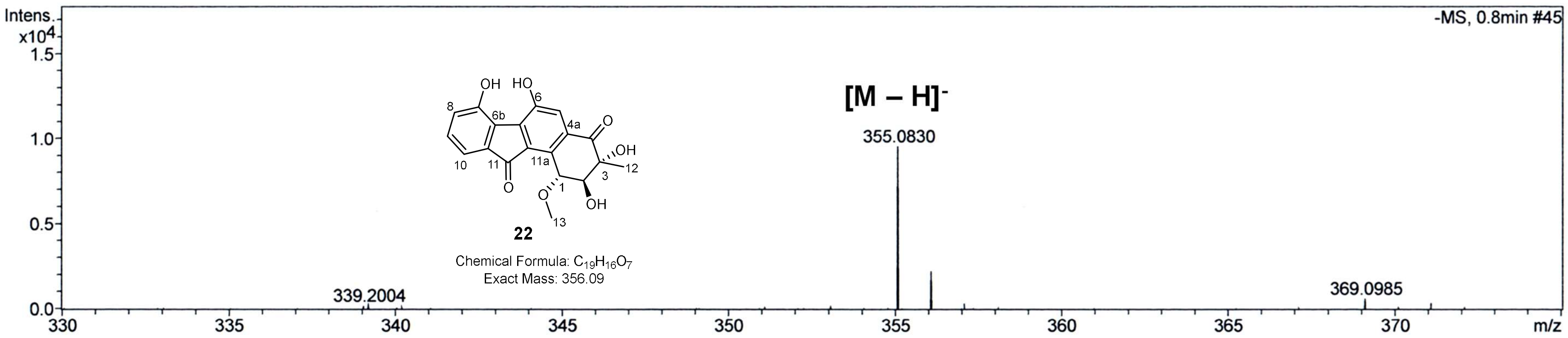

Meas. m/z	#	Ion Formula	Score	m/z	err [ppm]	err [mDa]	mSigma	rdb	e⁻ Conf	N-Rule
355.0830	1	C19H15O7	100.00	355.0823	1.9	0.7	11.9	12.5	even	ok
711.1733	1	C38H31O14	100.00	711.1719	-2.0	-1.4	4.0	23.5	even	ok

Supplementary Fig. 29. Spectroscopic data for 22. (a) HRESIMS spectrum.

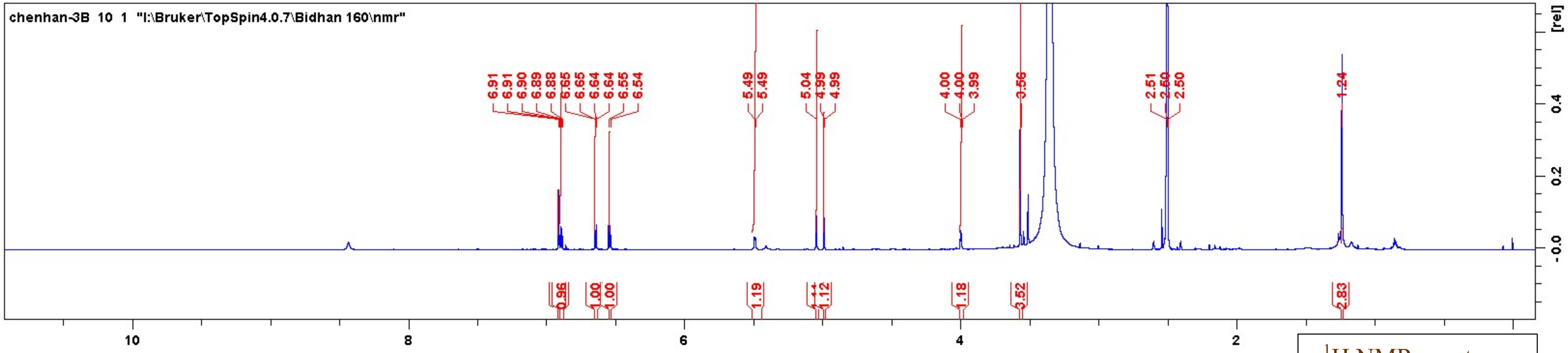

^{1}H NMR spectrum

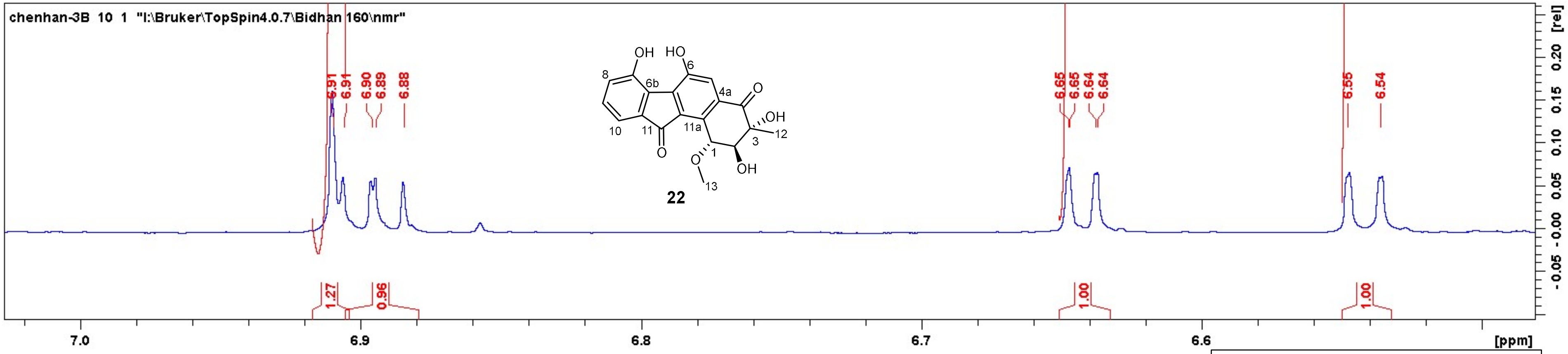

Enlarged ^{1}H NMR spectrum

Supplementary Fig. 29. Spectroscopic data for 22. (**b**) The ^{1}H and enlarged ^{1}H NMR spectrum of **22** in DMSO-d_6.

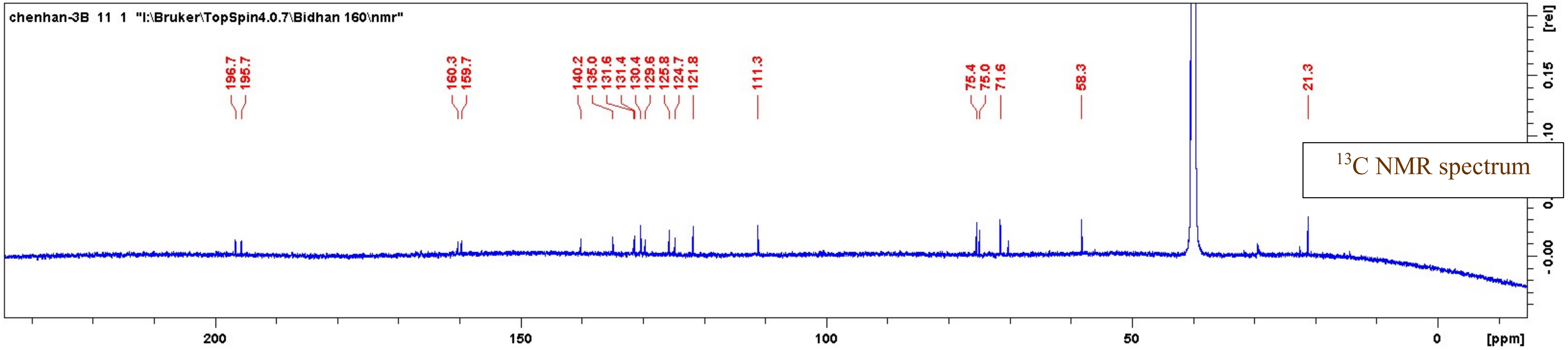

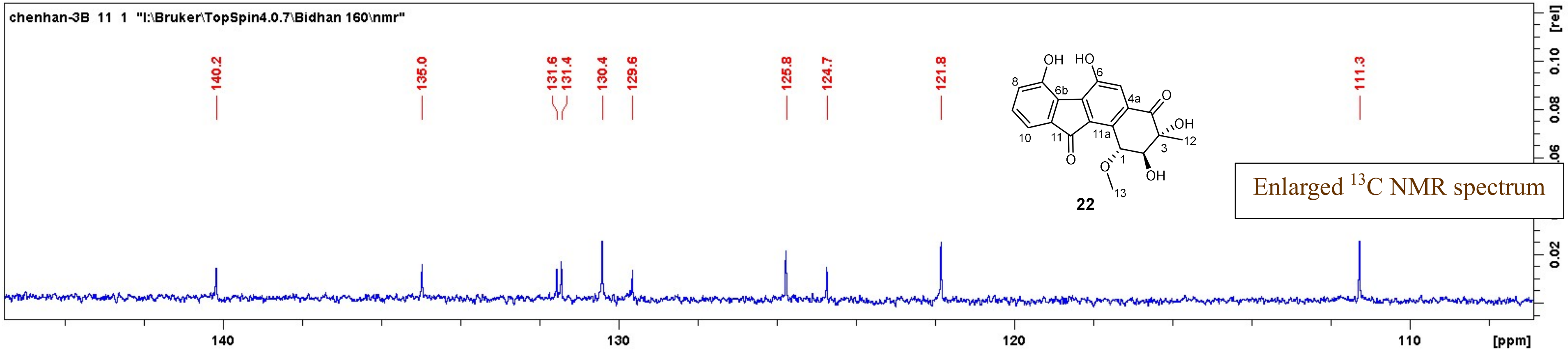

Supplementary Fig. 29. Spectroscopic data for 22. (**c**) The ^{13}C and enlarged ^{13}C NMR spectrum of **22** in DMSO-d_6.

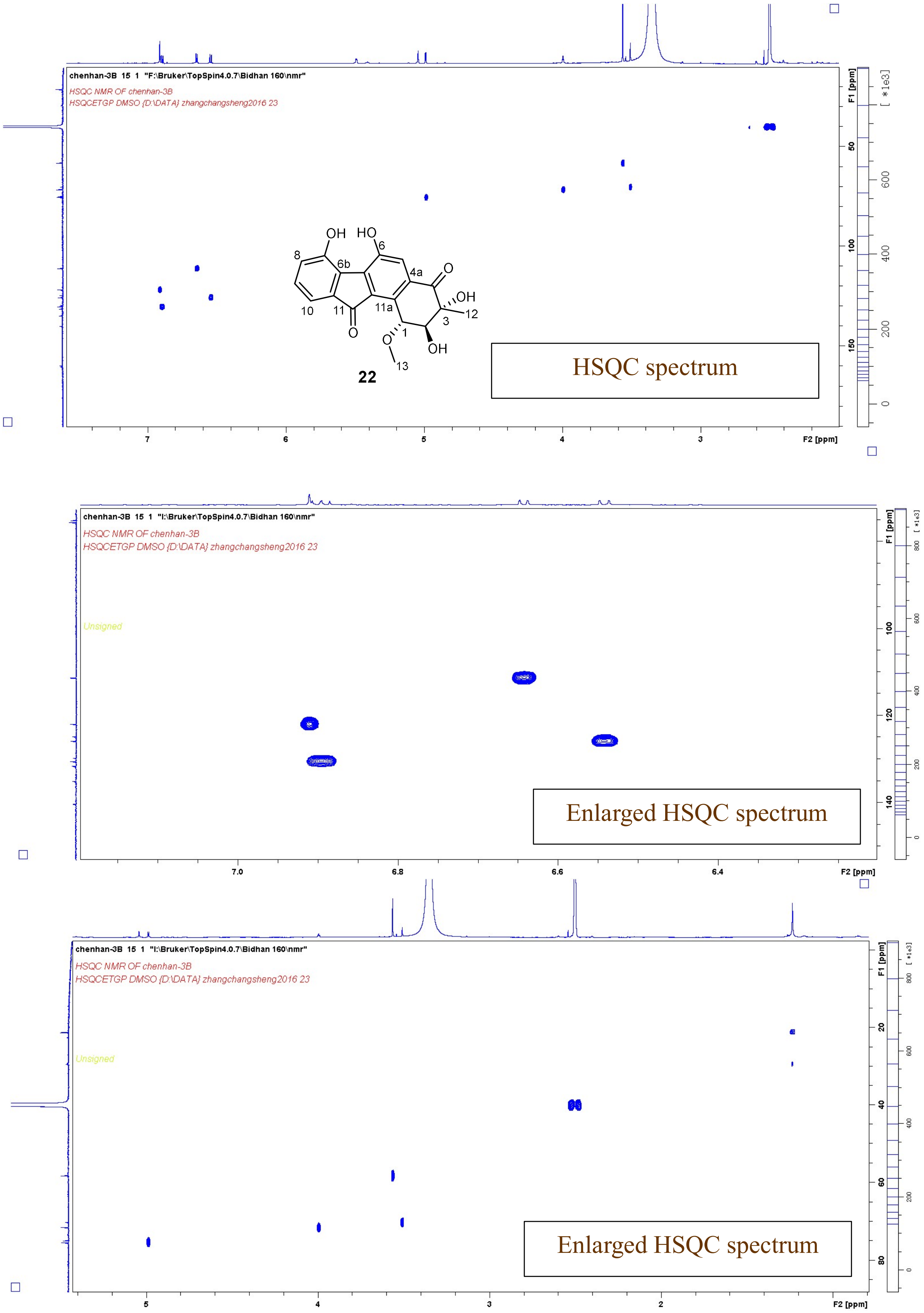

Supplementary Fig. 29. Spectroscopic data for 22. (**d**) The HSQC and enlarged HSQC spectrum of **22** in DMSO-d_6.

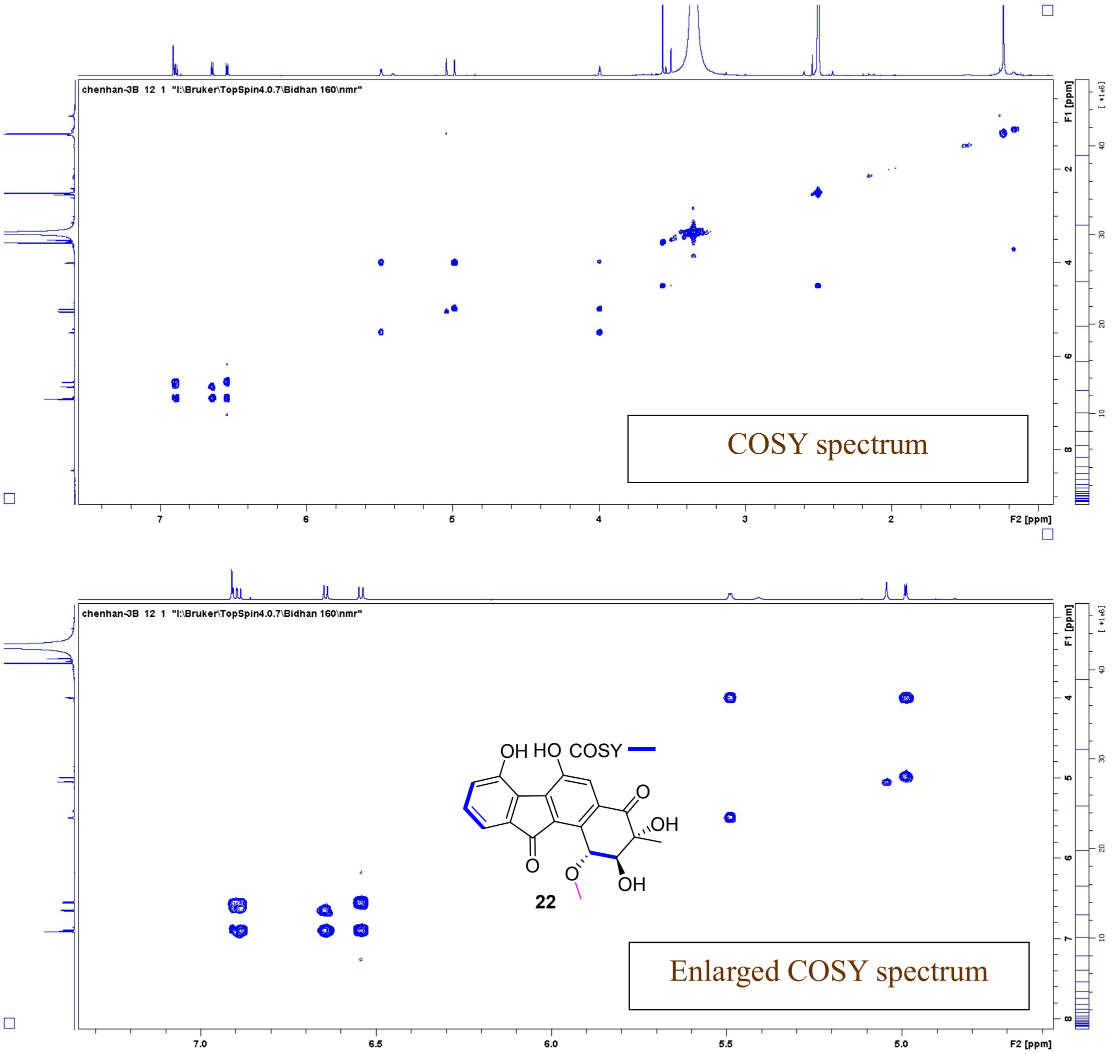

Supplementary Fig. 29. Spectroscopic data for 22. (**e**) The COSY and enlarged COSY spectrum of **22** in DMSO-d_6.

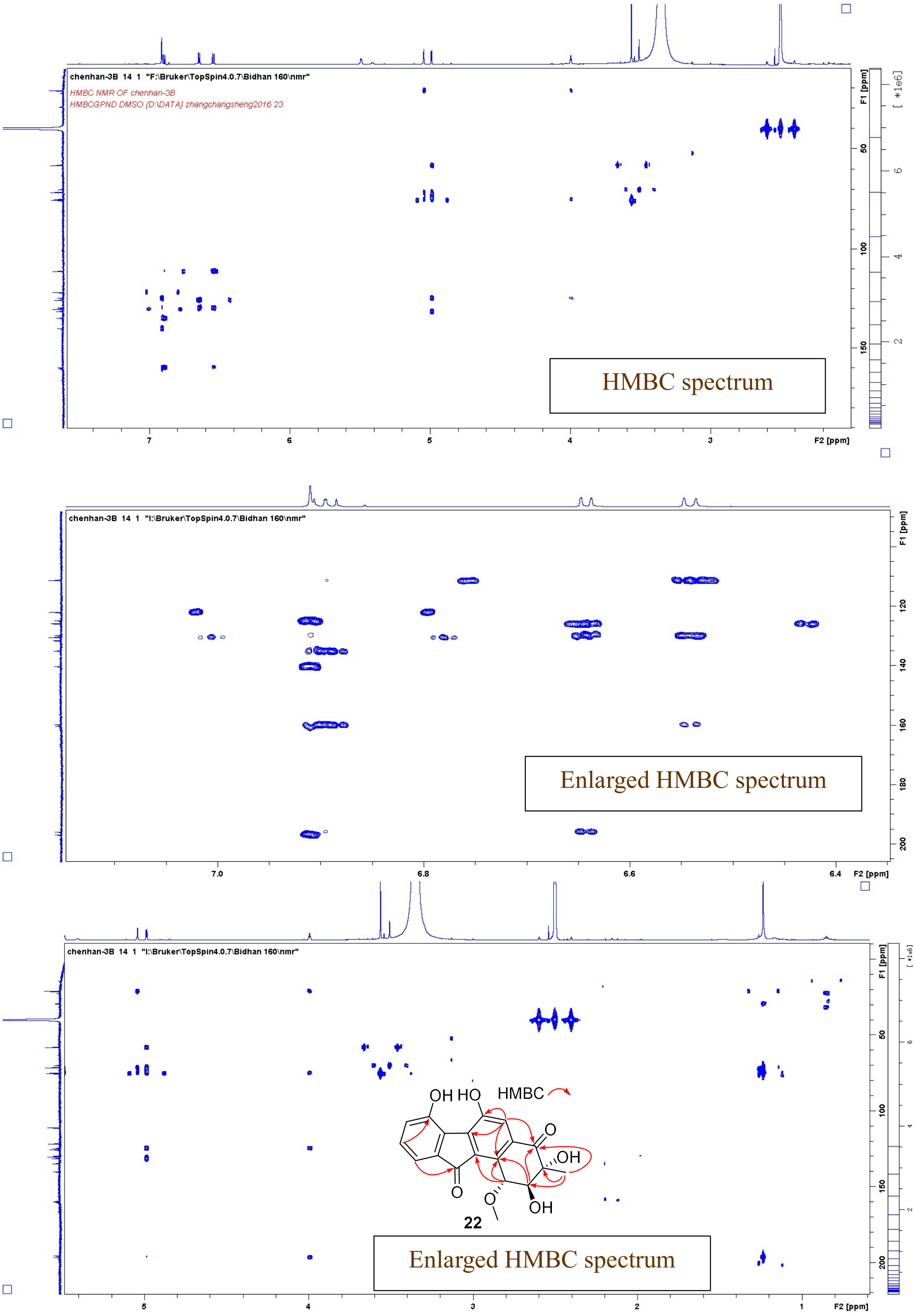

Supplementary Fig. 29. Spectroscopic data for 22. (**f**) The HMBC and enlarged HMBC spectrum of **22** in DMSO-d_6.

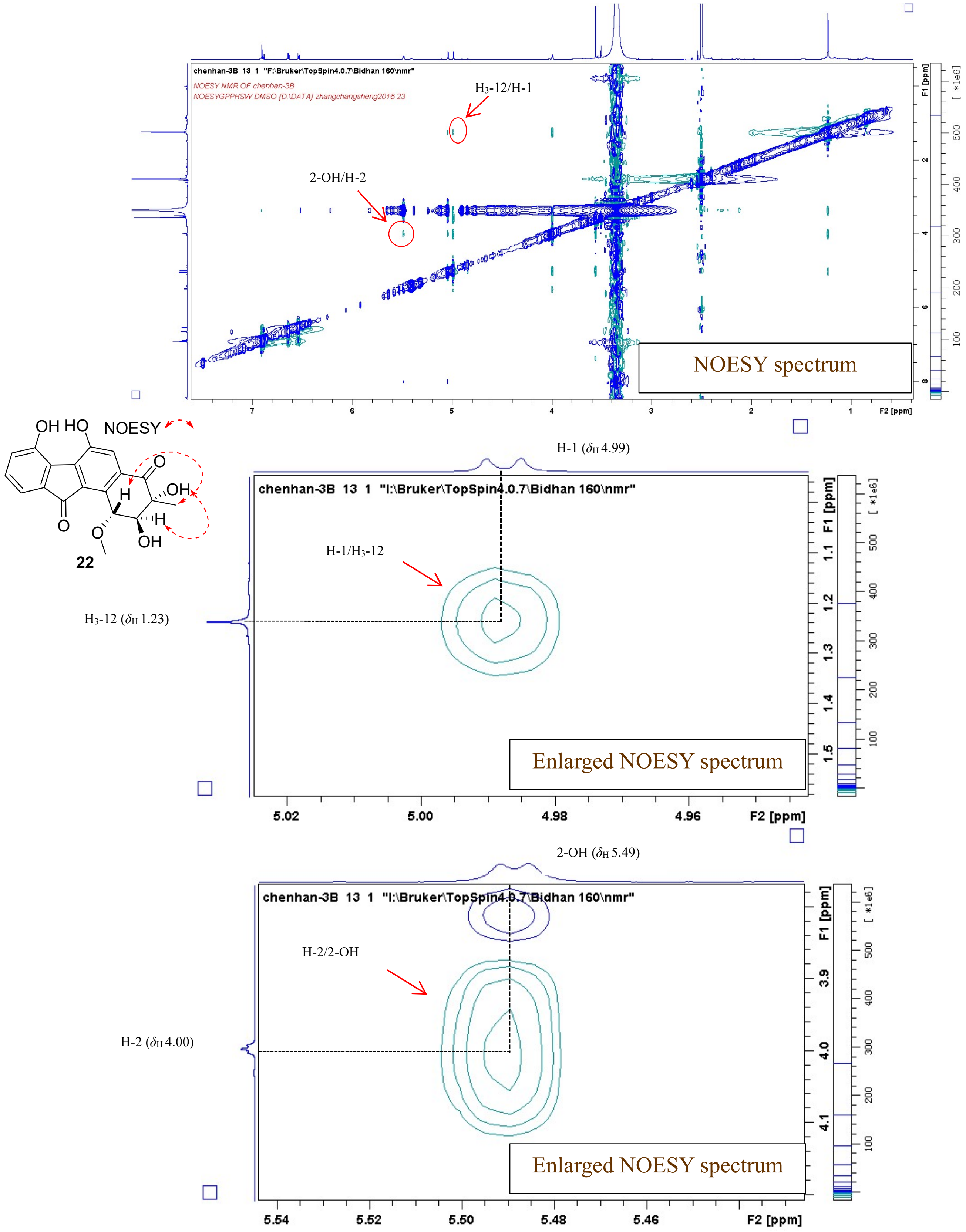

Supplementary Fig. 29. Spectroscopic data for 22. **(g)** The NOESY and enlarged NOESY spectrum of **22** in DMSO-d_6.

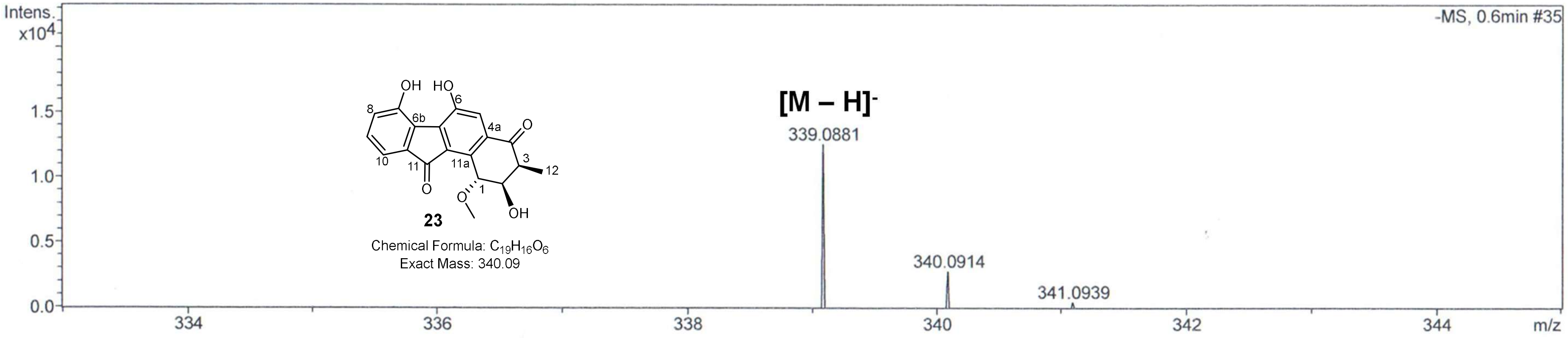

Meas. m/z	#	Ion Formula	Score	m/z	err [ppm]	err [mDa]	mSigma	rdb	e⁻ Conf	N-Rule
339.0881	1	C19H15O6	100.00	339.0874	-2.0	-0.7	6.8	12.5	even	ok
679.1831	1	C38H31O12	100.00	679.1821	-1.5	-1.0	22.3	23.5	even	ok

Supplementary Fig. 30. Spectroscopic data for 23. (**a**) HRESIMS spectrum.

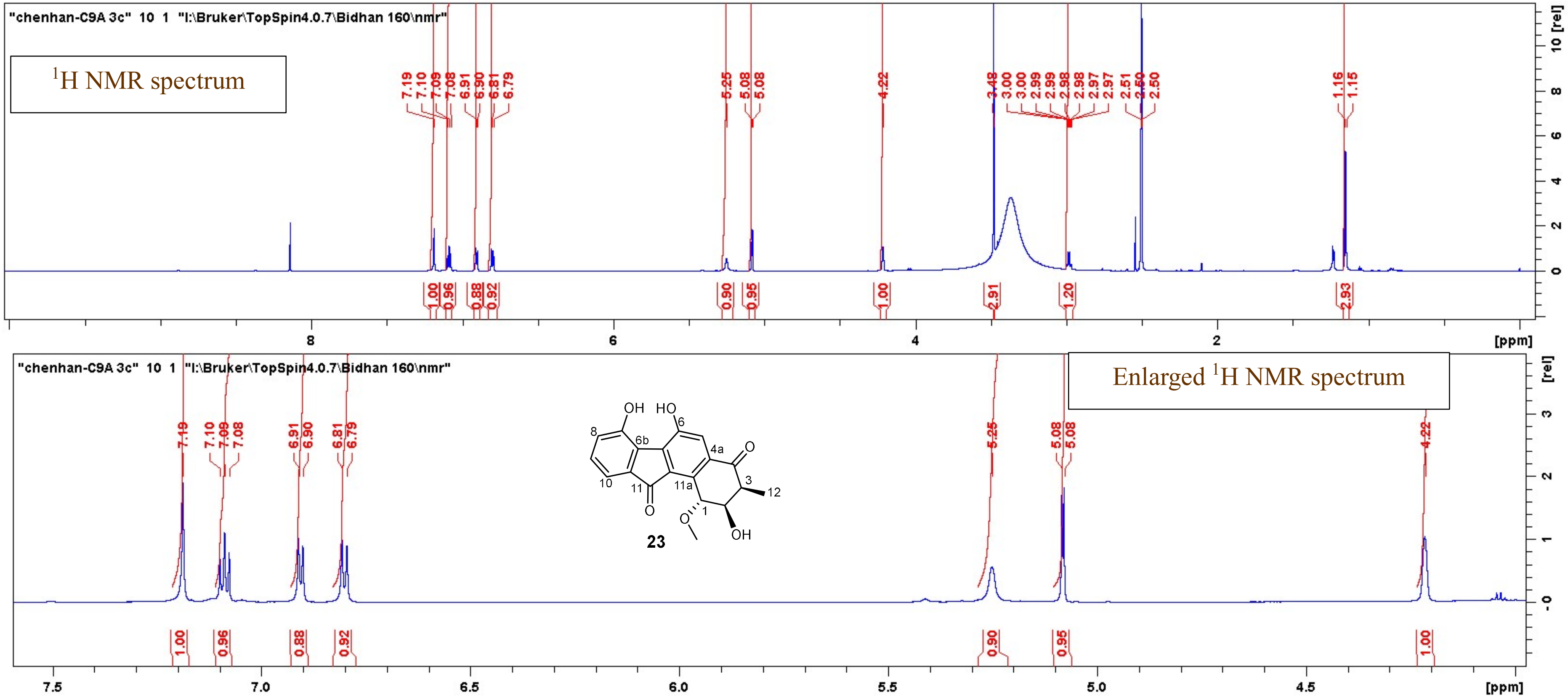

Supplementary Fig. 30. Spectroscopic data for 23. (**b**) The ^{1}H and enlarged ^{1}H NMR spectrum of **23** in DMSO-d_6.

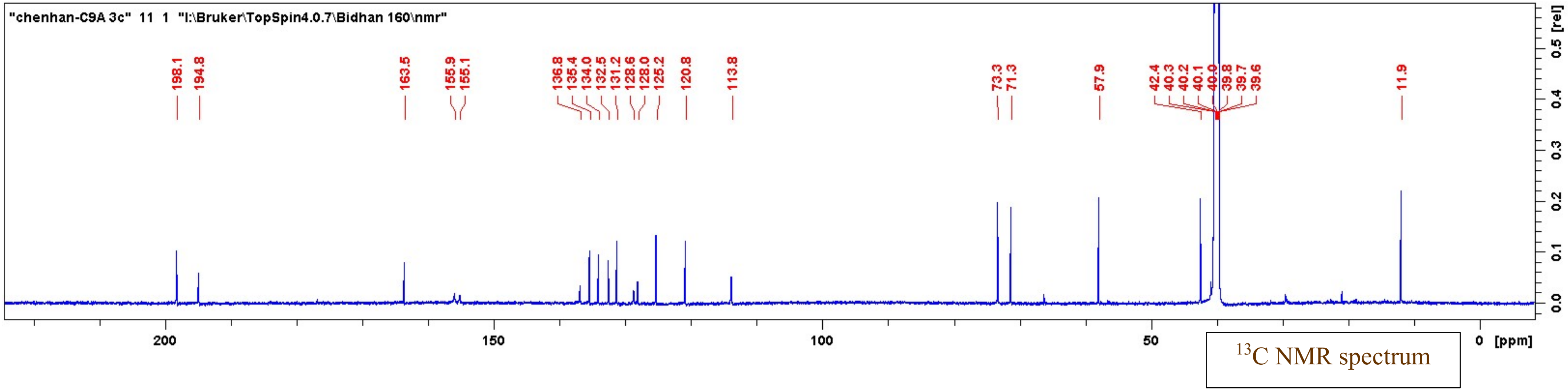

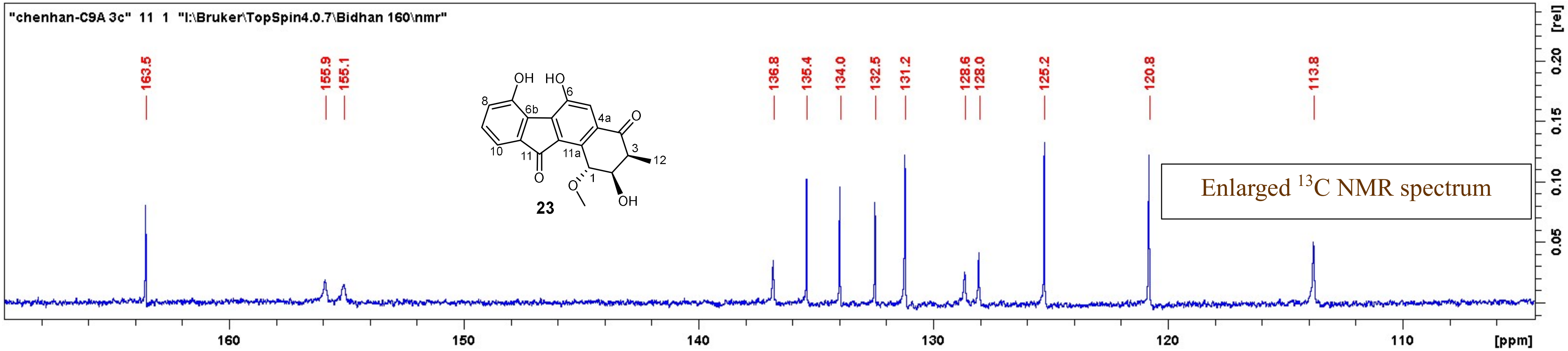

Supplementary Fig. 30. Spectroscopic data for 23. (**c**) The ^{13}C and enlarged ^{13}C NMR spectrum of **23** in DMSO-d_6.

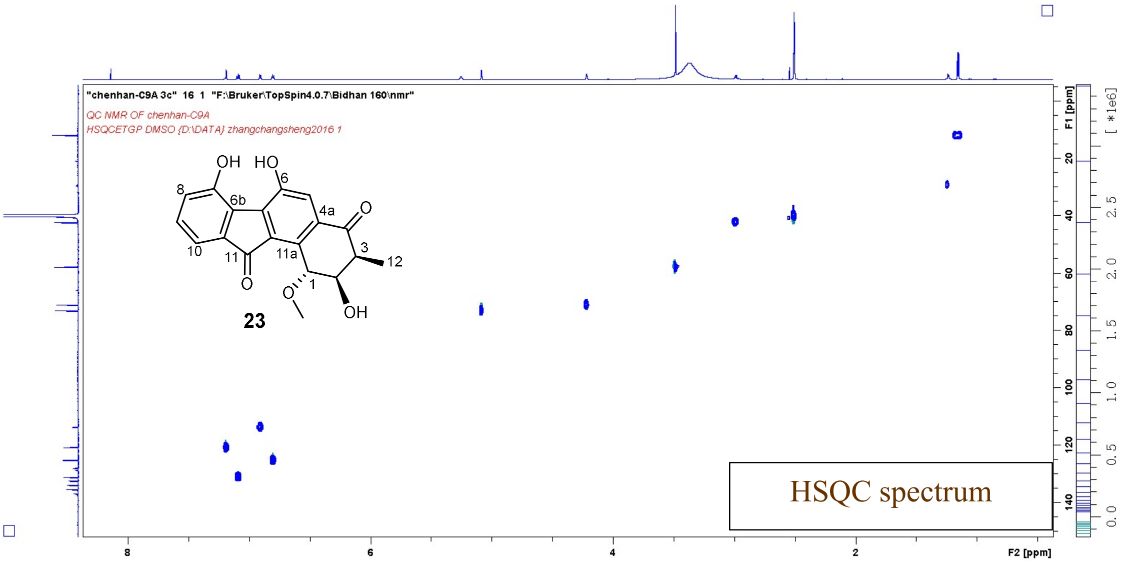

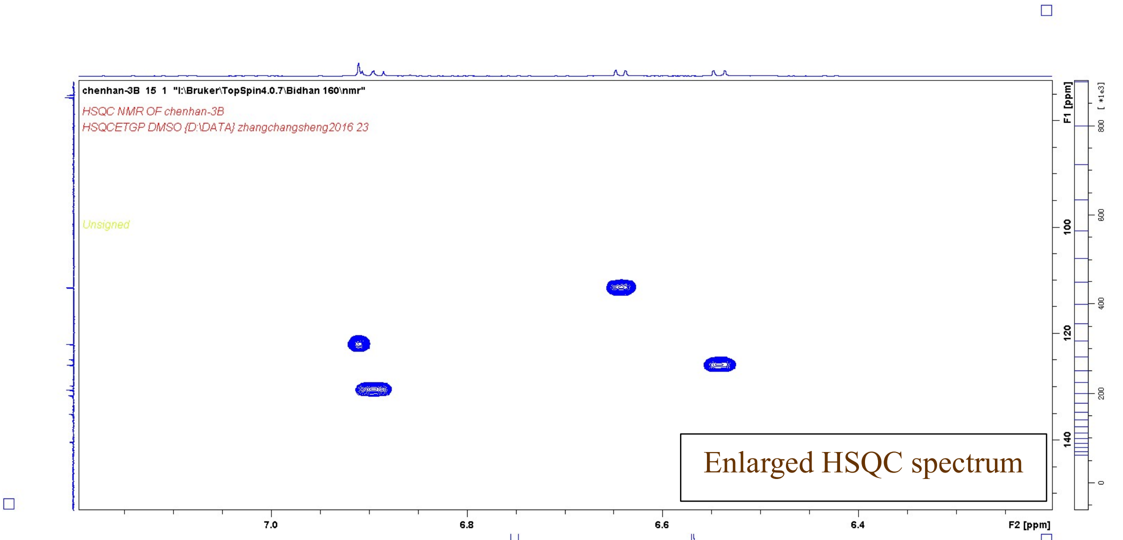

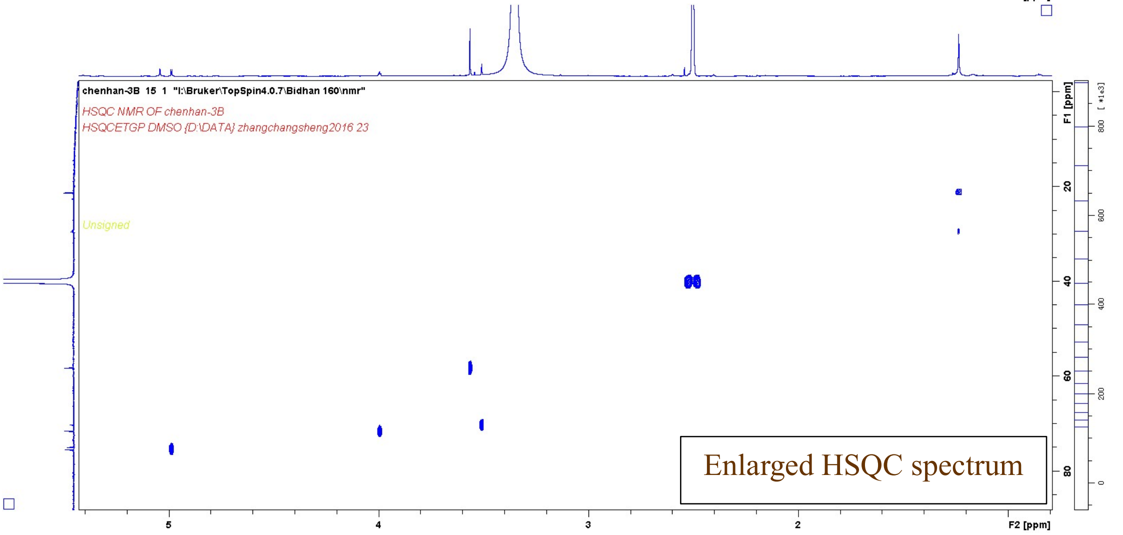

Supplementary Fig. 30. Spectroscopic data for 23. (**d**) The HSQC and enlarged HSQC spectrum of **23** in DMSO-d_6.

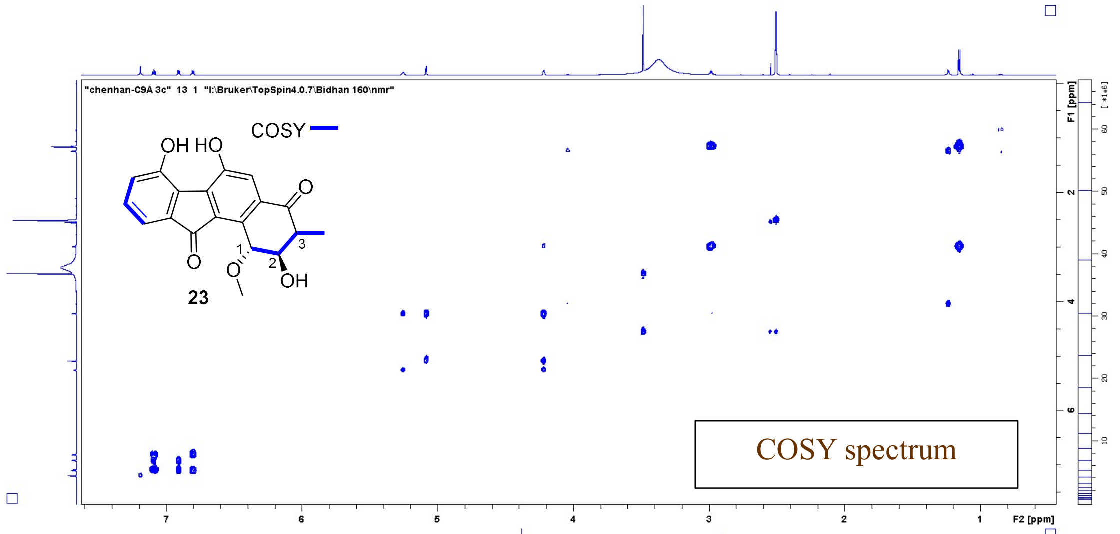

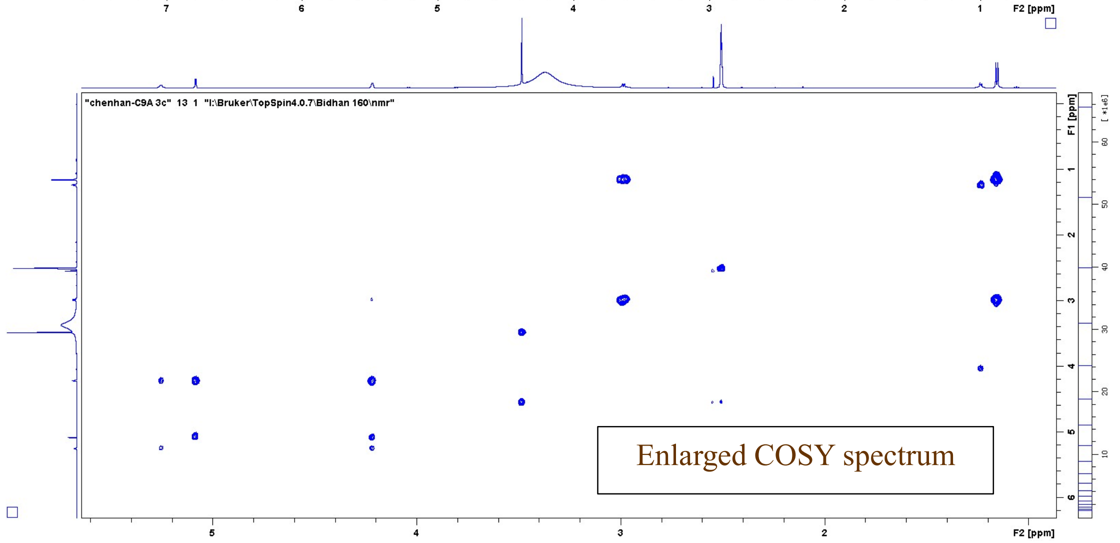

Supplementary Fig. 30. Spectroscopic data for 23. (**e**) The COSY and enlarged COSY spectrum of **23** in DMSO-d_6.

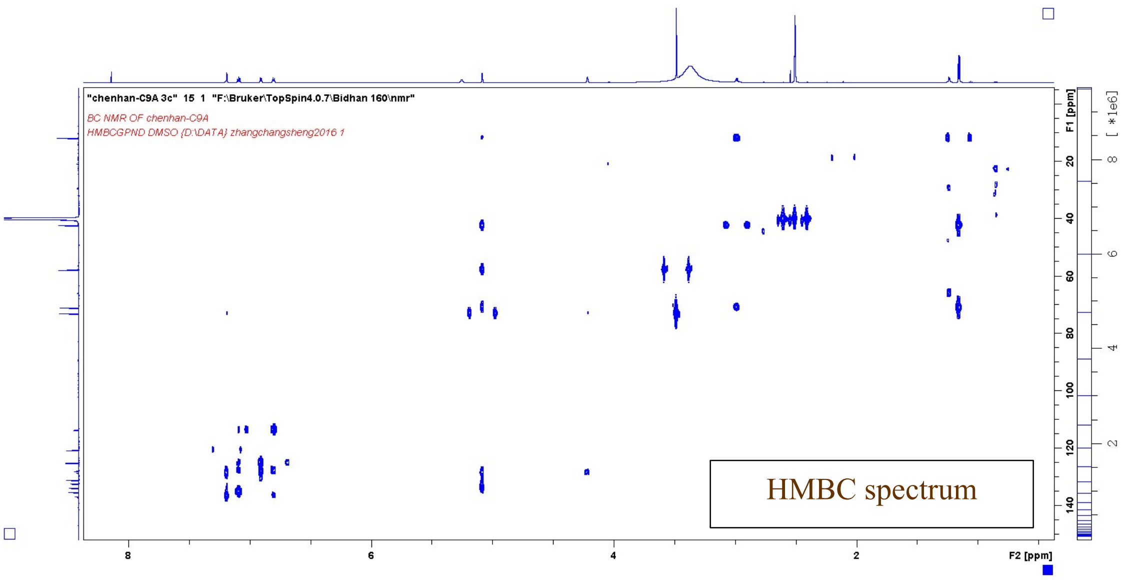

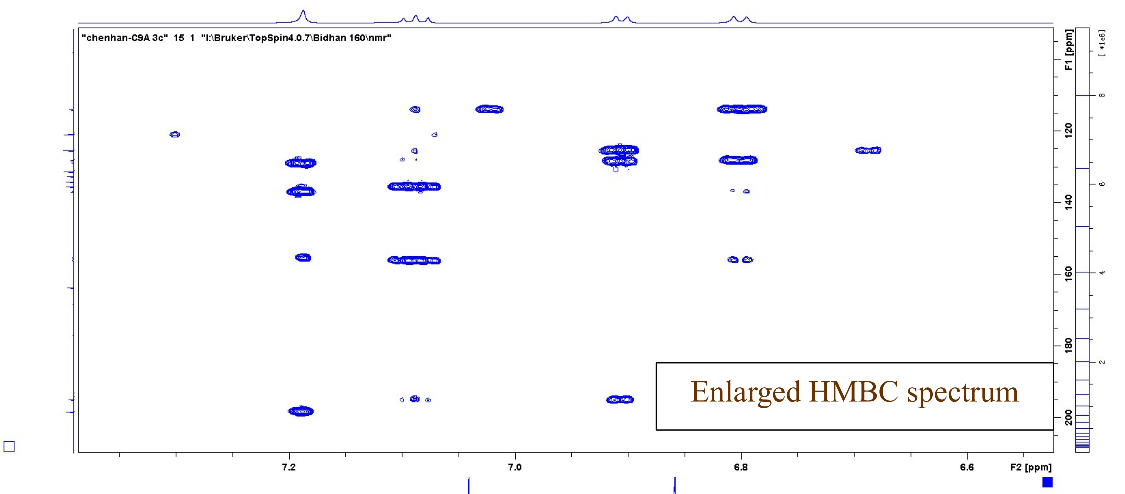

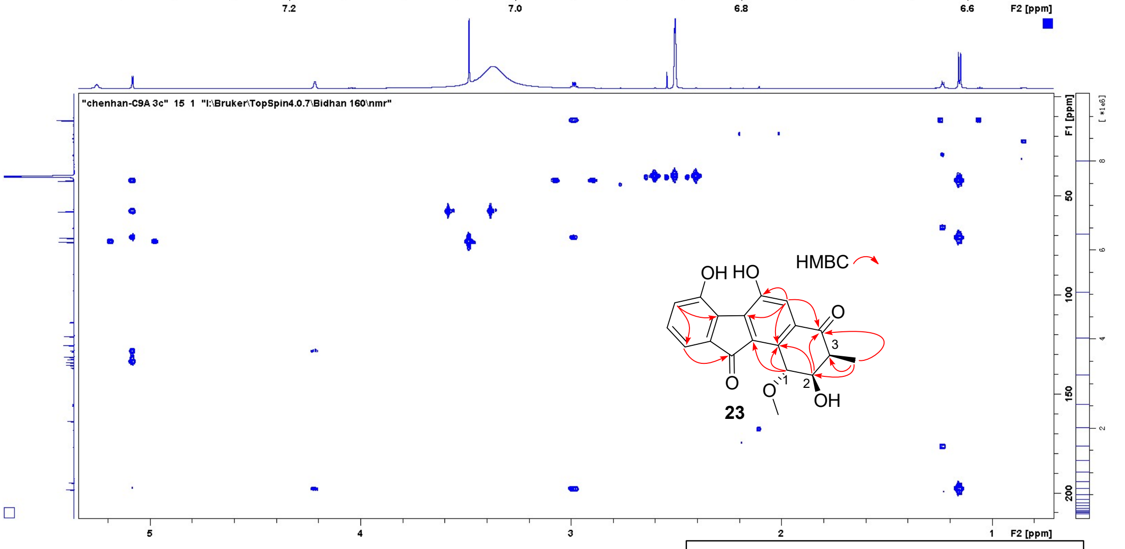

Enlarged HMBC spectrum

Supplementary Fig. 30. Spectroscopic data for 23. (f) The HMBC and enlarged HMBC spectrum of **23** in DMSO-d_6.

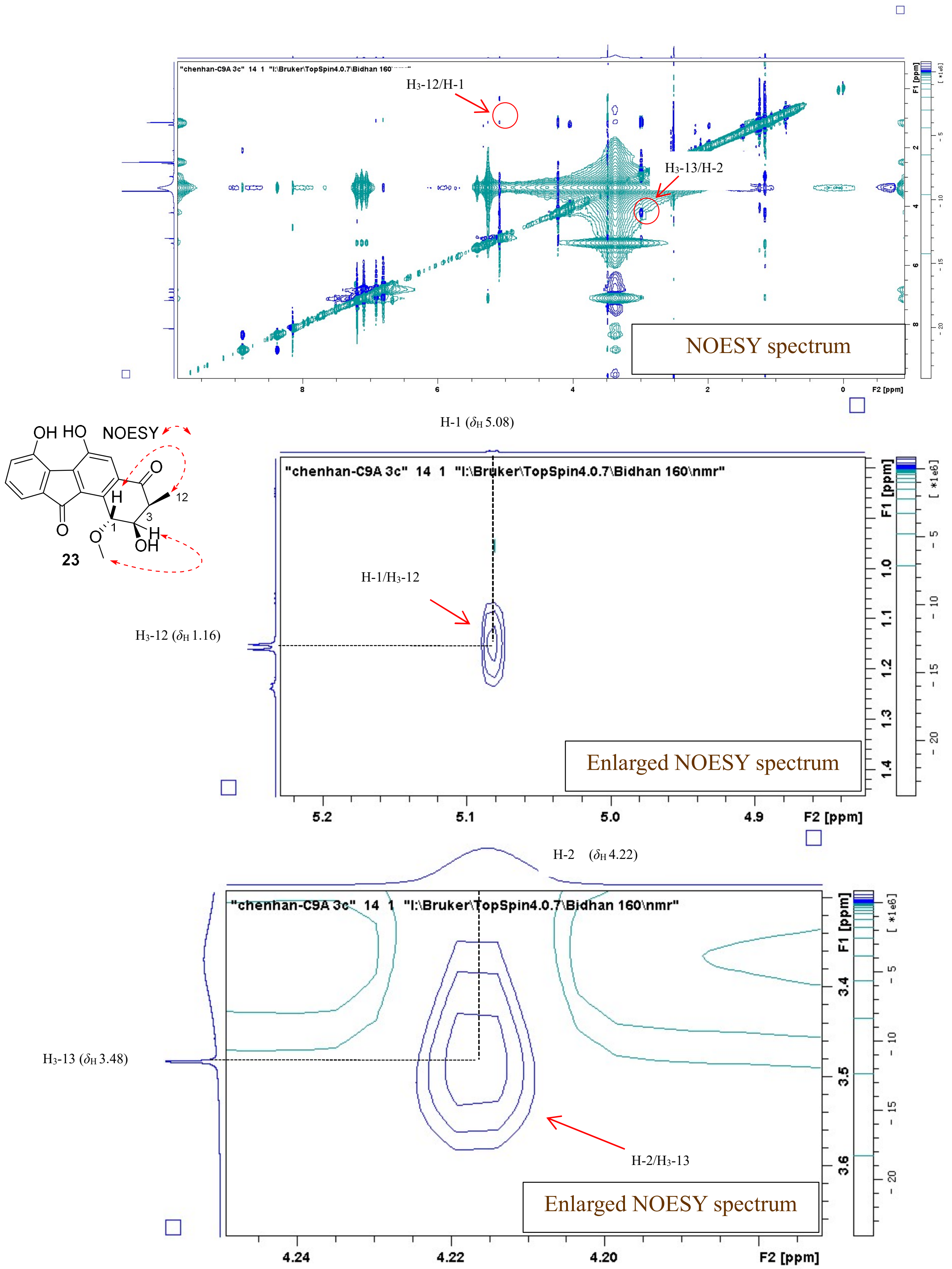

Supplementary Fig. 30. Spectroscopic data for 23. (g) The NOESY and enlarged NOESY spectrum of **23** in DMSO-d_6.

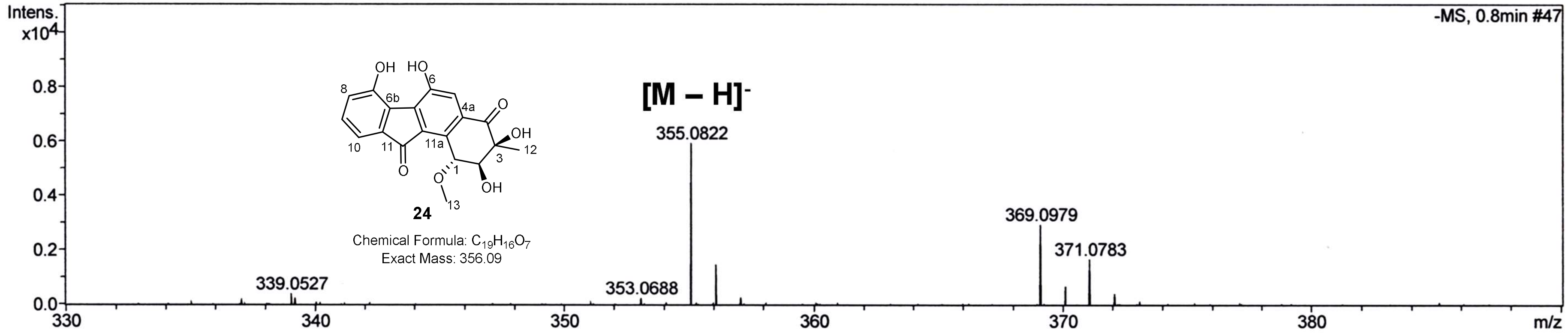

Meas. m/z	#	Ion Formula	Score	m/z	err [ppm]	err [mDa]	mSigma	rdb	e¯ Conf	N-Rule
355.0822	1	C19H15O7	100.00	355.0823	-0.2	-0.1	20.8	12.5	even	ok
711.1716	1	C38H31O14	100.00	711.1719	-0.4	-0.3	42.6	23.5	even	ok

Supplementary Fig. 31. Spectroscopic data for 24. (a) HRESIMS spectrum.

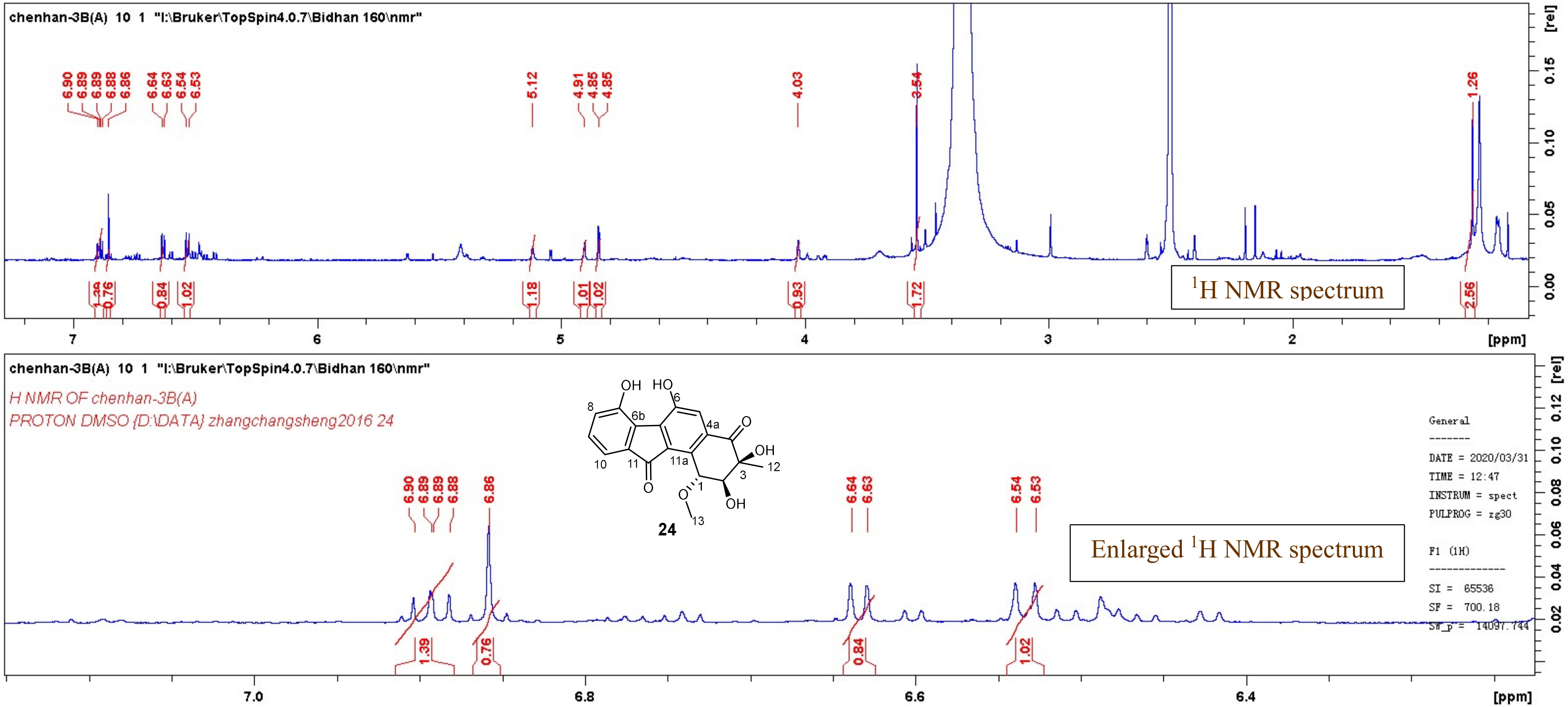

Supplementary Fig. 31. Spectroscopic data for 24. (b) The ^{1}H and enlarged ^{1}H NMR spectrum of **24** in DMSO-d_6.

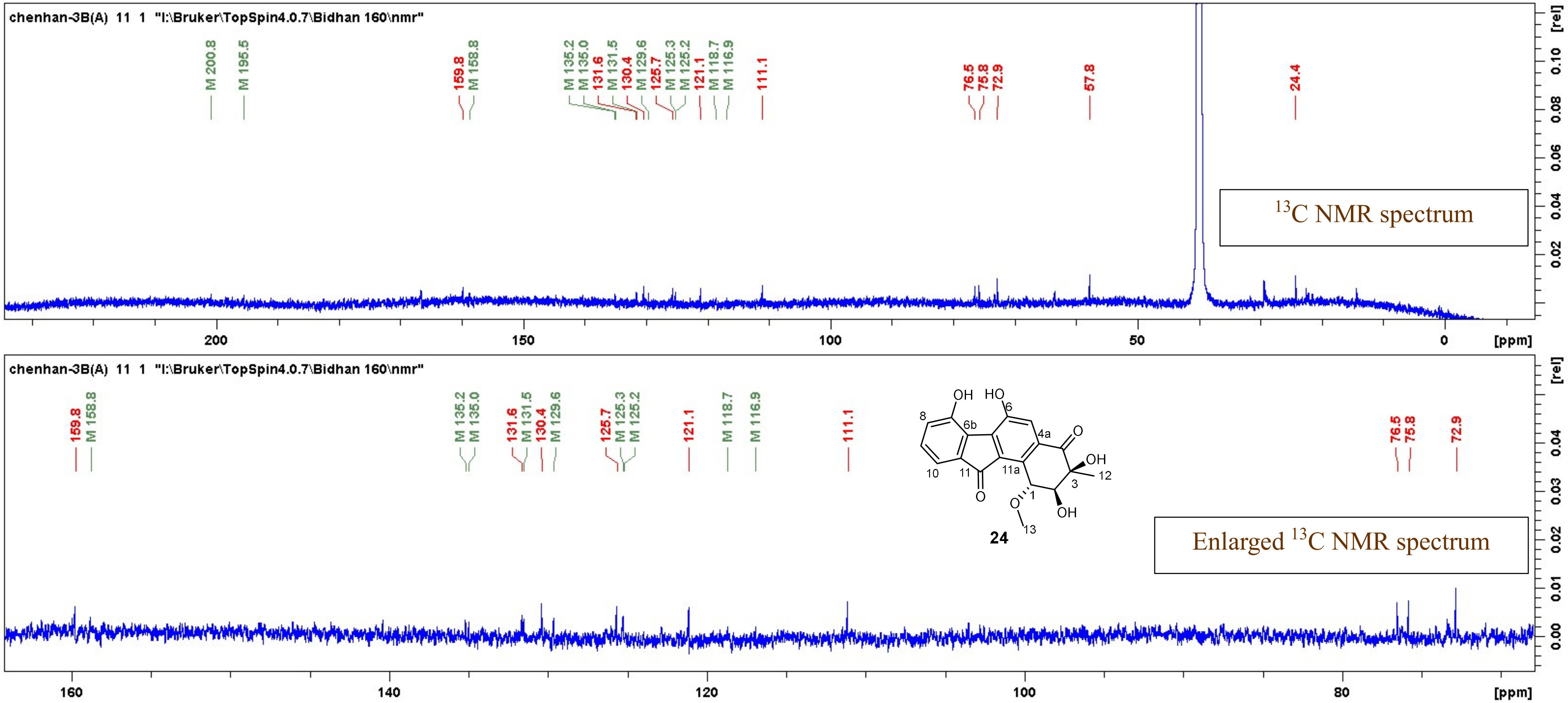

Supplementary Fig. 31. Spectroscopic data for 24. (**c**) The ^{13}C and enlarged ^{13}C NMR spectrum of **24** in DMSO-d_6.

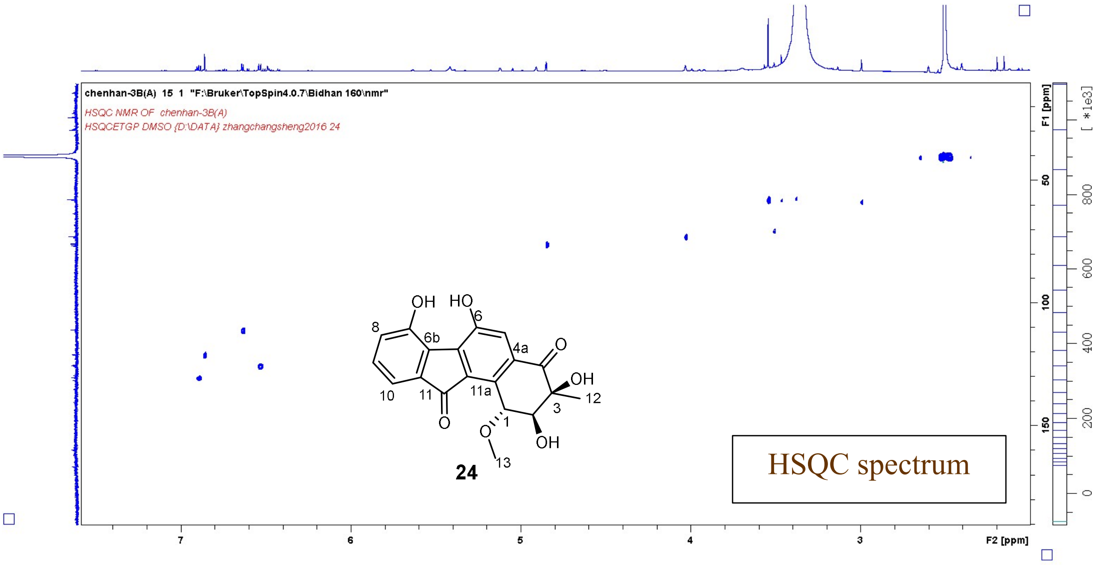

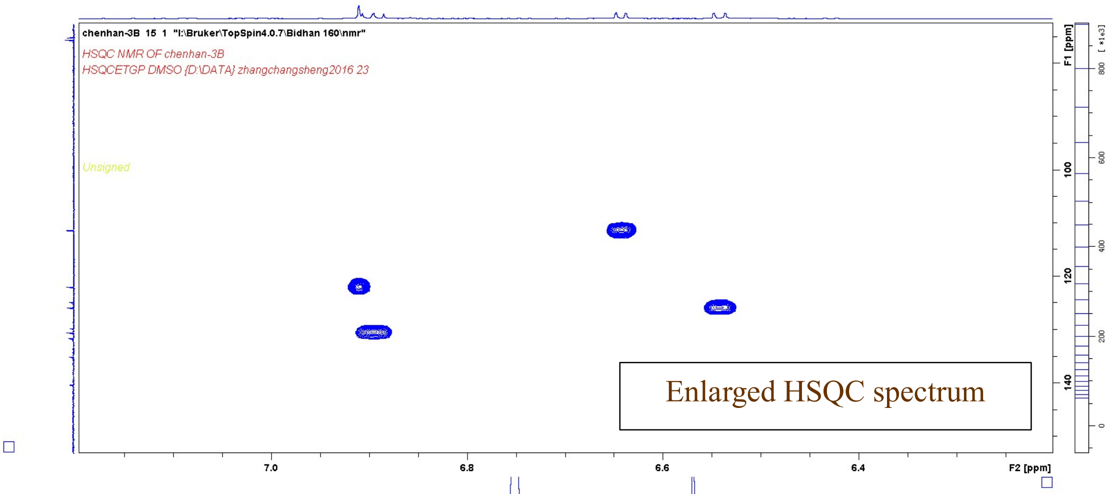

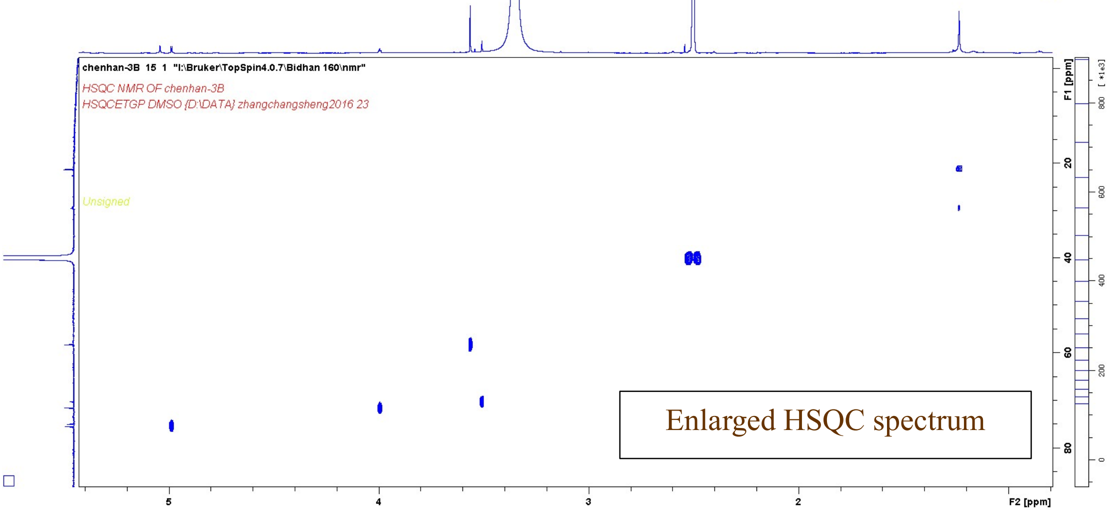

Supplementary Fig. 31. Spectroscopic data for 24. (**d**) The HSQC and enlarged HSQC spectrum of **24** in DMSO-d_6.

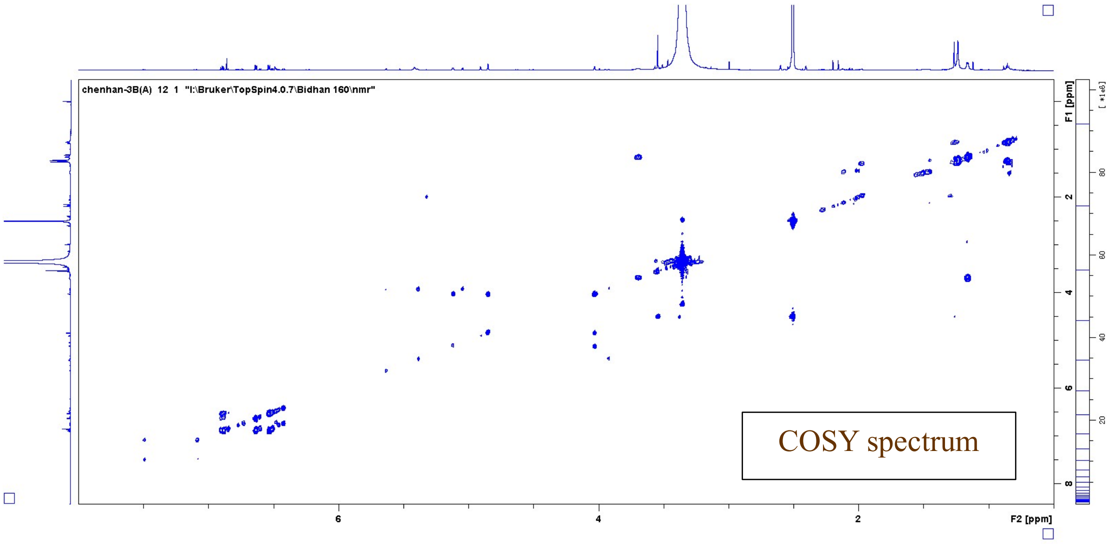

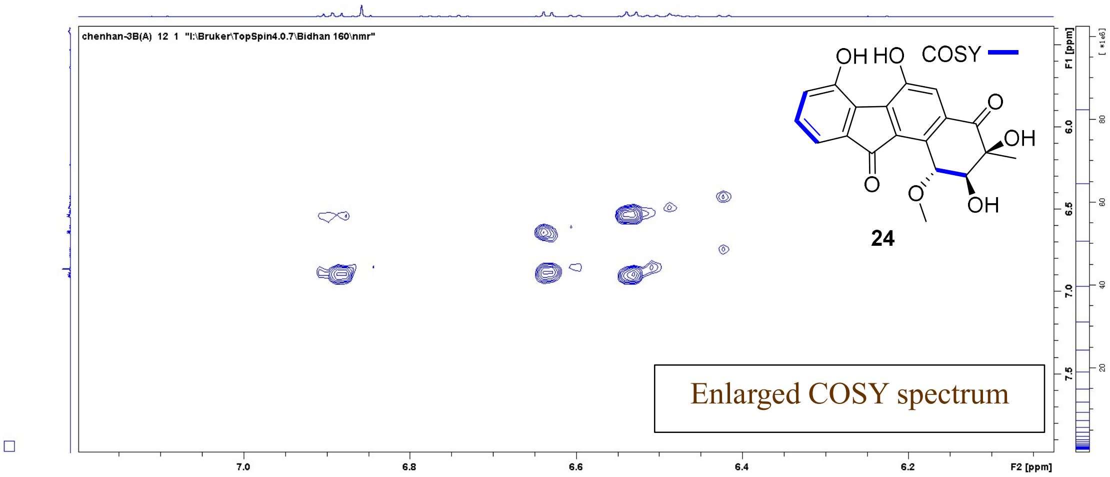

Supplementary Fig. 31. Spectroscopic data for 24. (**e**) The COSY and enlarged COSY spectrum of **24** in DMSO-d_6.

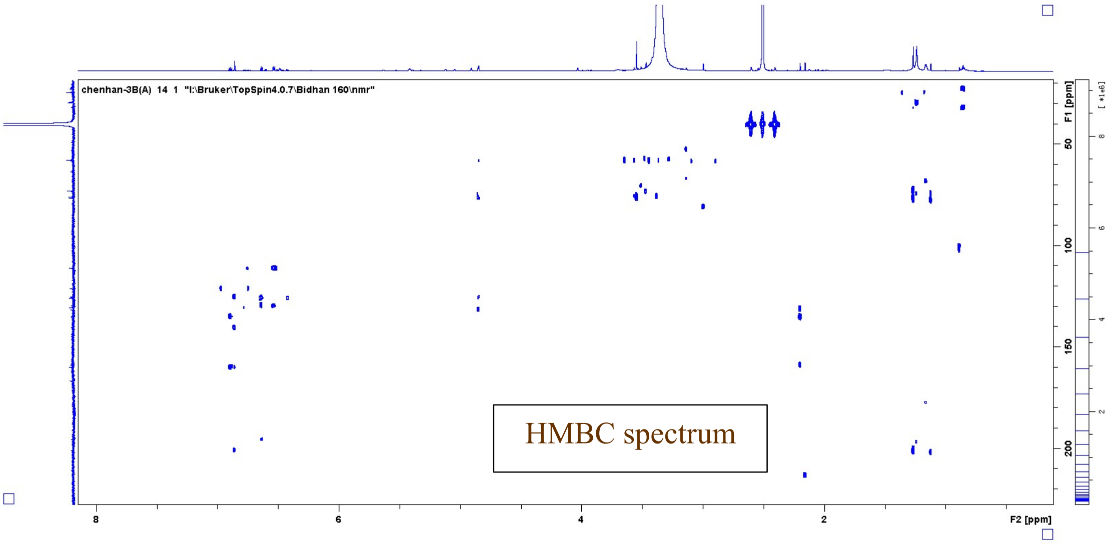

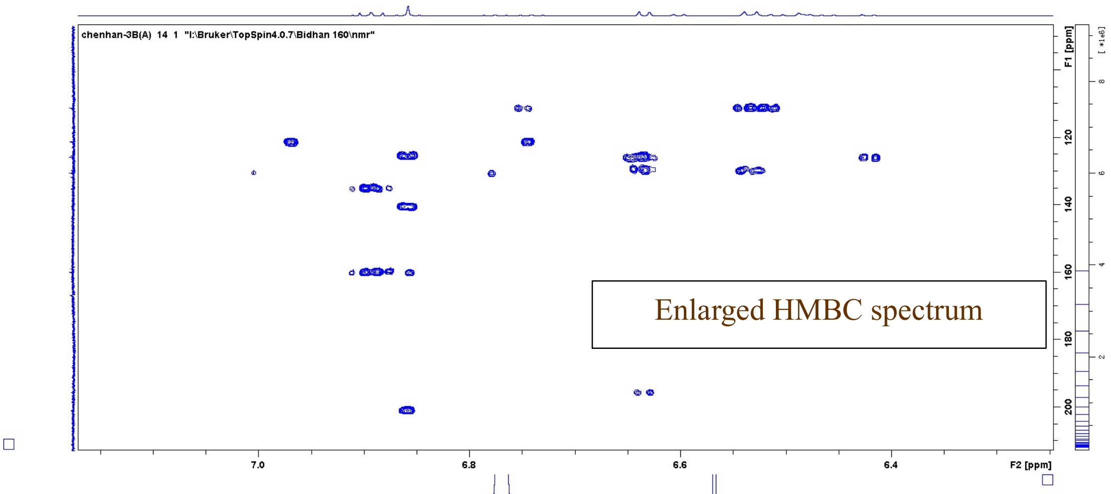

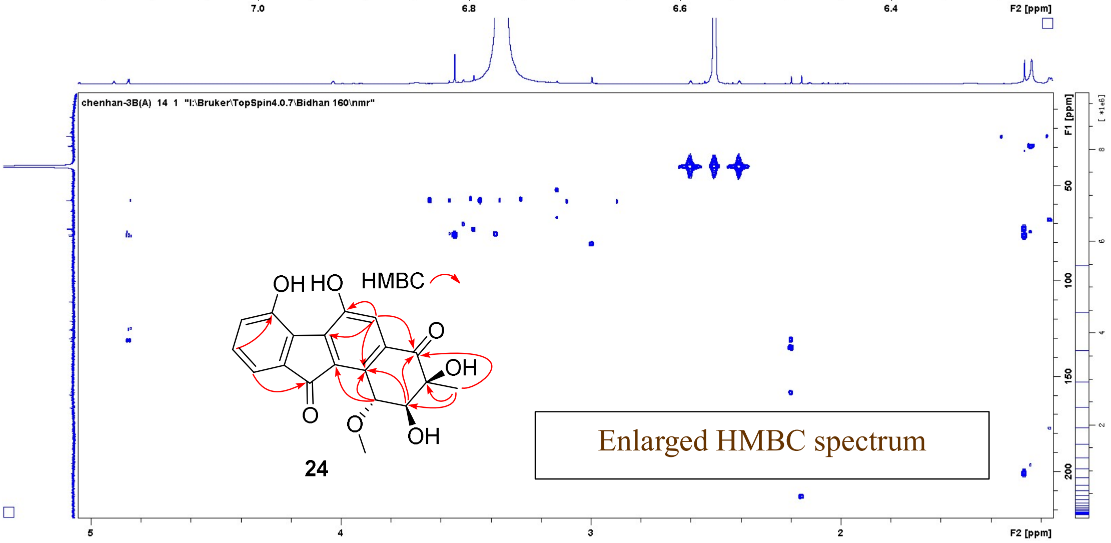

Supplementary Fig. 31. Spectroscopic data for 24. (**f**) The HMBC and enlarged HMBC spectrum of **24** in DMSO-d_6.

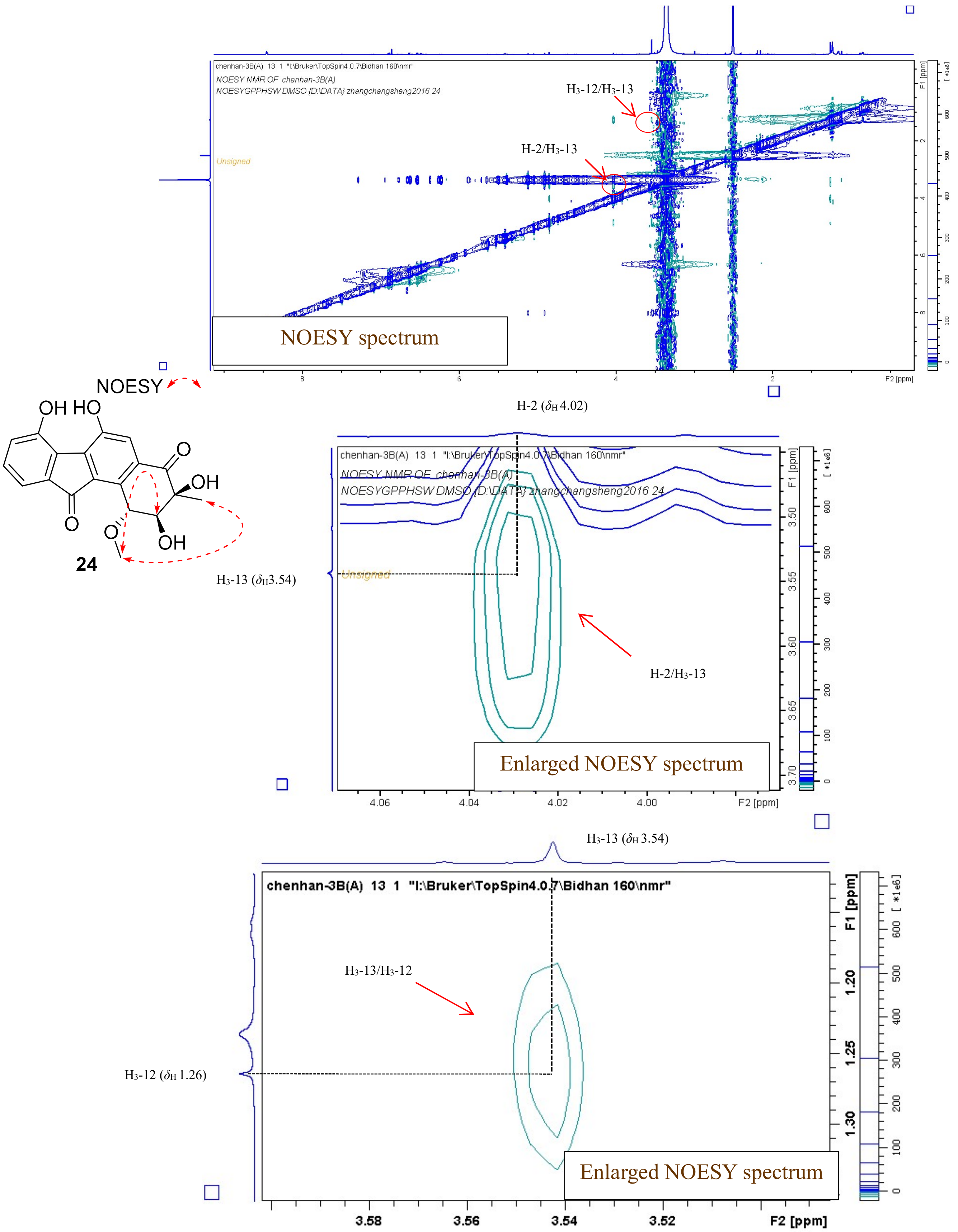

Supplementary Fig. 31. Spectroscopic data for 24. **(g)** The NOESY and enlarged NOESY spectrum of **24** in DMSO-d_6.

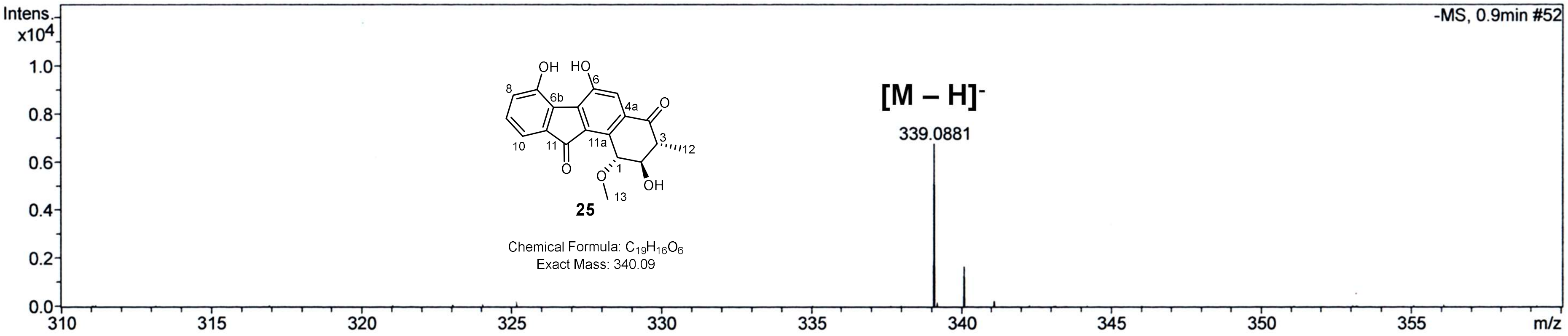

Meas. m/z	#	Ion Formula	Score	m/z	err [ppm]	err [mDa]	mSigma	rdb	e¯ Conf	N-Rule
339.0881	1	C19H15O6	100.00	339.0874	-2.1	-0.7	17.9	12.5	even	ok
679.1826	1	C38H31O12	100.00	679.1821	0.7	0.5	43.7	23.5	even	ok

Supplementary Fig.32. Spectroscopic data for 25. (**a**) HRESIMS spectrum.

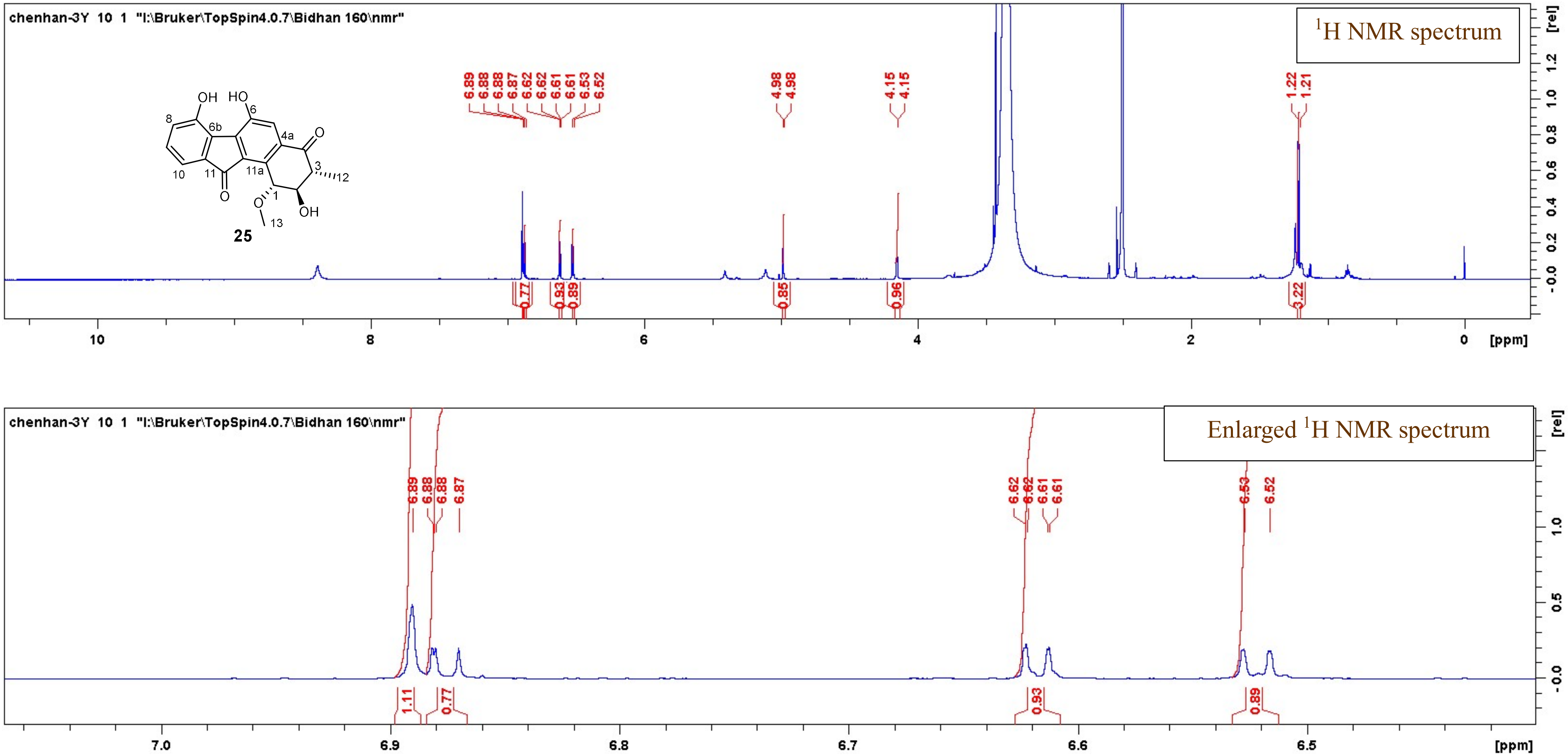

Supplementary Fig.32. Spectroscopic data for 25. (**b**) The ^{1}H and enlarged ^{1}H NMR spectrum of **25** in DMSO-d_6.

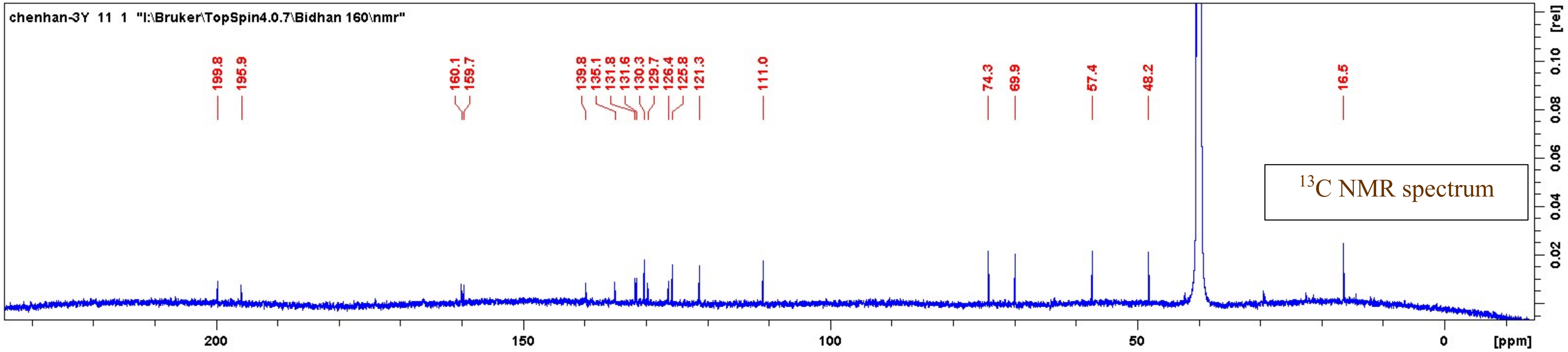

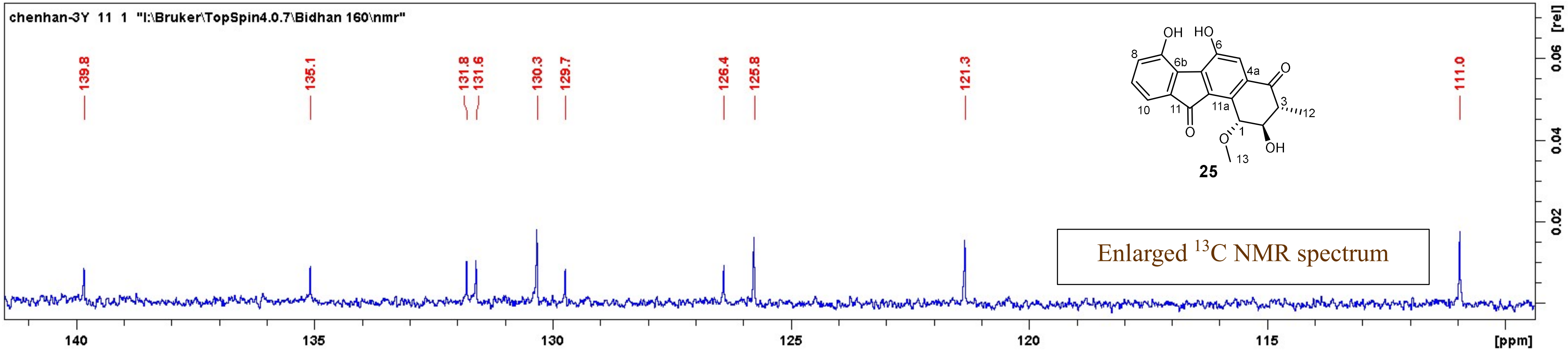

Supplementary Fig.32. Spectroscopic data for 25. (**c**) The ^{13}C and enlarged ^{13}C NMR spectrum of **25** in DMSO-d_6.

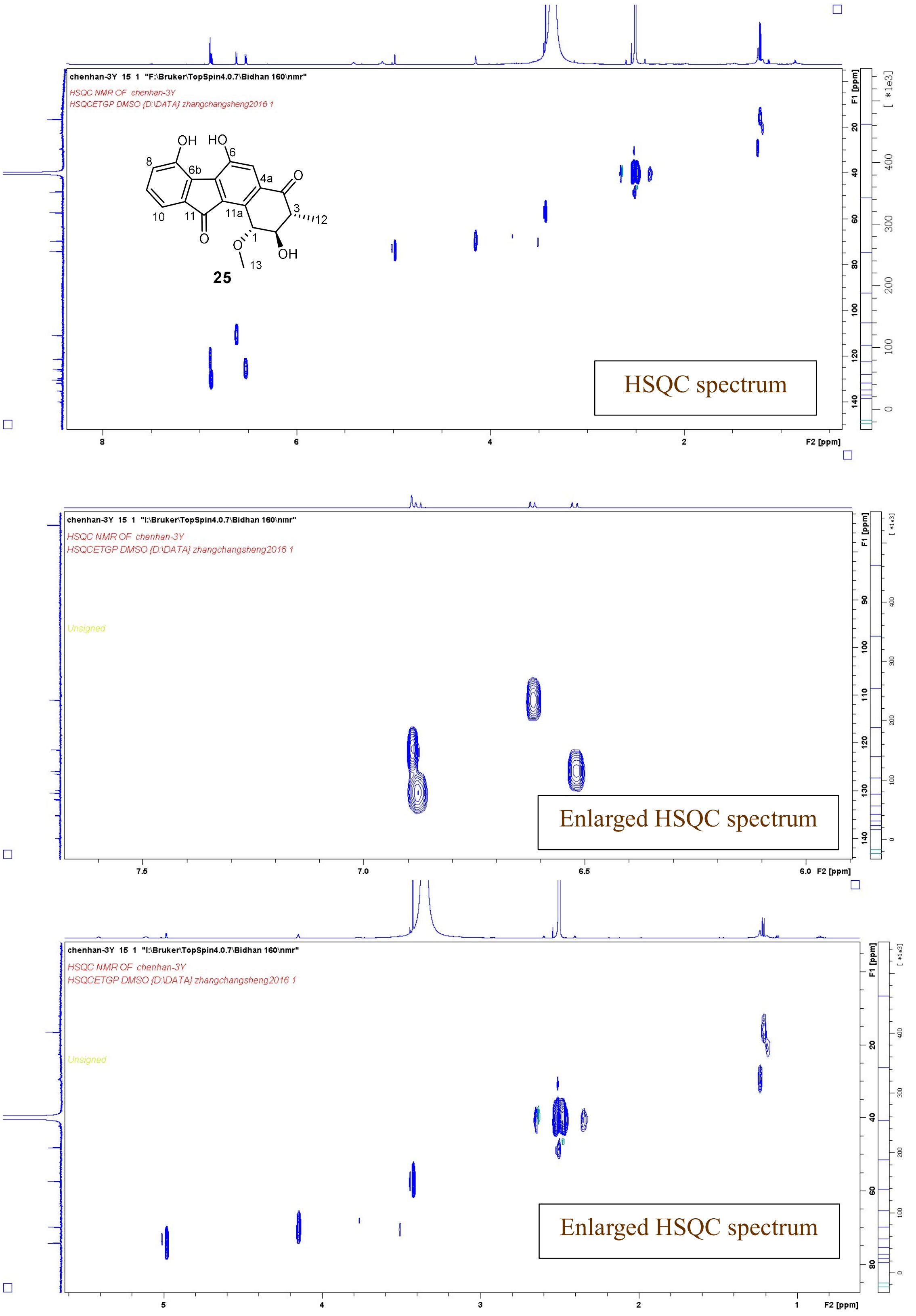

Supplementary Fig.32. Spectroscopic data for 25. (**d**) The HSQC and enlarged HSQC spectrum of **25** in DMSO-d_6.

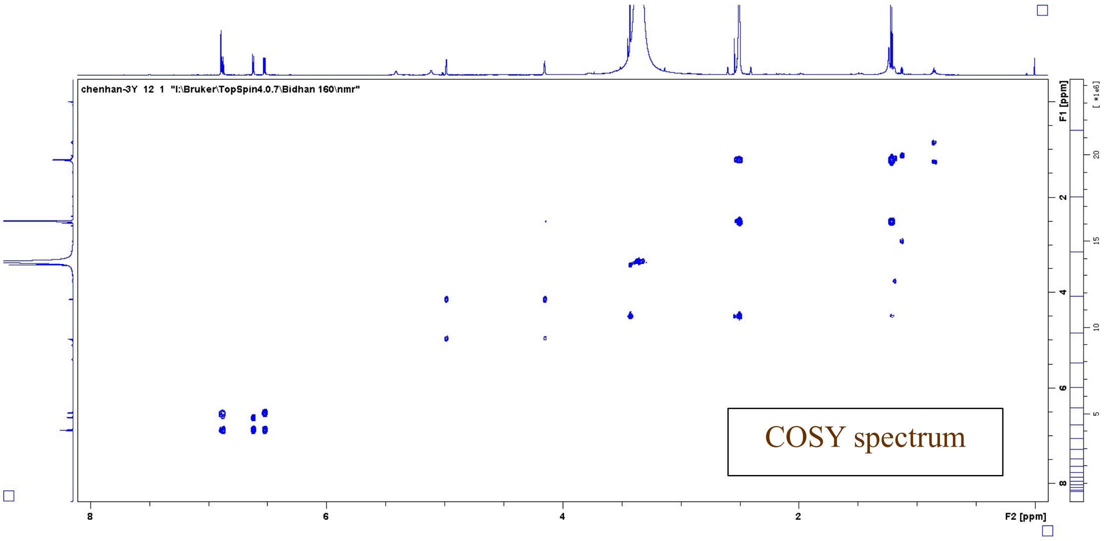

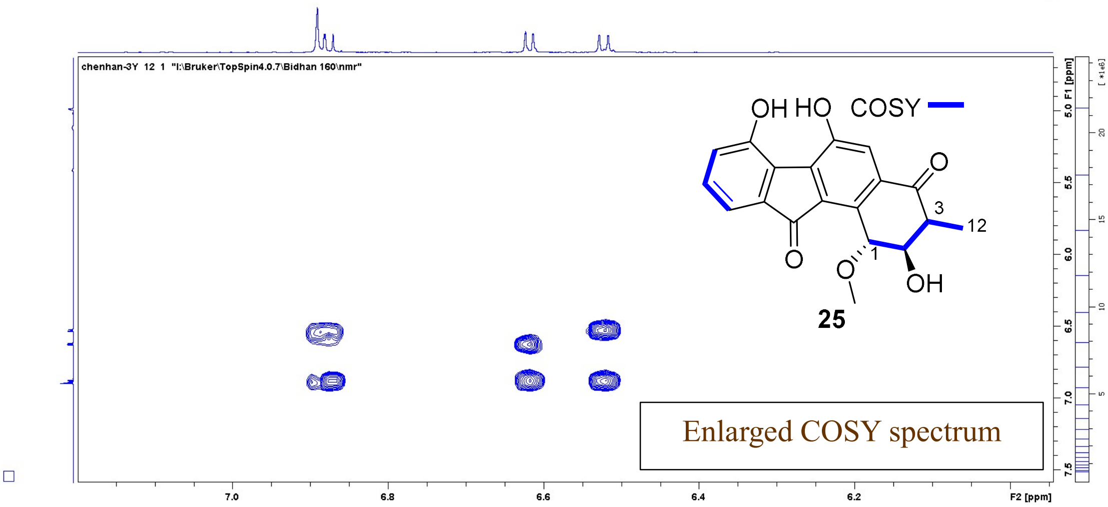

Supplementary Fig.32. Spectroscopic data for 25. (**e**) The COSY and enlarged COSY spectrum of **25** in DMSO-d_6.

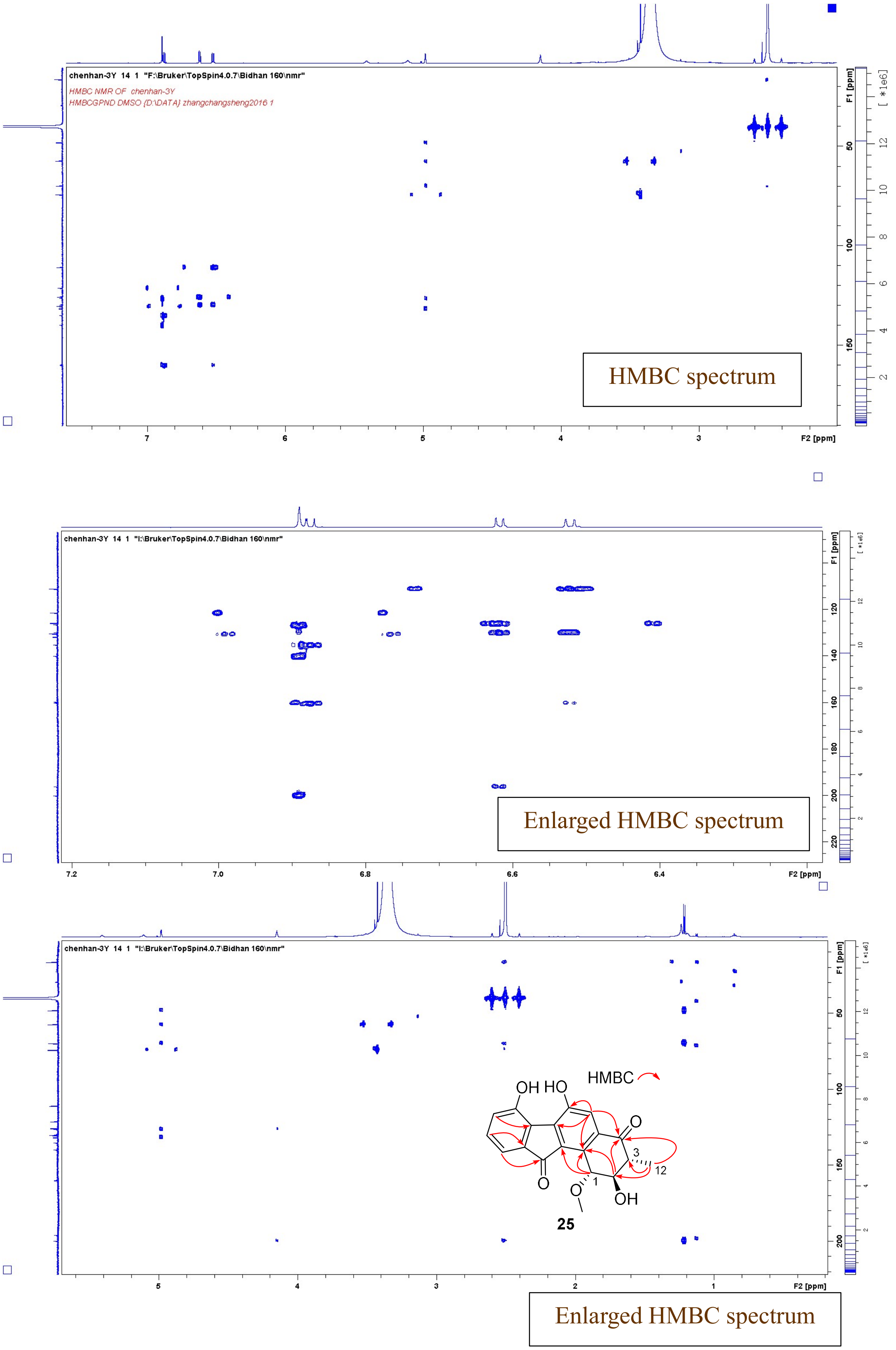

Supplementary Fig.32. Spectroscopic data for 25. (f) The HMBC and enlarged HMBC spectrum of **25** in DMSO-d_6.

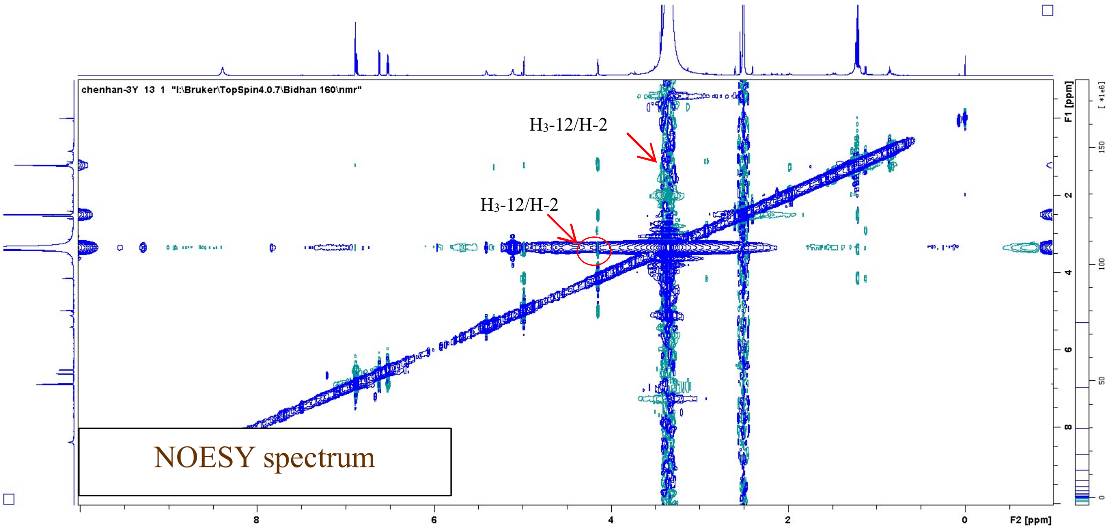

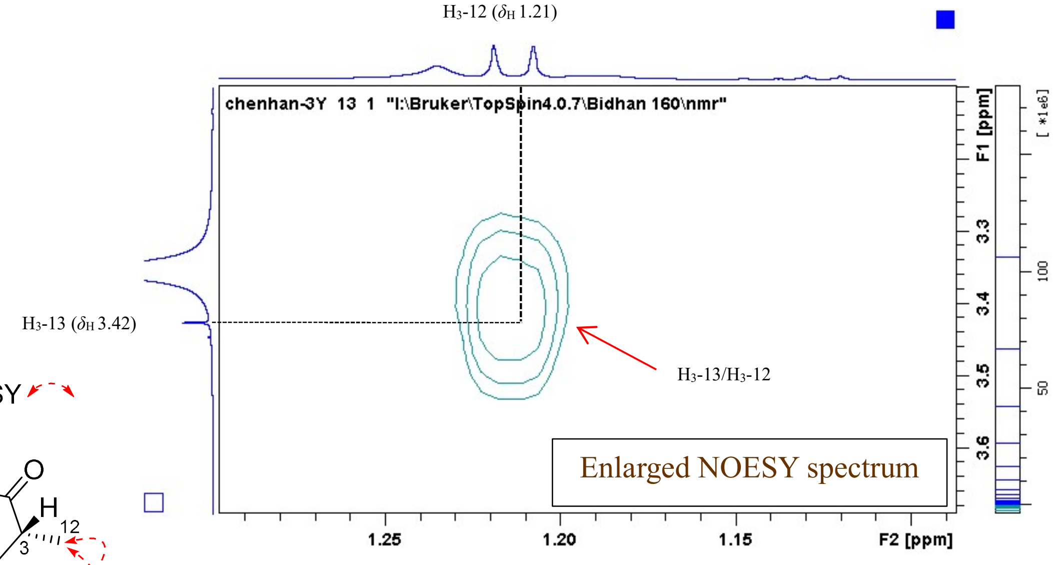

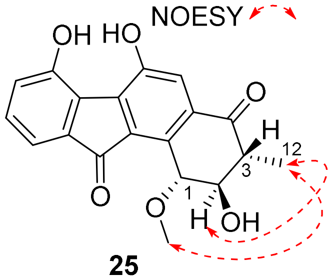

25

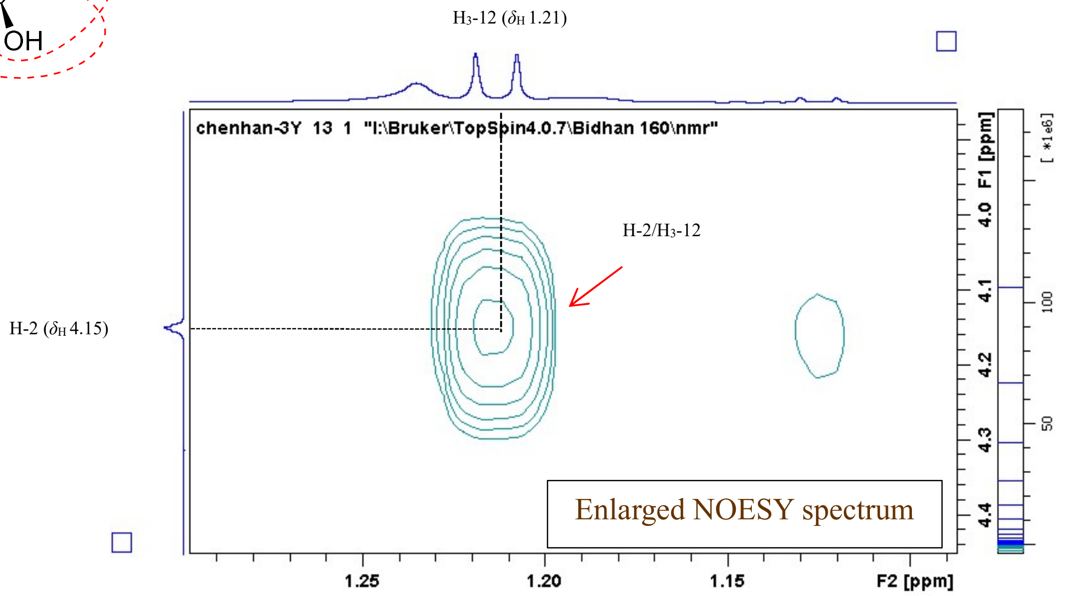

Supplementary Fig.32. Spectroscopic data for 25. (g) The NOESY and enlarged NOESY spectrum of **25** in DMSO-d_6.

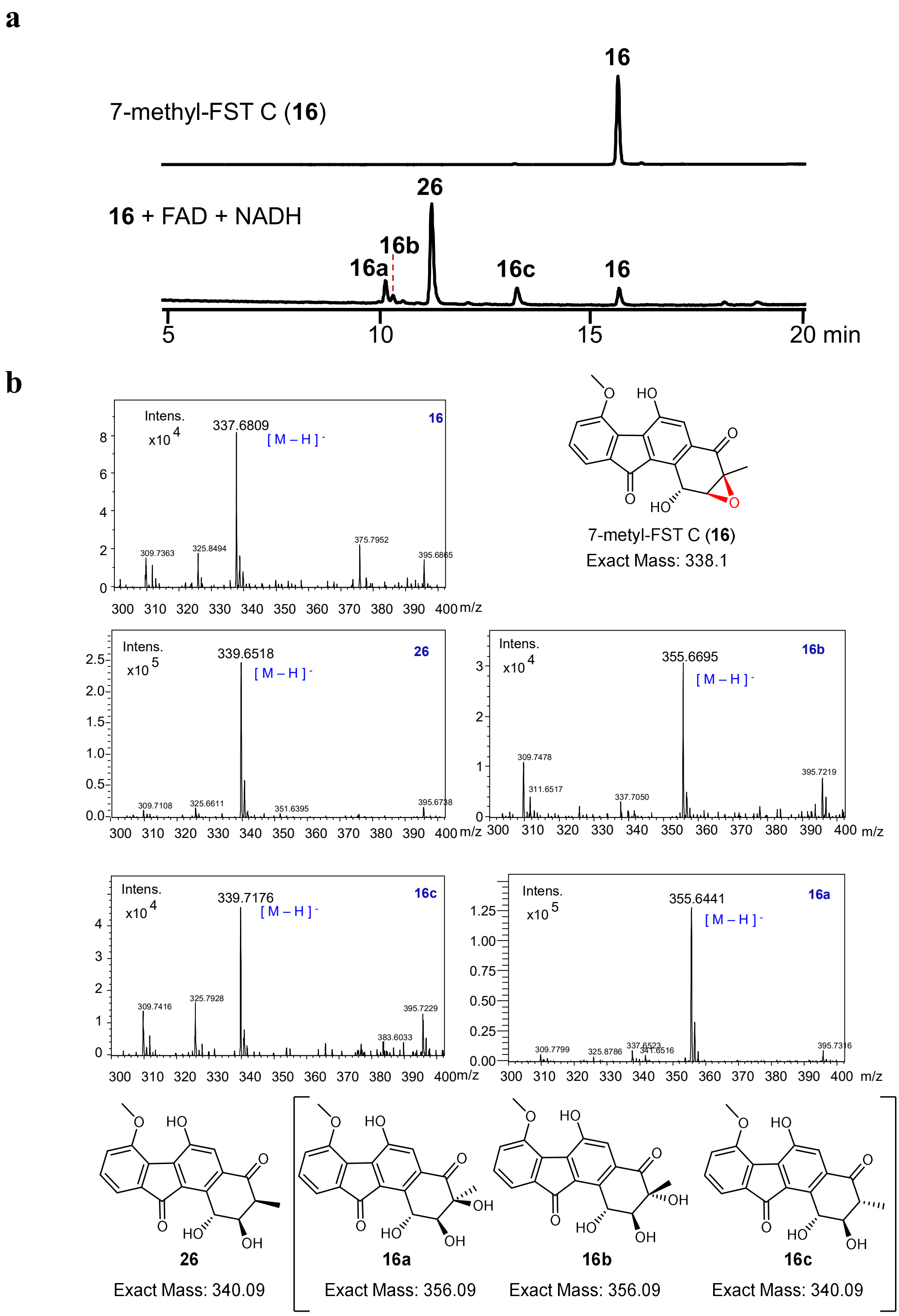

Supplementary Fig. 33. HPLC and LC-MS analysis of 7-methyl-FST C (16) reaction with FAD/NADH. (**a**) HPLC analysis of **16** standard and a reaction mixture containing 100 μM **16**, 100 μM FAD and 10 mM NADH in 50 mM PBS buffer (pH 7). The reaction mixtures were incubated at 30 ºC for 30 min. (**b**) LC-MS analysis of 7-methyl-FST C (**16**) reaction products with putative structures.

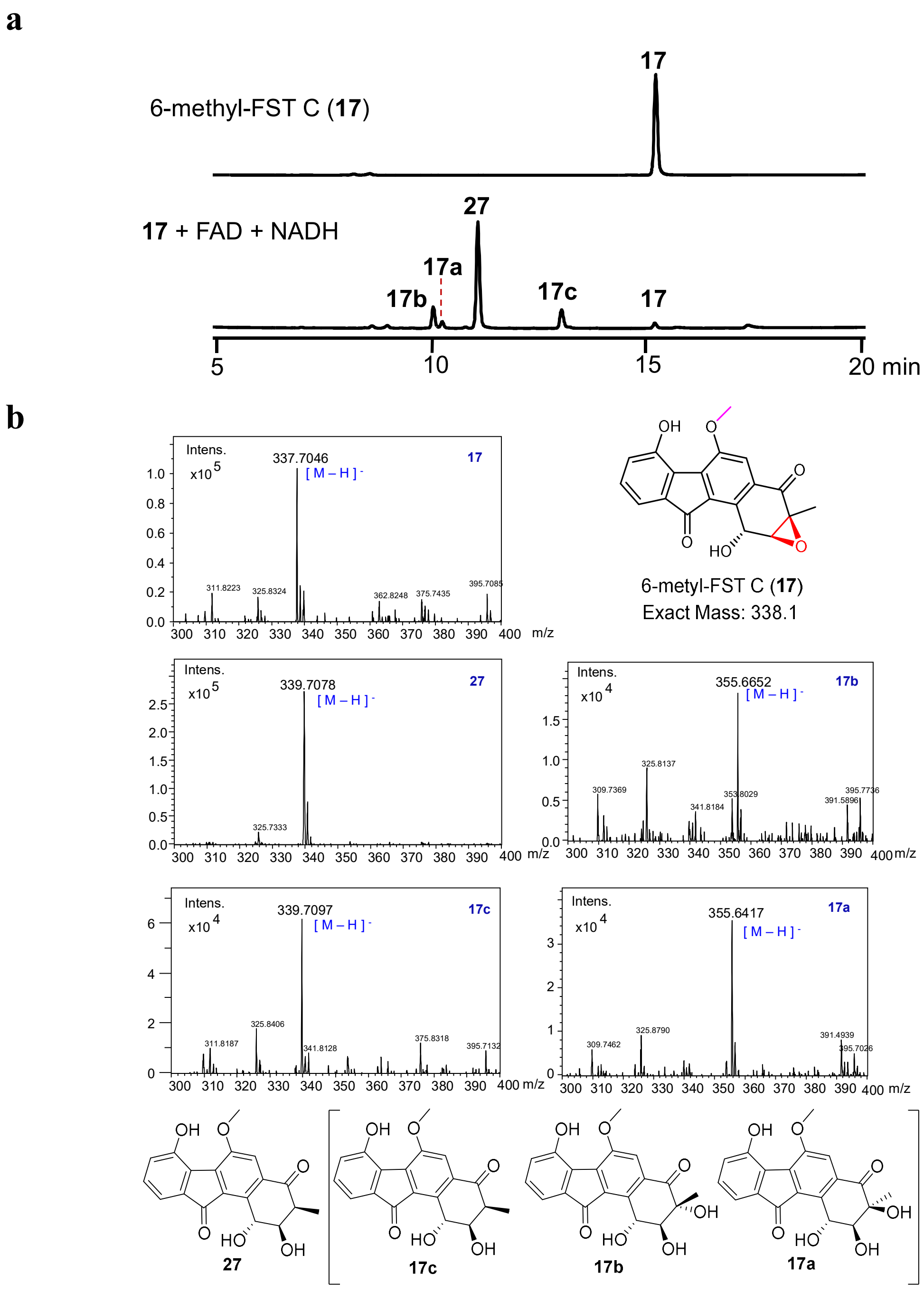

Supplementary Fig. 34. HPLC and LC-MS analysis for 6-methyl-FST C (17) reaction with FAD/NADH. (**a**) HPLC analysis of **17** standard and a reaction mixture containing 100 μM **17**, 100 μM FAD and 10 mM NADH in 50 mM PBS buffer (pH 7). The reaction mixtures were incubated at 30 °C for 30 min. (**b**) LC-MS analysis of 6-methyl-FST C (**17**) reaction products with putative structures.

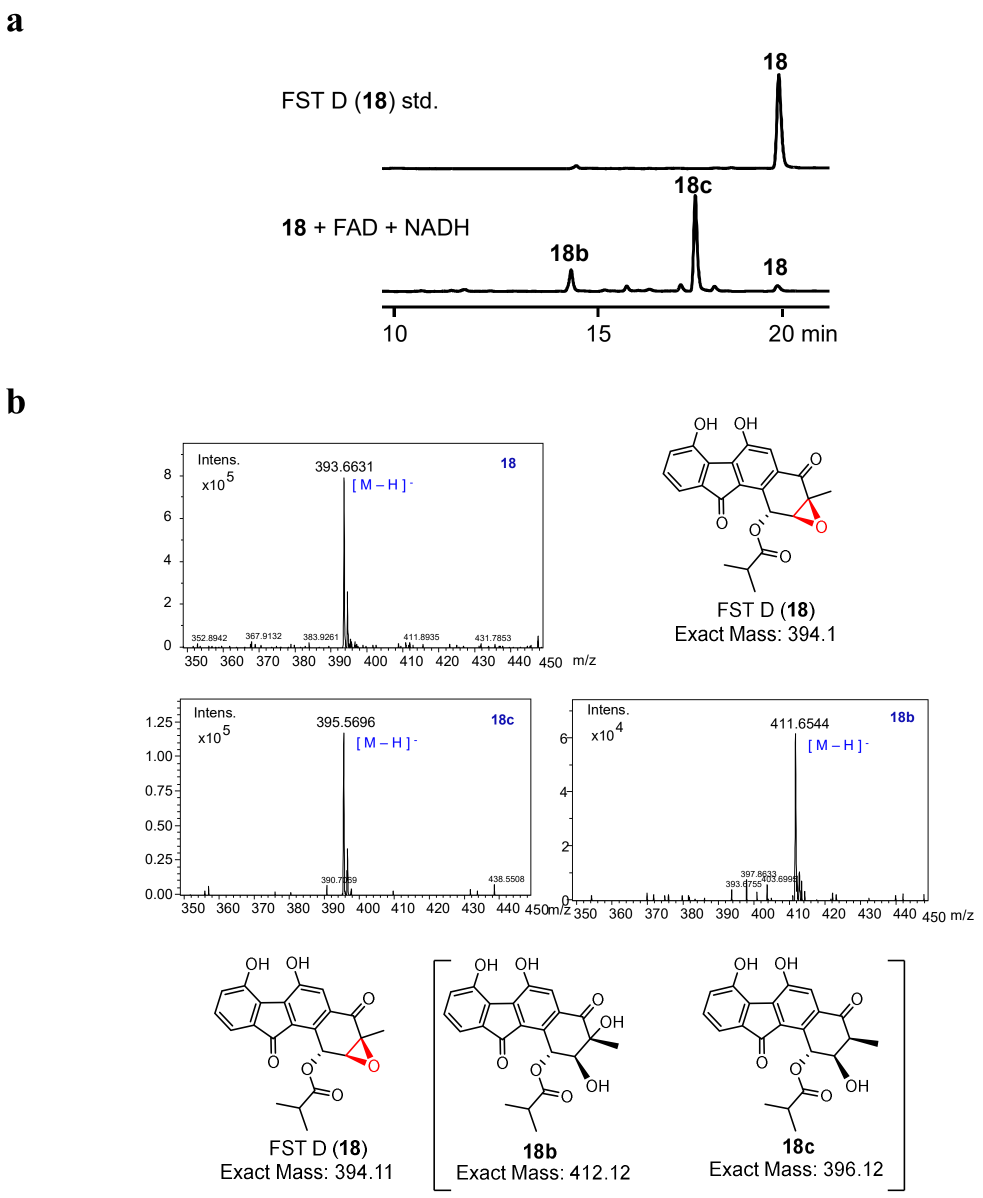

Supplementary Fig. 35. HPLC and LC-MS analysis of FST D (18) reaction with FAD/NADH. (**a**) HPLC analysis of **17** standard and a reaction mixture containing 100 µM **18**, 100 µM FAD and 10 mM NADH in 50 mM PBS buffer (pH 7). The reaction mixtures were incubated at 30 °C for 30 min. (**b**) LC-MS analysis of FST D (**18**) reaction products with putative structures.

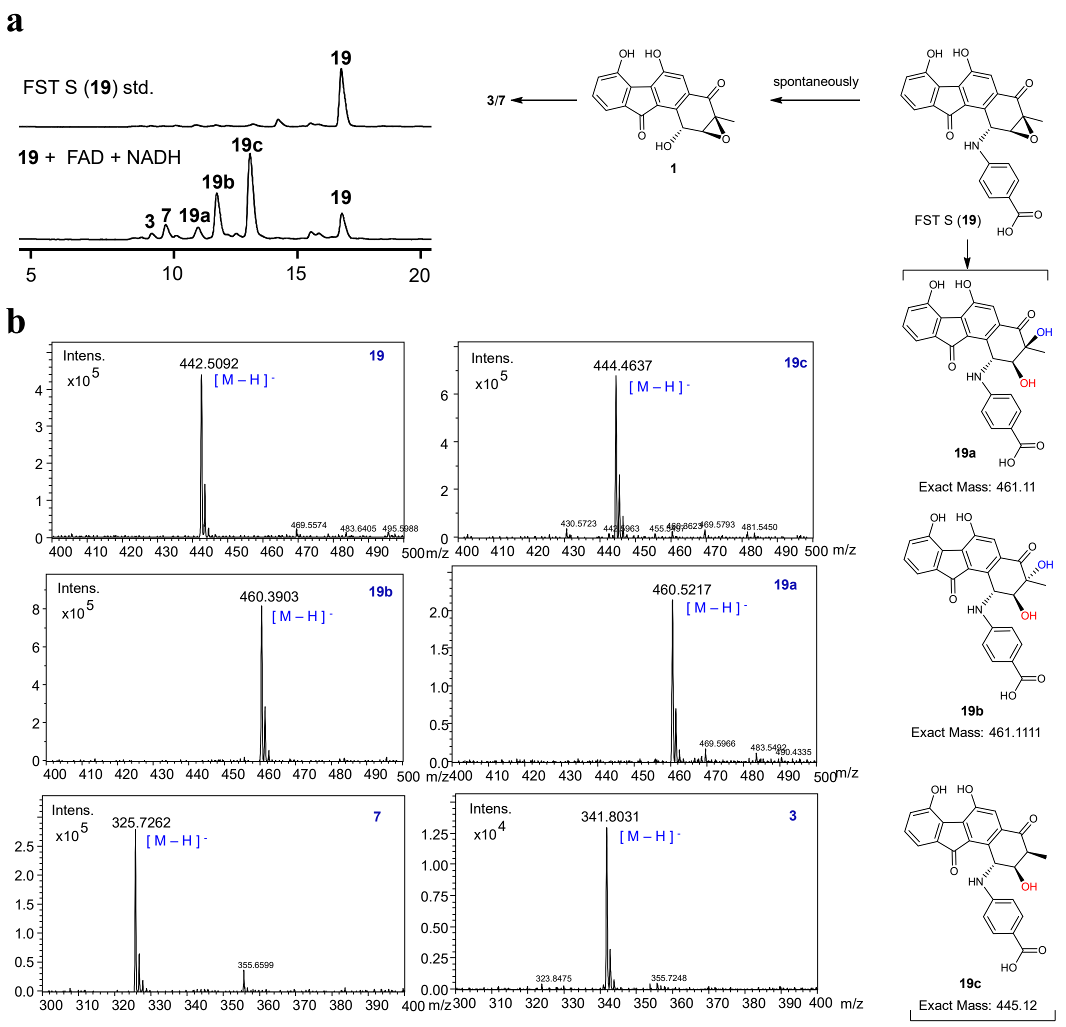

Supplementary Fig. 36. HPLC and LC-MS analysis of FST S (19) reaction with FAD/NADH. (**a**) HPLC analysis of **19** standard and a reaction mixture containing 100 μM **19**, 100 μM FAD and 10 mM NADH in 50 mM PBS buffer (pH 7). The reaction mixtures were incubated at 30 °C for 30 min. (**b**) LC-MS analysis of FST S (**19**) reaction products with putative structures. The spontaneously conversion of **19** to **1** has been previously reported.

a

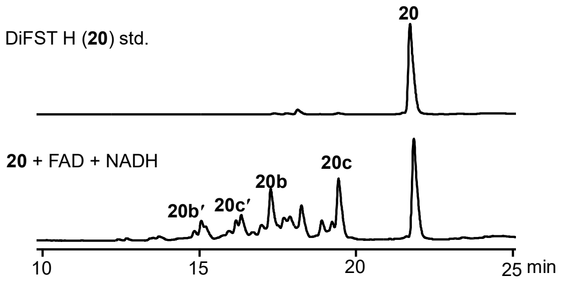

b

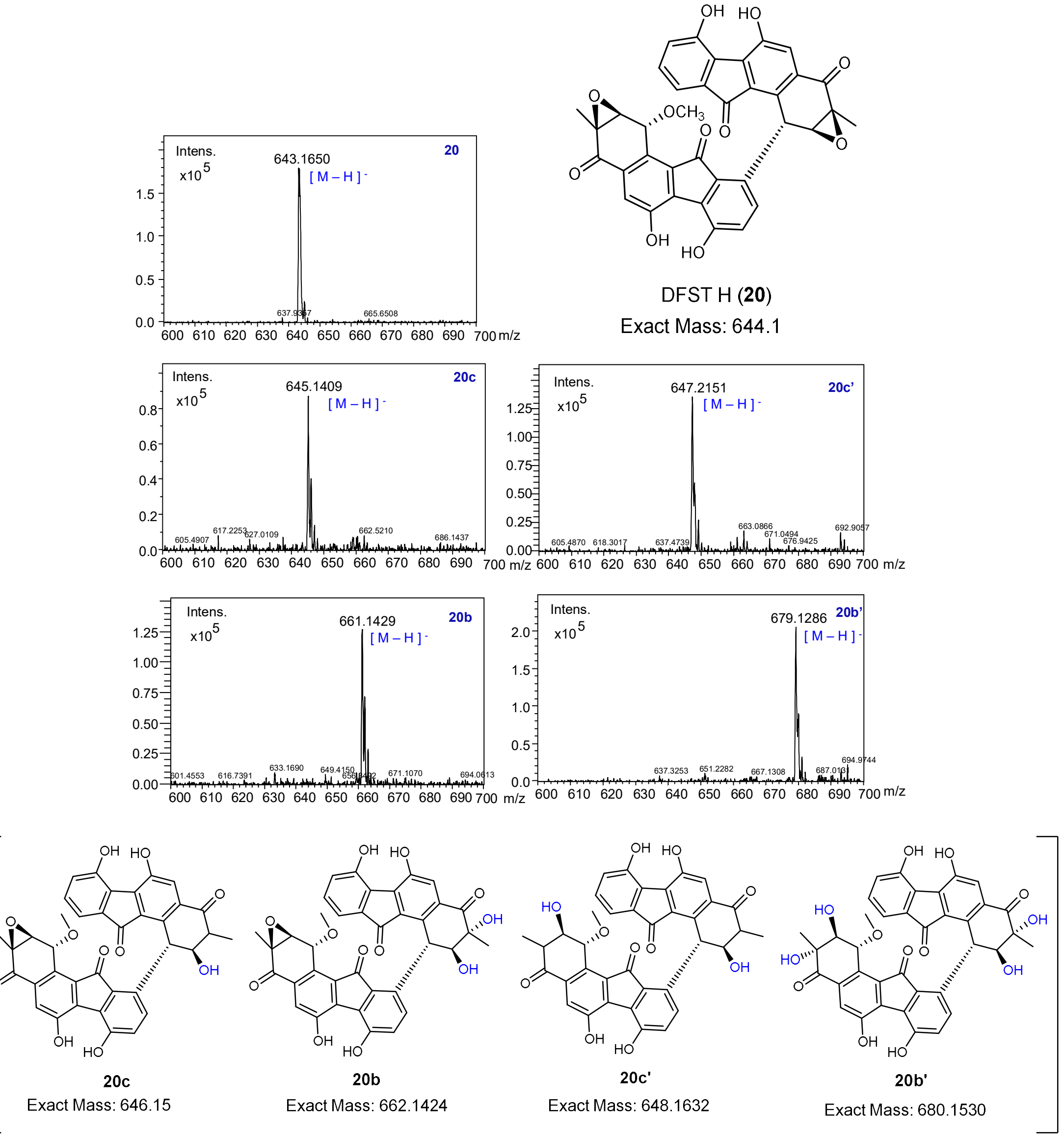

Supplementary Fig. 37. HPLC and LC-MS analysis of DiFST H (20) reaction with FAD/NADH. (**a**) HPLC analysis of **20** standard and a reaction mixture containing 100 μM **20**, 100 μM FAD and 10 mM NADH in 50 mM PBS buffer (pH 7). The reaction mixtures were incubated at 30 °C for 30 min. (**b**) LC-MS analysis of DiFST H (**20**) reaction products with putative structures.

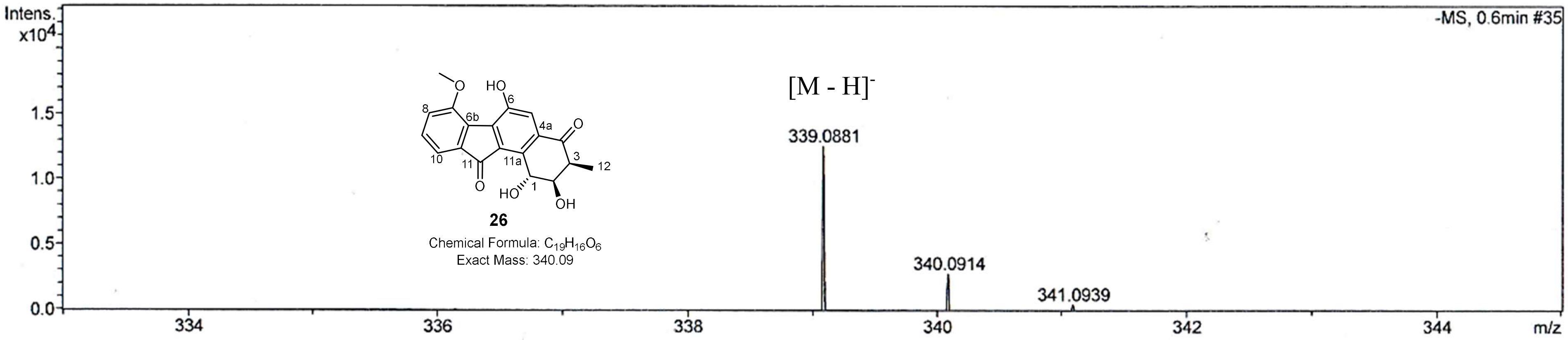

Meas. m/z	#	Ion Formula	Score	m/z	err [ppm]	err [mDa]	mSigma	rdb	e⁻ Conf	N-Rule
339.0881	1	C19H15O6	100.00	339.0874	-2.0	-0.7	6.8	12.5	even	ok
679.1831	1	C38H31O12	100.00	679.1821	-1.5	-1.0	22.3	23.5	even	ok

Supplementary Fig. 38. Spectroscopic data for 26. (**a**) HRESIMS spectrum.

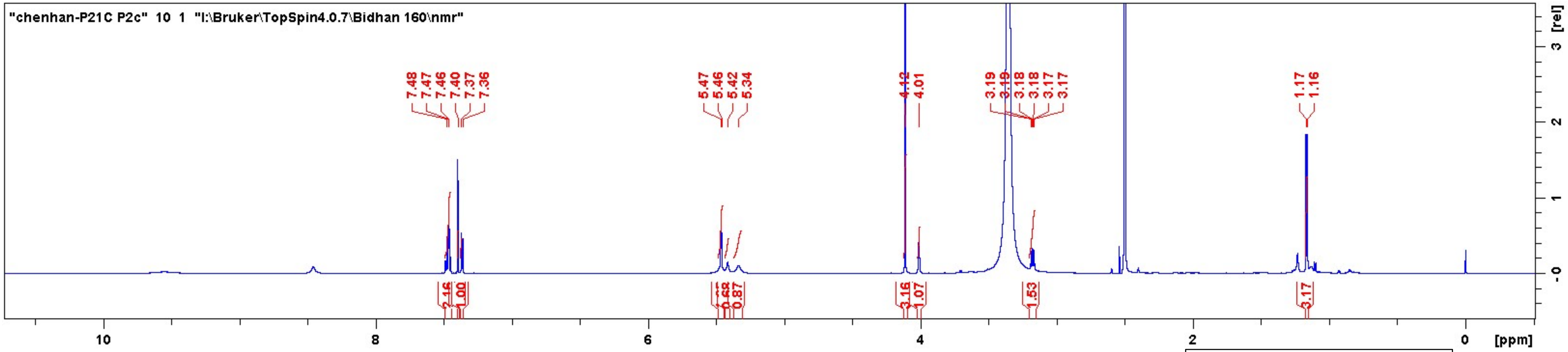

^{1}H NMR spectrum

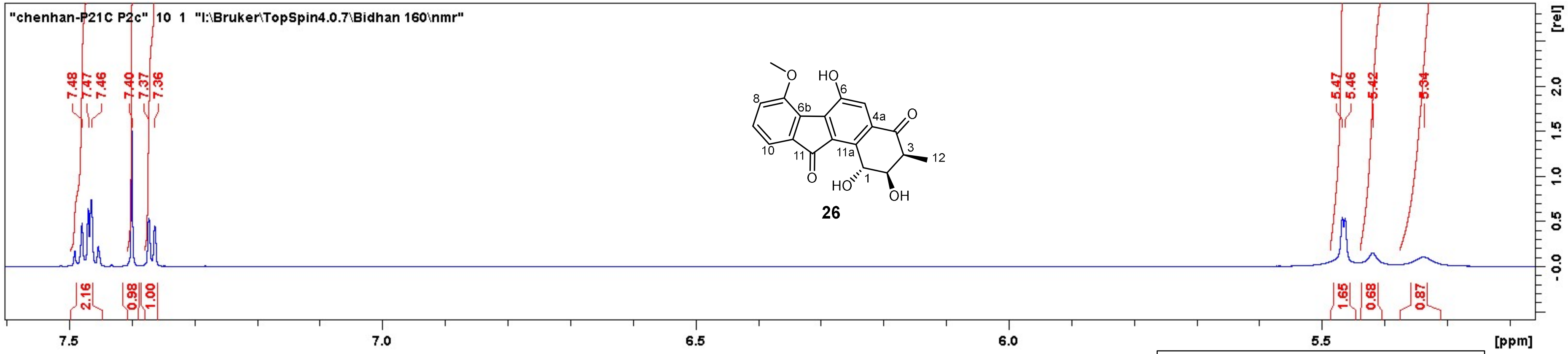

Enlarged ^{1}H NMR spectrum

Supplementary Fig. 38. Spectroscopic data for 26. (**b**) The ^{1}H and enlarged ^{1}H NMR spectrum of **26** in DMSO-d_6.

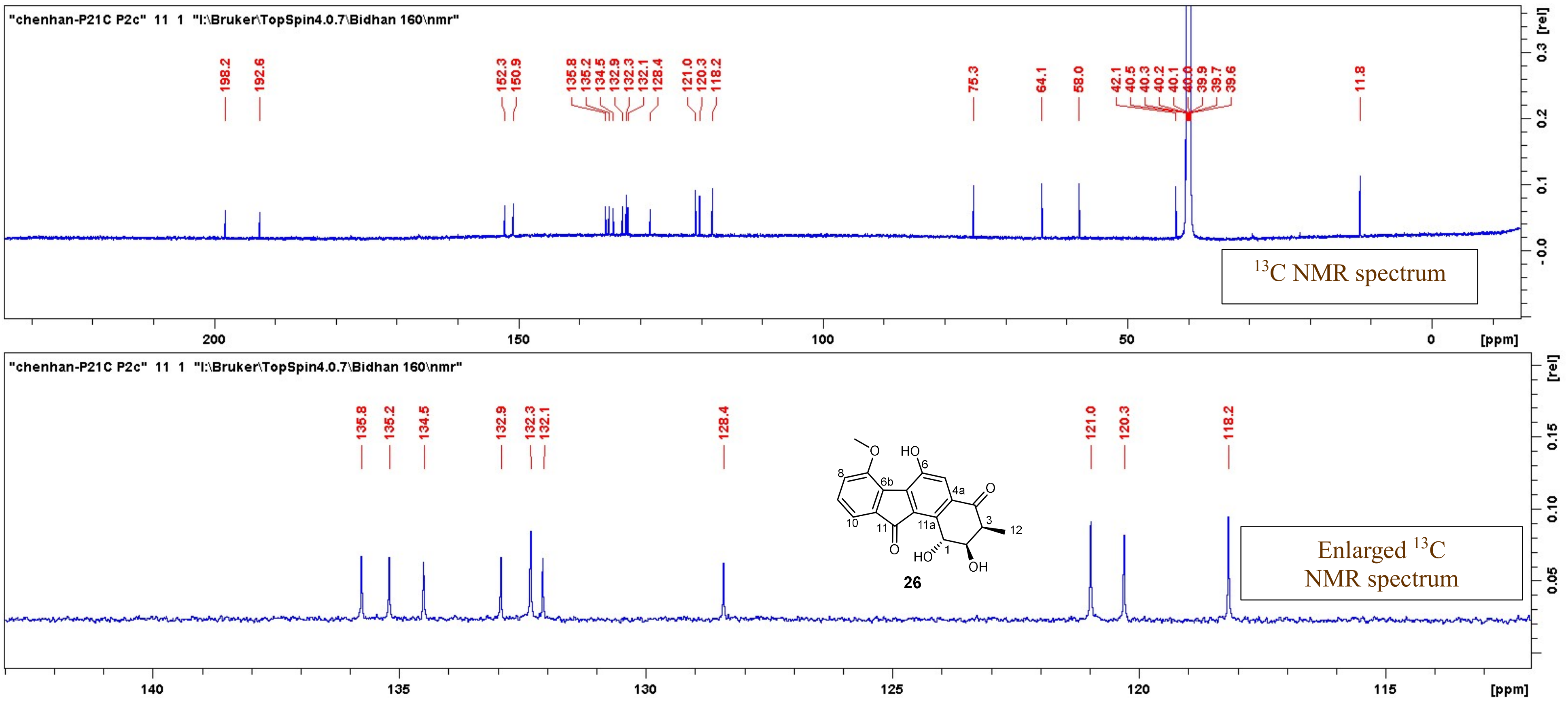

Supplementary Fig. 38. Spectroscopic data for 26. (**c**) The ^{13}C and enlarged ^{13}C NMR spectrum of **26** in DMSO-d_6.

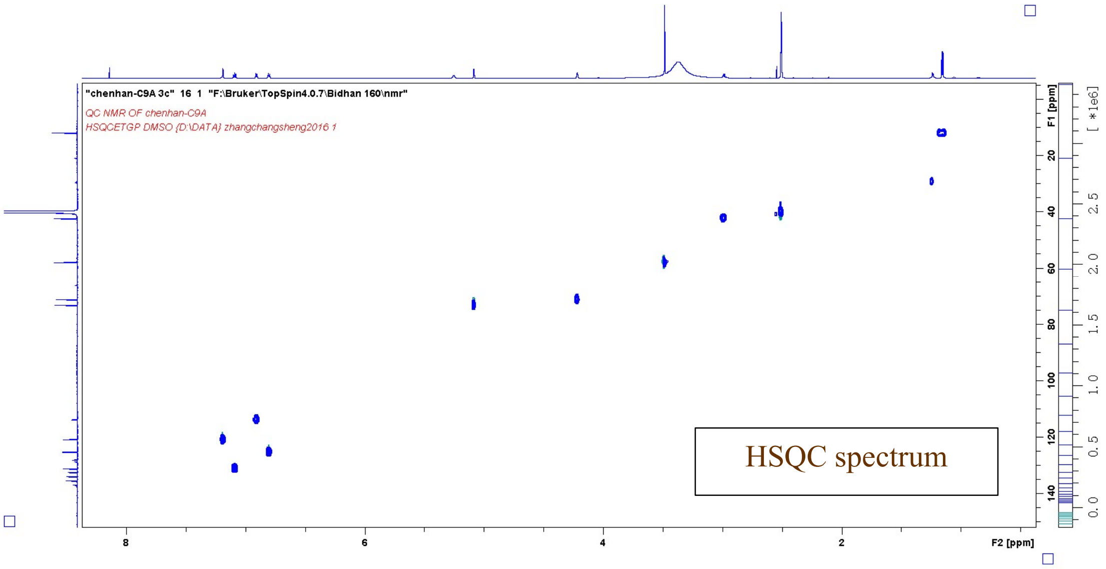

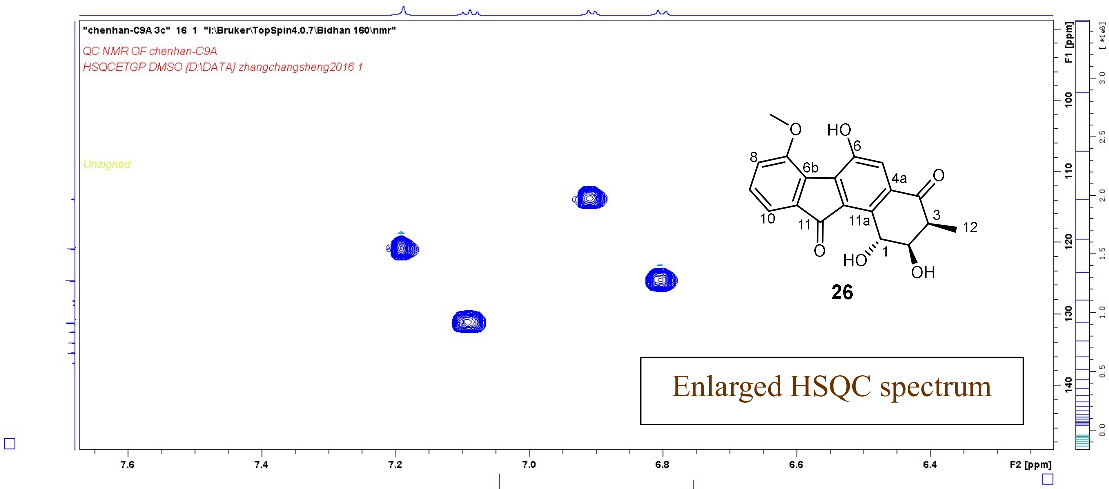

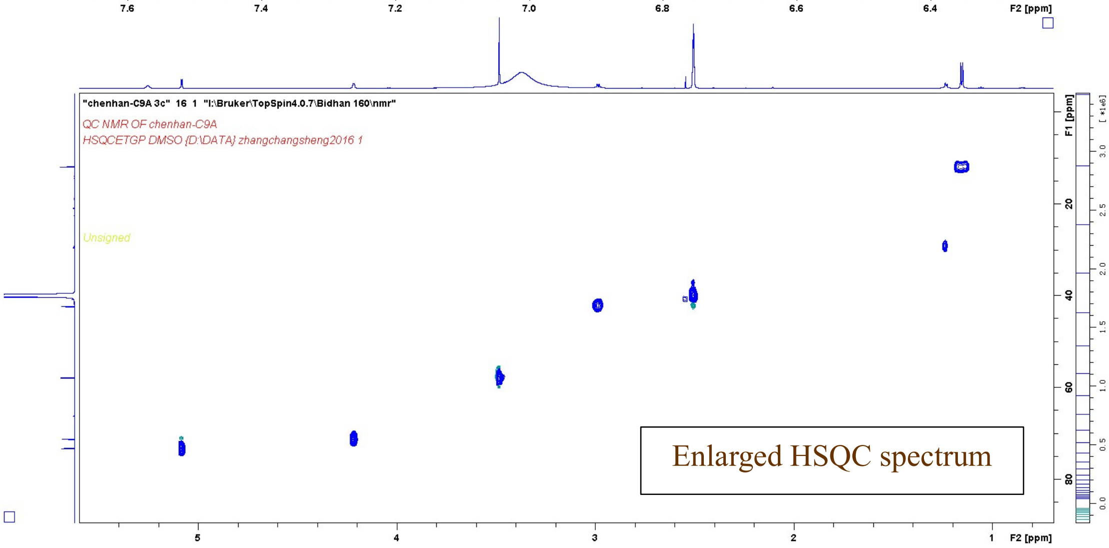

Supplementary Fig. 38. Spectroscopic data for 26. (**d**) The HSQC and enlarged HSQC spectrum of **26** in DMSO-d_6.

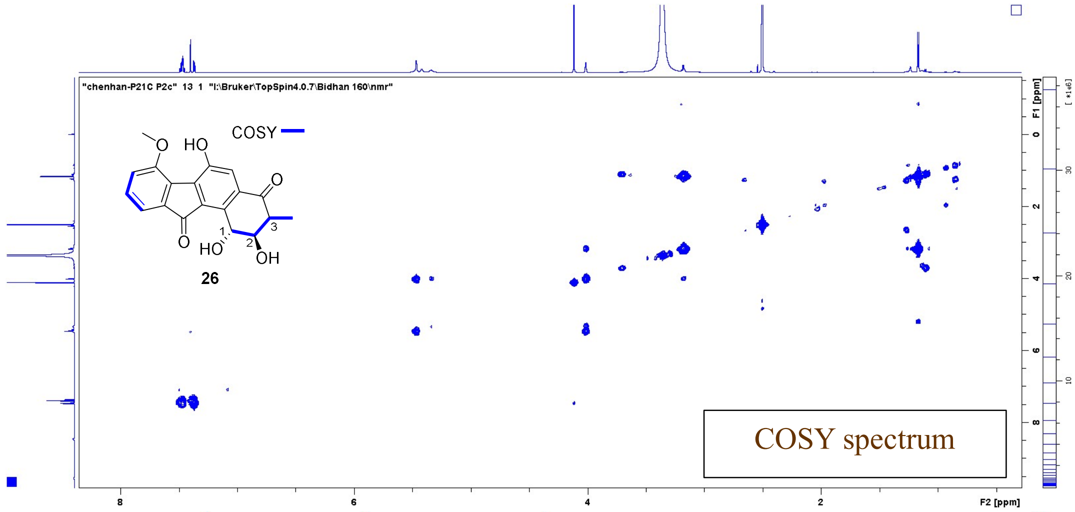

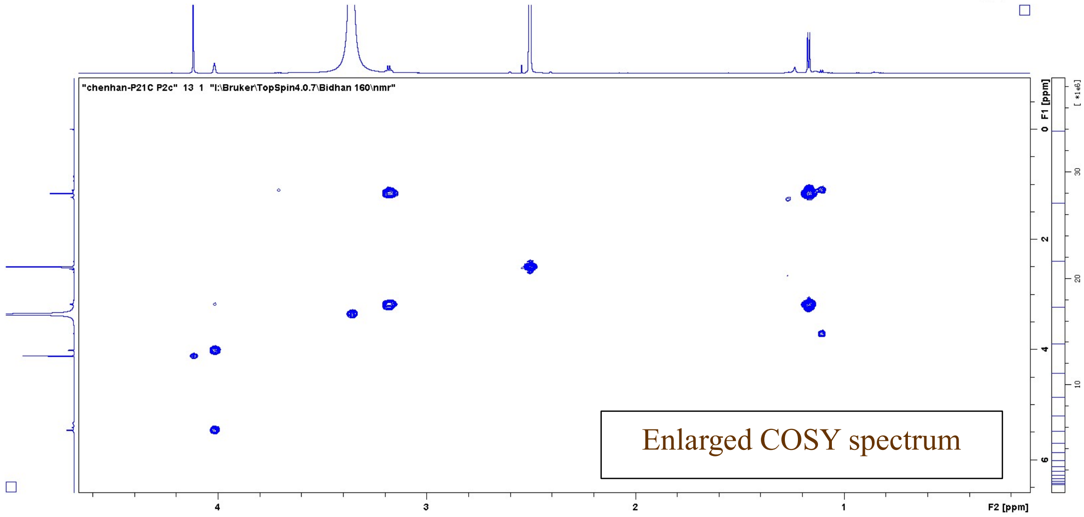

Supplementary Fig. 38. Spectroscopic data for 26 (**e**) The COSY and enlarged COSY spectrum of **26** in DMSO-d_6.

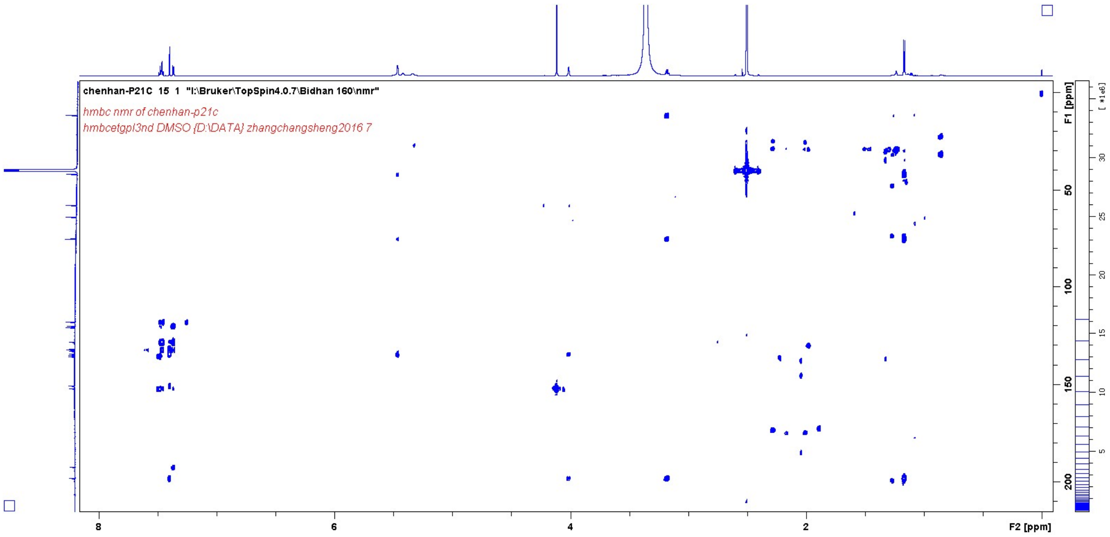

HMBC spectrum

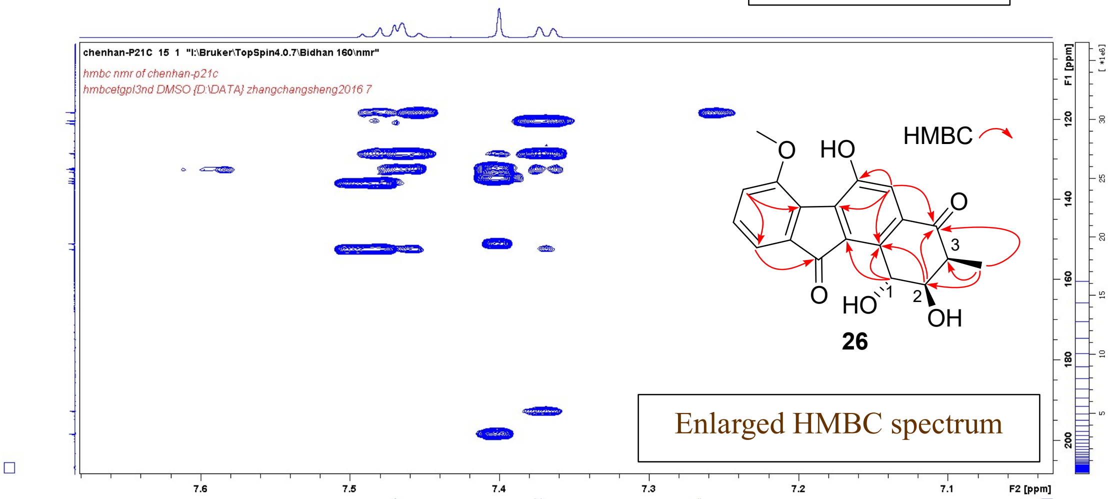

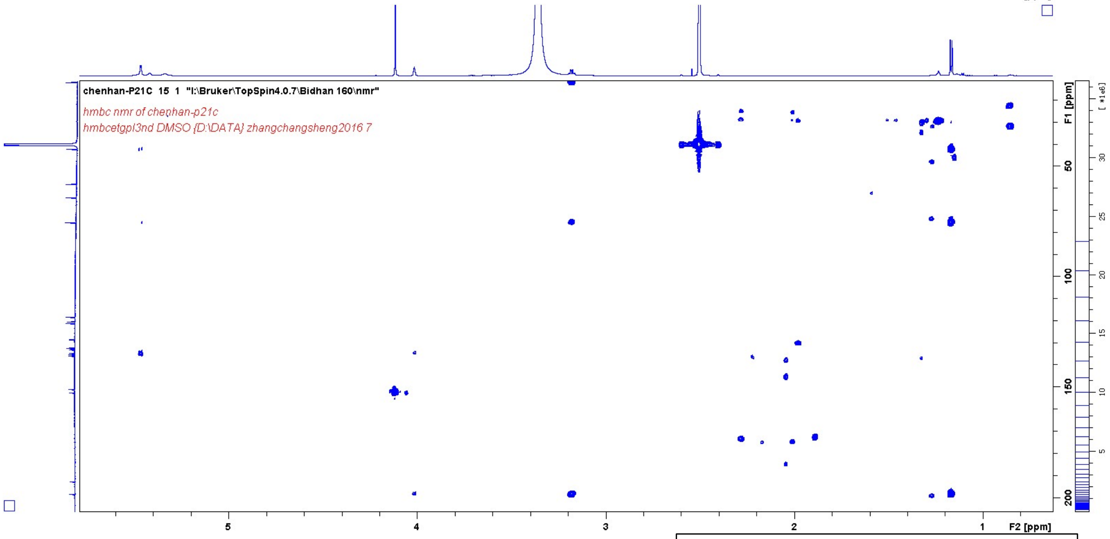

Enlarged HMBC spectrum

Supplementary Fig. 38. Spectroscopic data for 26. (**f**) The HMBC and enlarged HMBC spectrum of **26** in DMSO-d_6.

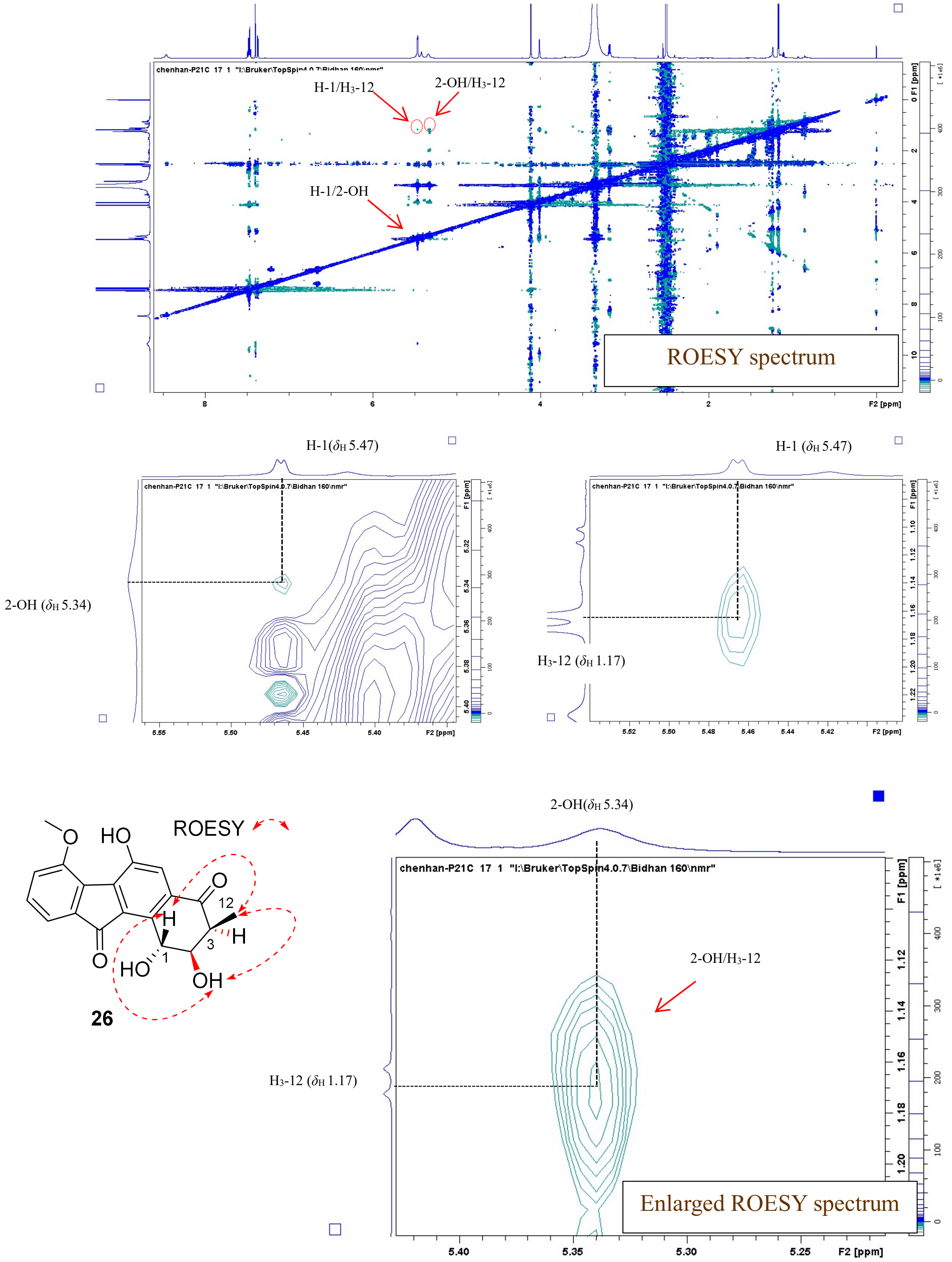

Supplementary Fig. 38. Spectroscopic data for 26. **(g)** The ROESY and enlarghed ROESY spectrum of **26** in DMSO-d_6.

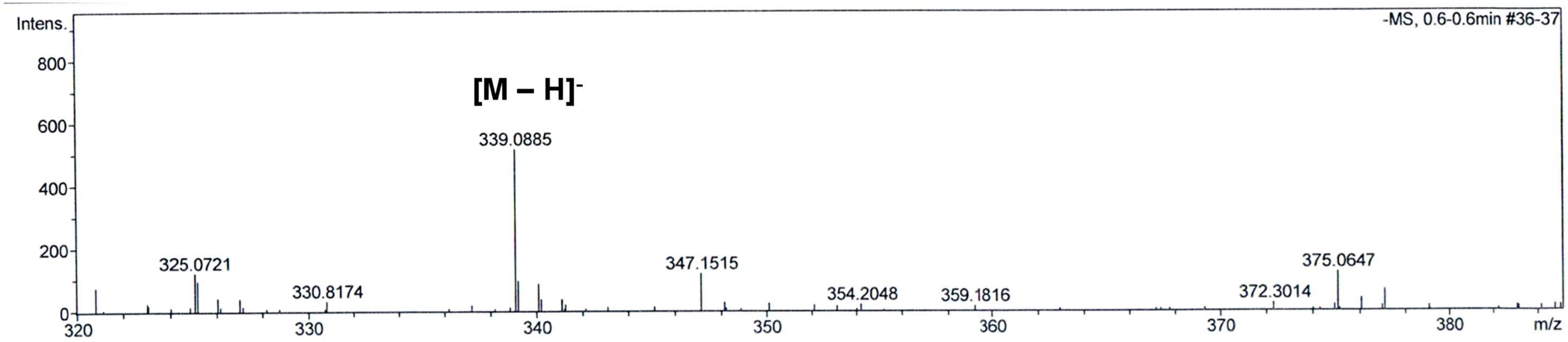

Meas. m/z	#	Ion Formula	Score	m/z	err [ppm]	err [mDa]	mSigma	rdb	e⁻ Conf	N-Rule
339.0885	1	C19H15O6	95.91	339.0874	-3.1	-1.1	25.4	12.5	even	ok
	2	C20H11N4O2	100.00	339.0887	-0.8	-0.3	39.6	17.5	even	ok

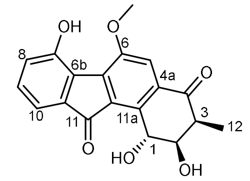

27

Chemical Formula: $C_{19}H_{16}O_6$

Exact Mass: 340.09

Supplementary Fig. 39. Spectroscopic data for 27. (**a**) HRESIMS spectrum.

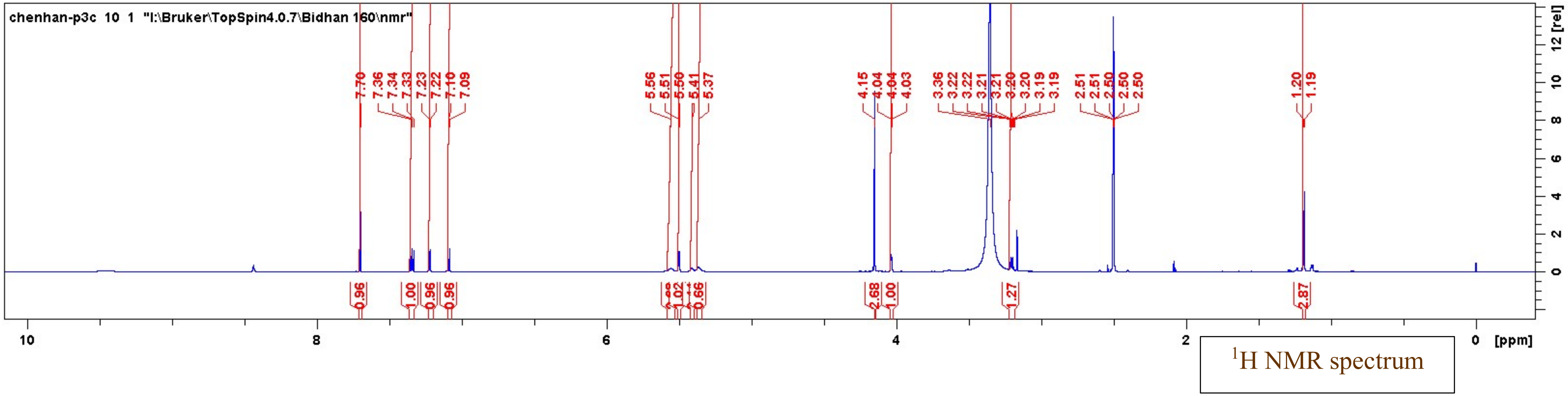

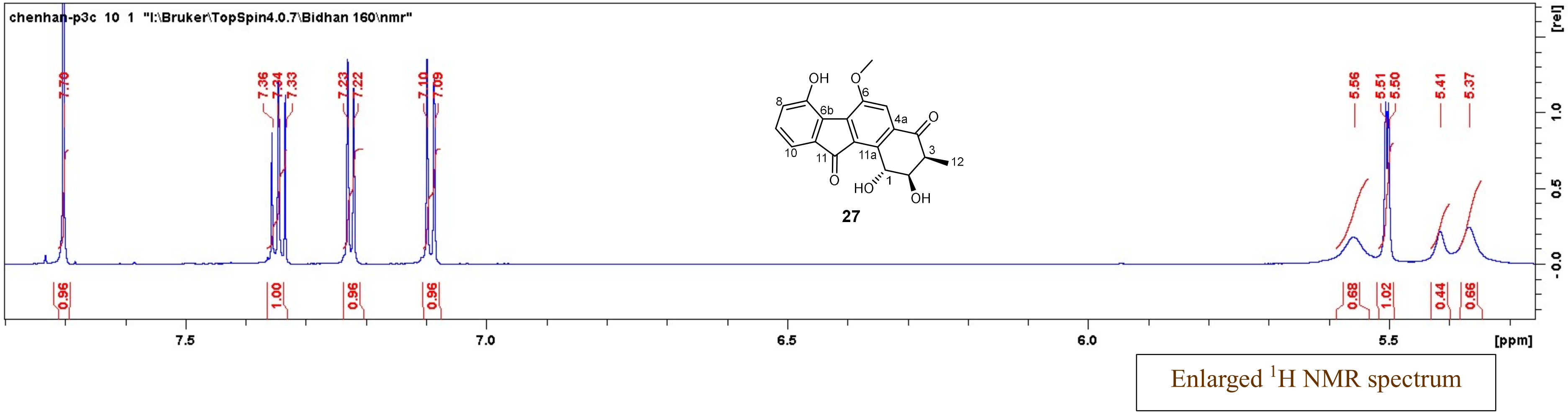

Supplementary Fig. 39. Spectroscopic data for 27. (b) The ^{1}H and enlarged ^{1}H NMR spectrum of **27** in DMSO-d_6.

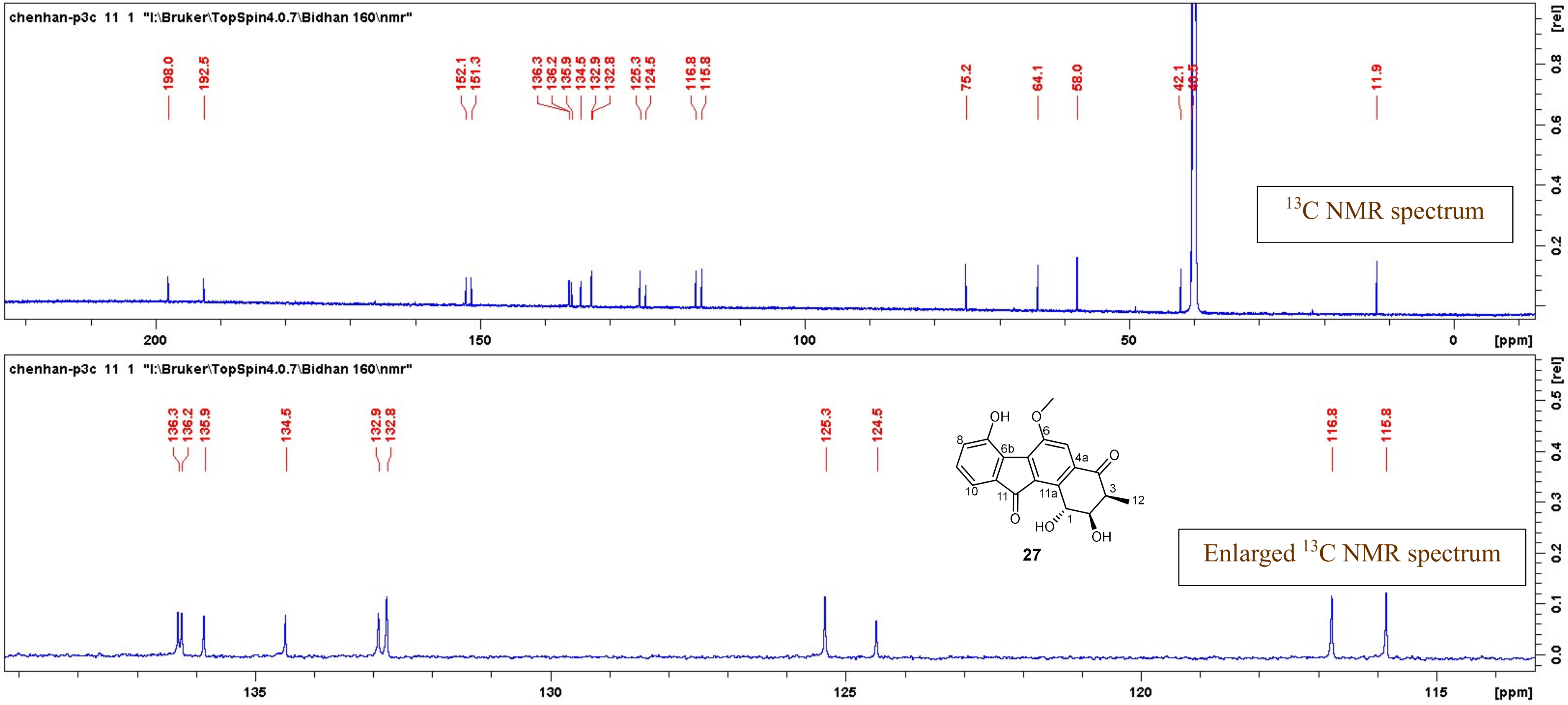

Supplementary Fig. 39. Spectroscopic data for 27. (**c**) The ^{13}C and enlarged ^{13}C NMR spectrum of **27** in DMSO-d_6.

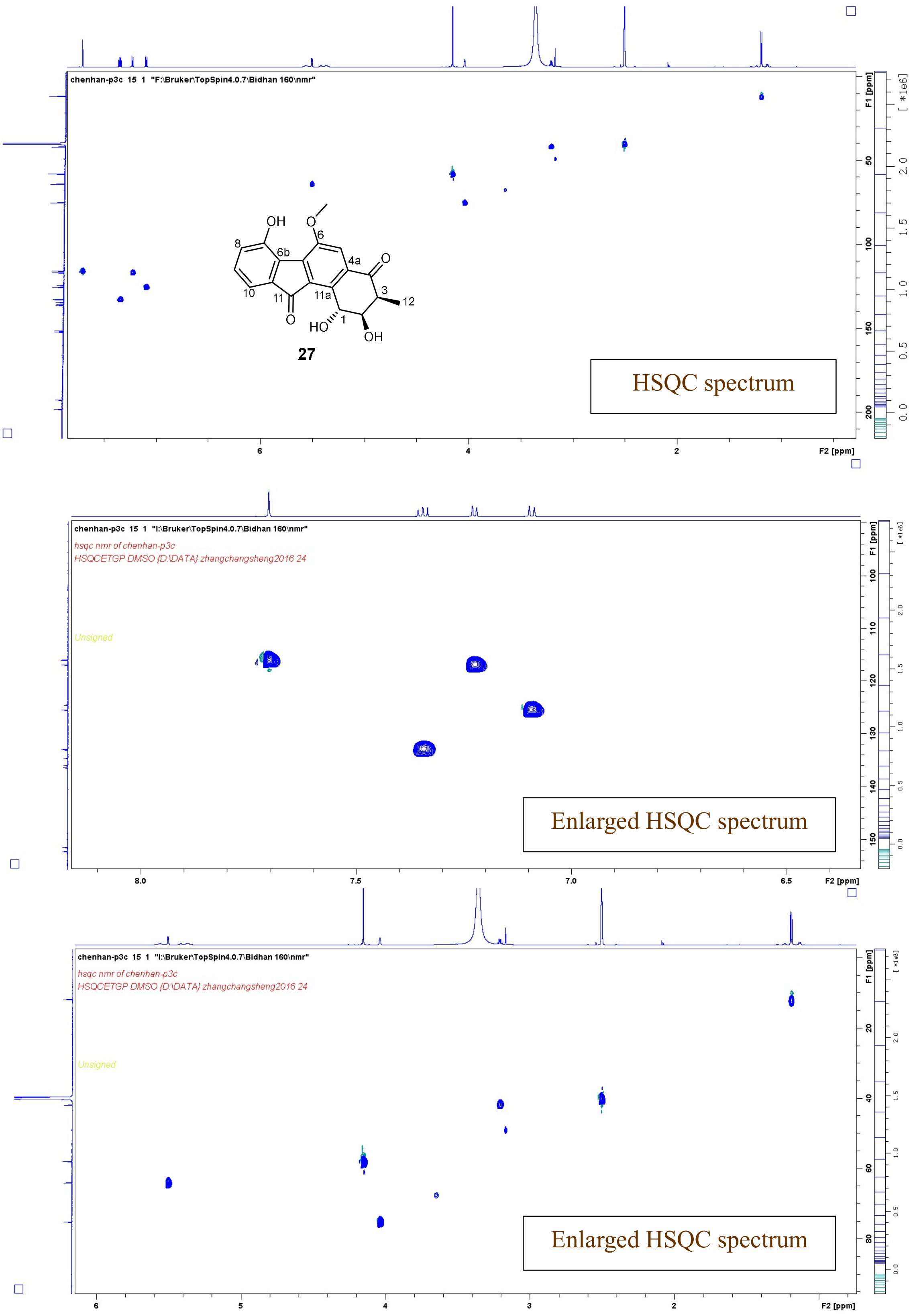

Supplementary Fig. 39. Spectroscopic data for 27. (**d**) The HSQC and enlarged HSQC spectrum of **27** in DMSO-d_6.

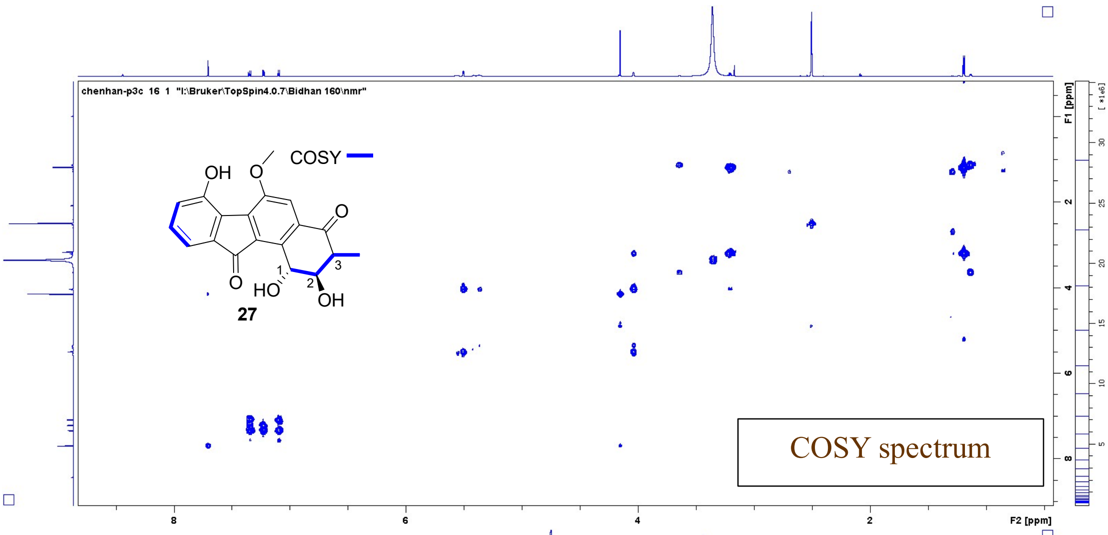

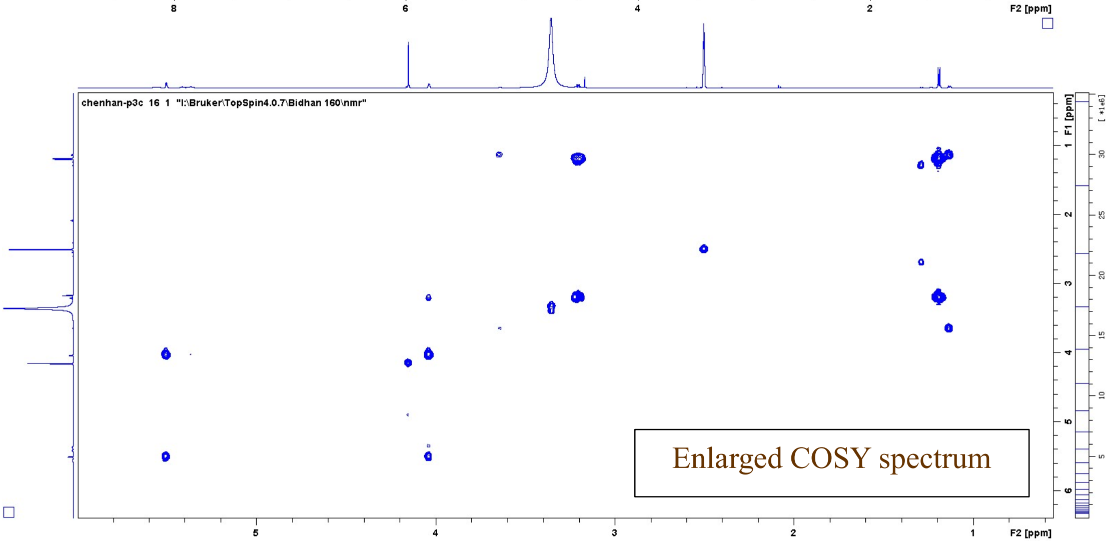

Supplementary Fig. 39. Spectroscopic data for 27. (**e**) The COSY and enlarged COSY spectrum of **27** in DMSO-d_6.

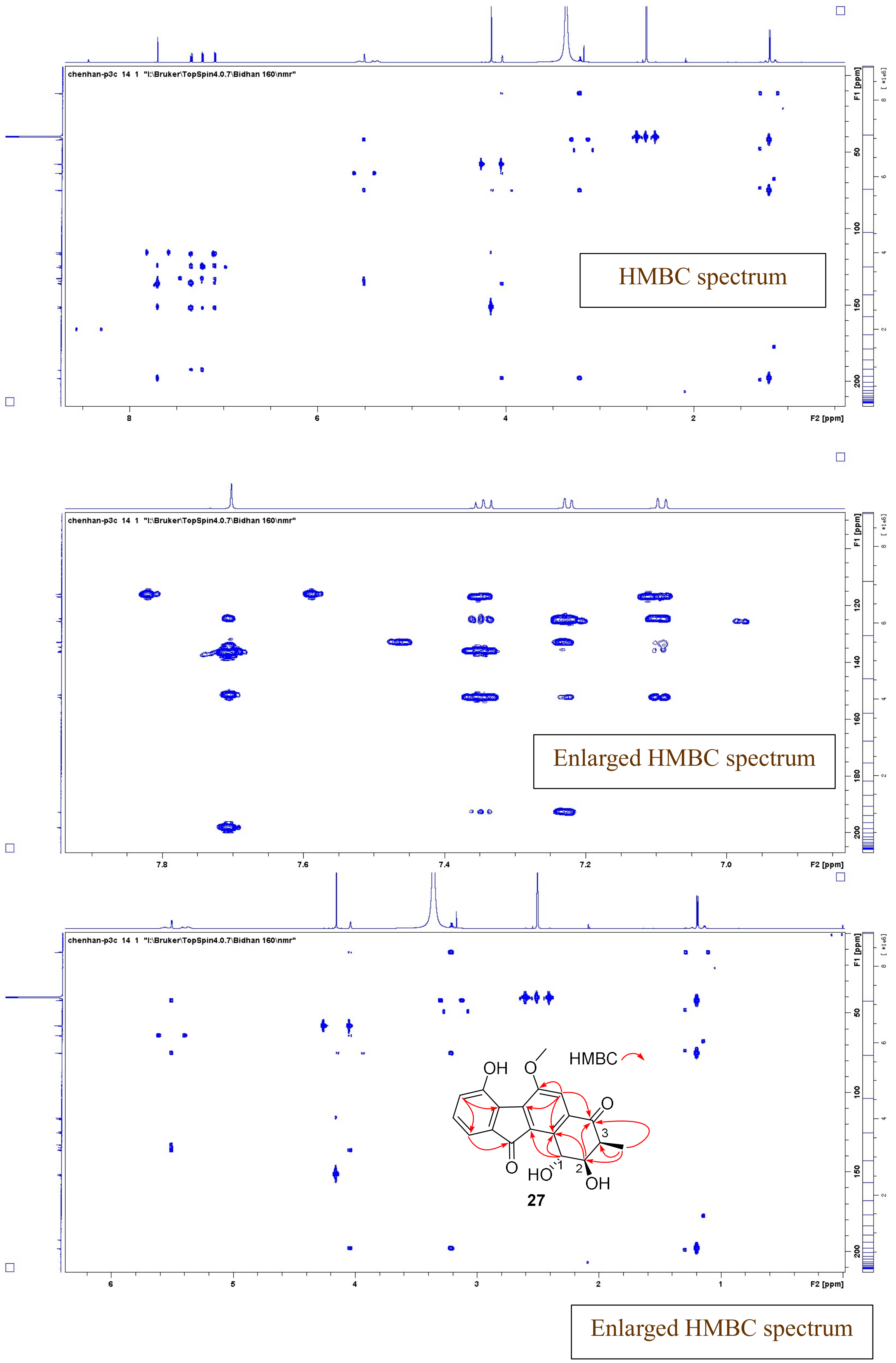

Supplementary Fig. 39. Spectroscopic data for 27. (**f**) The HMBC and enlarged HMBC spectrum of **27** in DMSO-d_6.

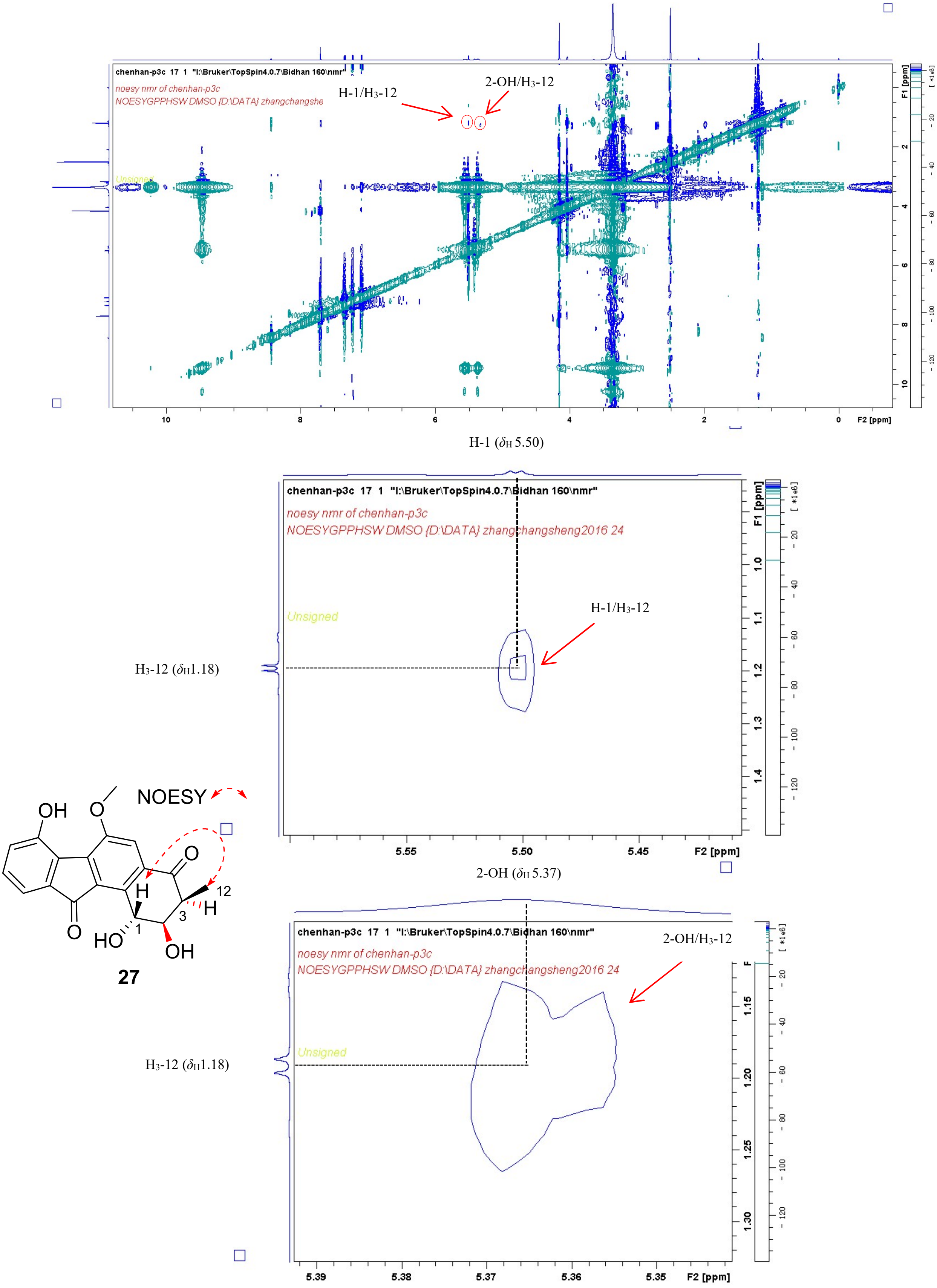

Supplementary Fig. 39. Spectroscopic data for 27. (g) The NOESY and enlarged NOESY spectrum of **27** in DMSO-d_6.

a

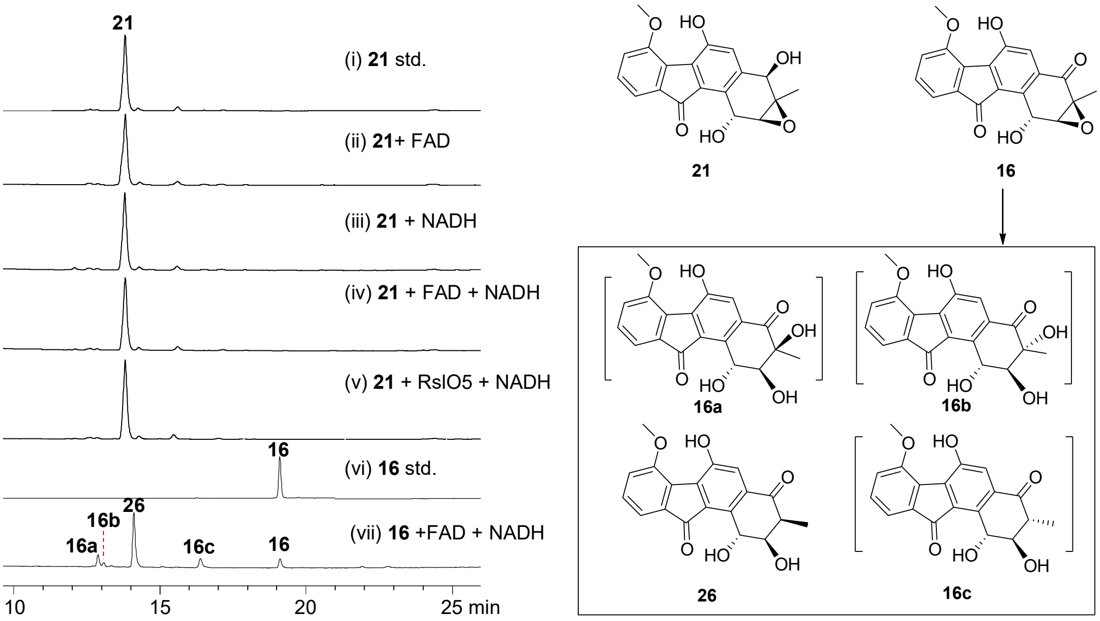

b

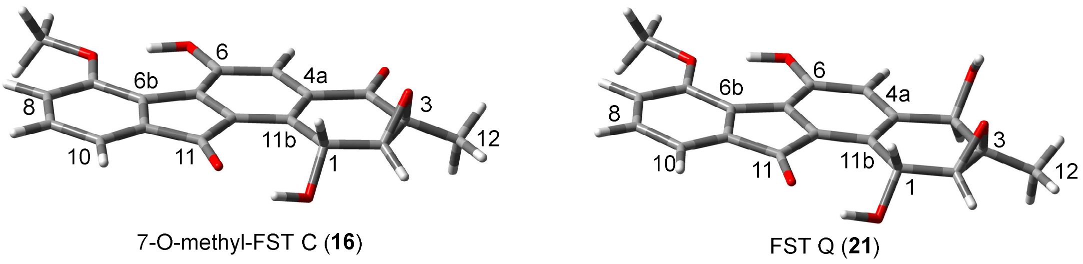

Supplementary Fig. 40. HPLC profile for FST Q (21) reaction with FAD/NADH . (**a**) HPLC analysis of reaction mixtures containing (i) **21** std.; (ii) 100 µM **21** + 100 µM FAD; (iii) 100 µM **21** + 10 mM NADH; (iv) 100 µM **21** + 100 µM FAD + 10 mM NADH; (v) 100 µM **21** + 10 µM RslO5 + 5mM NADH, (vi) **16** std.; (vii) 100 µM **16** + 100 µM FAD + 10 mM NADH. The assays were performed in 50 mM PBS buffer (pH 7) at 30 ºC for 2 h. HPLC analysis was performed on the Agilent 1260 Infinity series instrument (Agilent Technologies Inc., USA) using a reversed phase C18 column. (**b**) The 3D conformers of **16** and **21**.

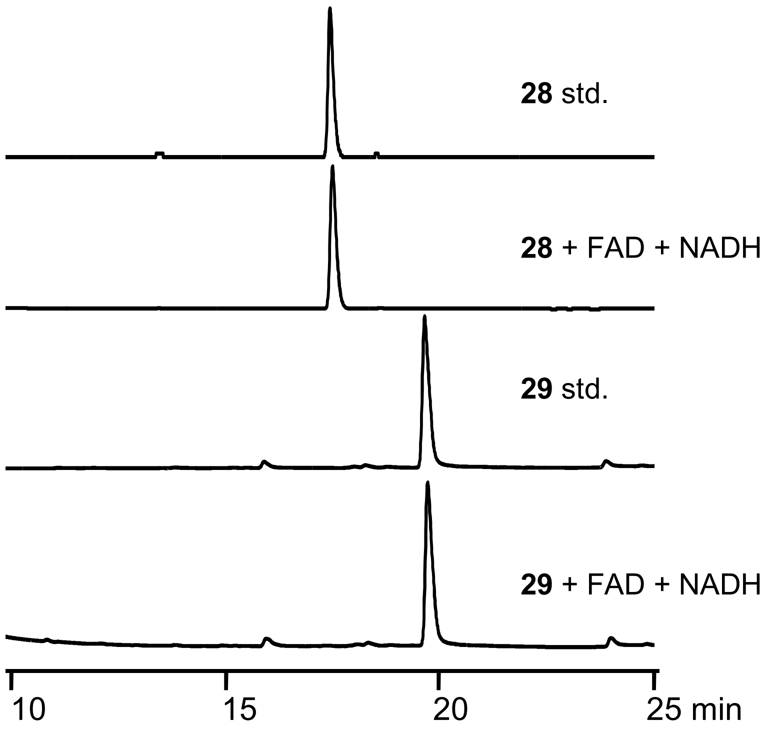

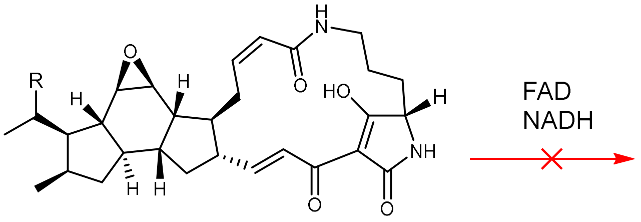

R = H (28 Epoxyikarugamycin)
R = OMe (29 Capsimycin)

Supplementary Fig. 41. HPLC analysis of FAD/NADH-mediated reactions with expoxyikarugamycin (28) and capsimycin (29). HPLC analysis of reaction mixtures containing 100 µM **28** (or **29**), 100 µM FAD and 10 mM NADH in 50 mM PBS buffer (pH 7). The reaction mixtures were incubated at 30 ºC for 2 h.

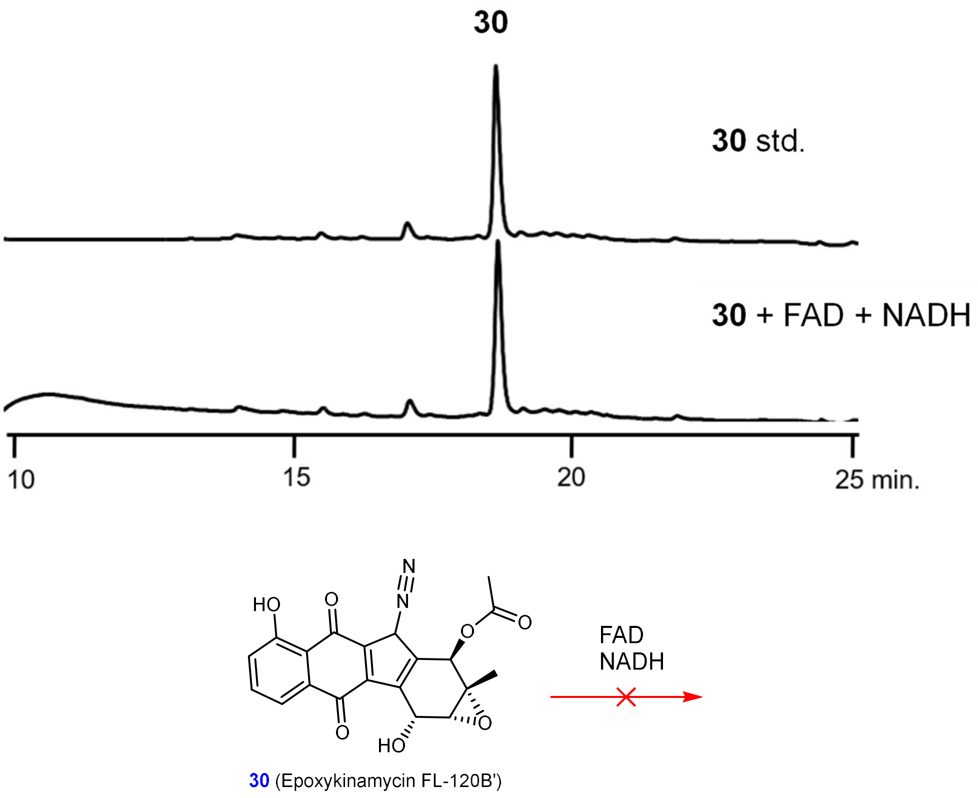

Supplementary Fig. 42. HPLC analysis of FAD/NADH-mediated reaction with epoxykinamycin FL-120B′ (30). HPLC analysis of reaction mixture containing 100 µM **30**, 100 µM FAD and 10 mM NADH in 50 mM PBS buffer (pH 7). The reaction mixtures were incubated at 30 ºC for 2 h.

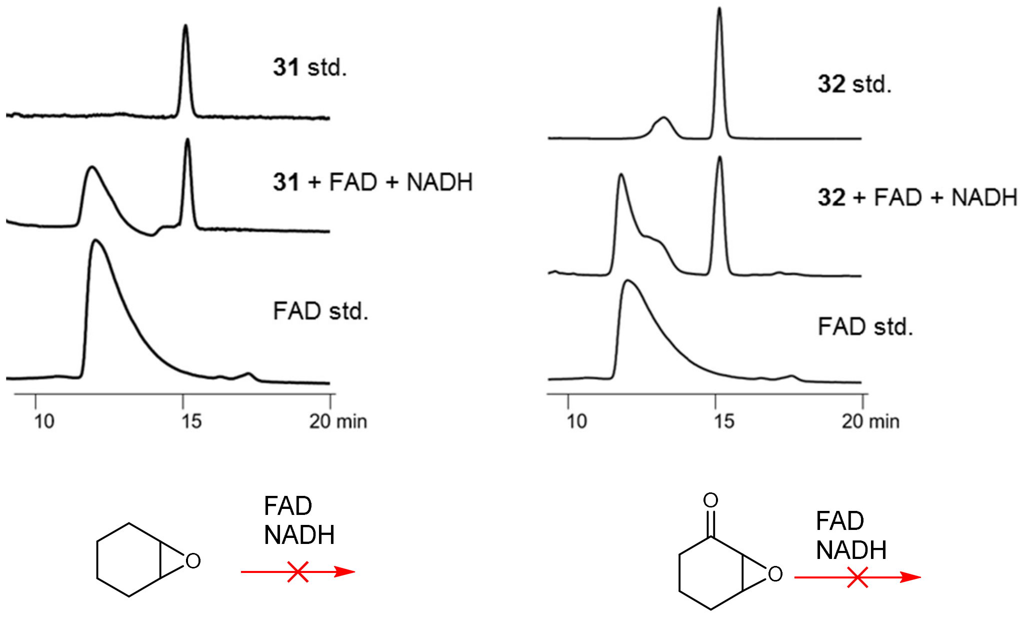

31 (1,2-epoxy-cyclohexane) 32 (2,3-epoxy-cyclohexanone)

Supplementary Fig. 43. HPLC analysis of FAD/NADH-mediated reaction with 1,2-epoxy-cyclohexane (31) and 2,3-epoxy-cyclohexanone (32). HPLC analysis of reaction mixtures containing 100 µM **31** (or **32**), 100 µM FAD and 10 mM NADH in 50 mM PBS buffer (pH 7). The reaction mixtures were incubated at 30 °C for 2 h.

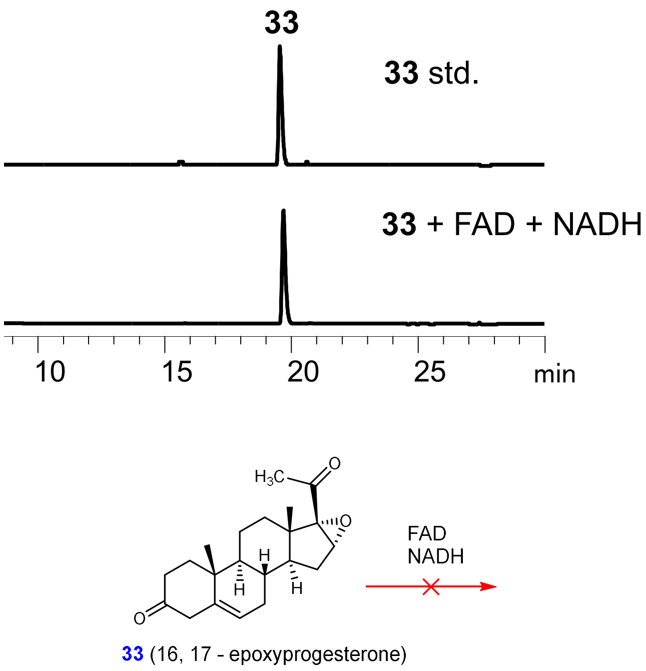

Supplementary Fig. 44. HPLC analysis of FAD/NADH-mediated reaction with 16,17-epoxyprogesterone (33). HPLC analysis of reaction mixture containing 100 μM **33**, 100 μM FAD and 10 mM NADH in 50 mM PBS buffer (pH 7). The reaction mixtures were incubated at 30 °C for 2 h.

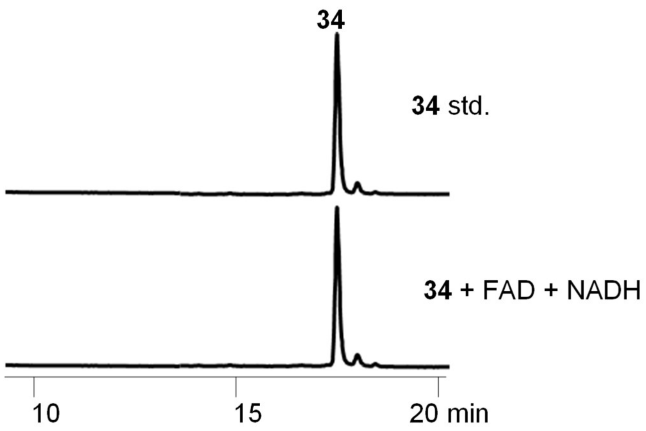

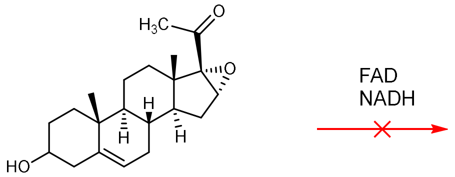

34 (16, 17 - epoxyprognenolone)

Supplementary Fig. 45. HPLC analysis of FAD/NADH-mediated reaction with 16,17-epoxypregnenolone (34). HPLC analysis of reaction mixture containing 100 µM **34**, 100 µM FAD and 10 mM NADH in 50 mM PBS buffer (pH 7). The reaction mixtures were incubated at 30 °C for 2 h.

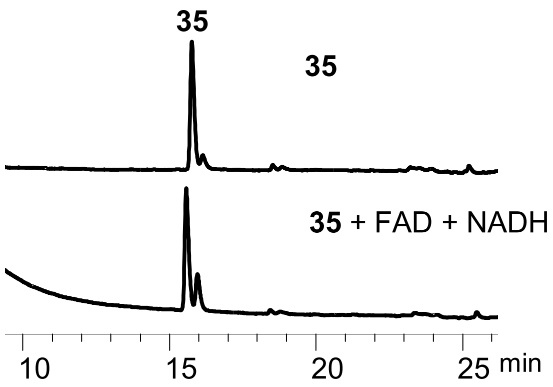

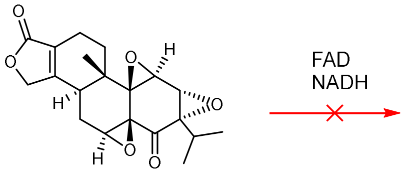

35 (Triptonide)

Supplementary Fig. 46. HPLC analysis of FAD/NADH-mediated reaction with triptonide (35). HPLC analysis of the reaction mixture containing 100 μM **35**, 100 μM FAD and 10 mM NADH in 50 mM PBS buffer (pH 7). The reaction mixtures were incubated at 30 °C for 2 h.

a

b

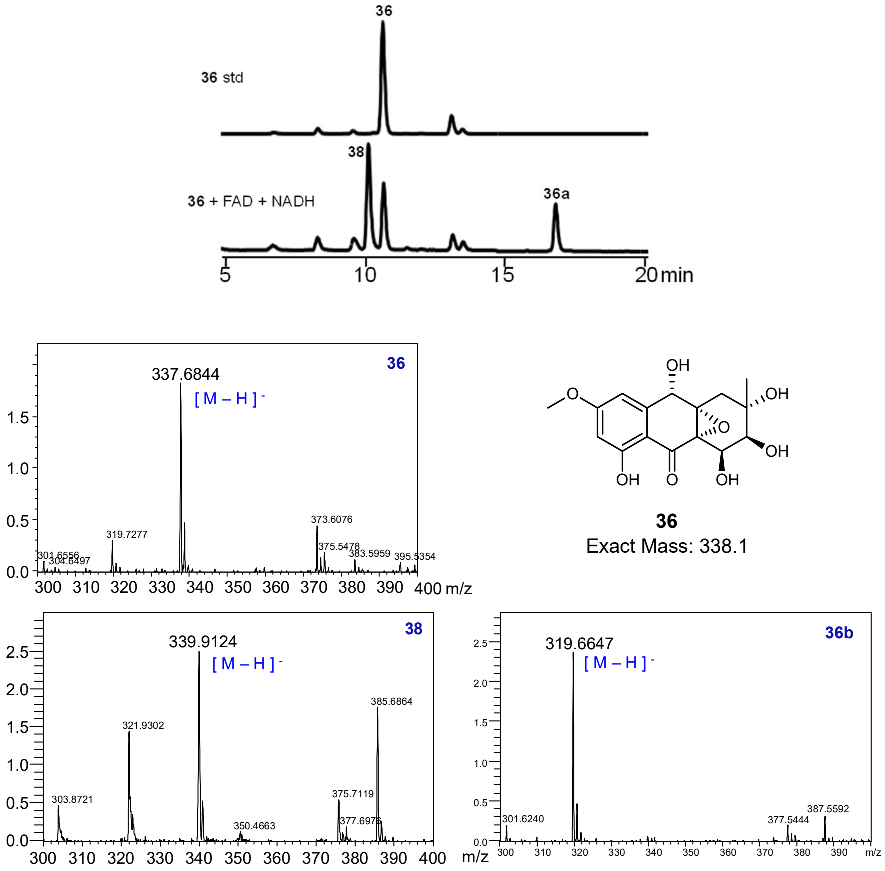

Supplementary Fig. 47. HPLC and LC-MS analysis of the reaction of auxarthrol H (36) with FAD/NADH. (**a**) HPLC analysis of the reaction mixture containing 100 μM **36**, 100 μM FAD and 10 mM NADH in 50 mM PBS buffer (pH 7). The reaction mixtures were incubated at 30 °C for 30 min. (**b**) LC-MS analysis of **36** reaction products.

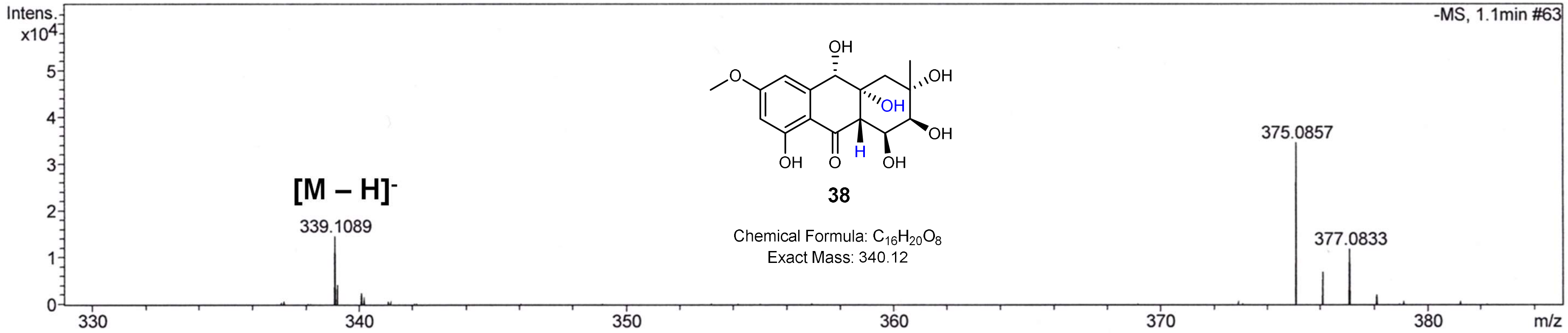

Meas. m/z	#	Ion Formula	Score	m/z	err [ppm]	err [mDa]	mSigma	rdb	e⁻ Conf	N-Rule
339.1089	1	C16H19O8	100.00	339.1085	1.0	0.3	8.6	7.5	even	ok
375.0857	1	C16H20ClO8	100.00	375.0852	1.2	0.5	11.1	6.5	even	ok
679.2251	1	C32H39O16	100.00	679.2244	1.0	0.7	13.7	13.5	even	ok
715.2014	1	C32H40ClO16	100.00	715.2010	0.5	0.4	13.1	12.5	even	ok

Supplementary Fig. 48. Spectroscopic data for 38. (**a**) HRESIMS spectrum.

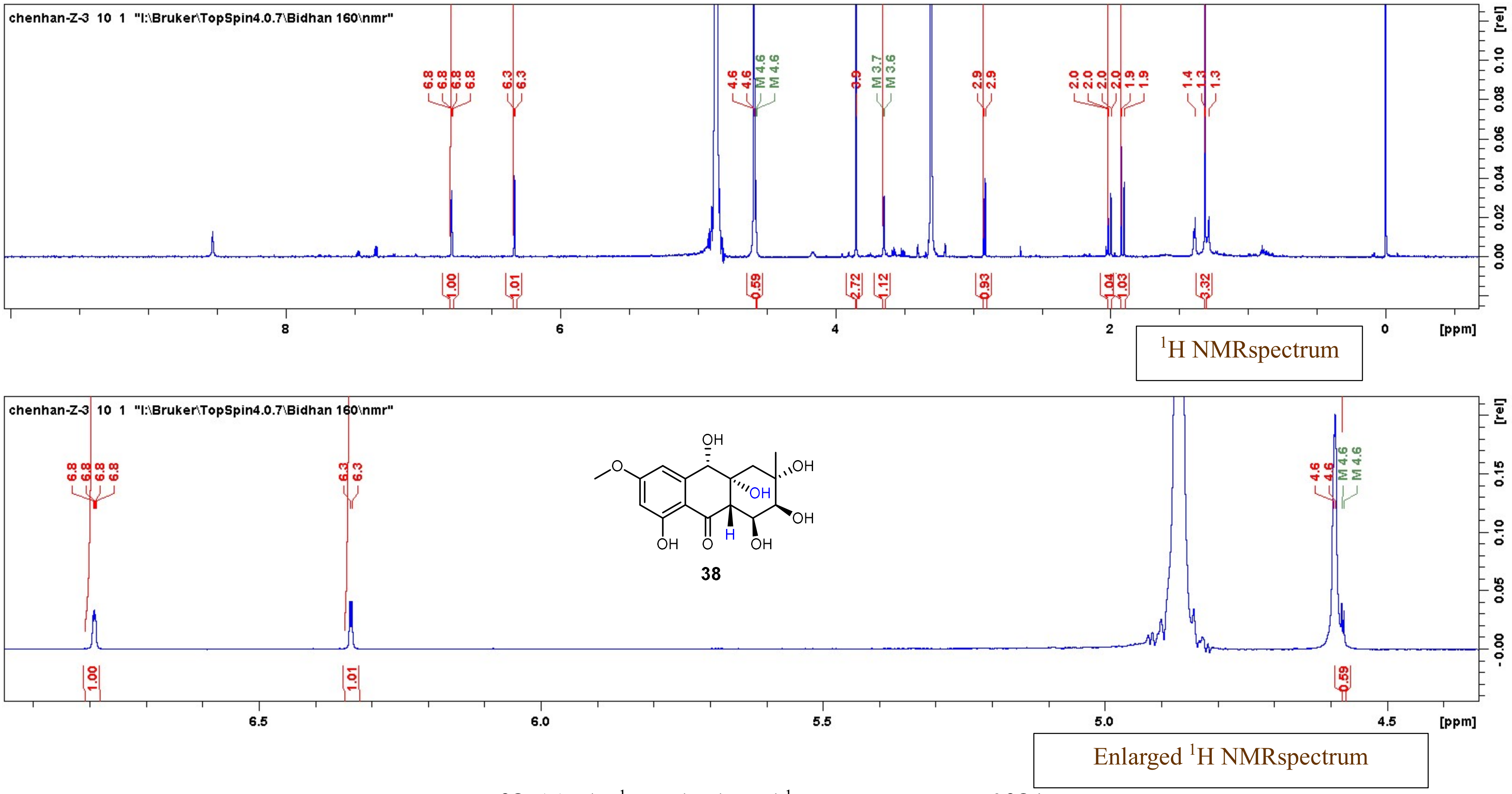

Supplementary Fig. 48. Spectroscopic data for 38. (**b**) The ^{1}H and enlarged ^{1}H NMR spectrum of **38** in DMSO-d_6.

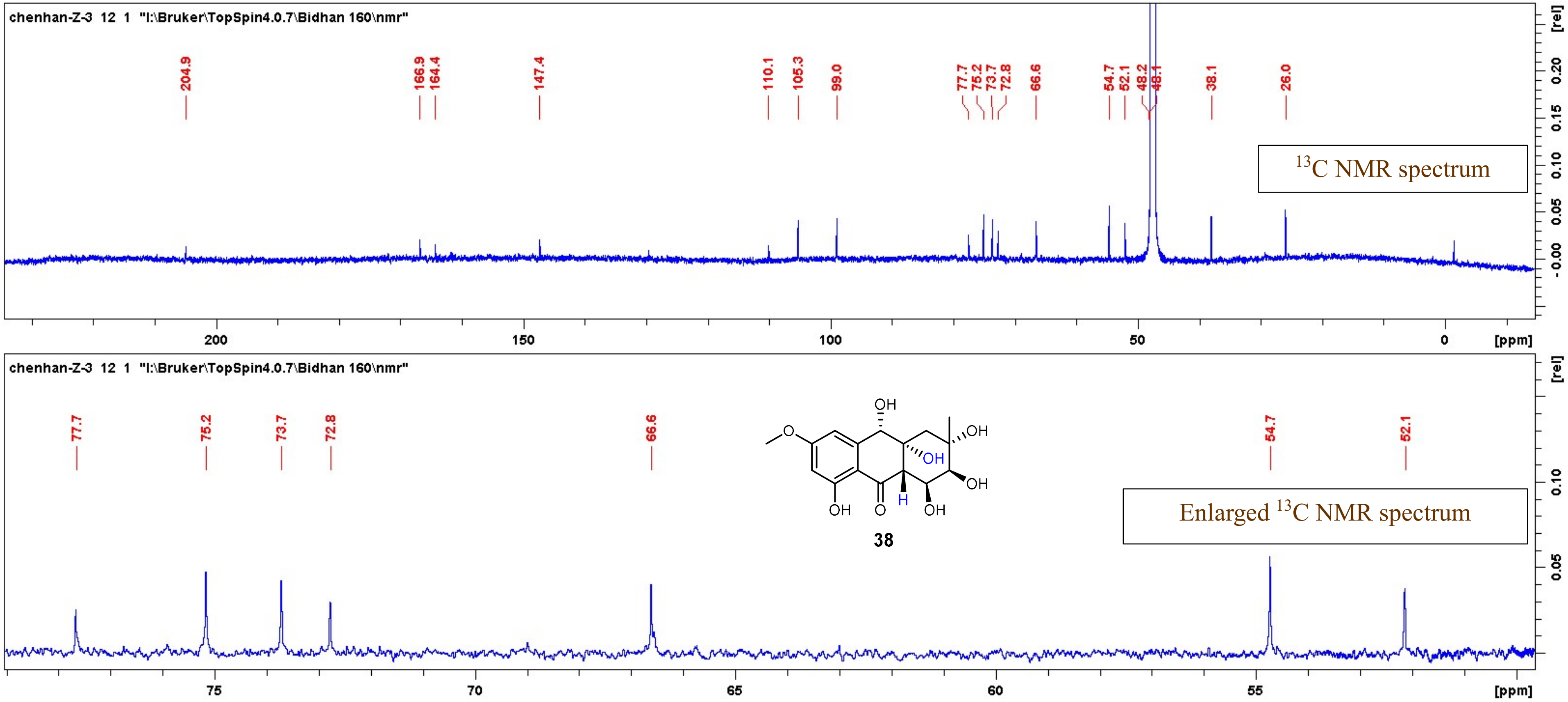

Supplementary Fig. 48. Spectroscopic data for 38. (**c**) The ^{13}C and enlarged ^{13}C NMR spectrum of **38** in DMSO-d_6.

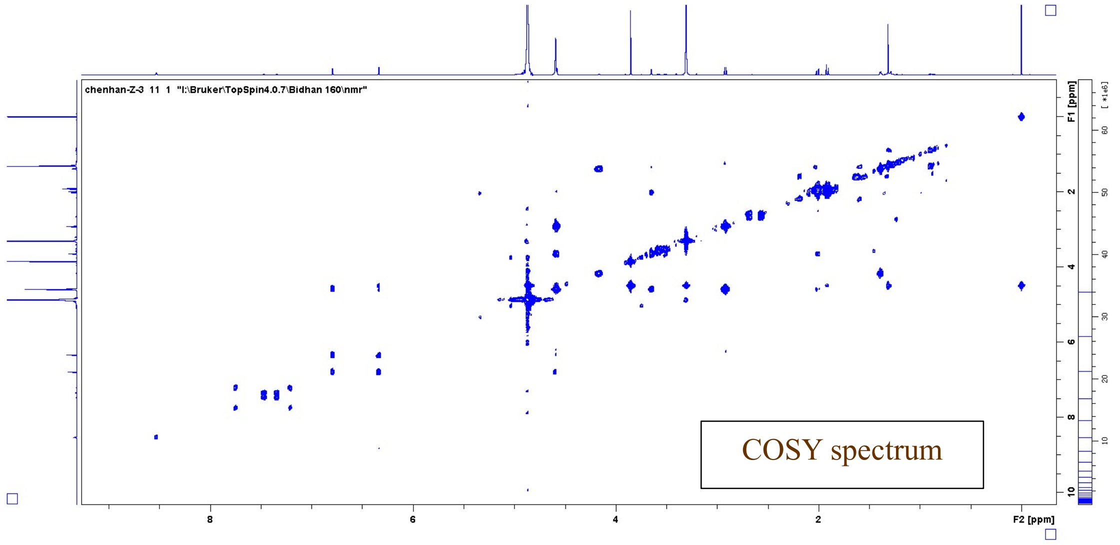

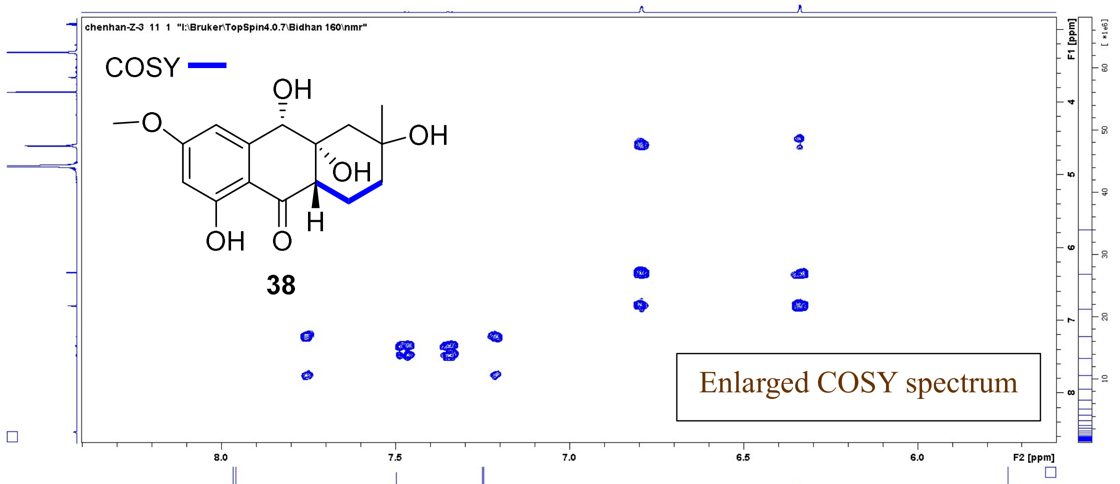

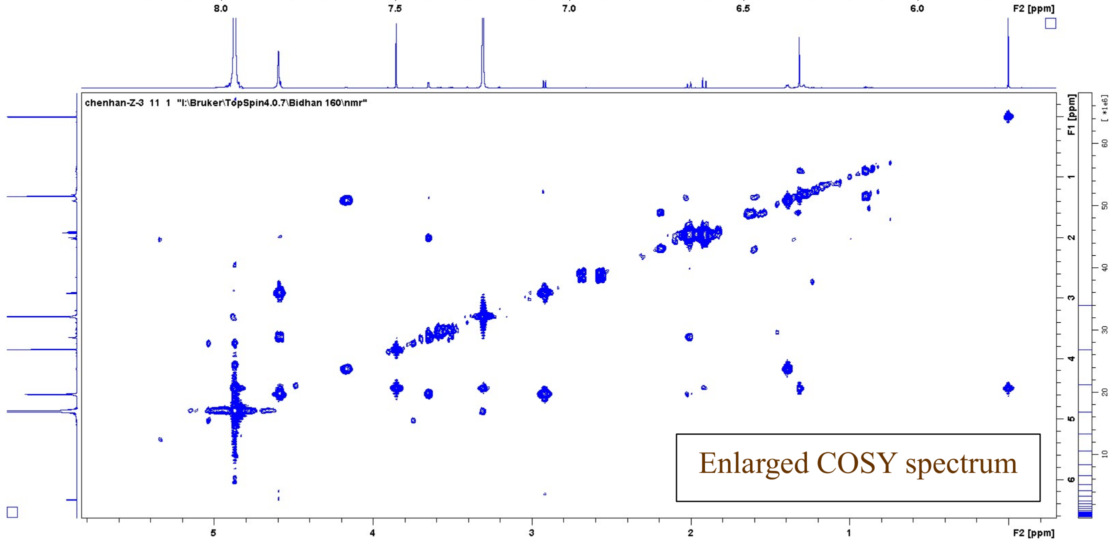

Supplementary Fig. 48. Spectroscopic data for 38. (**d**) The COSY and enlarged COSY spectrum of **38** in DMSO-d_6.

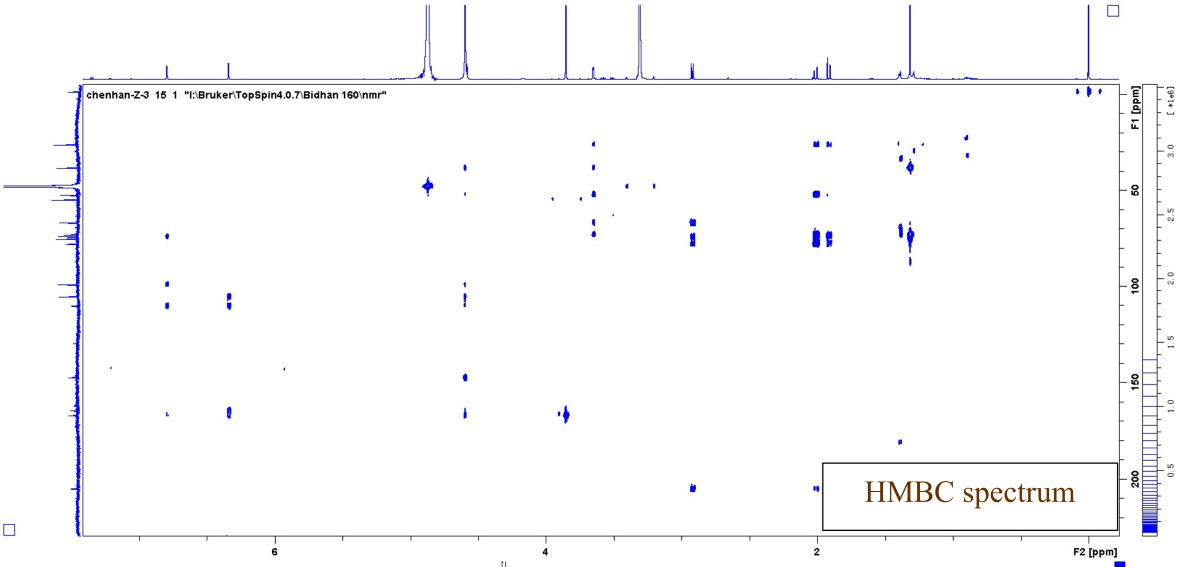

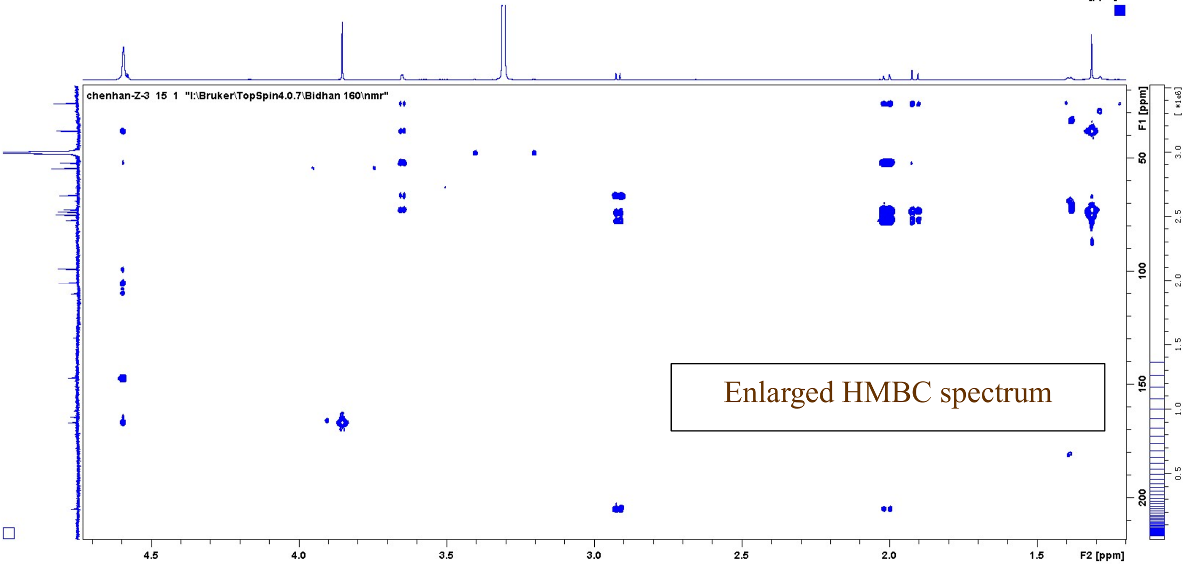

Supplementary Fig. 48. Spectroscopic data for 38. (**e**) The HMBC and enlarged HMBC spectrum of **38** in DMSO-d_6.

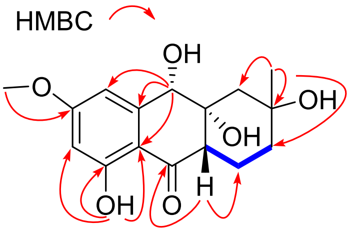

38

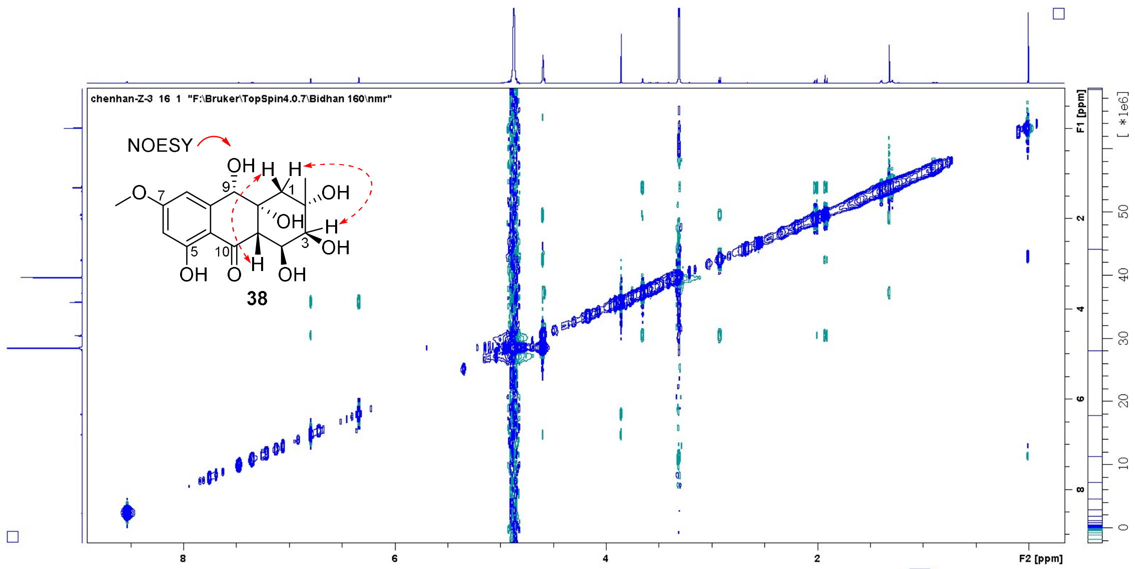

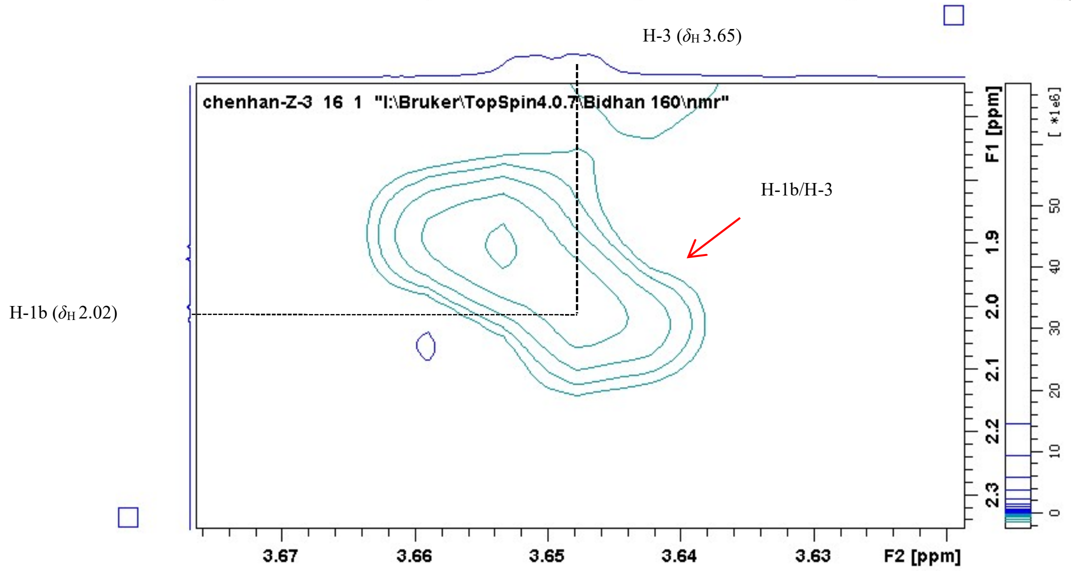

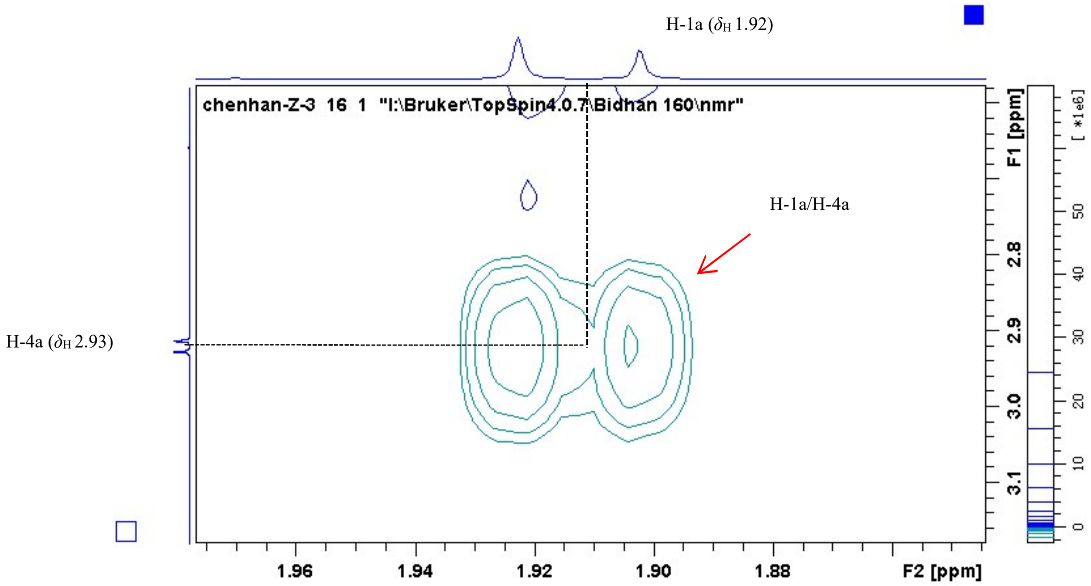

Supplementary Fig. 48. Spectroscopic data for 38. (f) The NOESY and enlarged NOESY spectrum of **38** in DMSO-d_6.

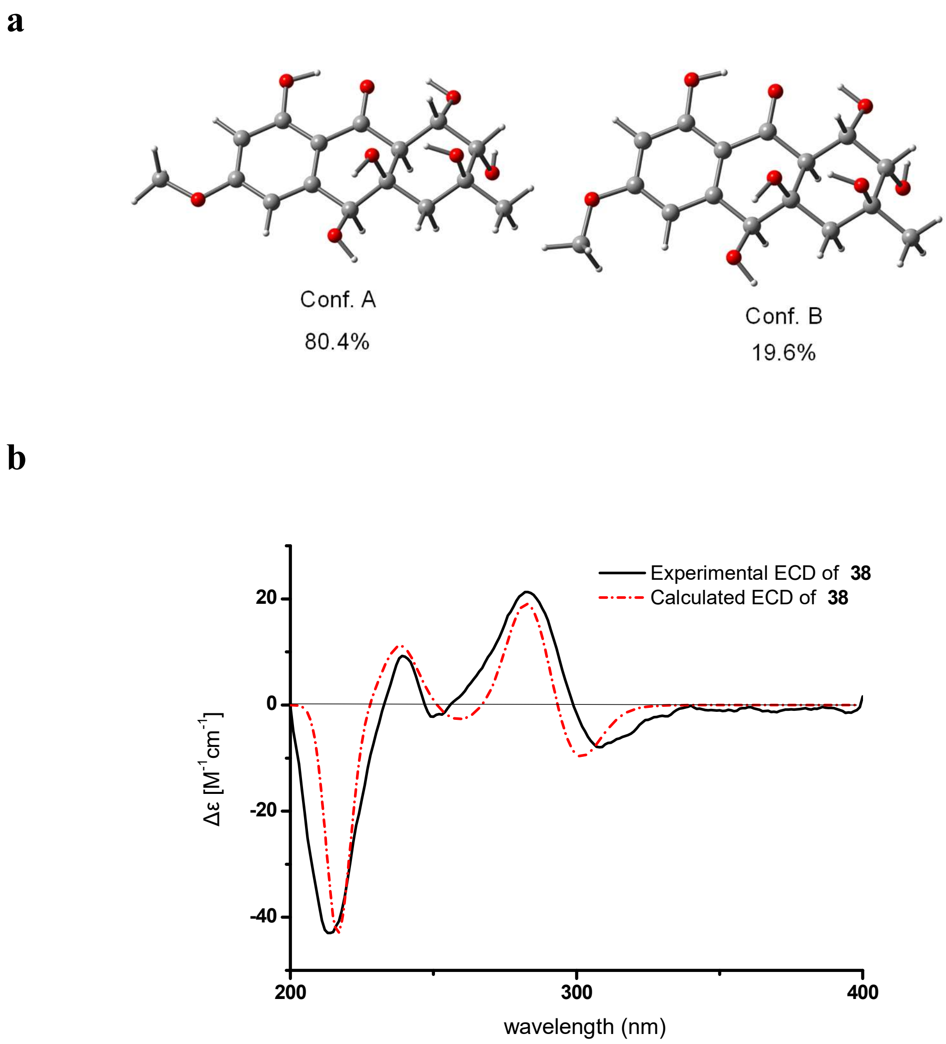

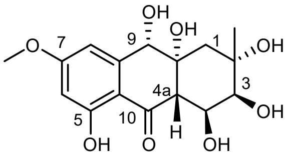

38

Supplementary Fig. 49. Comparison of experimental and calculated ECD spectra of 38 in MeOH. (**a**) Structure and population of the low-energy ωB97X/TZVP PCM/MeCN conformers (> 1%) of (2*S*,3*S*,4*S*,4a*S*,9*S*,9a*R*)-**38.** (**b**) Experimental ECD spectrum of **38** in MeOH compared with the Boltzmann-weighted B3LYP/TZVP PCM/MeCN ECD spectrum of 2*S*,3*S*,4*S*,4a*S*,9*S*,9a*R* computed for the ωB97X/TZVP PCM/MeOH conformers.

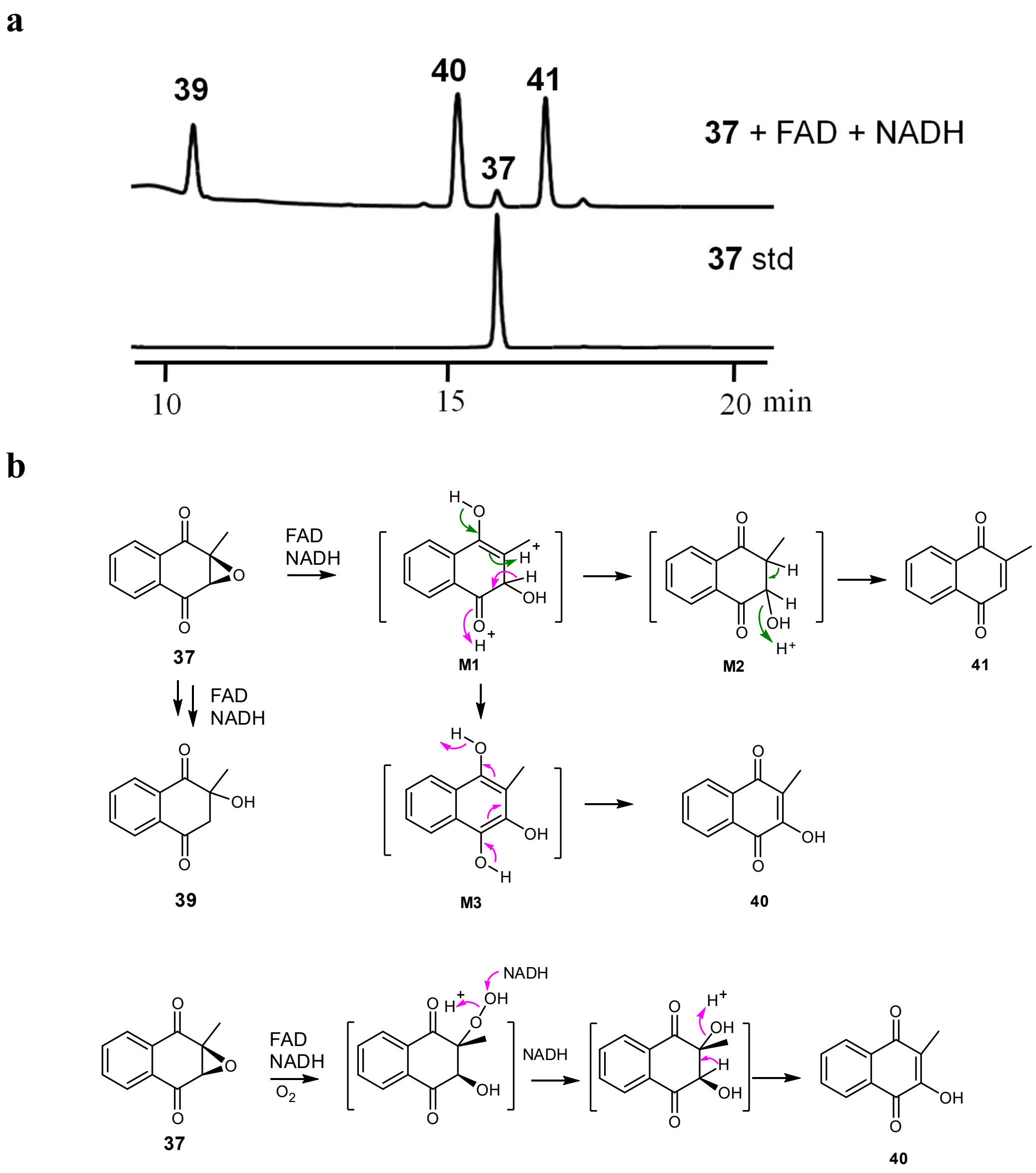

Supplementary Fig. 50. HPLC analysis of the reaction of menadione 2,3-epoxide (37) with FAD/NADH. (**a**) HPLC analysis of the reaction mixture containing 100 µM **37**, 100 µM FAD and 10 mM NADH in 50 mM PBS buffer (pH 7). The reaction mixtures were incubated at 30 ºC for 30 min. (**b**) The proposed mechanism for the flavin-mediated epoxide ring opening reactions of **37**.

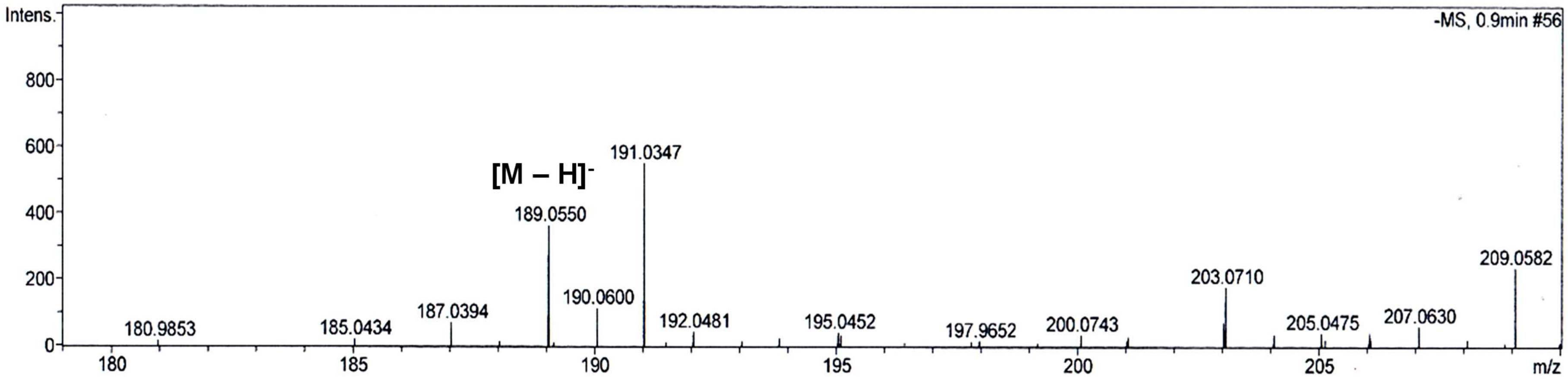

Meas. m/z	#	Ion Formula	Score	m/z	err [ppm]	err [mDa]	mSigma	rdb	e⁻ Conf	N-Rule
189.0550	1	C11H9O3	100.00	189.0557	3.7	0.7	115.3	7.5	even	ok

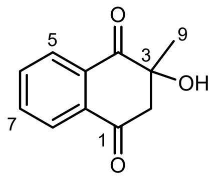

39

Chemical Formula: $C_{11}H_{10}O_3$
Exact Mass: 190.06

Supplementary Fig. 51. Spectroscopic data for 39. (**a**) HRESIMS spectrum.

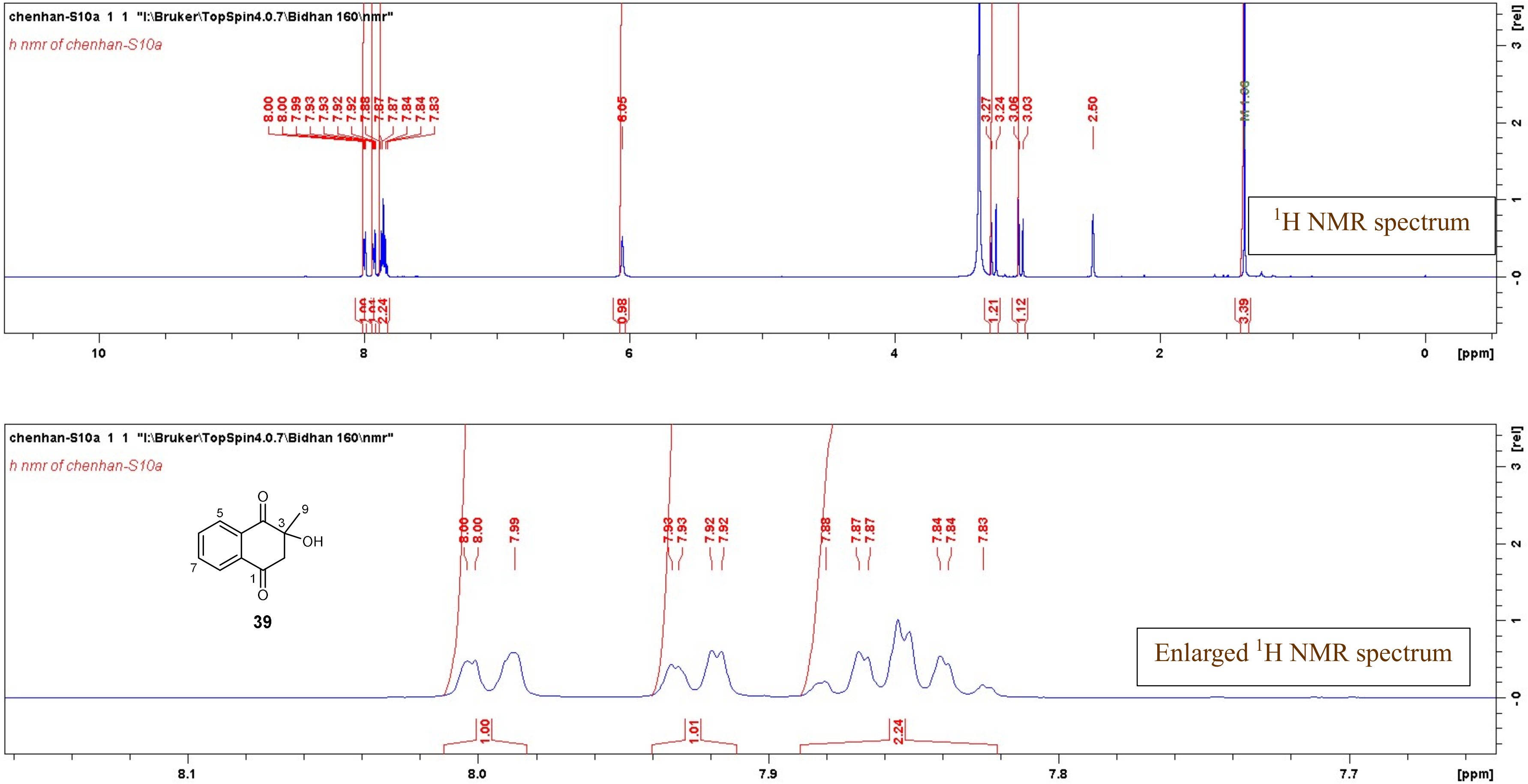

Supplementary Fig. 51. Spectroscopic data for 39. (**b**) The ^{1}H and enlarged ^{1}H NMR spectrum of **39** in DMSO-d_6.

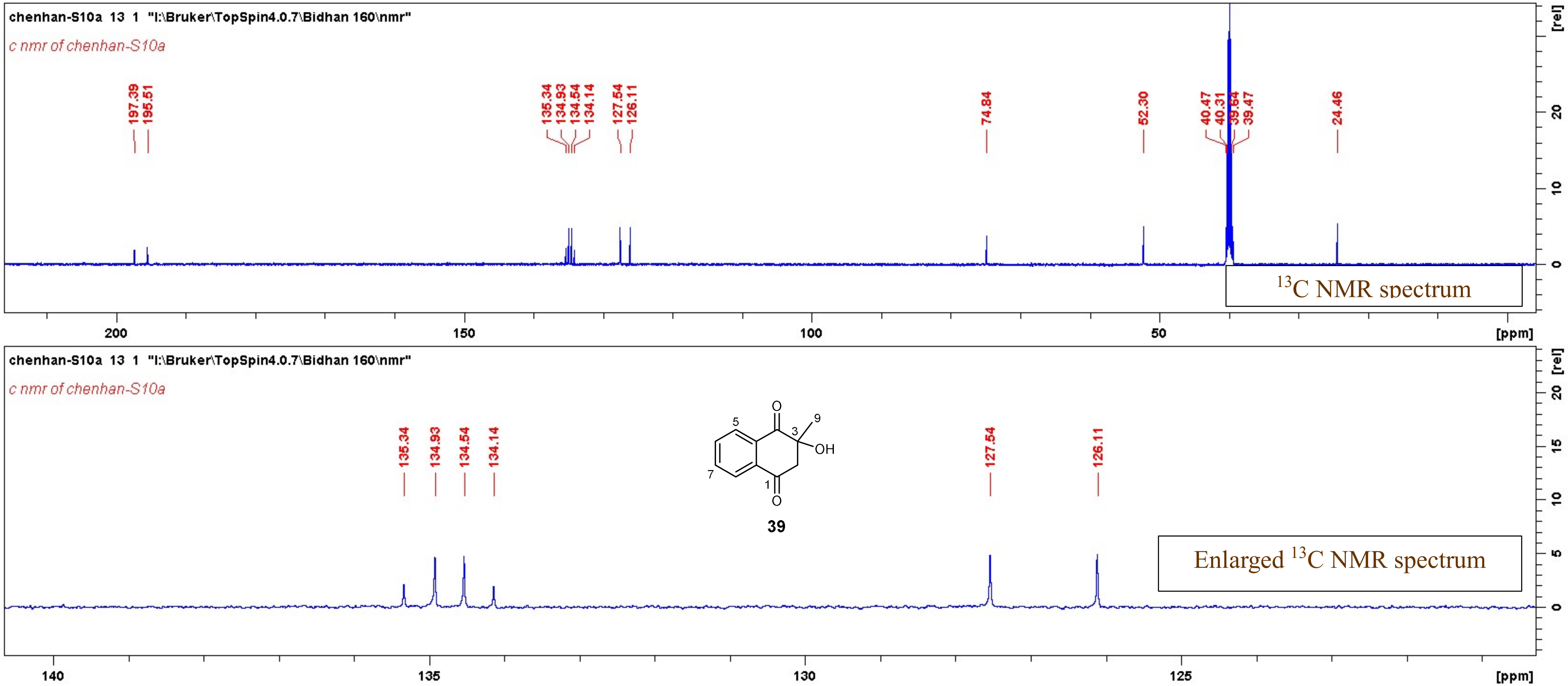

Supplementary Fig. 51. Spectroscopic data for 39. (**c**) The ^{13}C and enlarged ^{13}C NMR spectrum of **39** in DMSO-d_6.

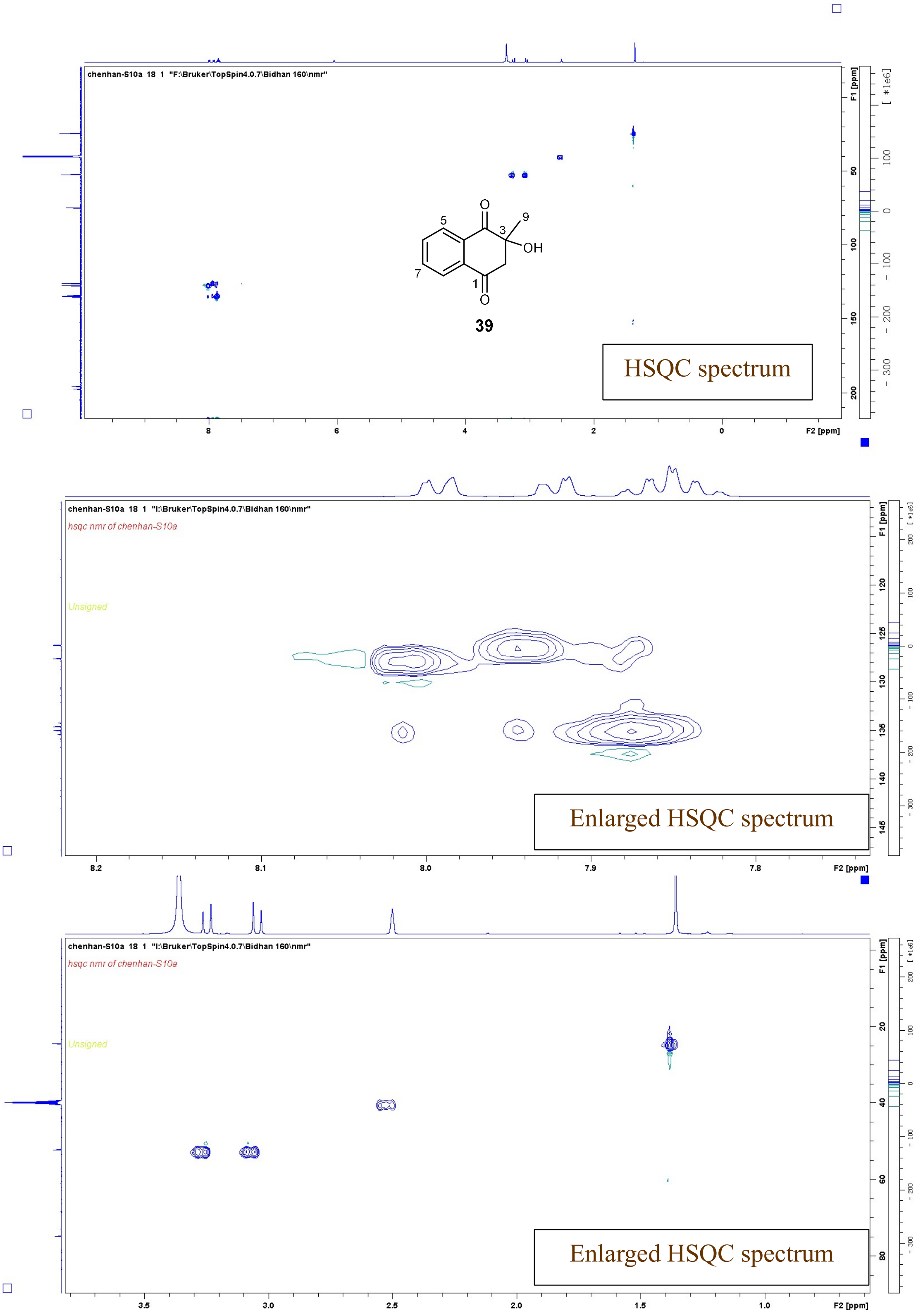

Supplementary Fig. 51. Spectroscopic data for 39. (**d**) The HSQC and enlarged HSQC spectrum of **39** in DMSO-d_6.

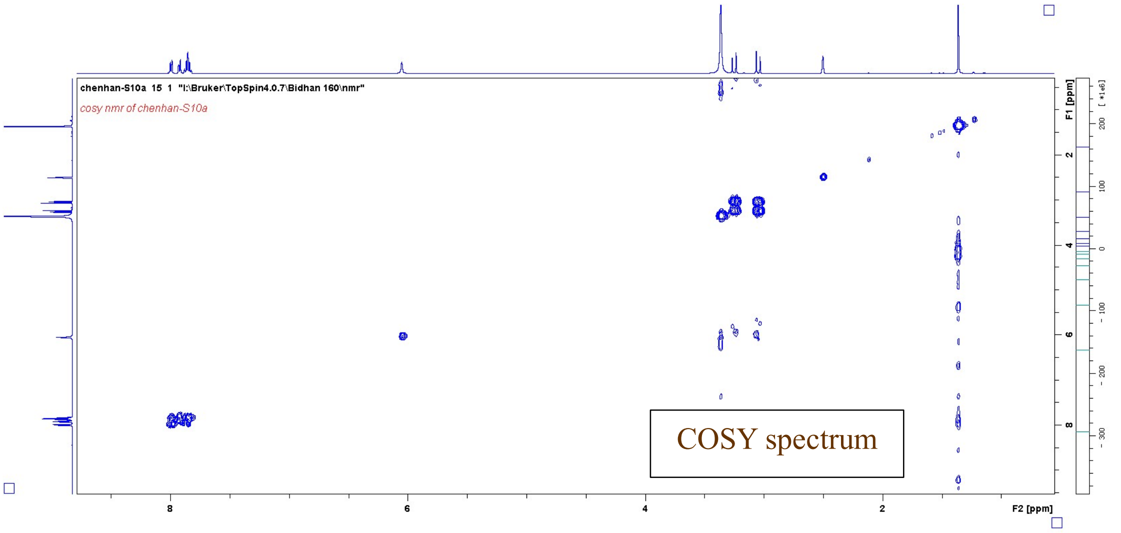

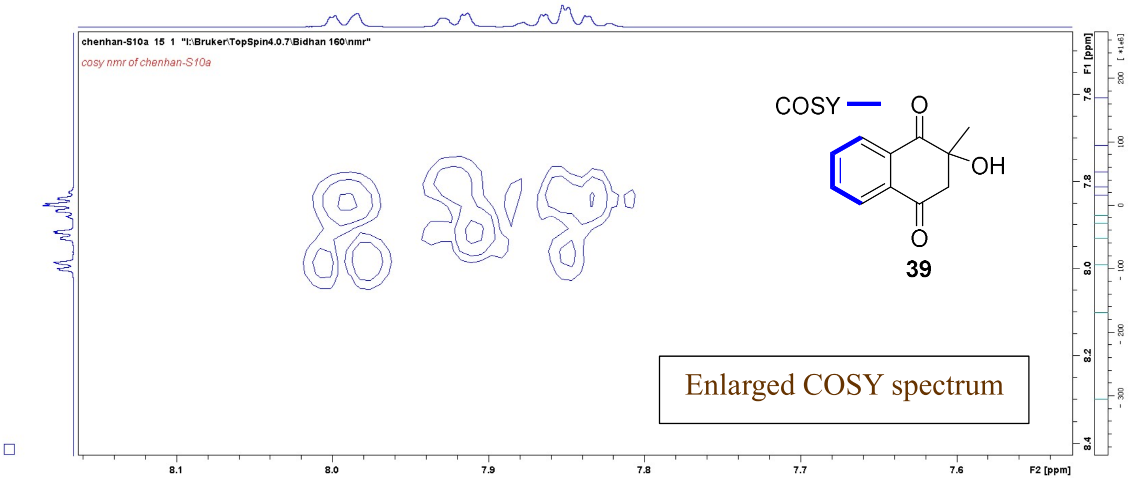

Supplementary Fig. 51. Spectroscopic data for 39. (**e**) The COSY and enlarged COSY spectrum of **39** in DMSO-d_6.

\

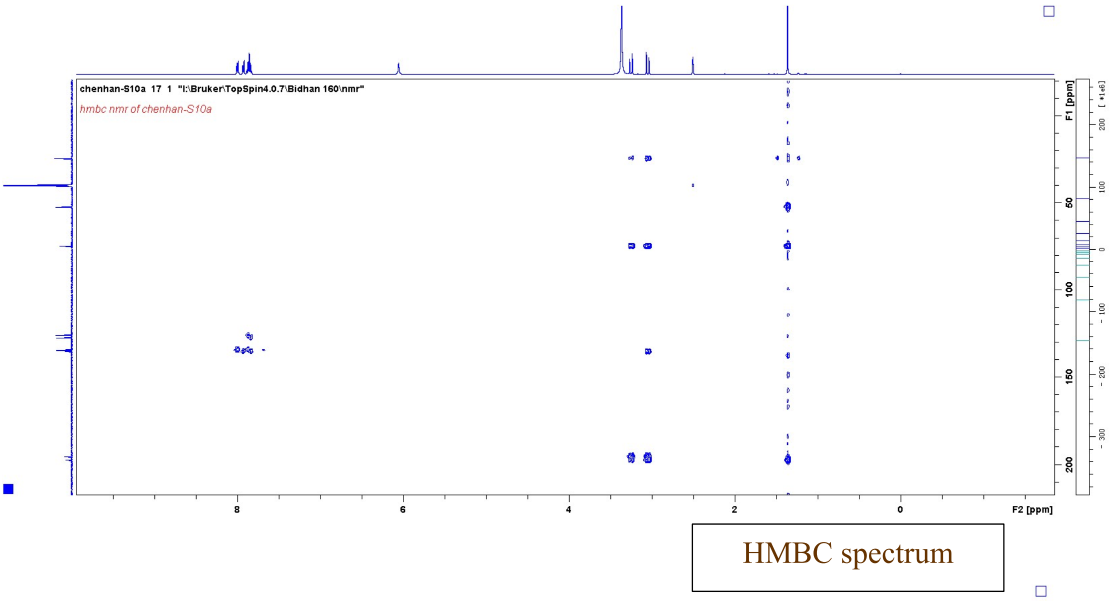

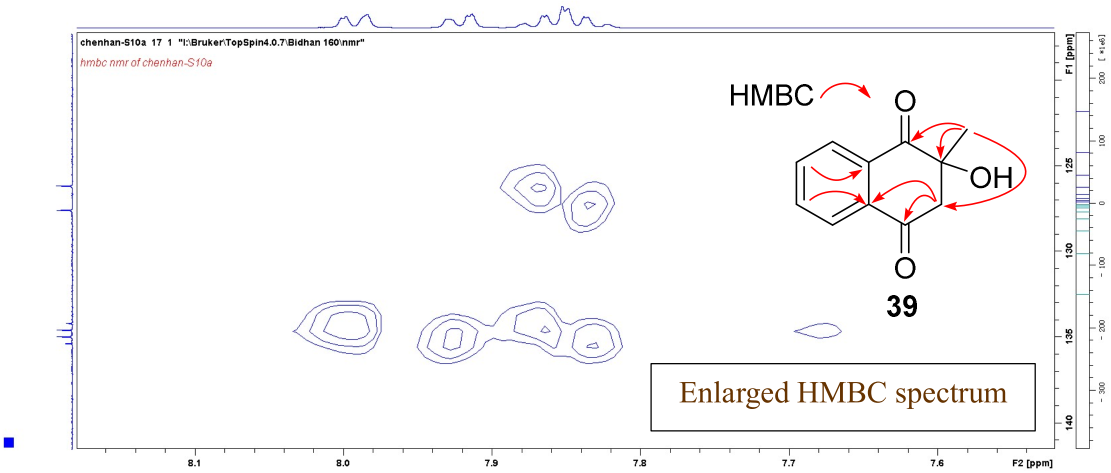

Supplementary Fig. 51. Spectroscopic data for 39. (**f**) The HMBC and enlarged HMBC spectrum of **39** in DMSO-d_6.

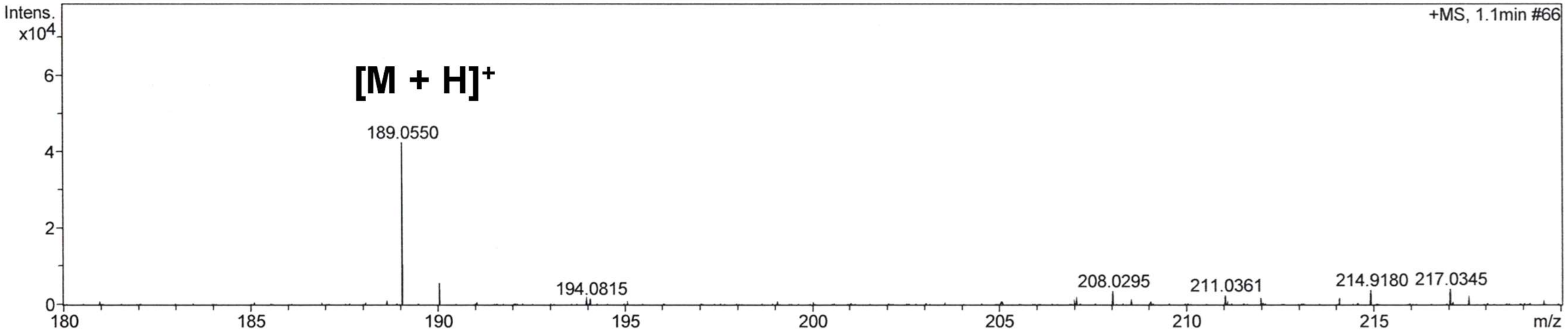

Meas. m/z	#	Ion Formula	Score	m/z	err [ppm]	err [mDa]	mSigma	rdb	e⁻ Conf	N-Rule
189.0550	1	C11H9O3	100.00	189.0546	1.9	0.4	7.0	7.5	even	ok
211.0361	1	C11H8NaO3	100.00	211.0366	2.4	0.5	32.6	7.5	even	ok

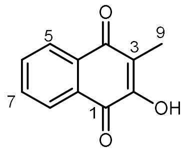

40

Chemical Formula: $C_{11}H_8O_3$
Exact Mass: 188.05

Supplementary Fig. 52 Spectroscopic data for 40. (**a**) HRESIMS spectrum

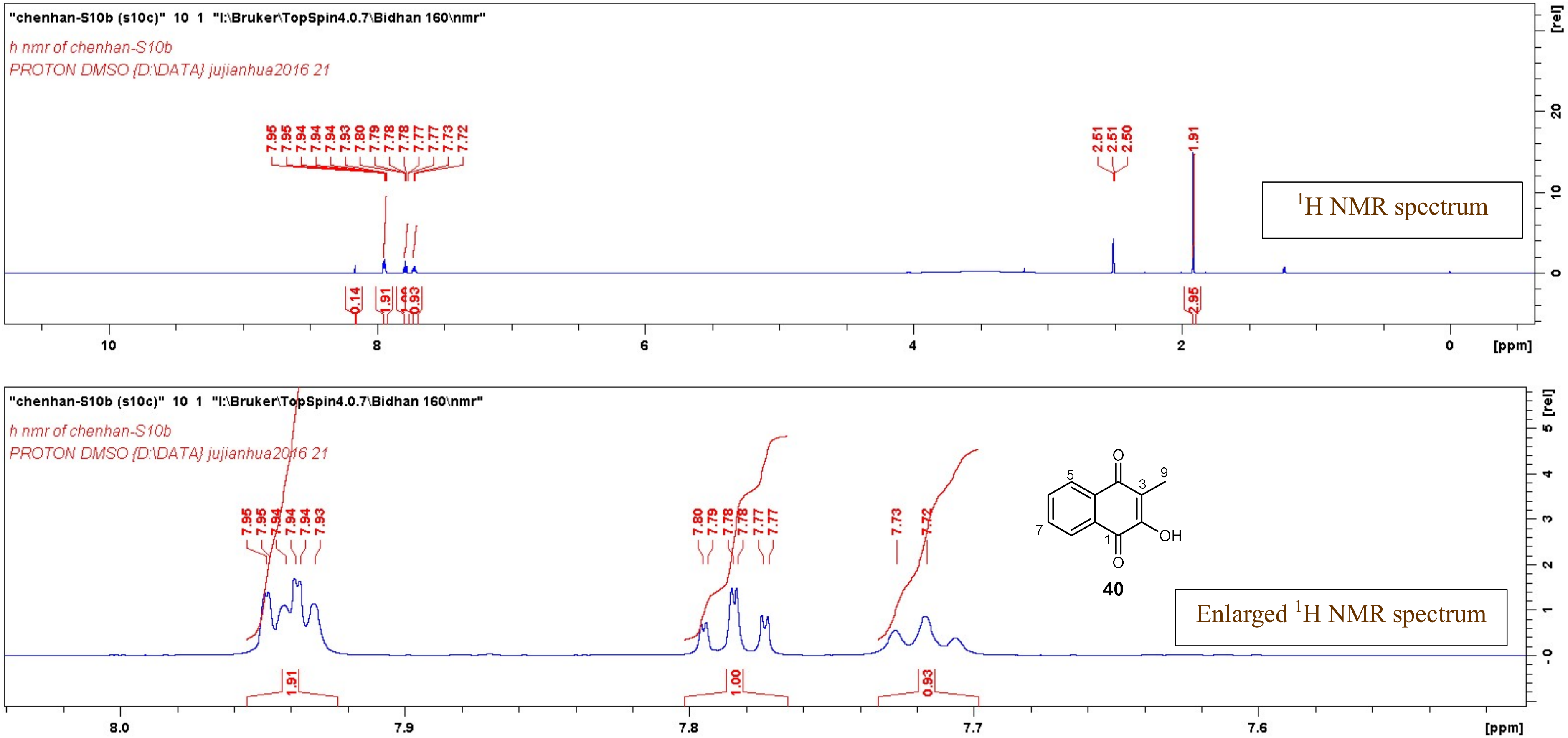

Supplementary Fig. 52 Spectroscopic data for 40. (**b**) The ^{1}H and enlarged ^{1}H NMR spectrum of in **40** in DMSO-d_6.

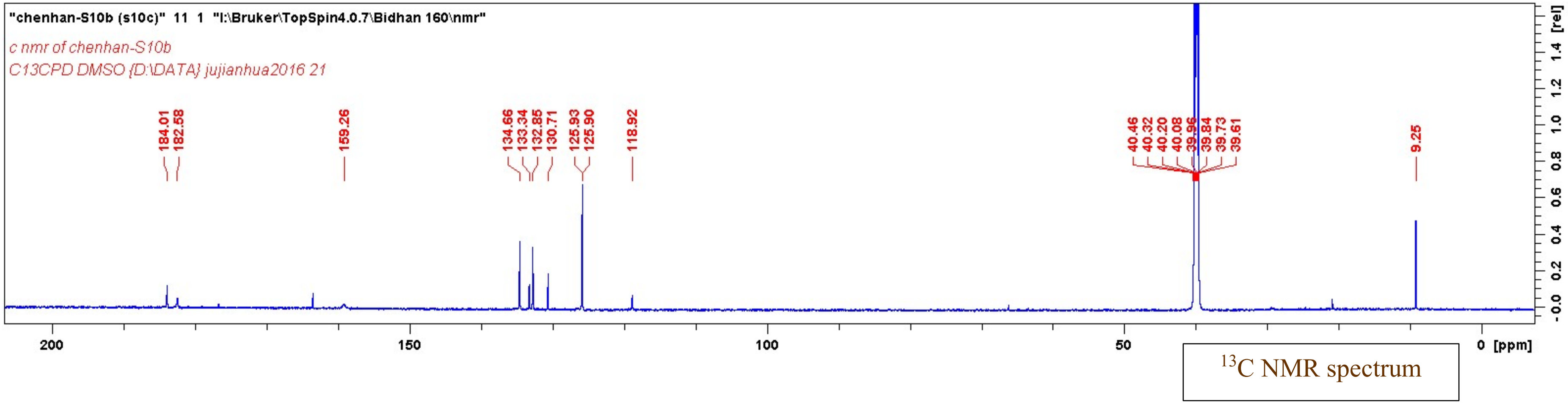

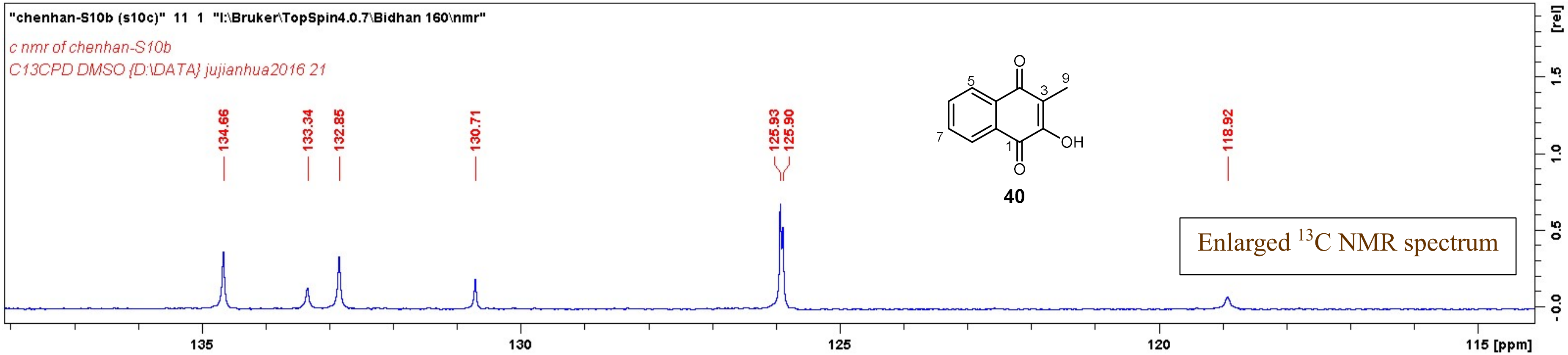

Supplementary Fig. 52 Spectroscopic data for 40. (**c**) The ^{13}C and enlarged ^{13}C NMR spectrum of **40** in DMSO-d_6.

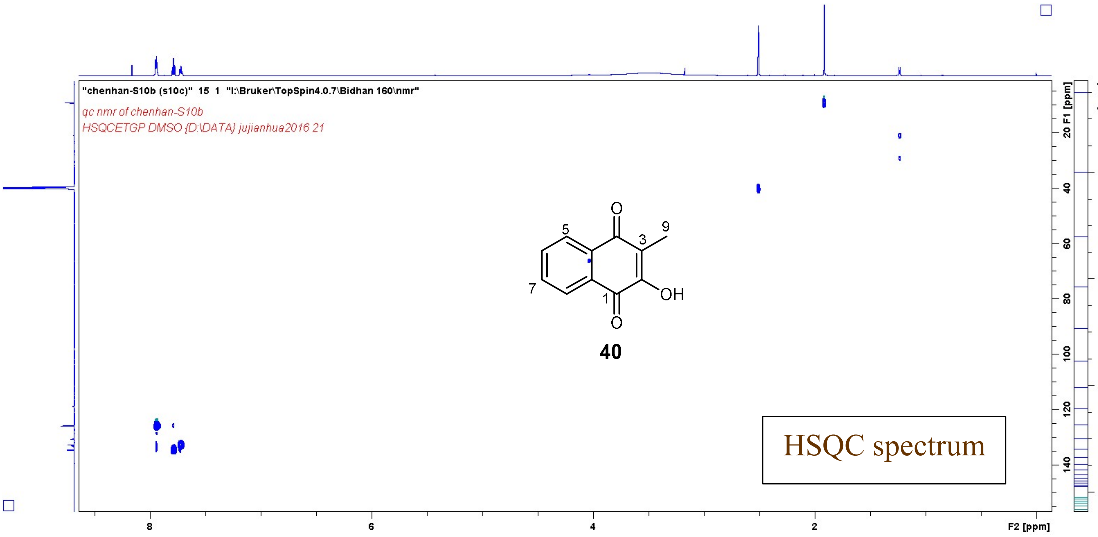

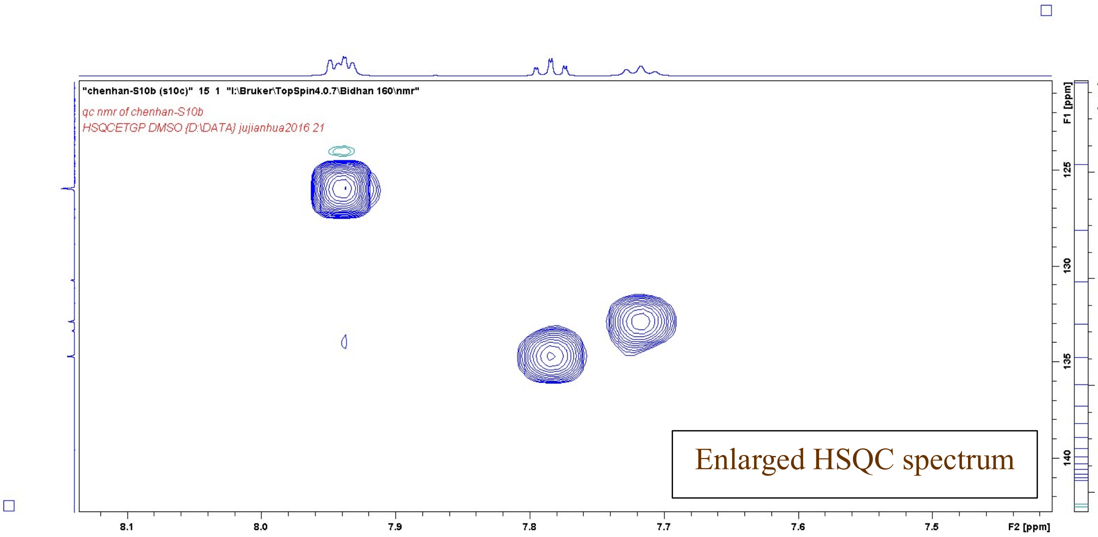

Supplementary Fig. 52 Spectroscopic data for 40. (d) The HSQC and enlarged HSQC spectrum of **40** in DMSO-d_6.

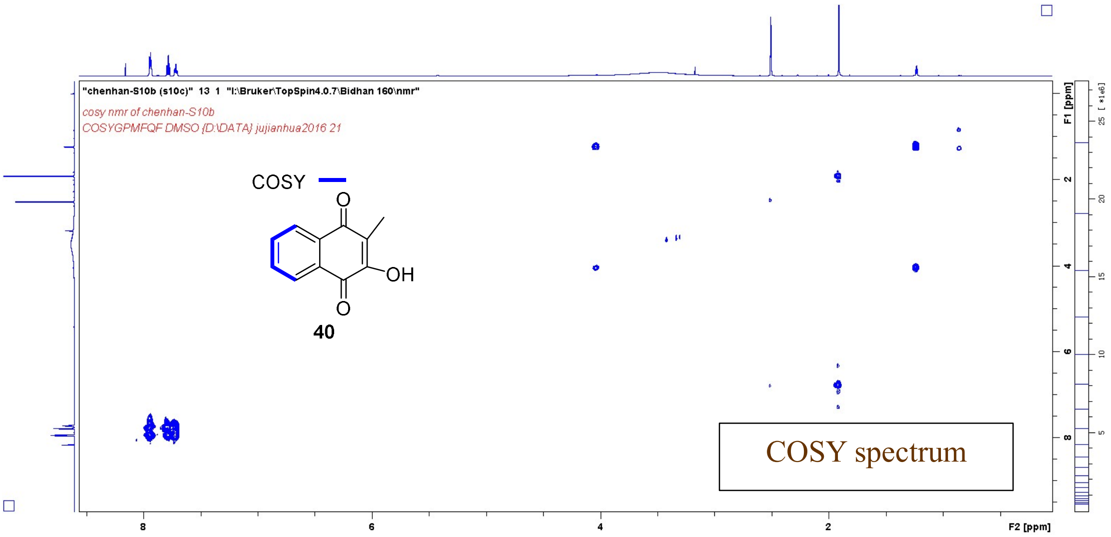

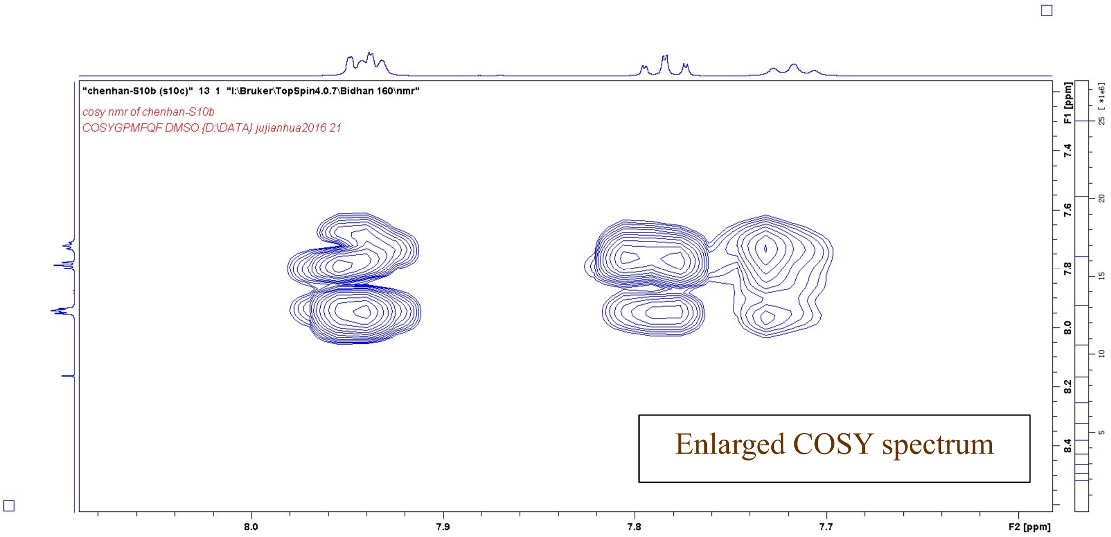

Supplementary Fig. 52 Spectroscopic data for 40. (**e**) The COSY and enlarged COSY spectrum of **40** in DMSO-d_6.

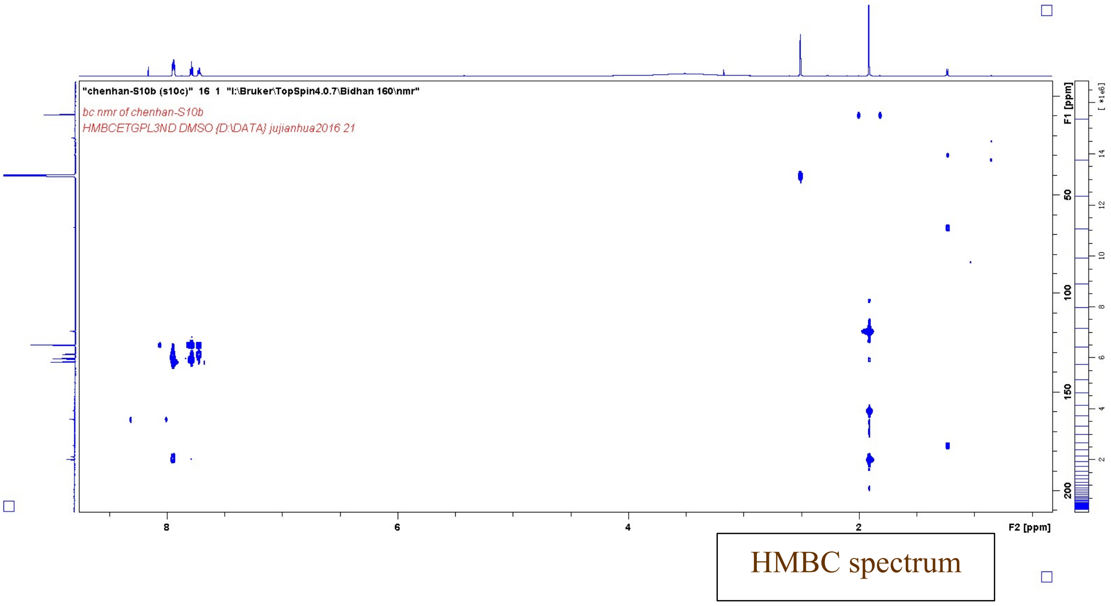

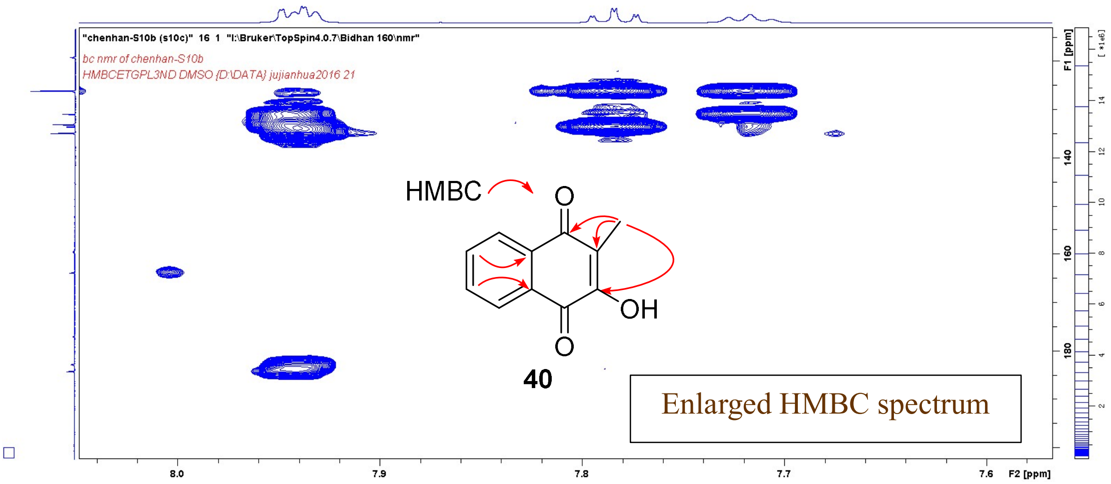

Supplementary Fig. 52 Spectroscopic data for 40. (**f**) The HMBC and enlarged HMBC spectrum of **40** in DMSO-d_6.

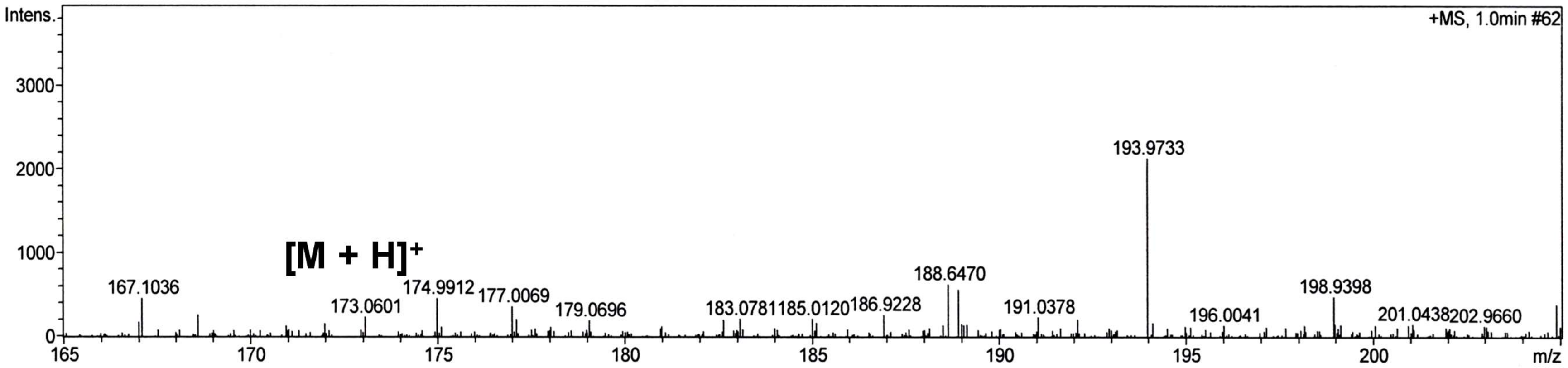

Meas. m/z	#	Ion Formula	Score	m/z	err [ppm]	err [mDa]	mSigma	rdb	e⁻ Conf	N-Rule
173.0601	1	C11H9O2	100.00	173.0597	-2.2	-0.4	34.3	7.5	even	ok

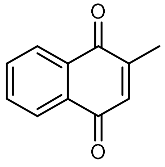

41

Chemical Formula: $C_{11}H_8O_2$

Exact Mass: 172.05

Supplementary Fig. 53 Spectroscopic data for 41. (**a**) HRESIMS spectrum

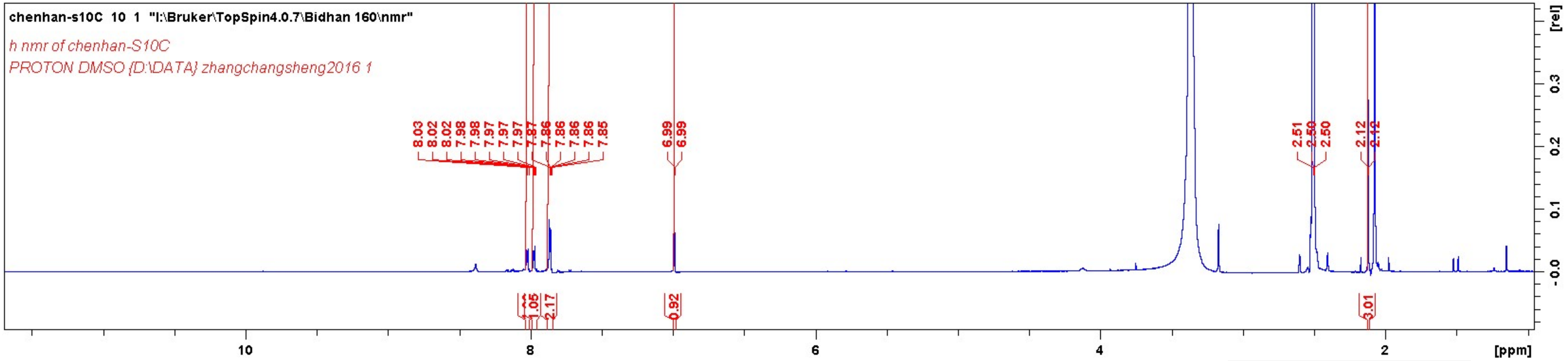

¹H NMR spectrum

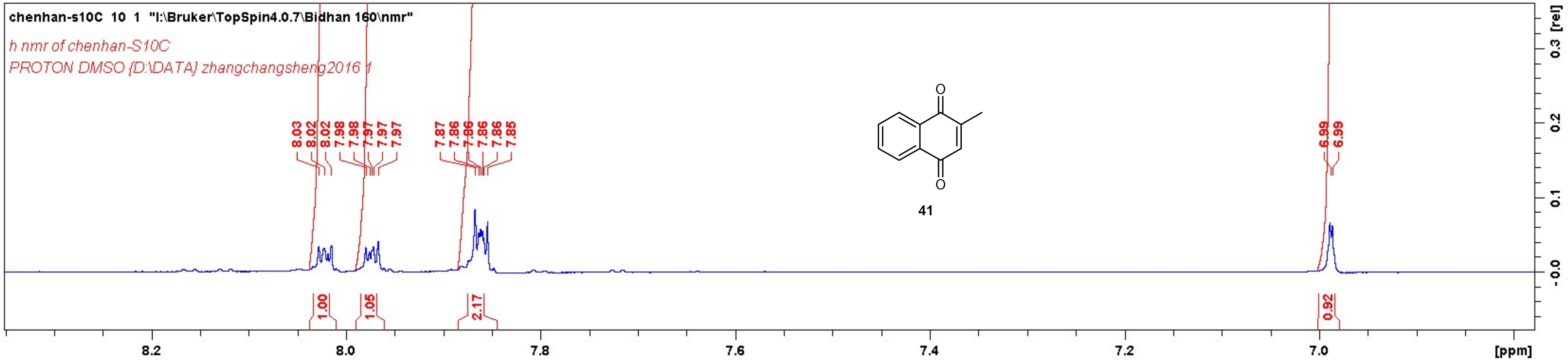

Enlarged ¹H NMR spectrum

Supplementary Fig. 53 Spectroscopic data for 41. (**b**) The ¹H and enlarged ¹H NMR spectrum of **41** in DMSO-d_6.

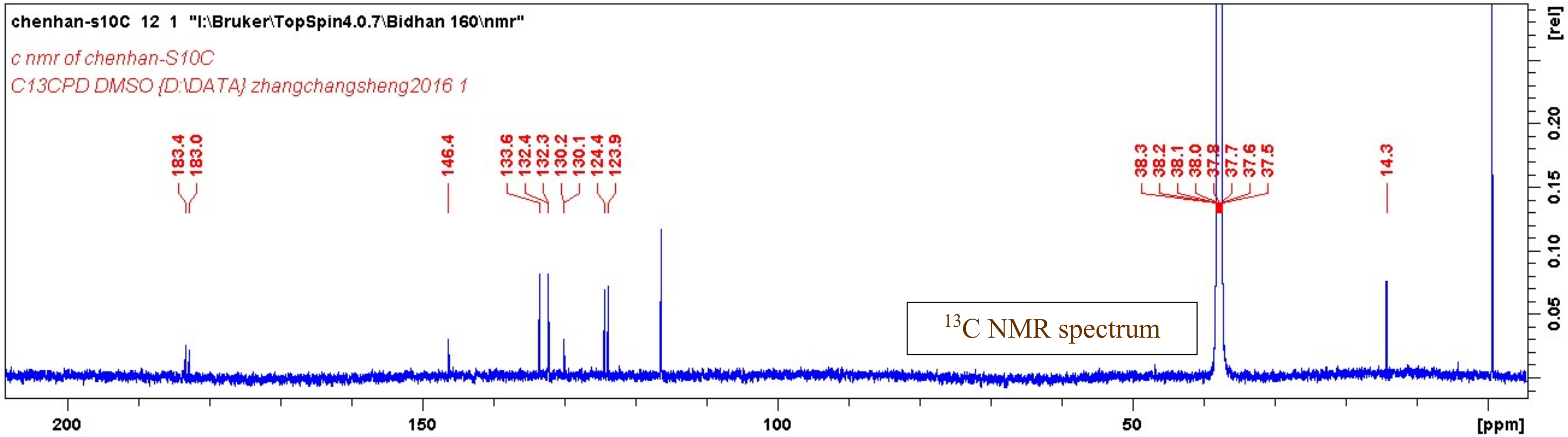

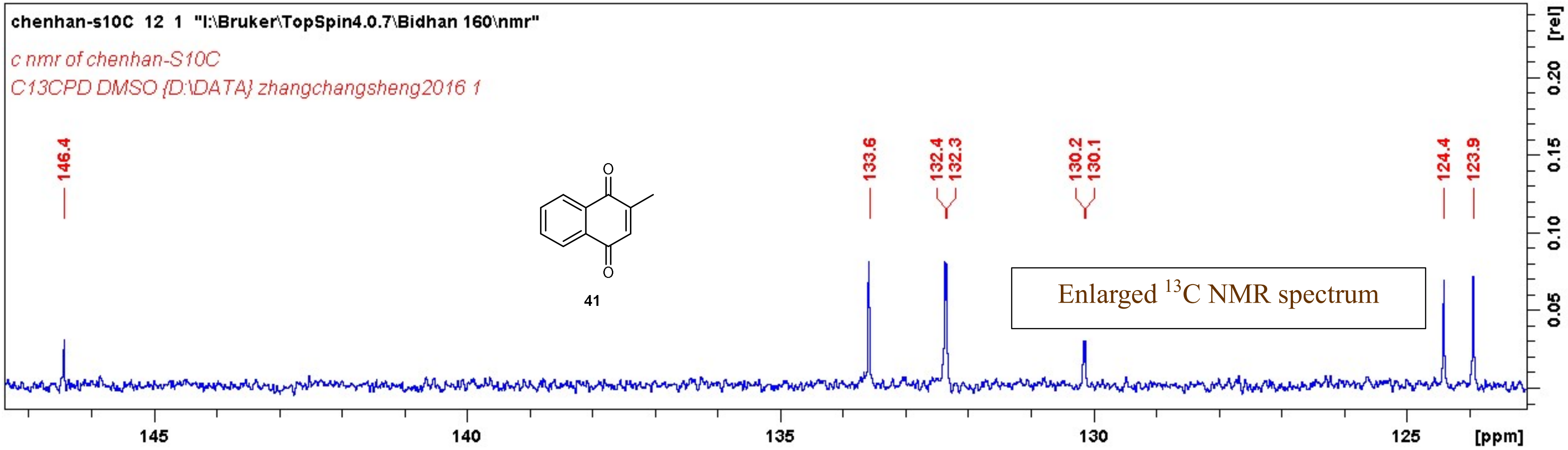

Supplementary Fig. 53 Spectroscopic data for 41. (**c**) The ^{13}C and enlarged ^{13}C NMR spectrum of **41** in DMSO-d_6.

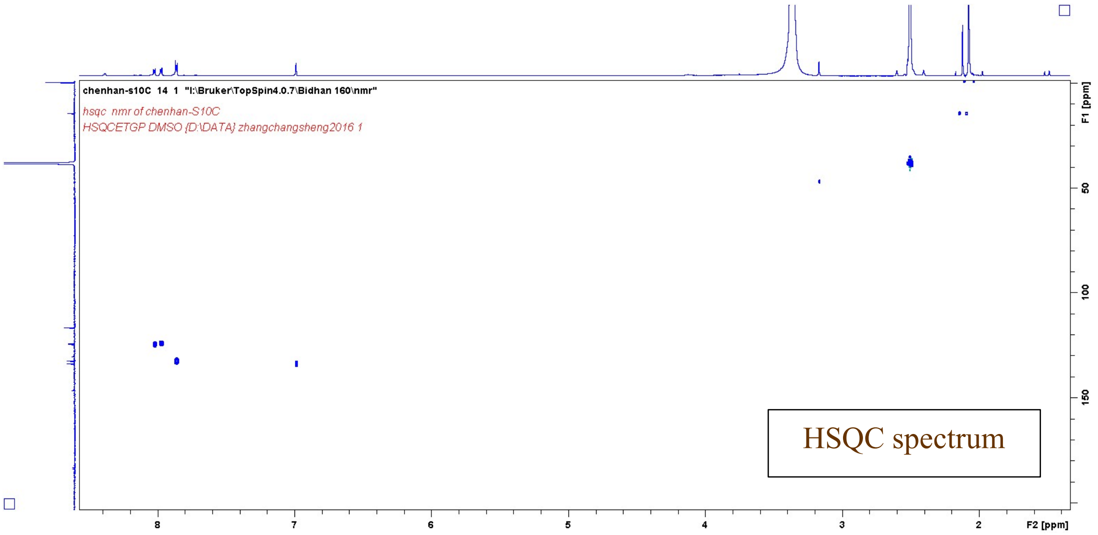

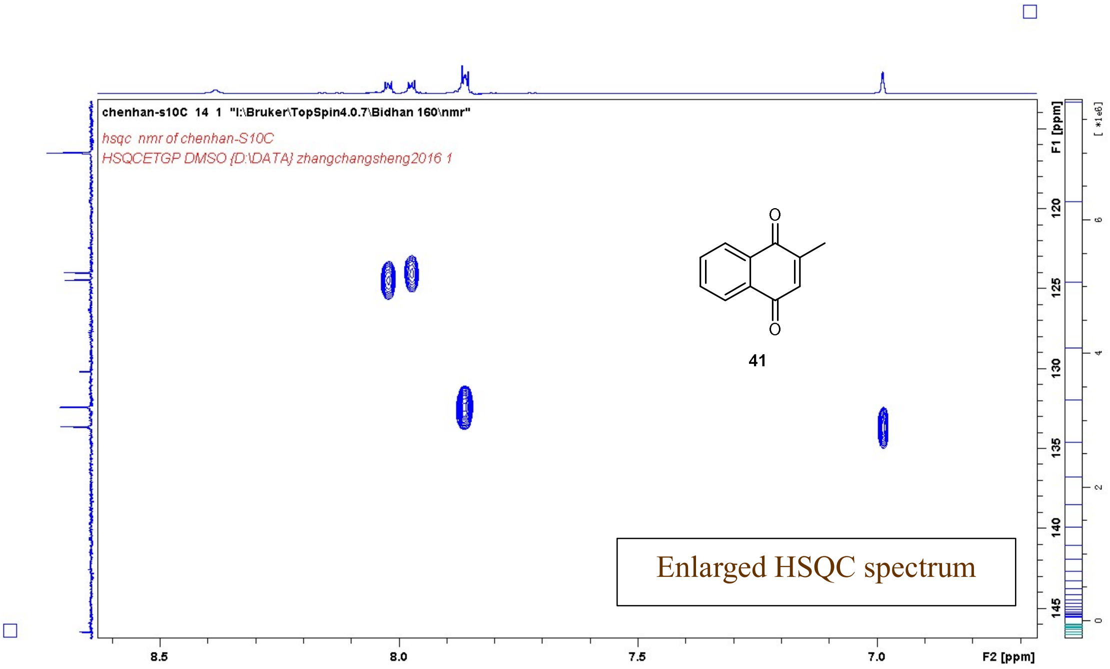

Supplementary Fig. 53 Spectroscopic data for 41. (**d**) The HSQC and enlarged HSQC spectrum of **41**in DMSO-d_6.

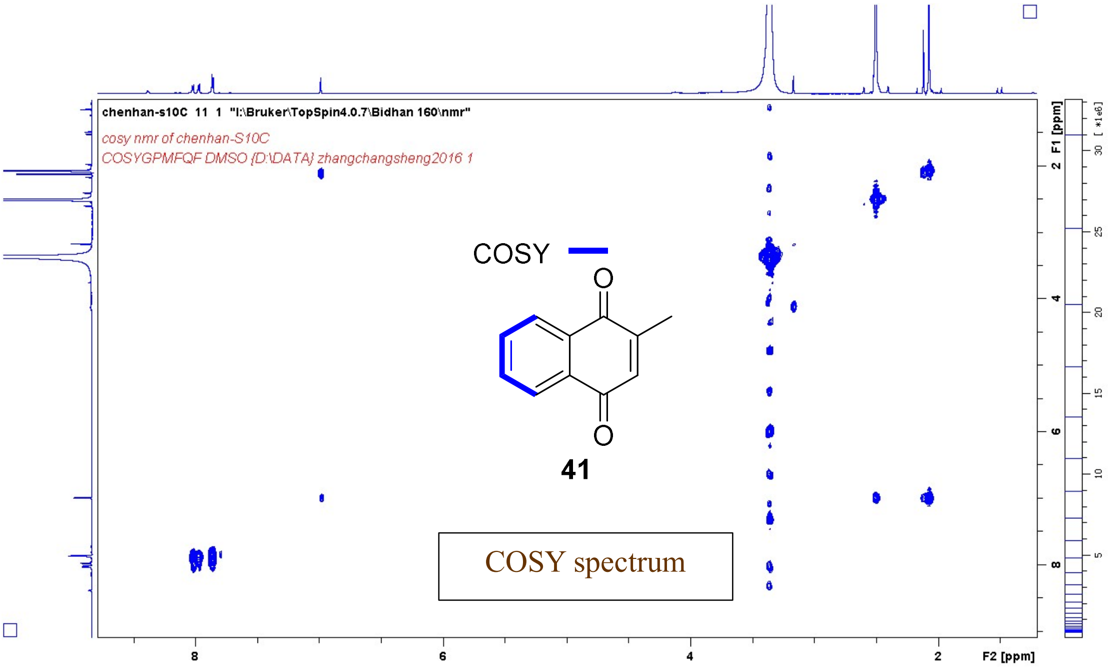

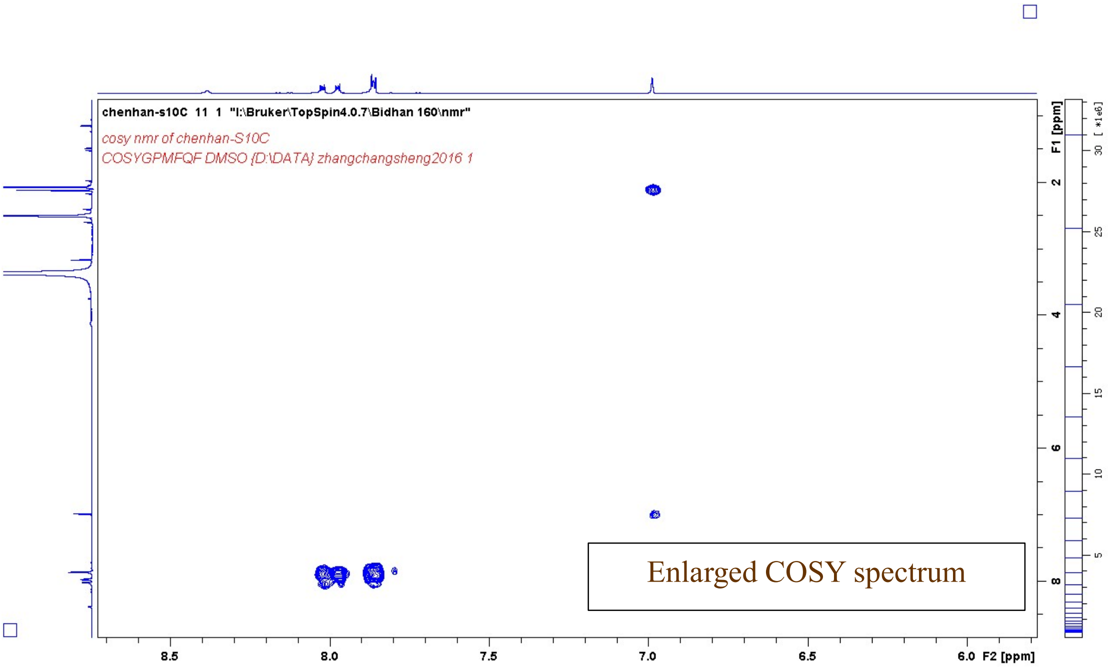

Supplementary Fig. 53 Spectroscopic data for 41. (**e**) The COSY and enlarged COSY spectrum of **41** in DMSO-d_6.

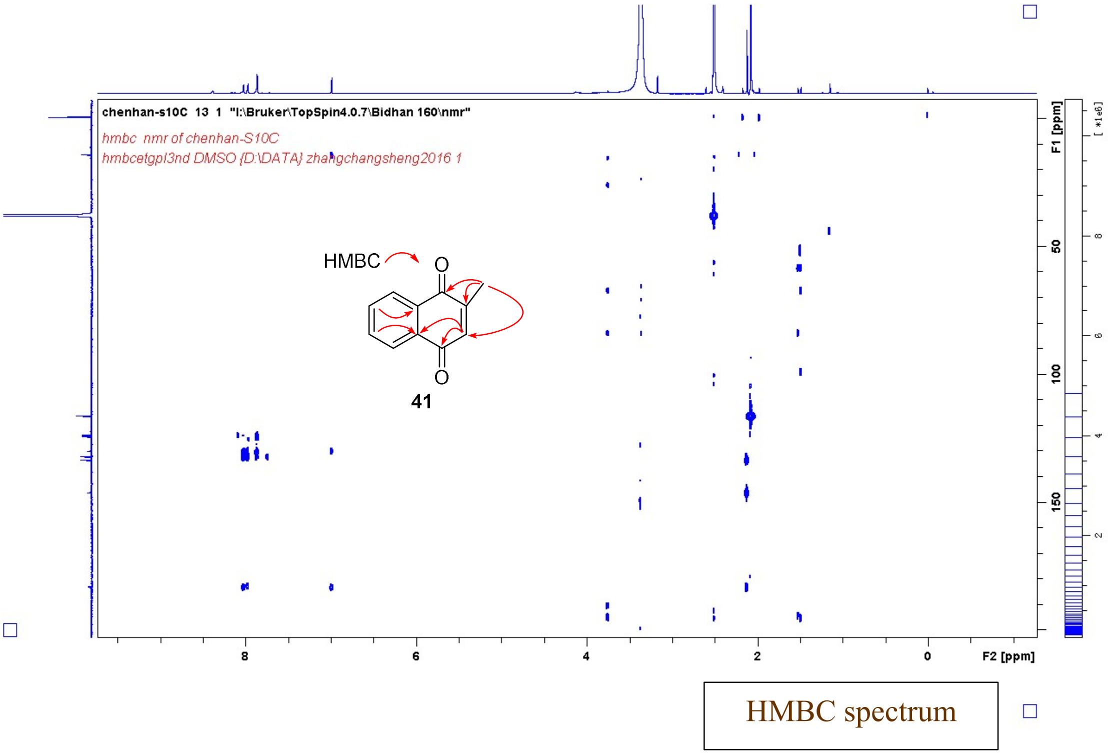

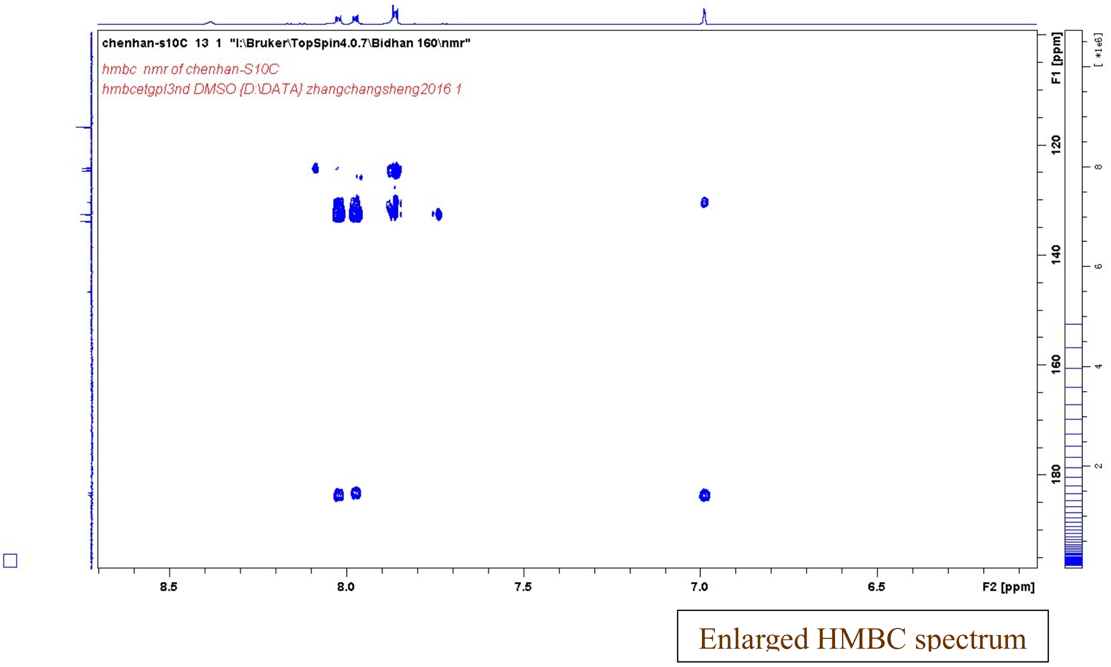

Supplementary Fig. 53 Spectroscopic data for 41. (**f**) The HMBC and enlarged HMBC spectrum of **41** in DMSO-d_6.

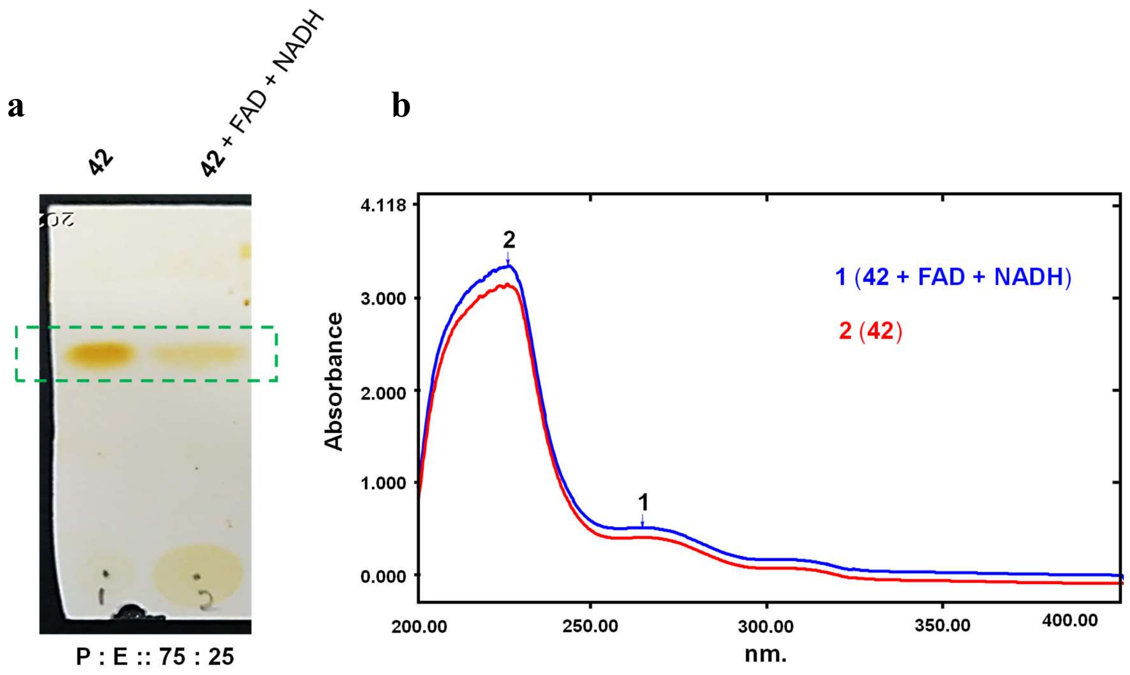

c

Substrate (**42**) $[\alpha]_D^{25}$ 19.6 (*c* 0.05, MeOH)

Reaction (**42**) $[\alpha]_D^{25}$ 19.2 (*c* 0.05, MeOH)

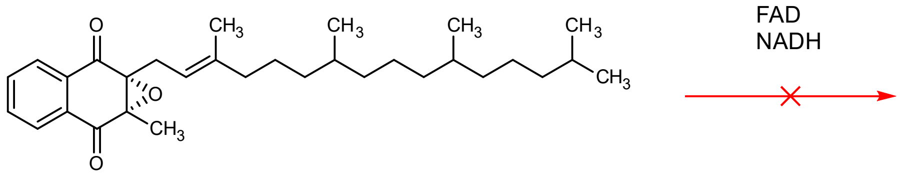

42 (Vitamin K1 2,3-epoxide)

Supplementary Fig. 54. TLC profile for the reaction, UV spectra, and Optical rotation of vitamin K1 2,3-epoxide (42) with FAD/NADH. (**a**) TLC analysis of the reaction mixtures containing 100 µM **42**, 100 µM FAD and 10 mM NADH in 50 mM PBS buffer (pH 7). (**b**) UV spectra of 100 µM **42** (substrate) and 100 µM **42** (after reaction with FAD and NADH, isolated from preparative TLC). (**c**) Optical rotation data of **42** (substrate) and 100 µM **42** (after reaction). The reaction mixtures were incubated at 30 ºC for 2 h. After ethyl acetate extraction, the crude extract was dissolved in MeOH. TLC condition: petroleum ether (P): ethyl acetate (E) (75:25).

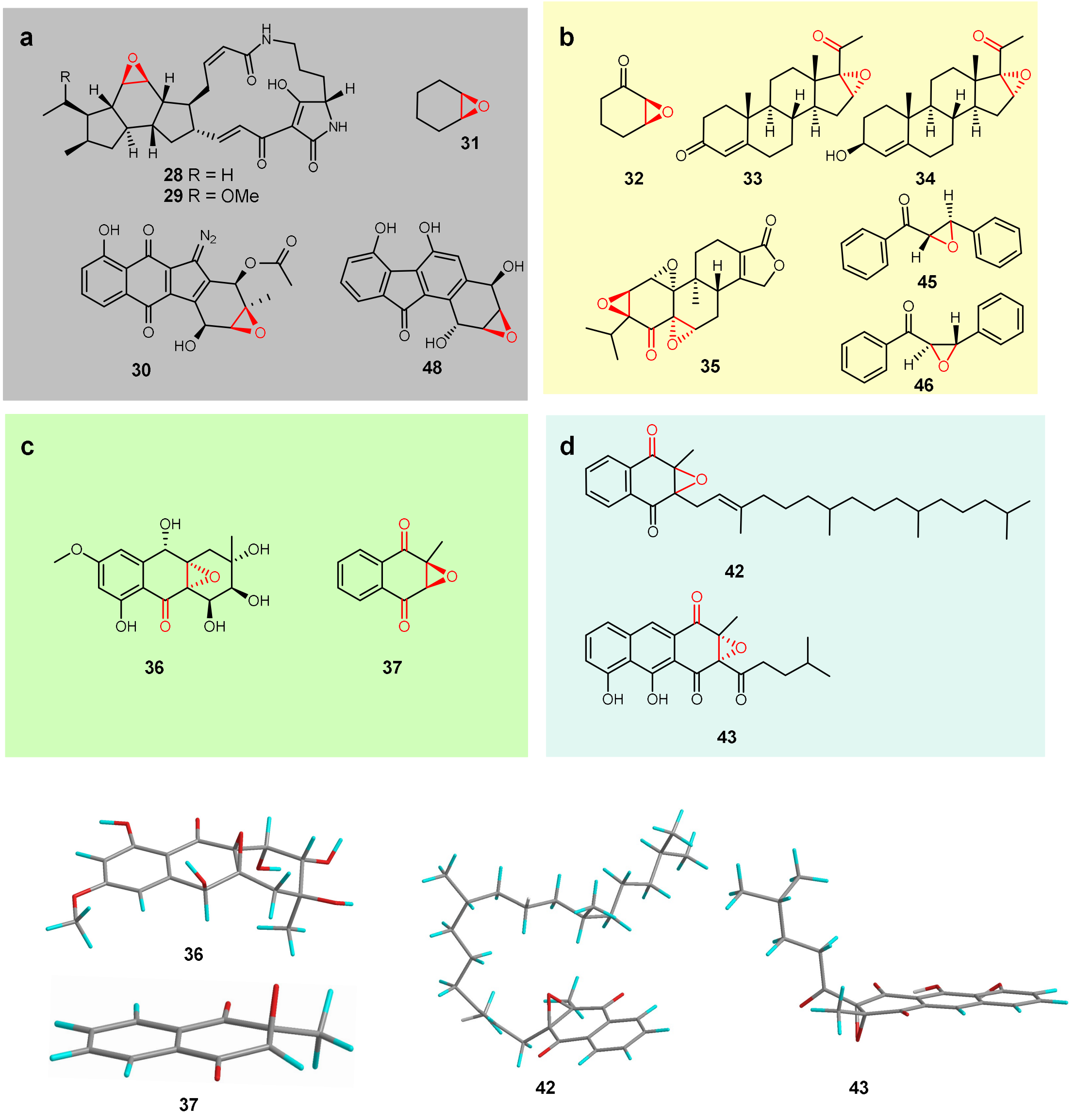

Supplementary Fig. 55. Structure features of substrated for FAD/NADH-mediated epoxide ring opening reactions. Features of substrates used for the FAD/NADH-mediated epoxide ring opening reaction. (**a**) A neighboring carbonyl group to the epoxide is absent. (**b**) An aromatic ring nearby is absent. (**c**) Substrates featuring both carbonyl and aromatic ring. (**d**). Substrates featuring both carbonyl and aromatic ring and presence of a long side chain next to the epoxide.

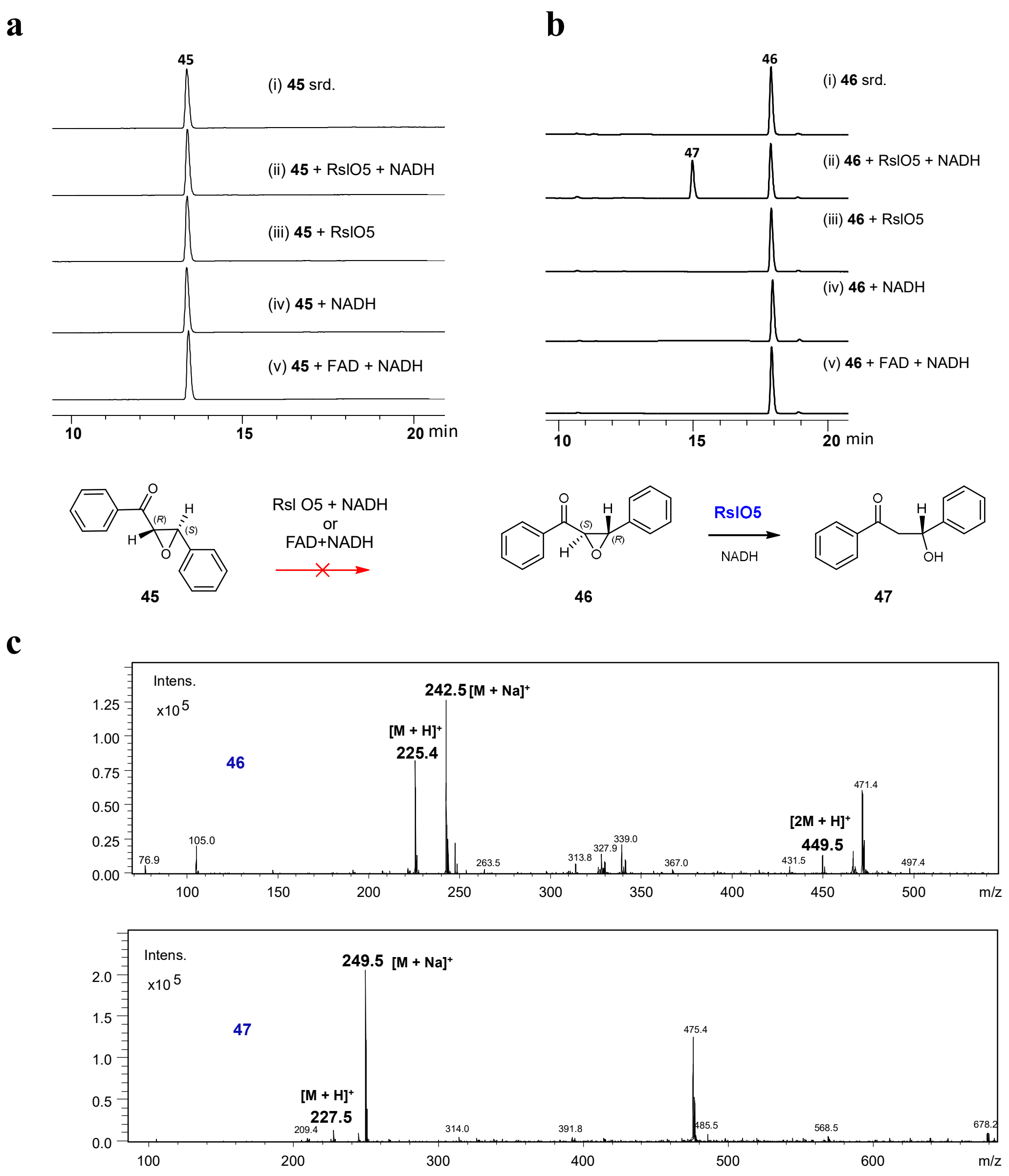

Supplementary Fig. 56. HPLC analysis of the reactions of *trans*-1,3-diphenyl-2,3-epoxypropan-1-one (45) and chalcone α,β-epoxide (46) reaction with RslO5. (**a**) HPLC analysis of reaction mixture containing (i) **45** std.; (ii) 100 μM **45** + 10 μM RslO5 and 5 mM NADH; (iii) **45** + RslO5; (iv) **45** + NADH; (v) **45** + FAD + NADH; in 50 mM PBS buffer (pH 7). The reaction mixtures were incubated at 30 ºC for 2 h. (**b**) HPLC analysis of reaction mixture containing (i) **46** std.; (ii) 100 μM **46** + 10 μM RslO5 and 5 mM NADH; (iii) **46** + RslO5; (iv) **46** + NADH; (v) **46** + FAD + NADH; in 50 mM PBS buffer (pH 7). The reaction mixtures were incubated at 30 ºC for 2 h. (**c**) LC-ESI-MS of **46** and **47**.

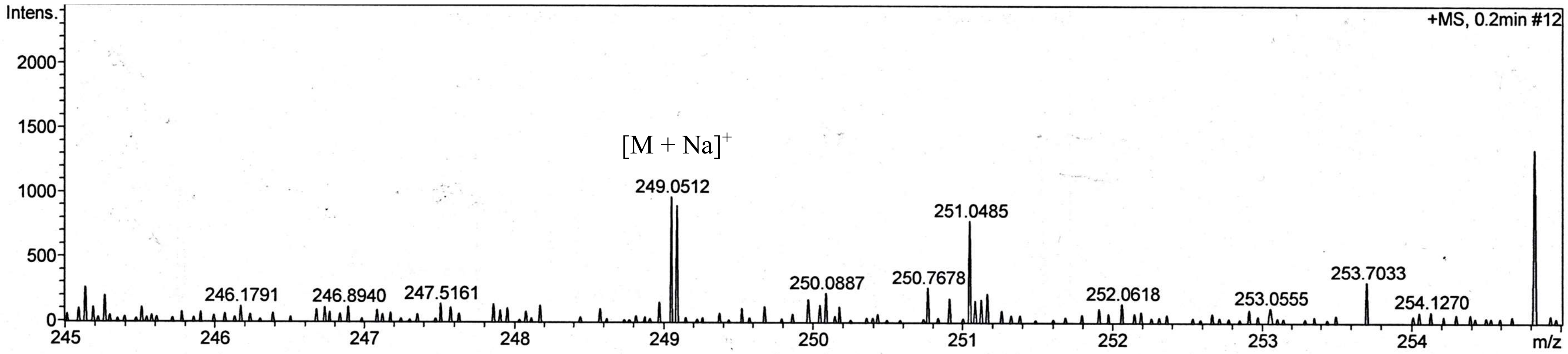

Meas. m/z	#	Ion Formula	Score	m/z	err [ppm]	err [mDa]	mSigma	rdb	e⁻ Conf	N-Rule
249.0894	1	C15H14NaO2	-1.#J	249.0886	-3.3	-0.8	n.a.	8.5	even	ok

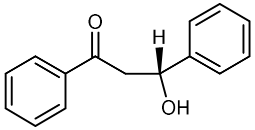

47

Chemical Formula: $C_{15}H_{14}O_2$
Exact Mass: 226.10

Supplementary Fig. 57. Spectroscopic data for 47. (**a**) HRESIMS spectrum.

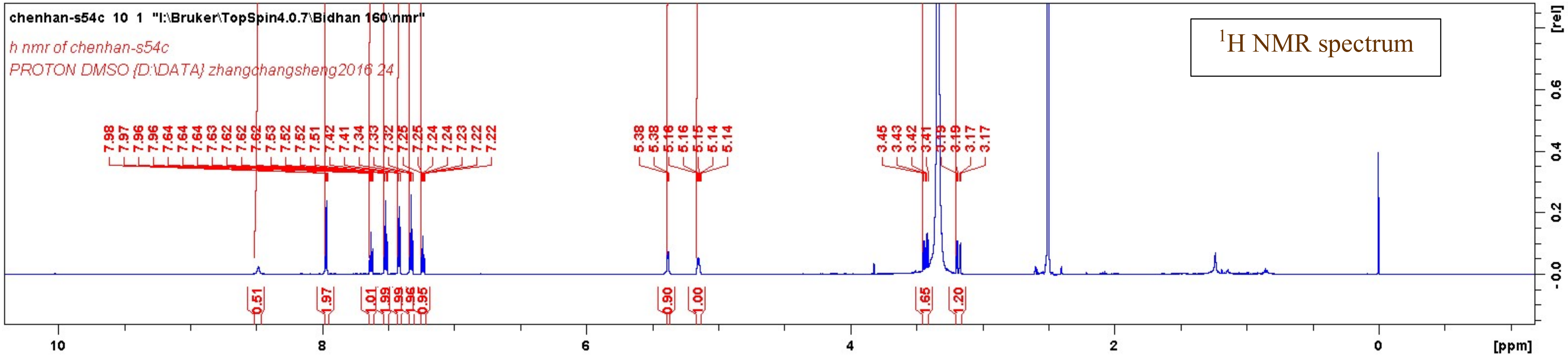

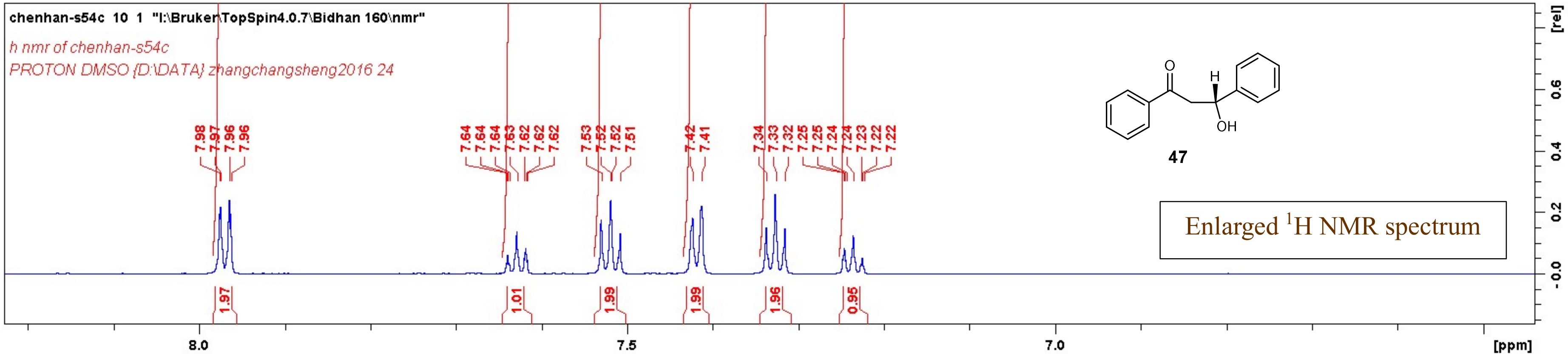

Supplementary Fig. 57. Spectroscopic data for 47. (**b**) The ^{1}H and enlarged ^{1}H NMR spectrum of **47** in DMSO-d_6.

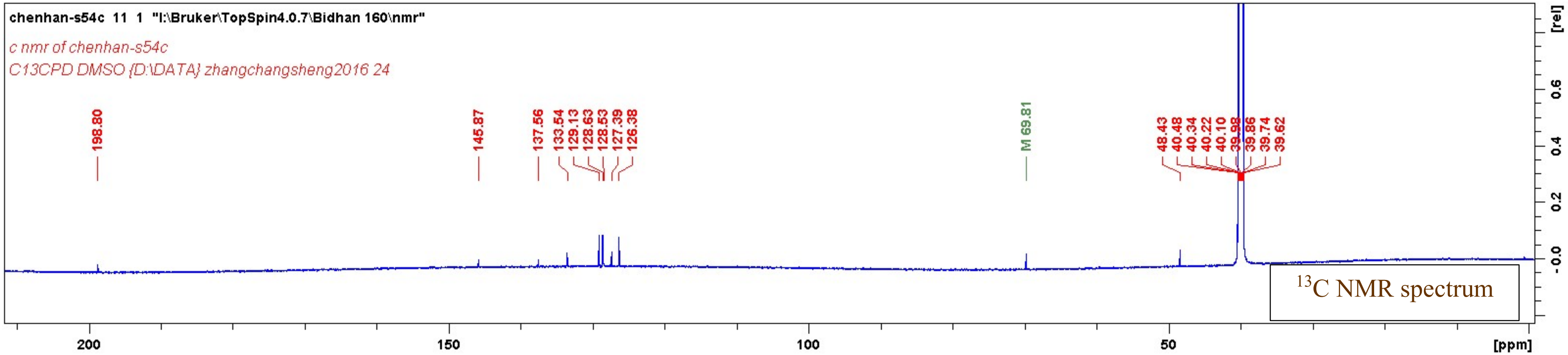

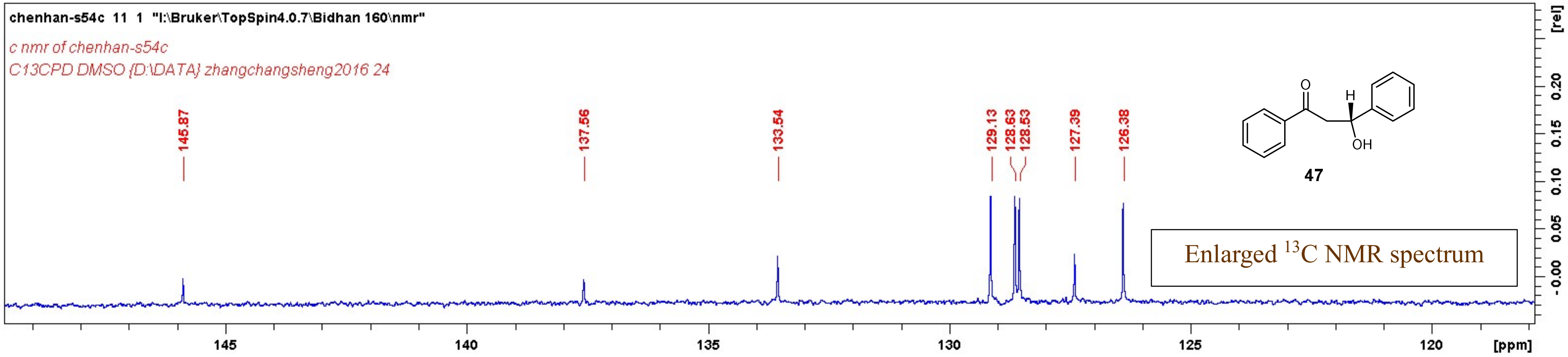

Supplementary Fig. 57. Spectroscopic data for 47. (**c**) The ^{13}C and enlarged ^{13}C NMR spectrum of **47** in DMSO-d_6.

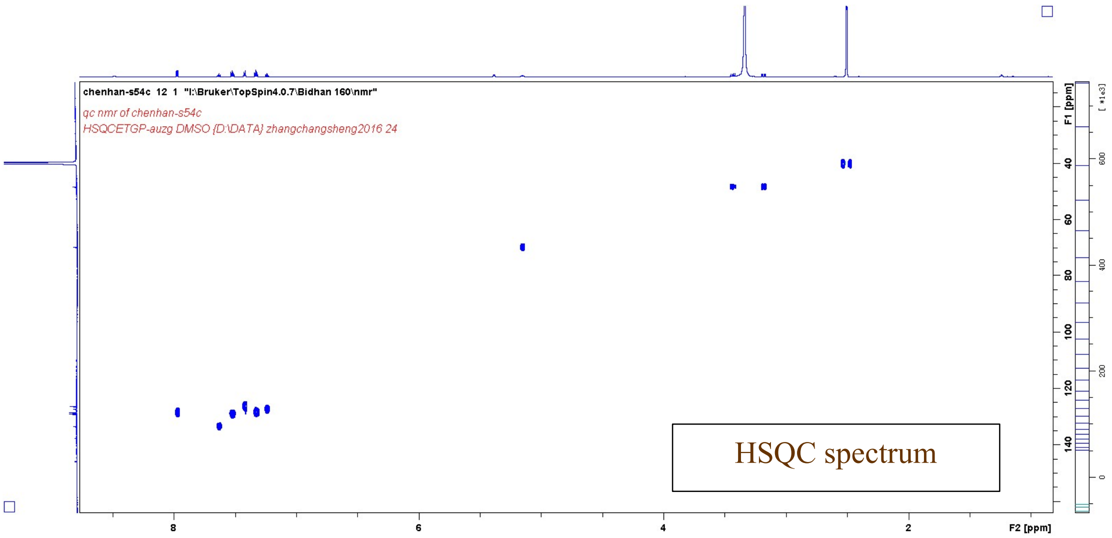

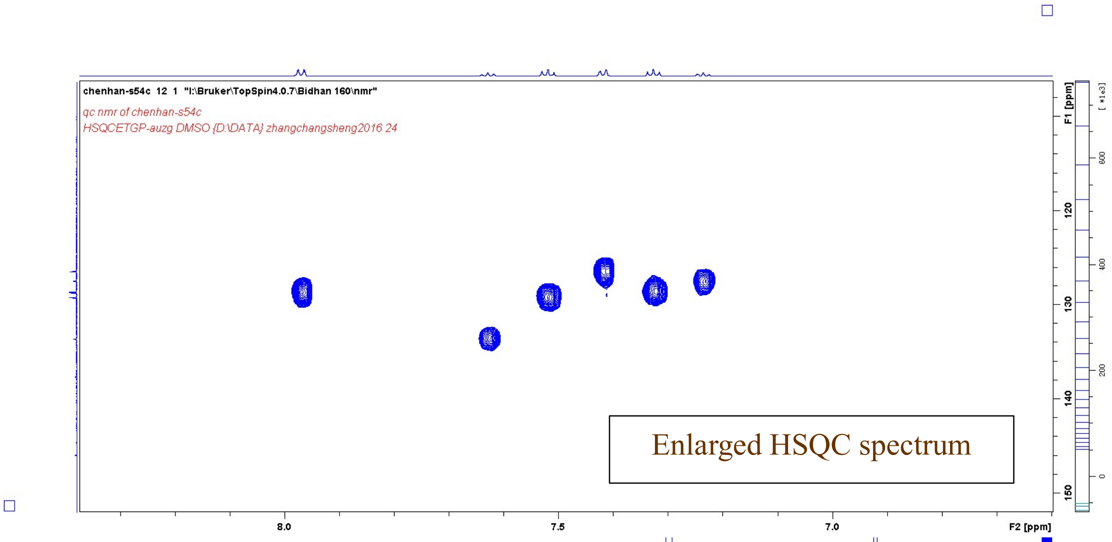

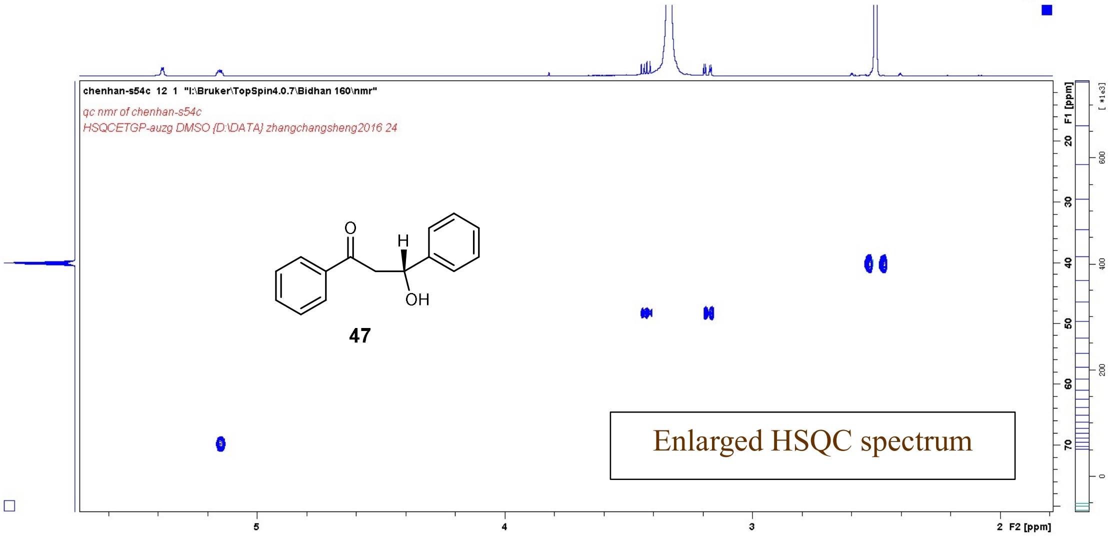

Supplementary Fig. 57. Spectroscopic data for 47. (**d**) The HSQC and enlarged HSQC spectrum of **47** in DMSO-d_6.

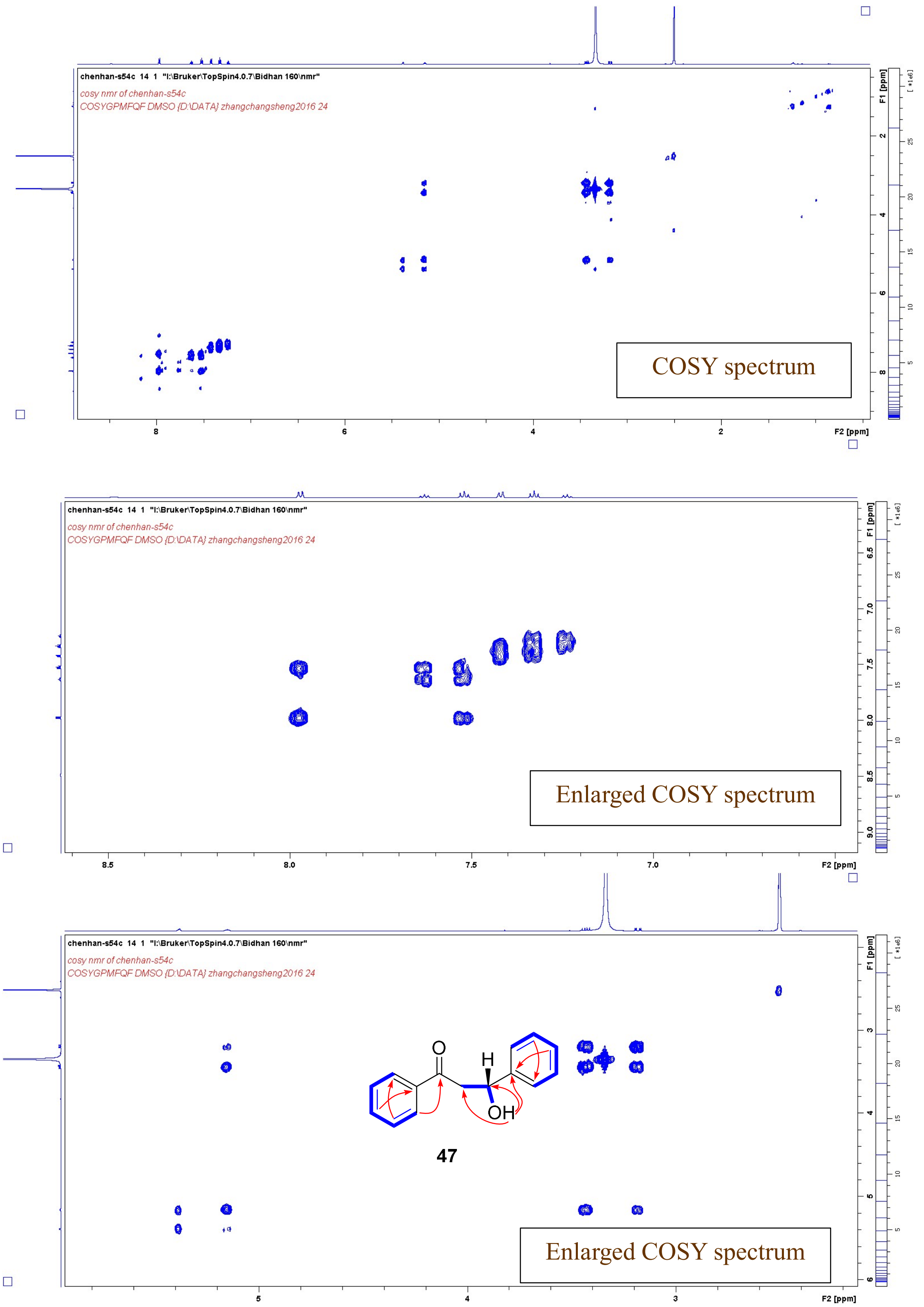

Supplementary Fig. 57. Spectroscopic data for 47. (**e**) The COSY and enlarged COSY spectrum of **47** in DMSO-d_6.

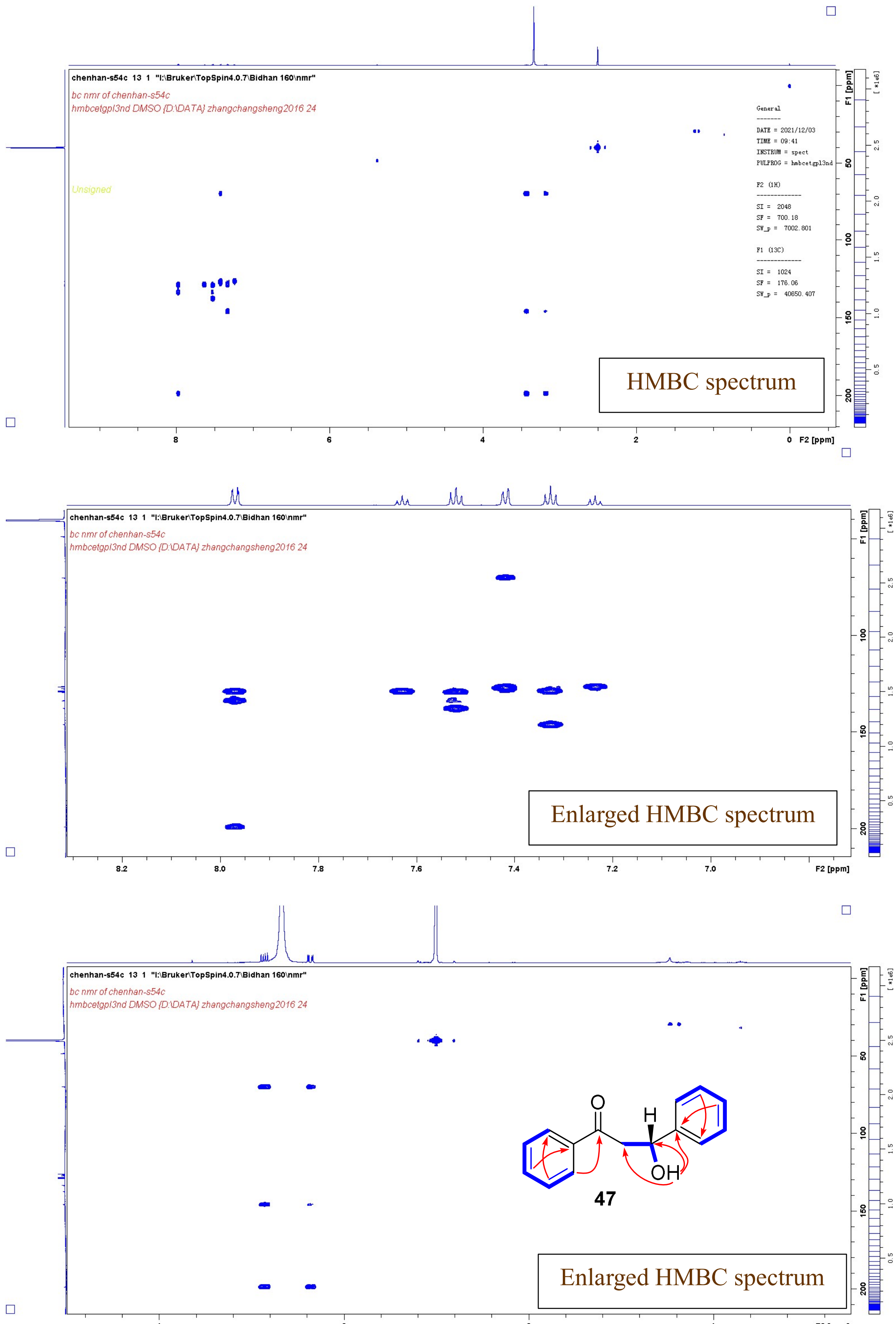

Supplementary Fig. 57. Spectroscopic data for 47. (**f**) The HMBC and enlarged HMBC spectrum of **47** in DMSO-d_6.

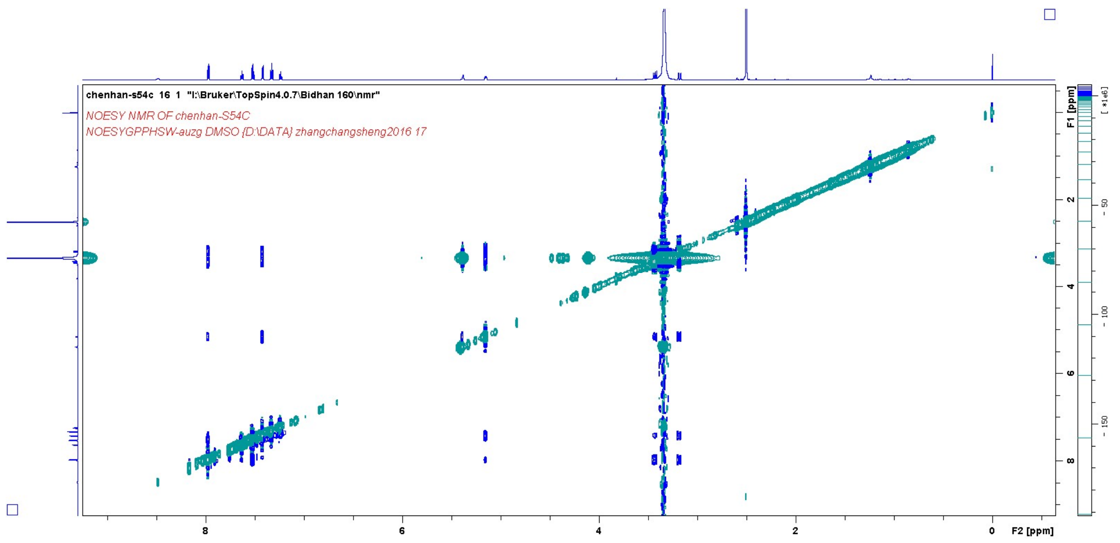

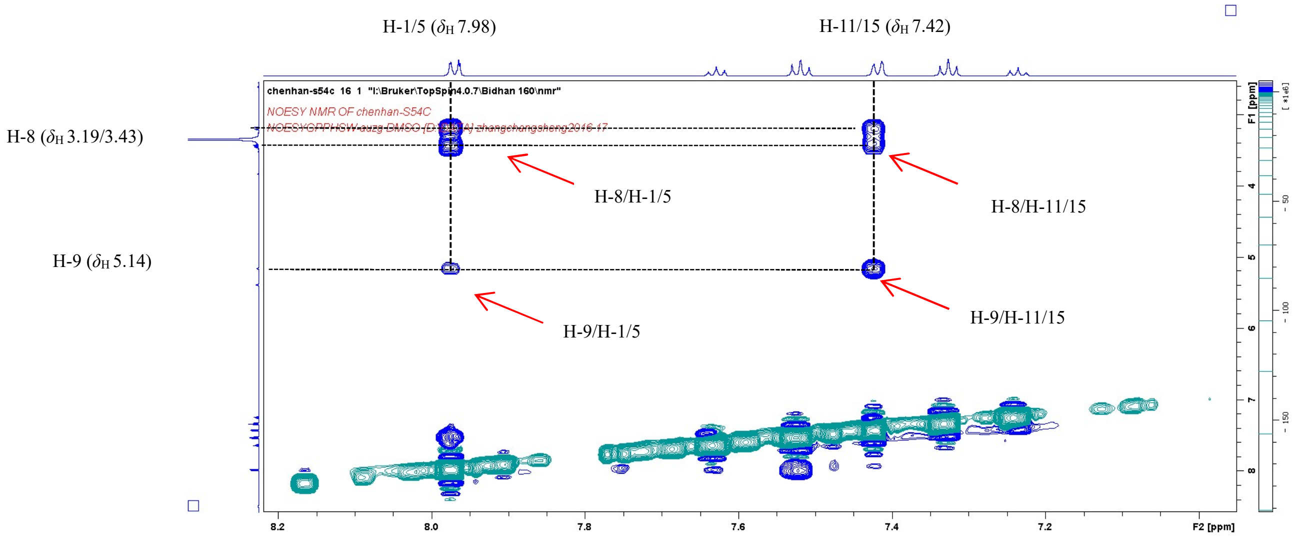

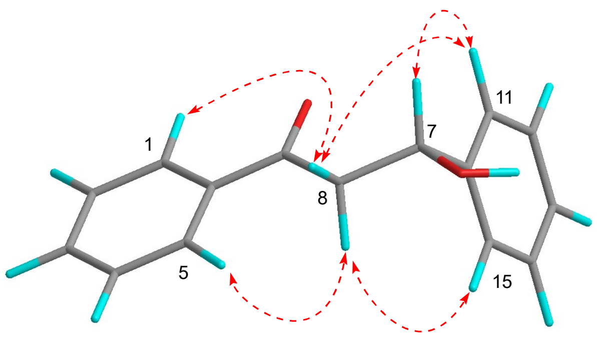

47

Supplementary Fig. 57. Spectroscopic data for 47. (**g**) The NOESY and enlarged NOESY spectrum of **47** in DMSO-d_6.

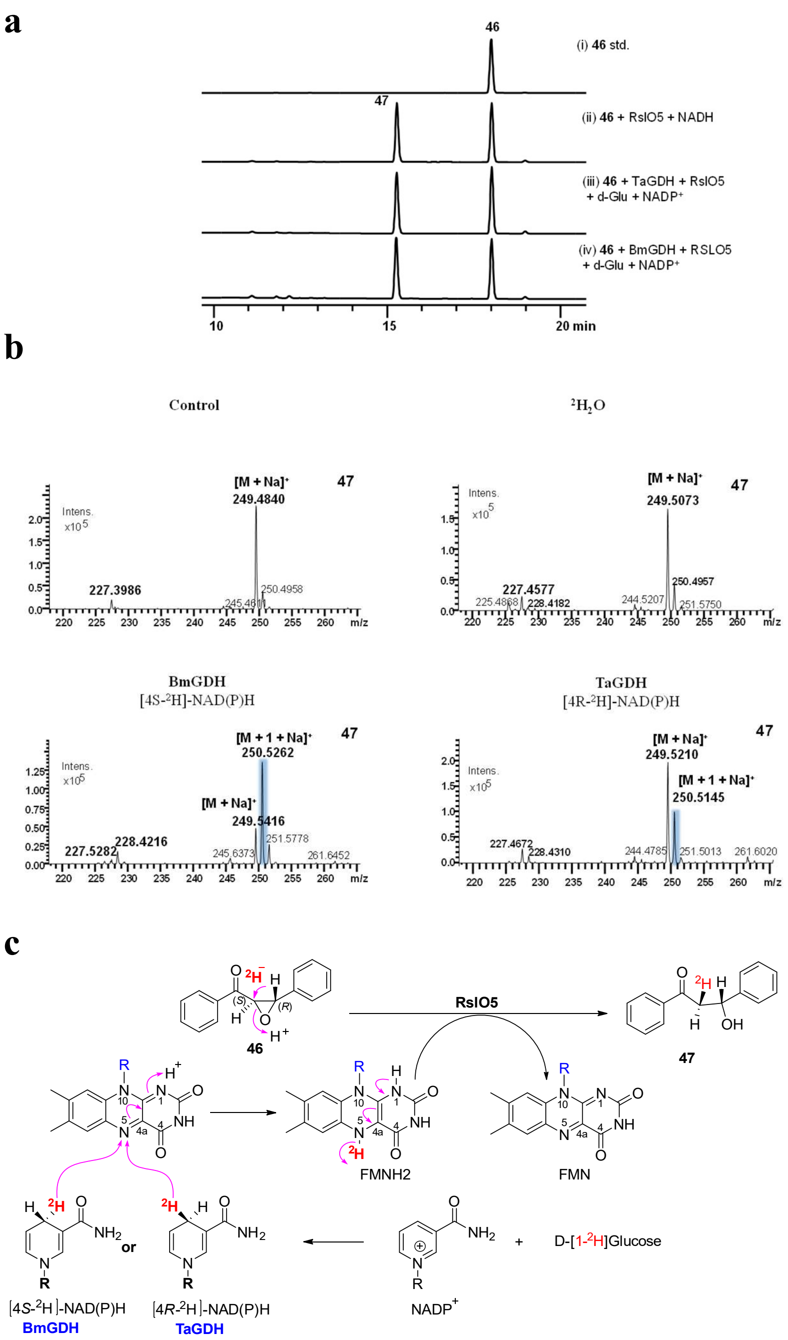

Supplementary Fig. 58. HPLC and LC-HRMS analysis of the reactions of 46 with RslO5 in coupling with GDHs. (**a**) HPLC analysis of RslO5 reactions. (i) The standard compound **46**; (ii) **46** + RslO5 + NADH reaction in PBS (pH 7) or PBS-$^{2}H_2O$; (iii) **46** + RslO5 + TaGDH + 2.5 mM $NADP^+$, and D-[1-^{2}H]glucose; iv) **46** + RslO5 + BmGDH + 2.5 mM $NADP^+$, and D-[1-^{2}H]glucose. (**b**) LC-HRMS analysis for the product **47** in 50 mM PBS buffer (pH 7.0), or 50 mM PBS buffer (pH 7) prepared with $^{2}H_2O$, [4*S*-^{2}H]-NAD(P)H, [4*R*-^{2}H]-NAD(P)H. (**c**) Schematic representation of a proposed reaction mechanism of RslO5 catalysis.

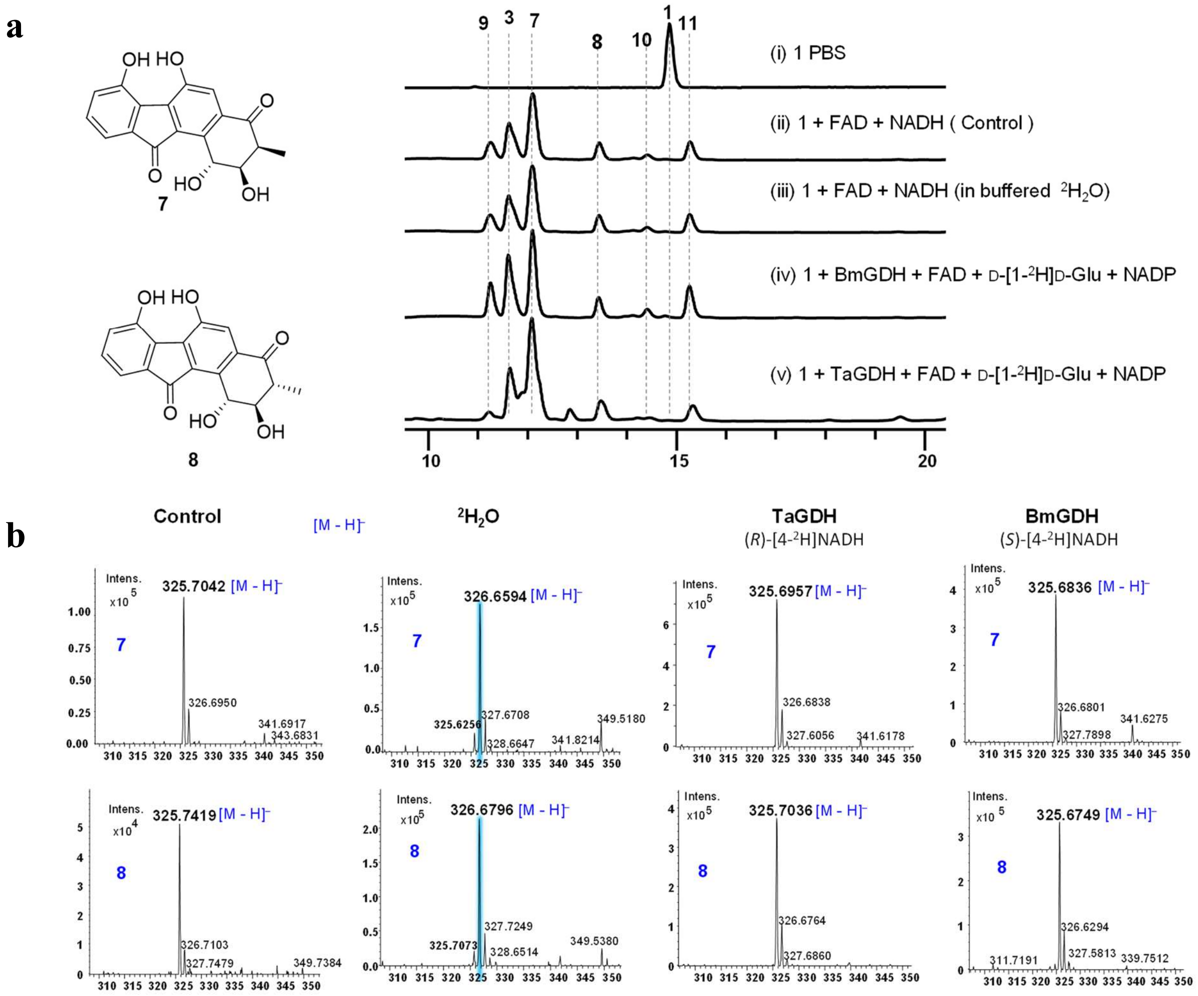

Supplementary Fig. 59. HPLC and LC-HRMS analysis of the reactions of 1 with FAD/NAD(P)H in the coupled reactions with GDHs. (**a**) HPLC analysis of reactions of **1** and FAD coupled with GDH system. (i) **1** std.; (ii) 100 µM **1** + 100 µM FAD + 2 mM NADH in 50 mM PBS buffer (pH 7); (iii) 100 µM 1 + 100 µM FAD + 2 mM NADH in 50 mM PBS buffer (pH 7) prepared with ^2H_2O; (iv) 100 µM **1** + 100 µM FAD + 10 µM BmGDH + 2.5 mM NADP, and 100 mM D-[1-^{2}H]glucose; (v) 100 µM 1 + 100 µM FAD + 10 µM TaGDH + 2.5 mM NADP, and 100 mM D-[1-^{2}H]glucose. (**b**) LC-HRMS analysis for the product **7** and **8** in 50 mM PBS buffer (pH 7), or 50 mM PBS buffer (pH 7.0) prepared with ^2H_2O, (*R*)-[4-^{2}H]NADH, (*S*)-[4-^{2}H]NADH.

a

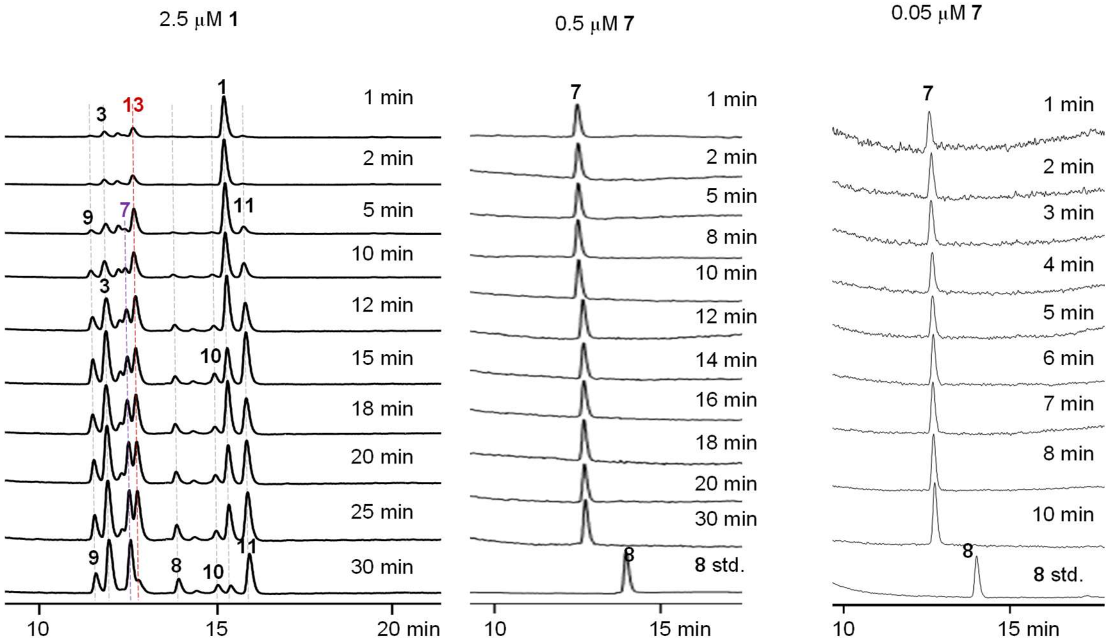

b

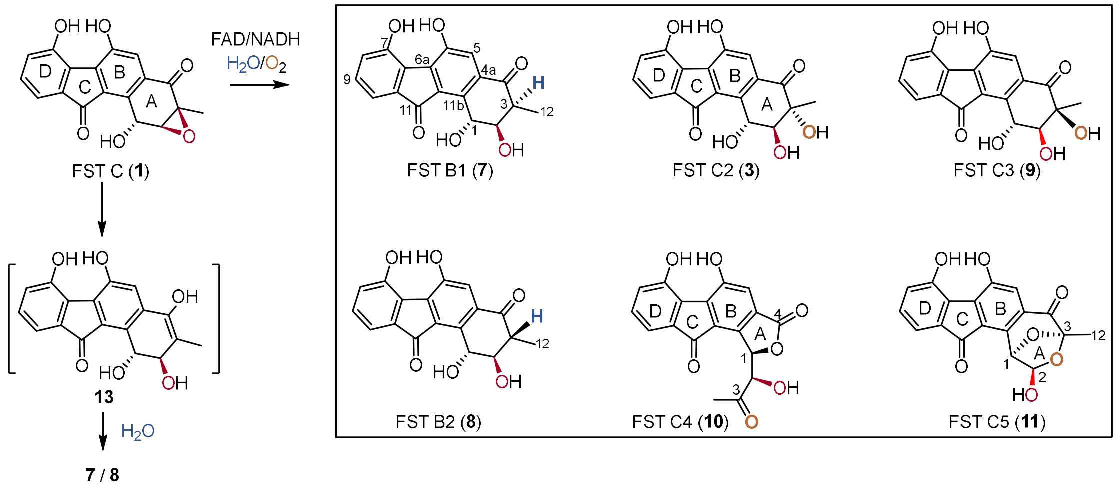

Supplementary Fig. 60. The time course assay of reduced concentration of 1 and 7 with FAD and NADH. (**a**) HPLC analysis of the time course assays of **1** or **7** with FAD and NADH. The standard assay of **1** contains 2.5 μM **1**, 10 μM FAD, 2 mM NADH in 50 mM PBS buffer (pH 7), with incubation at 30 °C and sampling at 1, 2, 5, 10, 12, 15, 18, 20, 25 and 30 min. The assay of **7** contains 0.5 μM **7**, 10 μM FAD, 2 mM NADH in 50 mM PBS buffer (pH 7), with incubation at 30 °C and sampling at 1, 2, 5, 8, 10, 12, 14, 16, 18, 20 and 30 min; or the assay contains 0.05 μM **7**, 10 μM FAD, 2 mM NADH in 50 mM PBS buffer (pH 7), with incubation at 30 °C and sampling at 1, 2, 3, 4, 5, 6, 7, 8 and 10min. HPLC analysis was performed on the Agilent 1260 Infinity series instrument, using a polar column (Comixsep®, P/N FMG-BPF5-EONU, Polar BiPFP 5u, 250 × 4.6 mm, China) with UV detection at 304 nm. (**b**) A schematic representation of the reactions. It was clearly demonstrated that the putative intermediate **13** in reactions of **1** and FAD/NADH should appear before the formation of **7**/**8**, and under the assay conditions, **7** kept unchanged and the formation of **13** from **7** was not observed in a short time.

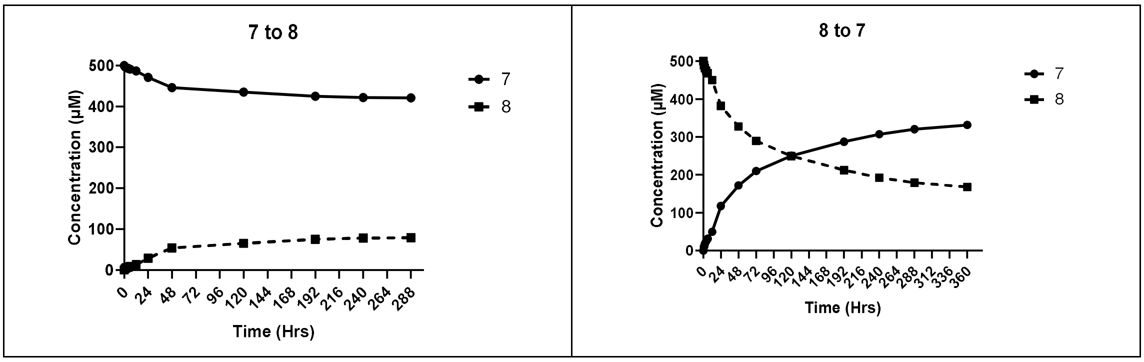

Supplementary Fig. 61. Determination of the equilibrium of the spontaneous conversion of 7 to 8 (or 8 to 7). The spontaneous assays were performed in 1 mL of 50 mM PBS buffer (pH 7.0) containing 500 μM 7 and incubated at 30 °C. 50 μL of samples were taken and stopped by addition of 50 μL of MeOH after 0, 1, 2, 4, 6, 12, 24, 48, 120, 192, 240, 288 h. The apparent $[7]_{eq}/[8]_{eq}$ was calculated as (421.7/78.9) = 5.4.

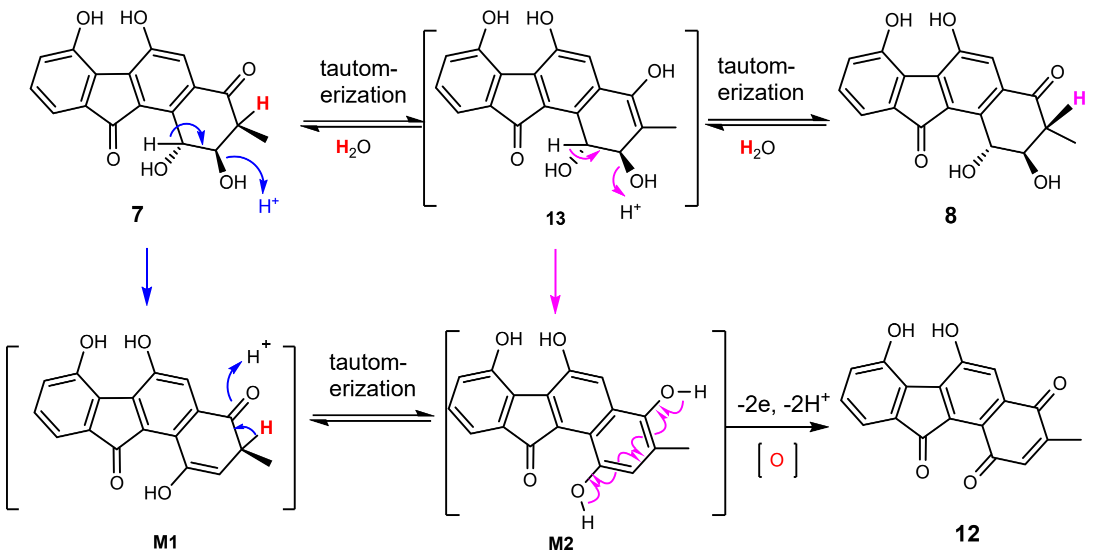

Supplementary Fig. S62. The proposed mechanism for the formation of 12 from 13 and 7.

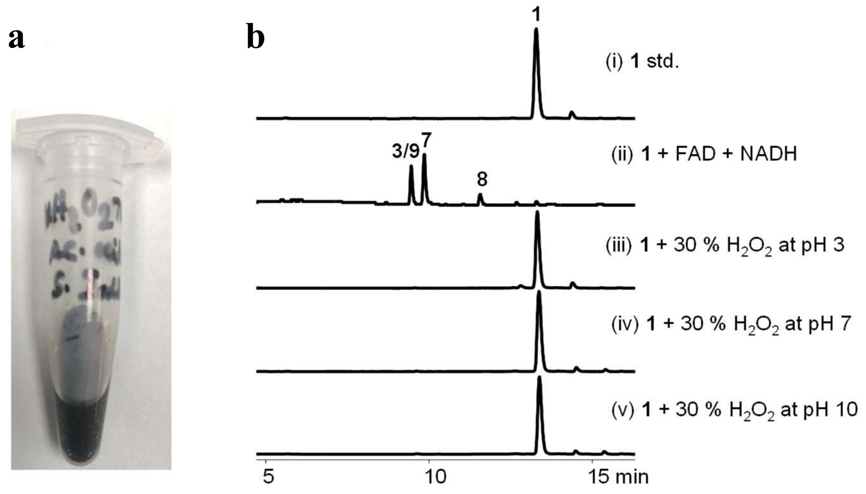

Supplementary Fig. 63. HPLC analysis of reactions of 1 with H_2O_2. (**a**) The purity of H_2O_2 was checked by chemical method containing 100 µL H_2O_2 solution with equal volume of glacial acetic acid containing about 0.1 g of sodium iodide. A brown color indicates the high concentration of peroxide. (**b**) HPLC analysis of reactions of **1**. (i) **1** std.; (ii) standard assay containing 100 µM **1**, 100 µM FAD and 10 mM NADH; (iii-v) **1** + 30 % H_2O_2 in buffers with pH 3, pH 7 and pH 10. The buffers include citric acid/Na_2HPO_4 buffer (50 mM, pH 3); PBS buffer (50 mM, pH 7); borax/NaOH buffer (50 mM, pH 10). The reaction mixtures were incubated at 30 ºC for 2 h. HPLC was run using a reversed phase C18 column.

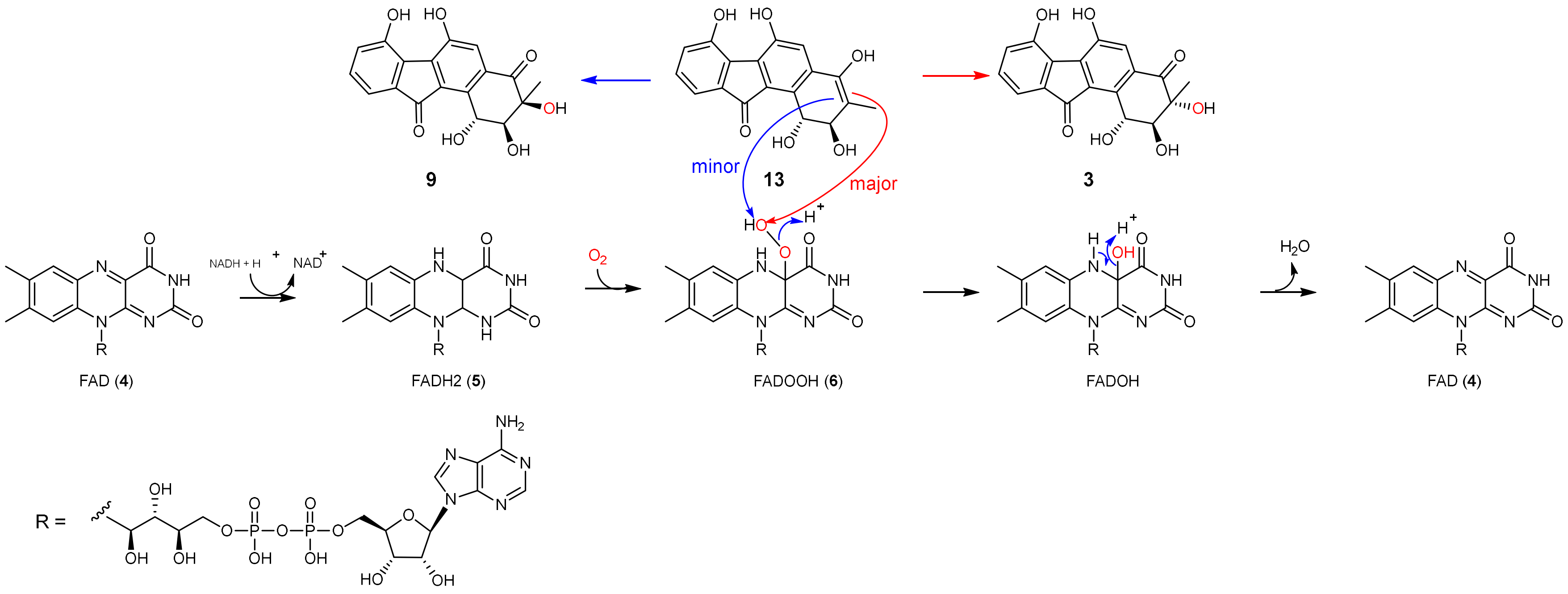

Supplementary Fig. 64. The proposed mechanism for the formation of 3 and 9 from 13.

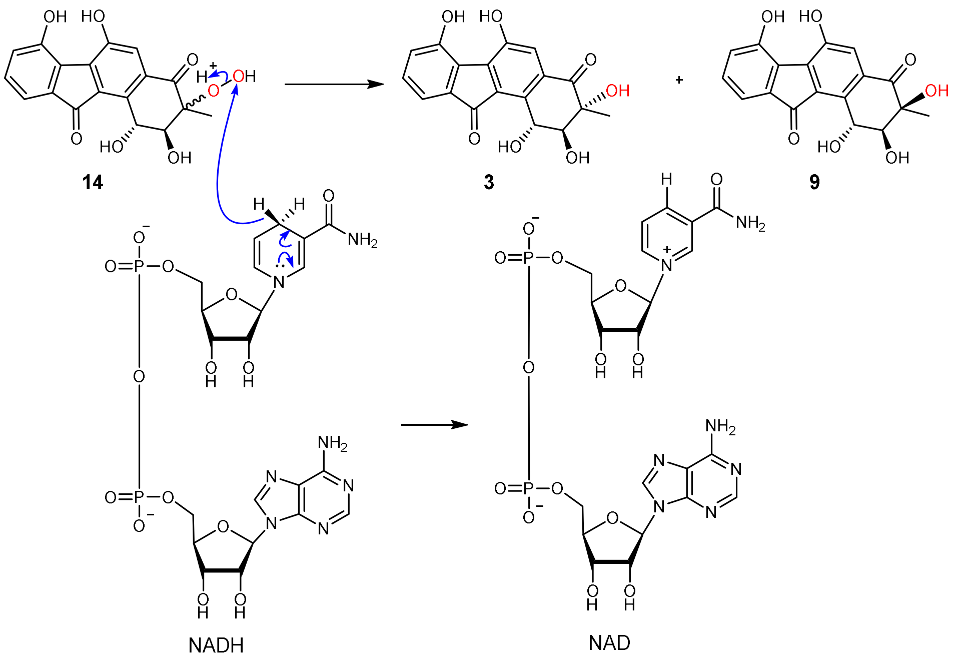

Supplementary Fig. 65. The proposed mechanism for the formation of 3 and 9 from 14.

Supplementary References

1. Zhang W*, et al.* Fluostatins I-K from the South China Sea-derived micromonospora rosaria SCSIO N160. *J Nat Prod* **75**, 1937-1943 (2012).
2. Zhang W*, et al.* Pyrazolofluostatins A-C, Pyrazole-Fused Benzo[a]fluorenes from South China Sea-Derived Micromonospora rosaria SCSIO N160. *Org Lett* **19**, 592-595 (2017).
3. Akiyama T*, et al.* Fluostatins A and B, new inhibitors of dipeptidyl peptidase III, produced by Streptomyces sp. TA-3391. I. Taxonomy of producing strain, production, isolation, physico-chemical properties and biological properties. *J Antibiot* **51**, 553-559 (1998).
4. Zhu Y*, et al.* Insights into Caerulomycin A Biosynthesis: A Two-Component Monooxygenase CrmH-Catalyzed Oxime Formation. *J Am Chem Soc* **135**, 18750-18753 (2013).
5. Huang C*, et al.* Molecular basis of dimer formation during the biosynthesis of benzofluorene-containing atypical angucyclines. *Nat Commun* **9**, 2088 (2018).
6. Zhang Q*, et al.* Characterization of the flavoenzyme XiaK as an N-hydroxylase and implications in indolosesquiterpene diversification. *Chem Sci* **8**, 5067-5077 (2017).
7. Xiao Y*, et al.* Characterization of tiacumicin B biosynthetic gene cluster affording diversified tiacumicin analogues and revealing a tailoring dihalogenase. *Journal of the American Chemical Society* **133**, 1092-1105 (2011).
8. Zhang G*, et al.* Mechanistic insights into polycycle formation by reductive cyclization in ikarugamycin biosynthesis. *Angew Chem Int Ed Engl* **53**, 4840-4844 (2014).
